# Supplementary material for: Apoptotic Cells induce Proliferation of Peritoneal Macrophages
Source: Int J Mol Sci. 2021 Feb 24;22(5):2230. doi: 10.3390/ijms22052230 (PMC7956251; doi:10.3390/ijms22052230)
Supplement: Supplementary file 1 [file ijms-22-02230-s001.pdf]

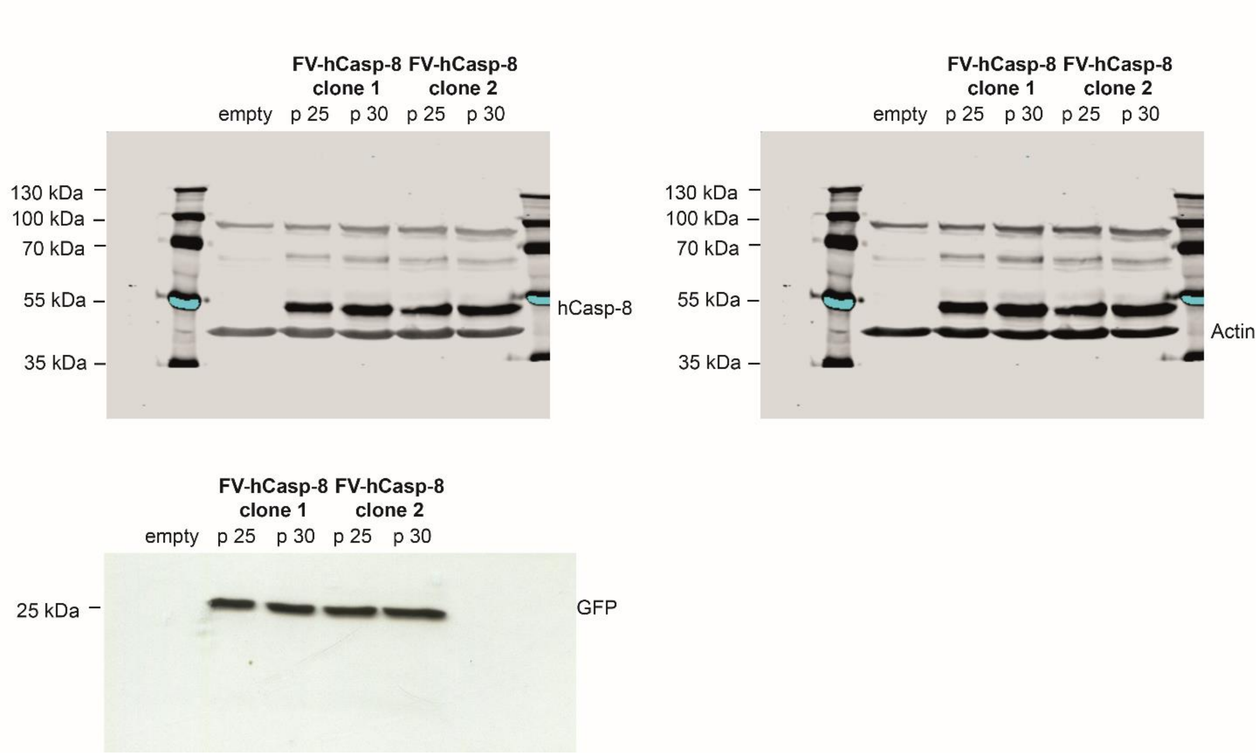

Figure S1. Uncropped Western Blot of Figure 1B. Uncropped images of Western Blots of Figure 1B are shown.

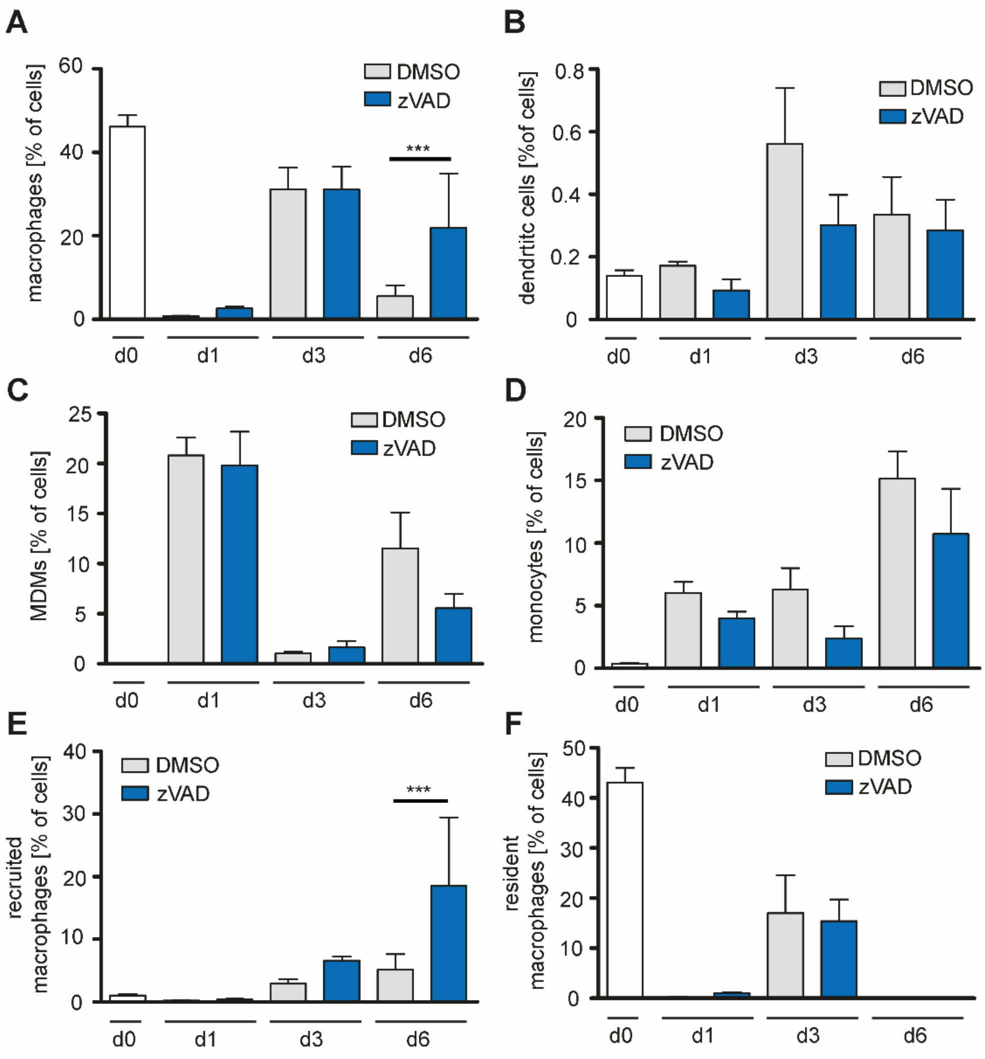

Figure S2. Immune cells of the lavage were analyzed. (**A**) Macrophages were defined as $CD45^+$CD11b F4/80 and percentage of macrophages in the lavage was determined by flow cytometry (n=3-9). (**B**) Dendritic cells were defined as $CD45^+$CD11b CD11c MHCII and percentage of dendritic cells in the lavage was determined by flow cytometry (n=3-9). (**C**) Monocyte derived macrophages (MDMs) were defined as $CD45^+$CD11b F4/80 Ly-6C and percentage of MDMs in the lavage was determined by flow cytometry (n=3-9). (**D**) Monocytes were defined as $CD45^+$CD11b Ly-6C and percentage of monocytes in the lavage was determined by flow cytometry (n=3-9). (**E**) Recruited macrophages were defined as $CD45^+$CD11b F4/80 MHCII low and percentage of recruited macrophages in the lavage was determined by flow cytometry (n=3-9). (**F**) Resident macrophages were defined as $CD45^+$CD11b F4/80 MHCII high and percentage of resident macrophages in the lavage was determined by flow cytometry (n=3-9). Data are shown as mean ± SEM. ***$p<0.001$.

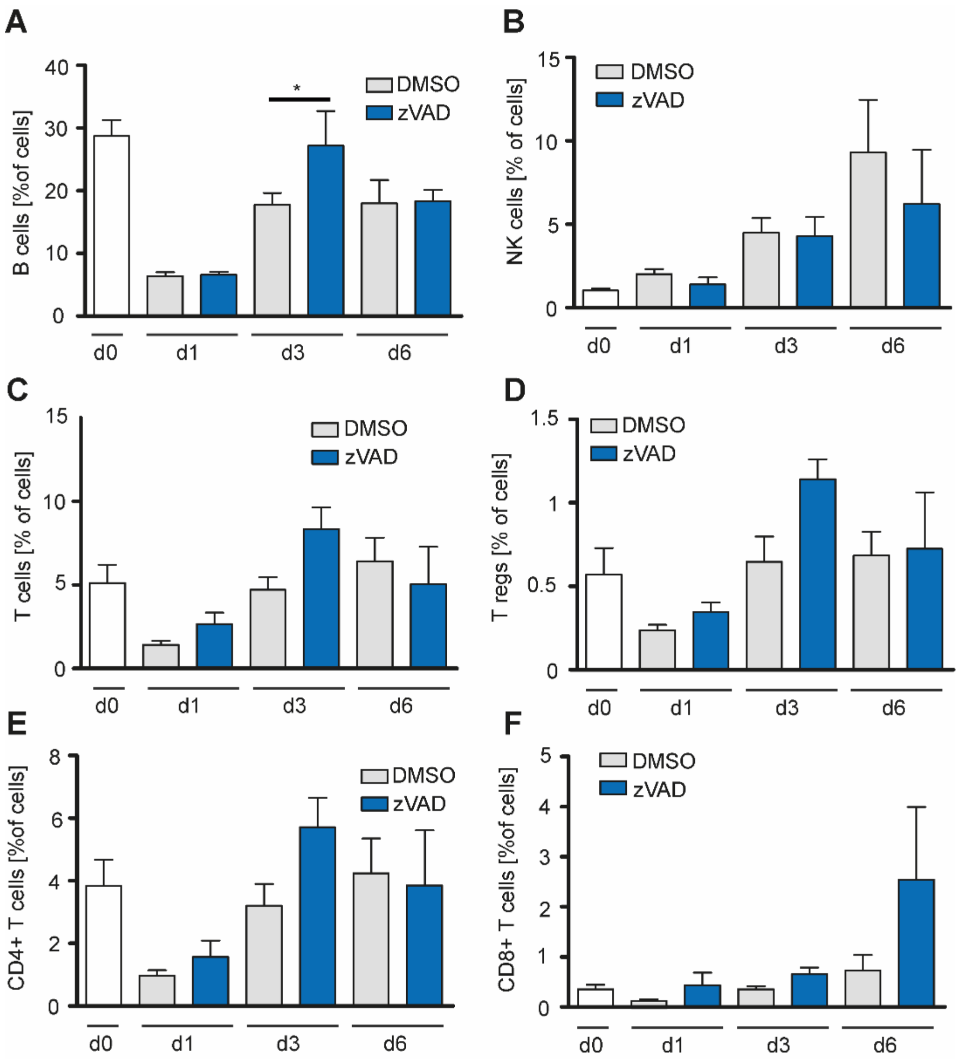

Figure S3. Immune cells of the lavage were analyzed (continued). (**A**) B cells were defined as CD45^{+}Ly-6G MHCII and percentage of B cells in the lavage was determined by flow cytometry (n=3-9). (**B**) NK cells were defined as CD45^{+}NK1.1 and percentage of NK cells in the lavage was determined by flow cytometry (n=3-9). (**C**) T cells were defined as CD45^{+}CD3 and percentage of T cells in the lavage was determined by flow cytometry (n=3-9). (**D**) Regulatory T cells (T regs) were defined as CD45^{+}CD3 CD4 CD34/CD117 and percentage of T regs in the lavage was determined by flow cytometry (n=3-9). (**E**) CD4+ T cells were defined as CD45^{+}CD3 CD4 and percentage of CD4+ T cells in the lavage was determined by flow cytometry (n=3-9). (**F**) CD8+ T cells were defined as CD45^{+}CD3 CD8 and percentage of CD8+ T cells in the lavage was determined by flow cytometry (n=3-9).

Table S1. Log2fold changes of genes after RNA sequencing with base mean >0 and p-value<0.05 are listed.

Gene	baseMean	log2FoldChange	lfcSE	stat	pvalue	padj
37500	1397.114049	0.239944253	0.094512616	2.538753687	0.011124812	0.049657956
39326	3380.68419	0.36398768	0.076839966	4.736957826	2.17E-06	2.91E-05
40057	273.8579169	-0.391199025	0.125181561	-3.125053084	0.00177773	0.010906946
0610009L18Rik	25.58035775	-0.825930237	0.268496223	-3.076133543	0.002097039	0.012542951
0610010F05Rik	400.2764227	0.263678146	0.11174755	2.35958772	0.018295255	0.073817418
0610012G03Rik	641.5156499	0.226771774	0.095082824	2.384992002	0.017079493	0.069839767
0610030E20Rik	403.1706068	-0.225624387	0.098792085	-2.283830609	0.022381488	0.08689726
1110004F10Rik	700.3038362	0.231369822	0.10523852	2.198527895	0.027911509	0.102998442
1110025M09Rik	64.37754349	-0.827816886	0.205559939	-4.027131416	5.65E-05	0.000545952
1110032A03Rik	100.0123685	-0.32355344	0.157044661	-2.060263864	0.039373322	0.133572446
1110038B12Rik	222.5524035	0.259039532	0.128651961	2.013490742	0.044063028	0.144563094
1110059E24Rik	219.0928955	0.324376997	0.122188951	2.654716284	0.007937514	0.038056955
1500004A13Rik	9.495922323	-0.782271232	0.356876978	-2.191991302	0.028380135	0.104381022
1600010M07Rik	51.956614	-0.420601267	0.205330949	-2.048406578	0.040520178	0.13643664
1600014C10Rik	167.8153889	0.481187675	0.185224797	2.597857759	0.009380734	0.043169789
1700017B05Rik	562.0584976	0.295187792	0.105687052	2.793036477	0.00522158	0.027104897
1700018L02Rik	4.586989523	-1.09291585	0.381362574	-2.865818318	0.004159329	0.022444821
1700028E10Rik	26.11892296	-0.575996056	0.264755799	-2.175574845	0.029587066	0.108032384
1700037H04Rik	321.0768751	0.894996651	0.136397676	6.561670828	5.32E-11	1.48E-09
1700047M11Rik	56.89882137	-1.253637458	0.25907936	-4.8388164	1.31E-06	1.84E-05
1700056E22Rik	3.9242939	-0.752295698	0.380523391	-1.977002508	0.048041347	0.153966418
1700061G19Rik	10.27322932	-0.791720885	0.365202423	-2.167896039	0.030166597	0.10972131
1700066M21Rik	63.60108135	0.460415751	0.191198188	2.408054993	0.016037765	0.066357524
1810026B05Rik	175.7319933	-0.391320848	0.145008171	-2.698612391	0.006962922	0.034316116
1810037I17Rik	558.7505354	0.313360622	0.093846687	3.339069621	0.000840595	0.00573928
1810058I24Rik	1013.177572	-0.305475853	0.100840883	-3.029285772	0.002451327	0.014308465
2210408F21Rik	202.5877627	-0.332246083	0.131050022	-2.535261551	0.01123634	0.050054159
2300009A05Rik	193.2531425	-0.327225934	0.116044512	-2.819831188	0.004804892	0.025282964
2310001H17Rik	77.93685028	-0.494264513	0.213866633	-2.311087546	0.02082802	0.081995302
2310009A05Rik	300.1591139	0.222682444	0.102684372	2.168610863	0.03011224	0.109557675
2310010J17Rik	91.92131794	-0.517581606	0.157120248	-3.294175078	0.00098711	0.006595529
2310030G06Rik	6.536174249	-1.175268121	0.376486813	-3.121671413	0.001798275	0.011010492
2310043M15Rik	1.146078183	0.616382384	0.302638901	2.03669251	0.041680866	
2410080I02Rik	3.448334909	-0.845629263	0.375302786	-2.253192078	0.024247041	0.092611754
2510009E07Rik	502.6305969	0.224548457	0.08837548	2.54084569	0.011058471	0.049425073
2510039O18Rik	721.4312737	0.346740229	0.088646541	3.911491924	9.17E-05	0.000839365
2610001J05Rik	700.4304672	0.385312314	0.12200959	3.158049403	0.001588286	0.009943852
2610203C22Rik	105.38155	0.391799723	0.176090368	2.224992357	0.026081751	0.097926306
2610507B11Rik	1187.080378	0.585478453	0.096026554	6.097047385	1.08E-09	2.55E-08
2700099C18Rik	24.0632511	1.321893289	0.290744088	4.546586998	5.45E-06	6.75E-05
2810004N23Rik	487.8289721	0.329251659	0.116562702	2.82467422	0.004732871	0.025000421

2810013P06Rik	150.113628	-0.412642584	0.141988962	-2.906159587	0.003658948	0.020127063
2810025M15Rik	206.9928203	0.69498684	0.132887591	5.229885163	1.70E-07	2.77E-06
2810408I11Rik	12.68898638	1.365159286	0.360834364	3.783340565	0.000154737	0.001326182
2900026A02Rik	20.82640319	0.941974886	0.299465247	3.145523208	0.0016579	0.010302324
2900076A07Rik	34.16999429	-0.567705264	0.250938655	-2.262326882	0.023677215	0.090922201
2900089D17Rik	72.36684661	-0.584675869	0.207553138	-2.816993636	0.004847549	0.02546278
3110082I17Rik	145.9721175	0.638146697	0.145981765	4.371413767	1.23E-05	0.000142393
3830406C13Rik	351.1697278	0.309244062	0.101884924	3.03522887	0.002403532	0.014051347
4833407H14Rik	3.57297616	0.997817062	0.378226334	2.638148044	0.008336018	0.039550624
4930404I05Rik	2.872227875	-0.726228053	0.362162559	-2.005254366	0.044935861	0.146821786
4930452B06Rik	1.52204998	-0.760033181	0.334096997	-2.274887793	0.022912662	
4930455G09Rik	376.9452104	0.397113285	0.126960655	3.127845284	0.001760928	0.010821562
4930481A15Rik	13.60175227	-0.788797527	0.335730558	-2.349495772	0.018798858	0.075451847
4930486L24Rik	29.31303079	1.277717333	0.26197327	4.877281298	1.08E-06	1.54E-05
4930518I15Rik	33.56107618	-0.700314407	0.267746066	-2.615591774	0.008907298	0.04154191
4930526A20Rik	2.927738655	1.193288228	0.369918317	3.225815465	0.001256143	0.008104446
4930555A03Rik	18.80031517	0.690033862	0.299811369	2.301560027	0.021359997	0.083585259
4930579G24Rik	10.40725269	0.970005994	0.353926897	2.740695896	0.006130922	0.031030944
4931406C07Rik	492.4928754	0.199075987	0.094875255	2.098291992	0.035879359	0.124957426
4933404O12Rik	15.65074007	0.877392163	0.319384086	2.747138016	0.006011783	0.030510256
4933440N22Rik	51.05748285	-0.597156815	0.218596218	-2.731780181	0.006299315	0.031648419
5031425E22Rik	193.3260273	-0.284281317	0.125864315	-2.258633164	0.023906213	0.091590641
5031439G07Rik	1803.923291	-0.290646445	0.080187013	-3.624607457	0.000289401	0.002313576
5330406M23Rik	67.77110123	-0.792741521	0.228852006	-3.463992009	0.000532222	0.00388854
5330438D12Rik	17.27733418	-0.74495422	0.304210392	-2.448812531	0.014332803	0.060606472
5430427O19Rik	208.2067266	0.622650216	0.132695367	4.69232823	2.70E-06	3.54E-05
5830415G21Rik	14.21497351	-0.976529393	0.34557918	-2.825776115	0.004716622	0.024932139
5930430L01Rik	31.3960154	-0.862153663	0.261123482	-3.301708663	0.000960978	0.006441001
6030400A10Rik	50.79893553	-0.521619389	0.222210182	-2.347414439	0.018904214	0.07575315
6330562C20Rik	72.1607478	-0.548149617	0.204252762	-2.683682761	0.007281616	0.035548217
6430548M08Rik	759.9492646	0.268099847	0.079810528	3.35920402	0.000781673	0.005393366
6530402F18Rik	30.38193248	-0.641455339	0.247932467	-2.587217991	0.009675436	0.044285963
9130214F15Rik	2.002874119	-0.938420772	0.348656972	-2.691530208	0.007112506	0.034881494
9330151L19Rik	19.97837996	-0.786561998	0.289124958	-2.720491522	0.006518494	0.032531774
9330175E14Rik	15.76318416	-0.792177924	0.311793898	-2.540710157	0.011062759	0.049425073
9430037O13Rik	2.971816536	1.060511679	0.371038216	2.858227622	0.004260147	0.022931141
9430038I01Rik	232.3742375	0.314644029	0.113214238	2.77919134	0.005449442	0.028122341
9530059O14Rik	16.48399086	-1.089090551	0.31087586	-3.503297274	0.000459536	0.003441503
9630010A21Rik	60.91061288	-0.781346666	0.227812706	-3.4297765	0.000604079	0.004331298
A130010J15Rik	120.3778734	0.430576676	0.1573411	2.736581073	0.00620813	0.031326631
A130050O07Rik	14.55508833	-0.911256555	0.336389738	-2.708930899	0.00675004	0.033431632
A230028O05Rik	20.61566331	-1.170645736	0.2974363	-3.935786369	8.29E-05	0.000767706
A330023F24Rik	65.10790376	-0.898922085	0.213324961	-4.213862648	2.51E-05	0.000265307
A430005L14Rik	165.7158951	0.359831779	0.127653141	2.818824324	0.004819989	0.025353514

A430018G15Rik	11.68919595	-0.890577568	0.339195066	-2.625561678	0.008650613	0.040619448
A430035B10Rik	28.88832541	-0.57757763	0.253975737	-2.274144913	0.022957275	0.088725168
A430057M04Rik	8.709660008	-0.888245184	0.367173526	-2.419142782	0.015557132	0.064743729
A430106G13Rik	12.85131935	-0.899027064	0.330750791	-2.718140331	0.006564999	0.032720338
A530030E21Rik	22.81423556	0.613414839	0.30639481	2.002040568	0.045280369	0.147722281
A530084C06Rik	4.7111488	-1.255134768	0.381377246	-3.291058341	0.000998112	0.006663109
A630035G10Rik	5.871643923	-0.796210567	0.376208048	-2.116410247	0.034309931	0.120951172
A630072M18Rik	66.48078115	-0.407117211	0.194964069	-2.088165344	0.036782922	0.127013648
A930007I19Rik	67.51361492	-0.652031957	0.193531176	-3.369131375	0.000754055	0.005232672
A930033H14Rik	34.03213169	1.084078117	0.246169403	4.403789025	1.06E-05	0.000123751
AA386476	33.01793736	-0.545786892	0.25230926	-2.163166312	0.03052839	0.110635005
Aacs	171.8875441	0.562986645	0.147137831	3.826253528	0.000130108	0.001144511
Aars	409.3895554	0.357116931	0.116130009	3.075147692	0.002103983	0.012579474
Abat	5.020121704	-1.278372567	0.381303827	-3.352635027	0.000800462	0.005502763
Abcb4	107.4475615	0.937964287	0.198994255	4.713524461	2.43E-06	3.22E-05
Abcc5	933.942963	-0.51482789	0.075542274	-6.81509654	9.42E-12	2.89E-10
Abcc9	3.915750506	0.970810165	0.380691511	2.550122967	0.010768493	0.048355109
Abcd2	538.9685408	-0.895517935	0.092649214	-9.66568306	4.22E-22	4.55E-20
Abcd3	553.3587293	-0.297952125	0.101126242	-2.946338349	0.003215604	0.018053339
Abce1	455.0926629	0.24612569	0.10861961	2.265941574	0.023454961	0.090253486
Abcg1	468.7371431	-2.466245379	0.102849795	-23.97909853	4.60E-127	1.72E-123
Abcg3	208.9928467	1.70648359	0.152586424	11.18371836	4.90E-29	1.02E-26
Abhd12	1821.089089	-0.507234495	0.078784054	-6.438288849	1.21E-10	3.23E-09
Abhd17b	852.924258	0.455063749	0.110866134	4.104623596	4.05E-05	0.000407335
Abhd3	42.42447044	-0.796019034	0.24305703	-3.275029873	0.001056508	0.006987667
Abhd6	51.08134163	0.591668449	0.246790449	2.397452788	0.016509508	0.067966029
Abi2	563.9678934	-0.234599607	0.097554552	-2.404804314	0.016181128	0.066876944
Abi3	701.1243703	-0.421160475	0.088046214	-4.783402433	1.72E-06	2.36E-05
Abl1	424.9403011	0.425723778	0.097254115	4.377437151	1.20E-05	0.000138729
Abl2	630.3141382	-0.235918975	0.08638788	-2.730926762	0.00631565	0.031719868
Abr	1036.082117	-0.752516226	0.092220011	-8.160010137	3.35E-16	1.84E-14
Abtb1	93.33344335	-0.458920418	0.170991398	-2.683880139	0.007277319	0.035538799
Acaca	203.9372737	0.348900502	0.125769959	2.774116376	0.005535187	0.028457196
Acad12	58.08628898	-0.755791008	0.227891175	-3.316455795	0.00091167	0.006162808
Acap2	855.5909722	0.373067791	0.080220493	4.650529794	3.31E-06	4.27E-05
Acat1	380.9221313	0.904207372	0.118499534	7.630471957	2.34E-14	1.01E-12
Acat2	136.9149559	0.380957147	0.147588758	2.581207089	0.00984555	0.044909475
Ace	4.011878962	-1.574080747	0.377128835	-4.17385413	2.99E-05	0.000310819
Acer2	10.42008189	0.874070876	0.35011324	2.496537626	0.012541236	0.054695244
Ackr3	12.13634415	0.923299397	0.347135919	2.659763357	0.007819557	0.037647768
Acly	745.8678348	0.314607169	0.084949615	3.703456094	0.000212682	0.001763381
Acot13	517.7392953	0.427146937	0.105103357	4.064065612	4.82E-05	0.000474568
Acot7	99.98558327	-0.441173612	0.182872074	-2.412471204	0.015844789	0.065722153
Acox3	799.7047274	-0.753438468	0.111154557	-6.778295817	1.22E-11	3.66E-10

Acp1	402.185296	0.265597627	0.13183426	2.014632816	0.043943136	0.144363977
Acp2	1533.905854	0.23305487	0.076220371	3.057645438	0.002230834	0.013195948
Acp5	167.6894298	-0.697886144	0.167346003	-4.170318567	3.04E-05	0.00031481
Acpp	541.7175856	-1.359849806	0.260691896	-5.216310228	1.83E-07	2.95E-06
Acrbp	70.80004798	-0.711298612	0.227369574	-3.128380815	0.001757723	0.010810716
Acsl1	398.9994582	-0.494567949	0.120952699	-4.088936873	4.33E-05	0.00043183
Acss1	209.930407	0.512543861	0.142088698	3.607210625	0.000309506	0.002443326
Acss2	60.16192414	-0.767333895	0.202223372	-3.794486691	0.000147949	0.001276019
Acta2	232.6268734	-1.626320439	0.184039502	-8.836800906	9.85E-19	7.47E-17
Actb	84196.88609	0.407005464	0.071980191	5.654409319	1.56E-08	3.06E-07
Actl6a	521.4188765	0.441053127	0.100028325	4.409282351	1.04E-05	0.000121123
Actn1	1356.267413	-0.28487559	0.111440225	-2.556308475	0.010578929	0.04763216
Actr1b	404.351211	-0.367434366	0.092376501	-3.977573975	6.96E-05	0.000656704
Actr2	2987.617582	0.248253742	0.062385434	3.979354266	6.91E-05	0.000652627
Actr3b	13.12090834	-0.713759749	0.326658486	-2.185033547	0.028886387	0.105963825
Actr8	193.2663018	-0.25277846	0.127899149	-1.976388916	0.048110746	0.154009805
Acvr2b	32.10378092	-0.628444946	0.274536467	-2.289112816	0.022072798	0.085946406
Acy1	49.35462773	1.082681912	0.25391192	4.264005844	2.01E-05	0.000218673
Acy3	34.91835843	0.805594857	0.250804189	3.21204705	0.001317928	0.008430796
Adam10	1347.337969	0.182846266	0.092772884	1.970902044	0.048735081	0.155246734
Adam12	46.1466289	-1.299074123	0.215162185	-6.037650732	1.56E-09	3.58E-08
Adam15	1763.92869	0.405128223	0.095213849	4.254929579	2.09E-05	0.000226094
Adam19	83.94673883	-0.465731251	0.196149058	-2.374374135	0.017578728	0.071510972
Adam23	7.503083482	-1.25478871	0.369604547	-3.394949336	0.000686413	0.004815802
Adam33	18.22632556	0.798079035	0.305793938	2.609858917	0.009057957	0.04209748
Adam8	4666.243842	0.517098179	0.135834295	3.806830818	0.000140759	0.001222771
Adam9	309.4654724	0.704946364	0.141898937	4.967946774	6.77E-07	1.00E-05
Adamts1	13.11556795	-0.779357509	0.326496507	-2.387031688	0.016985027	0.06952927
Adamts10	97.91891378	-0.360100033	0.163393563	-2.203881392	0.027532687	0.101894704
Adamts12	1.269677866	-0.647802356	0.298636182	-2.169202507	0.030067313	
Adap1	325.7708645	-0.331107915	0.11319082	-2.925218804	0.003442141	0.019110696
Adap2	1063.640626	-0.552178921	0.116913221	-4.722981006	2.32E-06	3.09E-05
Adarb1	87.49231532	0.800528022	0.171459578	4.668902331	3.03E-06	3.94E-05
Adcy3	44.77060951	1.018602359	0.224637987	4.534417239	5.78E-06	7.11E-05
Adcy4	21.19289907	-1.359499579	0.319924893	-4.249433564	2.14E-05	0.00023138
Adcy7	1061.094421	-0.50053534	0.115102358	-4.348610637	1.37E-05	0.000155671
Adcy9	143.3857757	-0.358104483	0.161210006	-2.221353947	0.026326998	0.098624377
Adcyap1r1	21.9188245	1.686611943	0.310628934	5.429667871	5.65E-08	9.99E-07
Adgb	302.8854287	1.796435802	0.159682125	11.25007451	2.31E-29	4.96E-27
Adgrd1	12.23231356	-0.731364071	0.366016752	-1.998171029	0.045698121	0.14885863
Adgre1	8494.904125	0.264104532	0.072243457	3.655757125	0.000256424	0.002077838
Adgre4	187.4556901	1.020679787	0.215724817	4.731397158	2.23E-06	2.97E-05
Adgre5	924.8641105	-0.311025387	0.076138084	-4.085017267	4.41E-05	0.000437731
Adgrg6	169.9220781	-0.421518619	0.132530595	-3.180538182	0.001470018	0.009284745

Adgrl1	100.6669759	-0.339586651	0.150793649	-2.251995712	0.024322543	0.092830214
Adh5	547.998272	0.289713391	0.092734312	3.12412292	0.001783359	0.010937014
Adh7	5.906362114	1.307854514	0.380793789	3.434547913	0.000593543	0.004265949
Adipor2	1078.202076	0.159175566	0.075905893	2.097011959	0.035992517	0.125177219
Adk	258.8081172	0.61034602	0.13282898	4.594976322	4.33E-06	5.49E-05
Ado	756.1590446	-0.313681466	0.085821414	-3.655048917	0.000257133	0.002082458
Adora2a	13.52845787	-0.710860666	0.333584724	-2.130974872	0.033091212	0.11753842
Adpgk	112.5159855	0.460125606	0.162411654	2.833082456	0.004610149	0.024472766
Adra2a	4.19996881	-1.057917116	0.378227789	-2.797036992	0.005157363	0.026845835
Adrb2	22.56121361	1.253405874	0.293197253	4.274957772	1.91E-05	0.000209412
Adssl1	461.6227216	-0.39708614	0.151077649	-2.628357942	0.008579818	0.040426164
Aebp1	158.4352346	-0.839596076	0.171779295	-4.887644201	1.02E-06	1.47E-05
Aebp2	529.7095521	-0.269154648	0.090771545	-2.965187465	0.003024985	0.017156446
Afap1l1	116.1772842	2.354659266	0.181225136	12.9930059	1.34E-38	5.75E-36
Afdn	789.1075149	-0.403526022	0.093016142	-4.338236484	1.44E-05	0.000161943
Agap1	405.8869272	-0.559930216	0.109780959	-5.100431095	3.39E-07	5.24E-06
Agfg1	889.8922828	0.737513078	0.101175512	7.289442538	3.11E-13	1.15E-11
Agfg2	333.1619879	0.350873417	0.11125668	3.153728986	0.001611987	0.010058665
Agl	483.5052085	-0.197650334	0.091391438	-2.162678898	0.030565885	0.110654054
Ago2	1073.478181	-0.148288793	0.070642637	-2.099140106	0.035804552	0.124728284
Agpat2	190.7681887	-0.337296623	0.123357742	-2.734296343	0.006251375	0.031470778
Agpat3	292.37861	0.308356414	0.11339738	2.719255198	0.006542911	0.032631924
Agpat4	225.4815745	-0.393399935	0.115306194	-3.411784915	0.00064539	0.004570727
Agpat5	373.12129	0.355391575	0.12187587	2.91601263	0.003545361	0.019581511
Agps	570.1807571	0.418945918	0.092799518	4.514526854	6.35E-06	7.72E-05
Agtpbp1	319.7351789	-0.312549348	0.104126622	-3.00162765	0.002685404	0.015499947
Ahcyl2	547.2969396	-0.285136819	0.117786021	-2.420803552	0.015486244	0.064520283
Ahi1	88.37456549	-0.433410405	0.172166918	-2.517384933	0.011822958	0.052123129
Ahnak	4573.562319	-0.366619153	0.095460806	-3.840520203	0.000122774	0.001088286
Ahnak2	536.3207312	-1.733348024	0.14432625	-12.00992906	3.15E-33	9.09E-31
Ahrr	241.3301761	-0.396855909	0.162242193	-2.446070913	0.014442259	0.060983394
AI480526	80.92647892	-0.65154488	0.189151009	-3.444575231	0.000571957	0.004138147
AI506816	13.02977548	0.755158209	0.332537456	2.270896696	0.023153232	0.089344448
AI662270	1961.565797	0.211645712	0.097037855	2.181063375	0.029178729	0.106905564
AI839979	69.58789614	0.517884415	0.196391149	2.637004862	0.008364163	0.039609024
Aif1	942.897929	0.756544684	0.227655332	3.323202128	0.000889904	0.006029251
Aifm1	245.9866756	0.421373132	0.123197064	3.420317964	0.00062548	0.004454948
Airn	73.09836425	-1.415875323	0.244411551	-5.792996768	6.91E-09	1.43E-07
Ak1	85.25206375	-0.769616682	0.202817985	-3.794617541	0.000147871	0.001276019
Ak2	488.9486846	-0.223183282	0.088259054	-2.52872959	0.011447619	0.050736685
Ak4	20.08507656	0.728356031	0.314078165	2.319027907	0.020393523	0.08060195
Ak6	221.9425382	0.368454234	0.135773468	2.713742516	0.006652785	0.033037177
Ak8	65.39986051	-1.079876479	0.191419197	-5.641422036	1.69E-08	3.26E-07
Akap10	144.8989349	-0.307378855	0.15222952	-2.019180349	0.043468478	0.143275082

Akap12	57.14964329	-1.359298561	0.255600977	-5.31804916	1.05E-07	1.78E-06
Akap13	2904.566844	-0.29092325	0.093399085	-3.114840453	0.001840444	0.011213783
Akap8l	222.498195	-0.481688716	0.147435564	-3.267113456	0.001086501	0.007157649
Akirin2	559.8953283	0.276720143	0.09262076	2.987668684	0.002811141	0.01606504
Akr1a1	13145.61799	-0.323165477	0.110278657	-2.930444431	0.003384775	0.018840995
Akr1b10	356.8050687	-0.235291647	0.09549383	-2.463946059	0.013741679	0.058735795
Akr1c12	42.39687479	0.808591616	0.245067612	3.299463401	0.000968699	0.006489848
Akr1c13	250.8008187	0.349406818	0.114822403	3.043019562	0.002342171	0.013756932
Akr1e1	241.6206531	0.361880392	0.134796352	2.684645297	0.007260682	0.035480641
Akt3	366.5218485	-0.562894337	0.133104109	-4.228977924	2.35E-05	0.000250211
Alas1	970.6503828	-0.739326761	0.124336526	-5.946175157	2.74E-09	6.01E-08
Alcam	3047.320416	-0.533639182	0.140257794	-3.804702511	0.000141975	0.001230858
Aldh1a1	5.845102605	-0.985001916	0.381082855	-2.584744773	0.009745111	0.044532544
Aldh1a2	66.87983832	-1.110399135	0.197671148	-5.617406221	1.94E-08	3.70E-07
Aldh1b1	76.66166378	-0.558805228	0.17968011	-3.110000479	0.00187087	0.011350467
Aldh1l2	19.45330636	-0.921418162	0.303168769	-3.039291164	0.002371355	0.013901145
Aldh7a1	192.1790738	0.386245691	0.129101435	2.991800136	0.002773378	0.015885526
Aldh9a1	832.1337896	0.32087153	0.098400332	3.260878533	0.001110676	0.007293473
Aldoc	181.9466723	1.196901477	0.176176614	6.793759099	1.09E-11	3.32E-10
Alg1	279.6078417	0.318680159	0.113993957	2.795588181	0.005180537	0.026957113
Alg14	239.9308681	0.554679859	0.122298335	4.535465343	5.75E-06	7.08E-05
Alg8	216.7351888	0.560807709	0.151802782	3.694317742	0.000220478	0.001821978
Alkbh1	127.3175786	-0.495052508	0.148816383	-3.326599518	0.000879126	0.005967005
Alkbh5	819.2325084	0.237788177	0.085964308	2.766126822	0.005672647	0.029084186
Alox15	759.6604005	2.878546725	0.140095602	20.547017	8.18E-94	1.75E-90
Alox5	455.031241	1.619697585	0.138773502	11.6715191	1.78E-31	4.61E-29
Alox5ap	5883.913516	0.531369641	0.094573405	5.618594767	1.93E-08	3.68E-07
Alyref	201.0357324	0.636441366	0.143713303	4.42854873	9.49E-06	0.000111751
Amacr	50.86177176	0.495178821	0.216345909	2.288829134	0.022089282	0.085953034
Amd-ps4	2.762596666	-0.767030933	0.368373659	-2.082208959	0.037323387	0.128362437
Ammecr1	356.5318871	-0.326796895	0.099637326	-3.279864183	0.001038571	0.006887243
Amn1	250.6105874	-0.723426615	0.124556793	-5.808006127	6.32E-09	1.31E-07
Amotl1	101.479765	-0.666372017	0.207177793	-3.216425895	0.001297981	0.008338525
Amotl2	29.54175789	-1.147011913	0.263767868	-4.348565738	1.37E-05	0.000155671
Ampd2	151.8959108	0.290533751	0.140527998	2.067443887	0.038692346	0.131757668
Ampd3	497.3959149	0.25060463	0.090280791	2.775835569	0.005506005	0.028354338
Amz1	126.1761171	-1.656646247	0.169756677	-9.758946036	1.69E-22	1.93E-20
Amz2	212.5797033	-0.33288545	0.127159714	-2.617853095	0.008848489	0.041341617
Anapc5	794.8935121	0.227501065	0.094006134	2.420066164	0.015517683	0.064633326
Ang	18.23353935	1.809891583	0.32881152	5.504343597	3.71E-08	6.78E-07
Angptl2	164.1216579	-0.579749504	0.22751682	-2.548161069	0.010829246	0.048579919
Angptl4	64.35522293	-1.247904816	0.278190415	-4.485793714	7.26E-06	8.74E-05
Ankfy1	781.2585238	0.352601537	0.08886675	3.967755519	7.26E-05	0.000680318
Ankib1	300.4932122	0.251624148	0.105335159	2.388795451	0.016903711	0.069290901

Ankle1	5.632613977	1.249173349	0.380576523	3.28231847	0.001029573	0.006836628
Ankrd1	91.39031539	-1.161040109	0.357717245	-3.245692305	0.001171654	0.007644788
Ankrd16	154.7978185	-0.353565712	0.142343399	-2.483892574	0.012995501	0.056251363
Ankrd28	45.33347481	0.769907388	0.227572634	3.383128165	0.000716652	0.00500223
Ankrd33b	236.2194917	0.281584952	0.117208926	2.402419022	0.01628704	0.067222115
Ankrd34a	10.77276246	-0.895734153	0.355488691	-2.519726157	0.011744617	0.051869176
Ankrd37	78.03062987	0.905980095	0.223501556	4.05357399	5.04E-05	0.000492497
Ankrd44	559.7339595	-0.292576211	0.112562806	-2.599226345	0.009343414	0.043049369
Ankrd50	229.8600938	-0.235198763	0.113906199	-2.064846037	0.03893757	0.132442456
Anks3	75.63804056	-0.525806263	0.184928195	-2.843299607	0.004464908	0.023870634
Anln	178.7294713	1.789762065	0.210397483	8.506575473	1.79E-17	1.17E-15
Ano3	2.373129568	-0.879057308	0.367265735	-2.393518438	0.016687643	0.06855501
Anp32a	879.4538992	0.326410597	0.080130838	4.073470405	4.63E-05	0.000457597
Anp32b	1446.5185	0.218496836	0.106895026	2.044031842	0.040950402	0.137259266
Anp32e	835.7023307	0.35916605	0.104126089	3.449337769	0.000561963	0.004078038
Anpep	548.6230937	-1.945697502	0.148194875	-13.12931708	2.24E-39	1.02E-36
Antxr1	54.21150717	-1.530390039	0.22620577	-6.765477456	1.33E-11	3.97E-10
Antxr2	967.348179	-0.617689904	0.170817134	-3.616088678	0.000299088	0.002374822
Anxa1	3994.532496	-1.047463226	0.152557658	-6.866015372	6.60E-12	2.08E-10
Anxa2	3122.735991	-0.356557742	0.105161648	-3.390568223	0.000697479	0.004884305
Anxa3	3585.699273	-0.20066251	0.066676129	-3.009510512	0.00261669	0.015185101
Anxa4	1919.038445	-0.889922305	0.107418276	-8.284645233	1.18E-16	6.89E-15
Anxa5	4328.758268	-0.464256481	0.094389807	-4.918502281	8.72E-07	1.27E-05
Anxa7	834.4776784	-0.192933767	0.092180954	-2.092989489	0.036350093	0.126011976
Aoah	254.5928728	1.067764138	0.175220011	6.093848128	1.10E-09	2.60E-08
Aox1	12.7990423	-0.820908026	0.358166434	-2.291973637	0.021907164	0.085348083
Aox3	5.465705733	-0.804432449	0.378858225	-2.123307337	0.033728106	0.119483408
Ap1b1	665.0879268	0.636263903	0.125852083	5.055648567	4.29E-07	6.56E-06
Ap1s2	1493.458665	0.493456753	0.082757909	5.962653705	2.48E-09	5.47E-08
Ap2a2	712.4142474	0.52904186	0.097249032	5.440073262	5.33E-08	9.47E-07
Ap2m1	487.9151164	0.243839165	0.098835565	2.467119656	0.013620483	0.058334074
Ap2s1	1292.38877	0.752599415	0.110839423	6.7899976	1.12E-11	3.39E-10
Ap3m1	509.7455555	0.497884475	0.106546494	4.672931566	2.97E-06	3.87E-05
Ap4m1	120.6870214	-0.445179885	0.144013546	-3.091236193	0.00199325	0.012003493
Apaf1	492.5027907	0.236947208	0.106528965	2.224251471	0.02613153	0.098062127
Apbb1ip	3061.831191	0.200678846	0.082034494	2.44627395	0.014434128	0.060966214
Apbb2	360.0988609	-1.400090189	0.189402811	-7.392129941	1.44E-13	5.57E-12
Apc-ps1	5.93865878	-0.980617452	0.377387286	-2.59843797	0.009364896	0.043110122
Aph1b	210.9374059	1.341085476	0.138578743	9.677425579	3.76E-22	4.12E-20
Aph1c	394.7820682	-0.710667651	0.113917157	-6.238460198	4.42E-10	1.10E-08
Apip	178.504538	0.303154618	0.133541801	2.270110303	0.023200891	0.089462369
Apln	23.40051606	1.614012491	0.315466971	5.11626458	3.12E-07	4.87E-06
Apmap	201.2369394	0.359987562	0.133694578	2.692611535	0.007089482	0.034814088
Apobec1	1296.122961	-0.569811609	0.09066429	-6.284851629	3.28E-10	8.32E-09

Apoc1	71.66477117	-2.176692055	0.224212678	-9.708157783	2.78E-22	3.09E-20
Apoc4	28.68818933	-0.8090852	0.299961711	-2.69729492	0.006990533	0.034418283
Apoe	97454.23047	-0.419493827	0.112226237	-3.737930069	0.000185542	0.001554689
App	7268.222989	0.367816432	0.093622475	3.928719373	8.54E-05	0.000786734
Appbp2	971.5260181	0.335902349	0.072075565	4.6604192	3.16E-06	4.08E-05
Aprt	772.4041107	0.25462473	0.106138721	2.398980571	0.016440787	0.06774489
Aqp1	11.14662128	2.179711409	0.369211249	5.903697181	3.55E-09	7.66E-08
Aqp9	9.592703249	0.747467505	0.362080885	2.064366102	0.038983018	0.132536962
Ar	12.59642117	-0.817026287	0.34002025	-2.402875379	0.01626673	0.067175238
Arap3	38.77500534	1.209373286	0.248084082	4.874852416	1.09E-06	1.56E-05
Arf1	1258.431796	0.159477667	0.06931571	2.300743473	0.021406135	0.083743825
Arf3	910.9753287	-0.206266412	0.09020557	-2.286626121	0.022217655	0.08637833
Arf6	2036.406206	0.163221227	0.081166122	2.010952647	0.044330459	0.145223138
Arfgap1	233.4187984	-0.496747978	0.119751431	-4.148159016	3.35E-05	0.000342643
Arfgef1	884.985425	-0.163154492	0.075932558	-2.14867634	0.031660065	0.113747331
Arg1	7229.529421	1.334612764	0.283332601	4.710410159	2.47E-06	3.26E-05
Arg2	122.6403791	-0.903245495	0.330139366	-2.73595211	0.006220008	0.031371712
Arhgap10	388.9073219	-0.36949538	0.132778922	-2.782786415	0.005389427	0.027860535
Arhgap11a	245.6818212	0.44051983	0.119187528	3.696022872	0.000219003	0.001810789
Arhgap12	846.1890367	0.369107759	0.087103475	4.23757788	2.26E-05	0.00024185
Arhgap17	742.8982869	-0.405951961	0.088942289	-4.564217582	5.01E-06	6.25E-05
Arhgap18	660.880996	0.383632331	0.094161831	4.07418089	4.62E-05	0.000456844
Arhgap23	327.5983004	0.379928608	0.099892185	3.803386702	0.000142731	0.001236702
Arhgap25	1288.429642	-1.267479682	0.127696066	-9.925753529	3.22E-23	3.99E-21
Arhgap27	130.2500844	-0.493883785	0.144321811	-3.422100791	0.000621393	0.004432149
Arhgap30	1918.059539	0.238857759	0.069278722	3.447779502	0.000565215	0.004099655
Arhgap31	534.8712356	-0.472842784	0.093101752	-5.078774292	3.80E-07	5.84E-06
Arhgap32	32.77639468	-0.856942757	0.271115269	-3.160805959	0.001573333	0.009866696
Arhgap39	102.9865178	-0.721249449	0.163234555	-4.418485096	9.94E-06	0.000116442
Arhgap4	250.5978358	0.561025288	0.149604501	3.750056211	0.000176795	0.001491378
Arhgap42	10.68486502	-1.007930135	0.358213689	-2.813767775	0.004896459	0.025692712
Arhgap44	3.347854511	-0.749245638	0.376024196	-1.992546347	0.046311145	0.150365936
Arhgap6	573.6750333	-1.331289594	0.181497951	-7.335011698	2.22E-13	8.38E-12
Arhgap9	788.6249766	-0.795844505	0.080162383	-9.927904791	3.15E-23	3.94E-21
Arhgdib	2036.553843	0.294235676	0.07182891	4.09634052	4.20E-05	0.000419985
Arhgef1	983.7864216	-0.196660843	0.094680286	-2.077104447	0.03779192	0.129479611
Arhgef18	75.34228877	-0.574979354	0.200572499	-2.866690877	0.004147879	0.022399146
Arhgef2	757.9256027	-0.186503685	0.079554351	-2.34435555	0.019059992	0.076214575
Arhgef25	14.60835281	-0.874695766	0.333137835	-2.625627214	0.008648948	0.040619448
Arhgef28	3.199972391	-0.949204976	0.377699648	-2.513121157	0.011966823	0.052679998
Arhgef39	46.55345258	2.147135452	0.280116061	7.665163657	1.79E-14	7.81E-13
Arhgef7	593.8374883	0.58924527	0.106358983	5.540155165	3.02E-08	5.63E-07
Arid1a	737.3630214	-0.228083043	0.103185995	-2.210406962	0.027076931	0.100706321
Arid1b	859.0136622	-0.238330322	0.090140508	-2.643986903	0.008193583	0.03907248

Arid4a	1701.047199	-0.343342648	0.101974895	-3.366933093	0.000760091	0.005266246
Arid5b	612.5166844	-0.820647027	0.145061835	-5.657222159	1.54E-08	3.01E-07
Arih1	1279.629546	-0.35160333	0.07374255	-4.76798445	1.86E-06	2.53E-05
Arl10	98.09300063	-0.505488351	0.161955684	-3.121152265	0.001801449	0.011016438
Arl16	65.5269915	0.442154117	0.222615549	1.98617805	0.047013557	0.151890733
Arl3	204.9487735	-0.32736728	0.137589073	-2.379311617	0.017345006	0.070734706
Arl4c	1101.281149	0.232718406	0.106890126	2.177174037	0.029467585	0.107775428
Arl5a	278.4403668	-0.521372449	0.122428476	-4.258588097	2.06E-05	0.00022323
Arl5c	343.0861701	-0.777628597	0.193200153	-4.024989554	5.70E-05	0.00055059
Arl6ip1	4670.493875	0.364654093	0.094376459	3.8638247	0.000111625	0.000997714
Arl6ip5	164.3820703	-0.355054792	0.130018095	-2.730810596	0.006317877	0.031720435
Arl8b	1303.083074	0.194661055	0.09260134	2.102140795	0.035540944	0.124066748
Armc1	598.1458389	0.363803043	0.09031903	4.027977745	5.63E-05	0.000544693
Armc2	157.6133858	-0.991038872	0.173718038	-5.704870285	1.16E-08	2.33E-07
Armc3	17.14425131	0.806381465	0.313729255	2.570310078	0.010160752	0.046025478
Armc7	97.77582724	-0.393570325	0.178472222	-2.205218941	0.027438735	0.101622184
Armcx2	67.26481959	-0.608500007	0.181686651	-3.349172901	0.000810532	0.005564343
Armcx4	11.27212514	-0.681381608	0.33974242	-2.005582958	0.044900762	0.146739053
Arpc2	12429.00255	0.194381989	0.071376553	2.723331128	0.006462725	0.03231793
Arpc3	2047.543943	0.744430964	0.085309462	8.726241447	2.63E-18	1.92E-16
Arpc4	1770.804214	0.387803965	0.099168443	3.910558168	9.21E-05	0.000841078
Arrb1	774.9761638	-0.377301307	0.077869447	-4.845306114	1.26E-06	1.78E-05
Arrb2	1528.925206	0.403378636	0.084332226	4.783208679	1.73E-06	2.36E-05
Arrdc1	223.4363422	-0.301884854	0.115293121	-2.618411676	0.008834016	0.041299712
Arrdc3	168.6549152	-0.668980121	0.16694277	-4.007242247	6.14E-05	0.000587577
Arsb	77.56237942	0.805283504	0.199900941	4.028412779	5.62E-05	0.000544037
Arsg	324.1605116	0.454996208	0.159431292	2.853870173	0.004319018	0.02321472
Arsj	2.47879753	-1.094662635	0.362334071	-3.021141874	0.002518233	0.014659088
As3mt	95.30159587	0.357112304	0.175368335	2.036355676	0.041714653	0.139082825
Asah2	121.5358131	0.301605367	0.147615801	2.04317807	0.041034814	0.137435414
Asap1	1800.549407	-0.77826556	0.086443149	-9.003206955	2.19E-19	1.79E-17
Asap2	82.69796737	-0.781235283	0.181378666	-4.307206031	1.65E-05	0.000184195
Asb10	35.96056471	0.88082192	0.281776936	3.12595464	0.001772289	0.010882463
Asb13	141.6595339	-0.307686854	0.144006113	-2.136623565	0.032628621	0.116313785
Asb4	151.4555192	0.897727951	0.16597497	5.408815269	6.34E-08	1.11E-06
Asf1a	159.4242303	0.355374805	0.135438441	2.623884337	0.00869333	0.040743537
Asf1b	225.9659016	1.762064388	0.18345972	9.604639041	7.64E-22	7.86E-20
Asic3	8.071653942	-0.773519892	0.36006392	-2.148284924	0.031691128	0.113777214
Asl	84.51559005	-0.362477353	0.167590812	-2.162871271	0.030551082	0.110654054
Asns	37.14273912	-0.657185863	0.243325527	-2.700850463	0.006916243	0.034145794
Asph	764.7760607	-1.194493663	0.124829475	-9.569003315	1.08E-21	1.09E-19
Asphd2	3.042751617	-1.314645136	0.370340715	-3.549826099	0.000385486	0.002945511
Aspm	132.7749768	1.805253462	0.313966855	5.749821784	8.93E-09	1.81E-07
Asrgl1	38.05441062	0.548601396	0.232134641	2.363289651	0.018113506	0.073194668

Ass1	35.23218002	-1.130396316	0.255377502	-4.426373921	9.58E-06	0.000112706
Atad2	525.7485183	1.026230793	0.138296587	7.420507026	1.17E-13	4.55E-12
Atad5	96.62714874	1.058457375	0.187412221	5.64775002	1.63E-08	3.16E-07
Atf3	1496.717242	-1.71776179	0.139504821	-12.31327905	7.68E-35	2.45E-32
Atf6	570.9496996	-0.360603766	0.094688385	-3.808321002	0.000139914	0.001217208
Atf7	361.7117312	-0.224030874	0.102222361	-2.191603404	0.028408156	0.104439293
Atg10	104.4674929	0.534551457	0.160836442	3.32357175	0.000888726	0.006023986
Atg101	381.1255243	0.622276855	0.11531259	5.396434657	6.80E-08	1.19E-06
Atg13	154.9007007	-0.401755779	0.132943399	-3.022006228	0.002511054	0.014622966
Atg14	148.3741126	-0.417906186	0.144384477	-2.894398297	0.00379886	0.020791205
Atg7	668.0614289	-0.423507342	0.080381169	-5.268738275	1.37E-07	2.29E-06
Atmin	334.6702656	0.263424219	0.111012906	2.372915266	0.017648312	0.071658068
Atoh8	11.2288229	-1.63161646	0.366256533	-4.454846019	8.40E-06	9.97E-05
Atox1	1764.112913	-0.405688657	0.072995284	-5.557737889	2.73E-08	5.12E-07
Atp11c	617.7590241	-0.286595776	0.092417431	-3.10110087	0.001928026	0.011649324
Atp13a1	189.5354951	-0.31004637	0.142751092	-2.171936938	0.02986042	0.108818679
Atp13a2	1255.416996	-0.224978326	0.085733291	-2.624165287	0.008686162	0.040735386
Atp13a3	601.1869178	0.214622654	0.091712664	2.340163762	0.019275286	0.076911517
Atp1a3	481.4342616	-1.039679907	0.107786437	-9.645739656	5.12E-22	5.42E-20
Atp1b1	65.17545923	-1.261983562	0.197297625	-6.396344427	1.59E-10	4.21E-09
Atp2a2	901.267447	0.275467781	0.072814676	3.78313545	0.000154865	0.001326518
Atp2a3	84.3214152	0.65798968	0.219975225	2.991199036	0.002778843	0.01590469
Atp2b1	6466.466502	-0.368694924	0.079637888	-4.629642185	3.66E-06	4.68E-05
Atp5e	1074.644622	0.186701915	0.083872674	2.22601602	0.026013108	0.097814763
Atp5f1	1516.447112	0.202005003	0.077878913	2.593834403	0.009491219	0.043571344
Atp5g3	3179.309088	0.407279908	0.09816056	4.149119643	3.34E-05	0.000341656
Atp5md	366.9388622	0.319123011	0.157845039	2.021748749	0.043202315	0.142679828
Atp5mpl	1608.222609	0.267668599	0.103368638	2.589456567	0.009612755	0.044061885
Atp5o	782.5737436	0.237185449	0.097189147	2.440452004	0.014668895	0.061766585
Atp6ap1	3063.502054	0.158007529	0.073873519	2.138892682	0.032444361	0.115871614
Atp6v0a1	1369.036244	-0.600418992	0.113417174	-5.293898353	1.20E-07	2.01E-06
Atp6v0a2	341.0266767	-0.238033232	0.110825306	-2.147823828	0.031727753	0.113881462
Atp6v0d2	2664.303591	-2.146068697	0.223799872	-9.589231103	8.87E-22	9.06E-20
Atp6v1b2	4030.500734	-0.549083692	0.12163779	-4.514088031	6.36E-06	7.73E-05
Atp6v1d	1887.686766	0.292754181	0.082619425	3.543406174	0.000394994	0.003015093
Atp6v1e1	3374.593722	-0.189353719	0.081238419	-2.330839549	0.019761822	0.078602083
Atp6v1g1	373.9853587	0.28176776	0.111029931	2.537763996	0.011156319	0.049768989
Atp6v1g2	34.83678805	-0.537218477	0.238589393	-2.251644422	0.024344751	0.092867739
Atp6v1h	1300.354967	0.271392596	0.122110543	2.222515677	0.026248476	0.098354776
Atp7a	1072.171703	-0.227861845	0.089546638	-2.544616414	0.010939784	0.048992342
Atp8b1	5.776374901	0.830997106	0.376778303	2.205533333	0.027416691	0.101622184
Atpaf2	70.32470932	0.379857621	0.183212575	2.073316314	0.038142851	0.130211504
Atrnl1	121.0065152	0.746505848	0.187563473	3.980017194	6.89E-05	0.00065163
Atxn1	522.8301157	0.280560452	0.112350757	2.497183466	0.012518417	0.054627474

Atxn7l2	18.74247961	-0.662332392	0.289351365	-2.289024596	0.022077923	0.085946406
AU020206	636.7095326	-1.559550023	0.093154724	-16.74150225	6.53E-63	6.53E-60
Auh	455.8978577	-0.431737839	0.111722742	-3.864368442	0.000111377	0.000996087
Aunip	23.18223465	1.153034814	0.337856592	3.412793592	0.000643006	0.004558146
Aup1	807.9127981	-0.337278223	0.100751435	-3.34762698	0.000815066	0.005587803
Aurka	85.99547848	1.314390493	0.228830644	5.743944391	9.25E-09	1.86E-07
Aurkb	86.17153176	1.861187916	0.235324907	7.909013712	2.59E-15	1.28E-13
AV356131	17.25255042	-0.799122017	0.322940653	-2.474516634	0.013341661	0.057402037
Avpi1	48.60367239	0.893605688	0.243108744	3.675744745	0.000237157	0.001938458
AW112010	258.7415444	-0.767992522	0.36272931	-2.117260725	0.034237726	0.120781748
Axin2	21.57522971	-1.632120265	0.339374199	-4.809205504	1.52E-06	2.10E-05
Axl	480.151082	-1.519857072	0.117759466	-12.90645352	4.14E-38	1.73E-35
B230118H07Rik	102.0421241	0.332006102	0.16343923	2.031373381	0.042217133	0.140135481
B230217C12Rik	25.32383217	-0.623569755	0.297896675	-2.093241746	0.03632758	0.125992141
B230303O12Rik	23.27069543	-0.651198575	0.288290357	-2.258828844	0.023894034	0.091590641
B2m	28783.19044	-0.447440703	0.102728079	-4.355583282	1.33E-05	0.000151454
B3galnt1	236.6179365	0.506901471	0.141339562	3.586408943	0.000335263	0.002610946
B3gat3	144.9290151	-0.295394123	0.141842721	-2.082546928	0.037292541	0.128301046
B3glct	384.717684	0.417517313	0.107456015	3.885471783	0.000102131	0.000922193
B4galnt1	124.8775799	-0.697036464	0.160405382	-4.345468059	1.39E-05	0.000157527
B4galt1	982.2254323	0.615218586	0.093026887	6.61334168	3.76E-11	1.07E-09
B4galt6	1532.012243	-0.239795446	0.110505408	-2.169988333	0.03000773	0.109275904
B4gat1	163.9163178	0.381102021	0.128893137	2.956728562	0.003109217	0.017581017
B930095G15Rik	6.310351684	-0.844628851	0.375444381	-2.249677697	0.024469412	0.093248469
Babam2	390.5095104	-0.244391402	0.105293746	-2.321043848	0.020284477	0.080276675
Bace1	51.72802985	-0.473441492	0.223972423	-2.113838327	0.034529078	0.121466919
Bach2os	46.39705399	1.314113706	0.260243776	5.049549032	4.43E-07	6.76E-06
Bad	312.2908479	0.384648275	0.103558236	3.71431855	0.000203752	0.001694959
Bag3	595.5811834	-0.570309413	0.113938188	-5.005428144	5.57E-07	8.36E-06
Baiap2l1	19.79548466	-0.883670665	0.301510093	-2.930816198	0.003380727	0.018835441
Bak1	233.0372328	0.369487001	0.137767718	2.681956302	0.007319302	0.035651011
Bambi	83.5837337	-0.463743415	0.226489821	-2.04752431	0.040606632	0.136571881
Banf1	620.5451003	0.380664803	0.103105717	3.691985425	0.00022251	0.001833724
Bank1	397.1234606	1.54708962	0.191732653	8.068993956	7.09E-16	3.76E-14
Bard1	36.17430098	1.853848689	0.294910289	6.286144489	3.25E-10	8.26E-09
Barx1	28.83325064	-0.967135205	0.284400462	-3.400610526	0.000672355	0.004730445
Basp1	2535.71883	0.396058422	0.108714152	3.643117446	0.000269356	0.002174407
Batf	77.89204831	0.453062634	0.190881589	2.373527143	0.017619098	0.071589594
Batf3	27.29390325	-1.239626288	0.28397628	-4.365245881	1.27E-05	0.000145799
Baz1a	1111.719679	0.275338593	0.095465123	2.884179955	0.003924344	0.02136113
Baz1b	1578.666181	0.328434676	0.088921744	3.693524901	0.000221167	0.001826229
Baz2a	769.2815177	-0.27913173	0.12134463	-2.300322071	0.02142998	0.083793568
Baz2b	1406.456241	-0.228985017	0.099530771	-2.300645478	0.021411678	0.083743825
Bbc3	319.3404575	-0.546467686	0.101839584	-5.365965436	8.05E-08	1.39E-06

Bbip1	376.1298614	0.345731829	0.102961821	3.357864348	0.000785471	0.005414594
BC005537	4132.814981	0.25961873	0.105612725	2.458214473	0.013962976	0.059427788
BC028528	120.3072402	-0.365496661	0.160673705	-2.274775839	0.02291938	0.08860153
BC030867	12.6390433	1.163461202	0.345944499	3.363144106	0.000770601	0.005326768
BC049352	21.00971479	0.565266397	0.280528759	2.015003376	0.043904295	0.144267957
BC051226	69.59401846	-0.947960705	0.202024311	-4.692310056	2.70E-06	3.54E-05
BC055308	63.26008266	-0.48394667	0.199090393	-2.430788666	0.015065999	0.063190456
BC055324	43.19210515	0.713807633	0.257135832	2.775994413	0.005503315	0.028351615
BC065397	19.94697526	-0.604833942	0.304196428	-1.988300607	0.046778454	0.151424559
Bcap29	448.6765425	0.363146294	0.105451521	3.443727425	0.000573754	0.004145558
Bcas2	318.6221188	0.391444585	0.120050349	3.260670104	0.001111493	0.007293473
Bcat1	32.75779922	-0.853587217	0.260409409	-3.277866262	0.001045949	0.006926991
Bcl11a	132.2238695	0.562209543	0.246816373	2.277845415	0.022735789	0.087959781
Bcl2	298.4481269	0.329891817	0.130154113	2.534624612	0.011256788	0.050127781
Bcl2a1a	43.73040036	-0.608421509	0.238129149	-2.555006444	0.010618583	0.047753395
Bcl2l1	549.6503926	0.400484724	0.114387927	3.501110069	0.000463324	0.003462704
Bcl2l11	812.8898575	-0.974544296	0.128888738	-7.561128413	4.00E-14	1.67E-12
Bcl6	795.115454	-0.513059379	0.152995278	-3.353432767	0.000798158	0.005491957
Bcl7a	51.76877286	0.537120468	0.213275462	2.518435382	0.011787751	0.052034287
Bcl9	131.2762984	-0.402085877	0.15035707	-2.674206645	0.007490628	0.03637924
Bco2	23.07168671	0.724030349	0.293004786	2.471052975	0.013471586	0.057844948
Bcorl1	128.4557365	-0.346873207	0.140808263	-2.463443542	0.013760957	0.058801445
Bdnf	9.801708207	1.073313795	0.365830284	2.933911824	0.003347193	0.018694203
Bend4	125.6324673	0.345001114	0.147000842	2.346932901	0.018928663	0.075830873
Best1	49.2448579	-1.364871337	0.242743446	-5.622690793	1.88E-08	3.61E-07
Bet1l	524.8518011	-0.246838729	0.09430586	-2.617427264	0.008859537	0.041380351
Bex1	28.34325122	-2.678367945	0.310079576	-8.637679339	5.74E-18	4.02E-16
Bex3	89.27405673	0.659029902	0.191489518	3.441597796	0.000578289	0.004172302
Bgn	253.5672975	-0.94286913	0.155881674	-6.048620753	1.46E-09	3.36E-08
Bicc1	82.10486868	-0.826275887	0.201474362	-4.101146558	4.11E-05	0.000412676
Bid	216.3768292	0.727457635	0.121725079	5.976234632	2.28E-09	5.06E-08
Bik	1.932423411	0.700699083	0.351653295	1.992585004	0.046306909	
Bin1	1101.452218	1.079072519	0.125129968	8.623613807	6.49E-18	4.51E-16
Bin2	822.2230993	-0.2450577	0.113070289	-2.167304087	0.030211675	0.109820843
Bin3	265.5171249	-0.307877344	0.11745648	-2.621203561	0.008761993	0.041014106
Birc2	204.7930261	-0.39041046	0.151078156	-2.584162202	0.009761587	0.04459426
Birc5	409.3438246	1.86717241	0.259364057	7.19904073	6.06E-13	2.15E-11
Blm	65.93614912	0.750826748	0.19456585	3.858985265	0.000113859	0.001015258
Blnk	1274.939058	-0.221210277	0.097957741	-2.258221507	0.023931853	0.091606585
Bloc1s6	290.5867187	-0.250375811	0.118069518	-2.120579599	0.033957196	0.11995705
Blvra	1032.48587	0.285695112	0.105196209	2.715830874	0.006610968	0.032883923
Blvrb	2889.801241	-0.414998512	0.096219064	-4.313059116	1.61E-05	0.000179784
Bmf	53.56351282	-0.590017671	0.234525464	-2.515793634	0.01187647	0.052328299
Bmp1	38.07567368	-1.094862022	0.267624944	-4.091031294	4.29E-05	0.000428517

Bmp2k	1607.748619	0.418195727	0.087946416	4.755119629	1.98E-06	2.68E-05
Bmper	29.6167825	-0.77479833	0.289476612	-2.676548974	0.007438469	0.036149323
Bmt2	701.8387692	-0.506418444	0.094435528	-5.36258394	8.20E-08	1.41E-06
Bnip2	2596.903932	0.187856442	0.07459357	2.518399954	0.011788937	0.052034287
Bnip3	372.872011	0.82830369	0.140824283	5.881824324	4.06E-09	8.67E-08
Bnip3l	752.4739149	-0.656267776	0.083220316	-7.885908255	3.12E-15	1.52E-13
Boc	10.38142226	-0.784448456	0.34410812	-2.279656921	0.022628044	0.087637239
Bora	37.56705942	0.871897635	0.250604559	3.479177064	0.000502956	0.0036945
Bpnt1	170.0346207	0.395491912	0.136070014	2.906532459	0.00365459	0.020111635
Bptf	1931.618881	-0.253766656	0.088064929	-2.881585896	0.003956794	0.021490989
Braf	546.0483961	-0.249938353	0.113685703	-2.19850295	0.027913285	0.102998442
Brca1	39.36680951	1.488707882	0.247704754	6.010009329	1.86E-09	4.17E-08
Brca2	61.95633751	1.051829732	0.215349811	4.884284454	1.04E-06	1.50E-05
Bri3bp	369.4736388	-0.396217634	0.112224505	-3.53058037	0.000414649	0.003150703
Brip1	109.5838184	1.602701278	0.204558266	7.834937738	4.69E-15	2.23E-13
Brip1os	558.1579795	0.306002743	0.098394782	3.109948896	0.001871197	0.011350467
Brpf3	95.61580744	-0.664499314	0.165245336	-4.021289381	5.79E-05	0.000557517
Bsg	912.6614588	0.227805781	0.09257971	2.460644801	0.013868759	0.059161021
Bsn	9.068273916	-0.759019547	0.363460217	-2.088315337	0.036769399	0.126996172
Bst1	959.2586895	0.510404255	0.136733061	3.732851825	0.000189324	0.001583715
Bst2	594.2732532	1.435186606	0.129207047	11.10764961	1.15E-28	2.27E-26
Btbd10	166.7389469	0.401127592	0.142204043	2.820788944	0.004790571	0.025234152
Btbd17	14.0319157	-1.360847653	0.335118391	-4.060796689	4.89E-05	0.000480425
Btbd2	85.36930084	-0.608913195	0.168866732	-3.60588013	0.000311097	0.002453299
Btg2	525.5621414	-0.254726979	0.118777171	-2.144578601	0.031986555	0.114728066
Btk	315.4906809	0.399508903	0.105457764	3.788330855	0.000151663	0.001303553
Bub1	81.76512096	2.002676297	0.247181458	8.102049051	5.40E-16	2.90E-14
Bub1b	78.38491604	1.457938722	0.236686904	6.159777751	7.28E-10	1.74E-08
Bub3	919.9870049	0.344509159	0.081218776	4.24174283	2.22E-05	0.000237914
Bud31	385.4855003	0.271030223	0.099689438	2.71874562	0.006552998	0.032671377
Bysl	122.2144808	0.590775465	0.146088923	4.043944283	5.26E-05	0.000512181
C030034L19Rik	1.548939642	-0.717356892	0.328874916	-2.181245378	0.029165272	
C130050O18Rik	113.8854476	1.58766896	0.18703684	8.488536072	2.09E-17	1.34E-15
C1d	354.2253512	0.272757493	0.133792652	2.038658252	0.041484147	0.138653139
C1galt1	412.8735155	0.607282062	0.11273272	5.386919287	7.17E-08	1.25E-06
C1qa	11332.83243	0.509535097	0.080189812	6.354112662	2.10E-10	5.48E-09
C1qb	15366.38538	0.235936144	0.065487034	3.602791745	0.000314818	0.002478736
C1qbp	971.1747524	0.292775371	0.095112141	3.078212376	0.002082464	0.012485635
C1qc	12908.78991	0.343418021	0.082486976	4.163299944	3.14E-05	0.000323533
C1ra	120.8043509	0.857736662	0.170710003	5.024524907	5.05E-07	7.61E-06
C1rl	46.39648564	1.69779621	0.238884266	7.107191443	1.18E-12	4.09E-11
C1s1	15.85071258	-0.875404199	0.358449414	-2.442197322	0.014598165	0.061503275
C2	10.41582635	-0.750679091	0.36591078	-2.05153587	0.04021479	0.135557807
C2cd2l	346.1628075	0.360706465	0.122969039	2.933311253	0.003353675	0.018716475

C3	459.0543464	-0.69368979	0.300203977	-2.310728185	0.020847873	0.082051937
C330011M18Rik	14.74184925	-0.776279876	0.311869136	-2.489120553	0.012805953	0.055559105
C530043K16Rik	18.1677039	-1.35091623	0.340246675	-3.970402443	7.18E-05	0.000674247
C5ar1	1643.610536	0.604648534	0.087954244	6.874580521	6.22E-12	1.97E-10
C77080	32.75765959	-1.049842425	0.272658977	-3.850386425	0.000117932	0.001047219
C920006O11Rik	16.11720584	-0.719387593	0.309526784	-2.324152963	0.020117297	0.079741226
Cab39	1267.365456	0.172768875	0.084009361	2.056543141	0.039730192	0.134286261
Cab39l	214.9373962	0.344212638	0.127392997	2.70197456	0.006892904	0.034060522
Cabin1	362.0332559	-0.528239905	0.12397775	-4.260763769	2.04E-05	0.000221388
Cacna1a	264.3404474	-0.352232267	0.116868406	-3.013922055	0.002578941	0.014995025
Cacna1f	26.17814373	0.807981624	0.279085448	2.895104816	0.003790319	0.020755462
Cacna2d1	37.43699675	-0.528266675	0.269368879	-1.961127344	0.04986417	0.158038352
Cacnb4	13.04457996	-0.851896888	0.325664767	-2.615870595	0.008900028	0.041530697
Cacul1	934.3509933	0.299209267	0.087934078	3.402654271	0.000667347	0.004704026
Cacybp	782.8313597	0.338064295	0.095207319	3.550822548	0.000384029	0.002935877
Cadm1	1321.159819	-0.611716024	0.259965162	-2.353069234	0.018619167	0.074830702
Cald1	1922.549822	-0.445772474	0.135469447	-3.290575735	0.000999826	0.006671581
Calhm2	299.2404915	-0.316779697	0.10955194	-2.891593675	0.003832933	0.020954764
Calhm6	40.5770639	-1.059116221	0.327547479	-3.23347389	0.001222945	0.007938035
Calm1	17900.38094	0.450209531	0.072536003	6.206704461	5.41E-10	1.32E-08
Calm2	5644.146631	-0.570110269	0.078889254	-7.22671644	4.95E-13	1.79E-11
Calm3	1146.729168	0.438357598	0.099622522	4.400185714	1.08E-05	0.000125727
Calml4	29.13033271	1.816840558	0.308314906	5.892808048	3.80E-09	8.17E-08
Calr	5542.886057	0.244399581	0.092662065	2.637536536	0.008351063	0.039596967
Camk1	1779.496551	0.776695803	0.093278781	8.326607574	8.32E-17	4.97E-15
Camk1d	1361.759111	0.240313667	0.087044625	2.760809948	0.005765822	0.029461249
Camk2b	51.88402549	-1.697540077	0.248665592	-6.826598188	8.70E-12	2.70E-10
Camk2d	704.3985772	0.261459409	0.085322418	3.06436943	0.002181294	0.012979649
Camkk2	1348.322813	0.751159042	0.077418699	9.702553139	2.94E-22	3.24E-20
Camta1	98.90281224	0.3866644	0.160541124	2.40850686	0.016017925	0.066312001
Camta2	637.2720448	-0.371543964	0.086570974	-4.291784507	1.77E-05	0.000195951
Canx	4692.697538	0.238864799	0.060147917	3.97128964	7.15E-05	0.000672161
Cap2	4.640348906	-0.818812609	0.380858448	-2.149913212	0.03156208	0.113583867
Capg	2581.477444	-0.600619597	0.10118296	-5.935975723	2.92E-09	6.36E-08
Capn1	229.4986639	-0.270979685	0.138219698	-1.96049976	0.049937406	0.158203642
Capn2	676.418053	0.400350828	0.136717614	2.928304679	0.003408159	0.018950071
Capn5	90.80596134	-0.45547158	0.170196107	-2.676157449	0.007447165	0.036179866
Caprin1	1318.468735	0.26713885	0.088119051	3.031567488	0.002432875	0.014211818
Capza1	281.9313193	0.279853733	0.115870965	2.415218796	0.01572576	0.065318705
Car2	6.970712088	-1.055149506	0.372218826	-2.834755879	0.004586072	0.024353565
Card6	42.22595299	-0.43095229	0.218435698	-1.972902294	0.048506695	0.154733765
Carhsp1	247.1606809	0.324595694	0.113141601	2.868933194	0.004118588	0.022265003
Casc4	28.25926918	-0.890265421	0.268976894	-3.309821179	0.000933556	0.006287122
Cask	287.1571395	-0.366803968	0.112874576	-3.249659764	0.001155432	0.007548786

Casp1	942.0779	-0.3918048	0.101482182	-3.860823576	0.000113005	0.001008248
Casp12	26.2007306	-0.723406872	0.300975242	-2.403542786	0.016237067	0.067071198
Casp2	175.7121708	-0.358199034	0.137658139	-2.602091209	0.009265721	0.042771661
Casp4	177.4431931	-0.493486733	0.163990421	-3.009241217	0.002619011	0.015192693
Casp8	803.1901133	0.465435136	0.113078843	4.116023162	3.85E-05	0.000389279
Casp8ap2	481.8880519	0.244792132	0.107231428	2.282839437	0.022439828	0.087022914
Casp9	285.7220742	-0.270322886	0.121950638	-2.216658235	0.026646455	0.099597347
Cass4	36.82753136	0.779587405	0.264504945	2.947345295	0.003205151	0.018021044
Cast	758.773987	-0.305218492	0.134412661	-2.270757005	0.023161692	0.089354116
Cat	3671.308923	-0.496688854	0.122320895	-4.060539746	4.90E-05	0.000480425
Cav2	477.6854952	0.319544466	0.141353539	2.260604642	0.02378375	0.091298095
Cbfb	906.9998512	0.339030999	0.086088252	3.938179626	8.21E-05	0.0007615
Cbl	4857.606419	-0.415338268	0.086393095	-4.807540078	1.53E-06	2.11E-05
Cbr2	601.7189192	4.919599498	0.19082958	25.7800677	1.48E-146	1.11E-142
Cbr3	233.9302295	-0.341348996	0.143324907	-2.381644635	0.017235521	0.070401051
Cbwd1	36.71251381	0.561943905	0.237146796	2.369603615	0.017807165	0.072224898
Cbx5	705.1925038	0.626149325	0.117745703	5.317810411	1.05E-07	1.78E-06
Cbx6	305.9653148	0.653203195	0.114979082	5.681061145	1.34E-08	2.65E-07
Cc2d1b	275.0156377	0.25147662	0.116447115	2.159577936	0.030805358	0.111289361
Cc2d2a	63.78060328	0.775681107	0.205799158	3.769117013	0.000163826	0.001392939
Ccbe1	25.54792446	-1.247410183	0.285989062	-4.361740881	1.29E-05	0.000147815
Ccdc112	119.8789785	-0.996423497	0.156400056	-6.370991934	1.88E-10	4.94E-09
Ccdc115	532.066913	0.503146596	0.095966772	5.242925074	1.58E-07	2.60E-06
Ccdc125	181.7157166	0.352976699	0.133612079	2.641802308	0.008246618	0.039250552
Ccdc14	32.78535562	1.055353885	0.263727531	4.001682652	6.29E-05	0.000600028
Ccdc141	6.195971155	-0.872704562	0.375674412	-2.323034348	0.020177307	0.079936867
Ccdc149	8.110350832	1.060948392	0.365796194	2.900381165	0.003727091	0.020458747
Ccdc163	58.70957583	-0.392847216	0.189279686	-2.075485355	0.037941575	0.129760531
Ccdc18	28.24714882	1.118975882	0.266593292	4.197314471	2.70E-05	0.000283453
Ccdc191	89.08557105	-0.490997164	0.171036597	-2.870714062	0.004095458	0.022171911
Ccdc22	166.036029	0.322657288	0.137184765	2.351990685	0.018673243	0.075007856
Ccdc25	310.5638648	0.464132522	0.12289687	3.776601636	0.000158983	0.001356369
Ccdc34	364.6479719	0.559008367	0.137208229	4.074160655	4.62E-05	0.000456844
Ccdc6	267.9618867	-0.420090864	0.116718328	-3.599185071	0.000319216	0.002506789
Ccdc71l	554.611012	0.204480646	0.08166115	2.504013788	0.012279325	0.053740399
Ccdc80	206.803699	-0.975542601	0.156460603	-6.235068663	4.52E-10	1.12E-08
Ccdc88a	1974.476568	-0.453481218	0.104752197	-4.329085513	1.50E-05	0.000168314
Ccdc9	214.6360387	-0.266938712	0.115430168	-2.312555872	0.02074707	0.081740951
Ccdc93	239.5644496	0.414827251	0.123780961	3.351300943	0.000804328	0.005524282
Ccdc97	300.763271	-0.21332678	0.103261904	-2.065880745	0.038839741	0.132199592
Ccl12	21.92837012	3.268769164	0.364816754	8.96003028	3.25E-19	2.62E-17
Ccl17	5.155855857	-0.75901096	0.381125774	-1.991497329	0.046426239	0.150641851
Ccl2	470.8009529	2.103058624	0.169596921	12.40033497	2.60E-35	8.68E-33
Ccl24	372.0219318	0.931632713	0.175809361	5.299107541	1.16E-07	1.96E-06

Ccl3	393.0401411	0.853346966	0.103437735	8.249861307	1.59E-16	9.01E-15
Ccl4	163.4719667	1.646055625	0.219392303	7.502795681	6.25E-14	2.51E-12
Ccl7	188.8559249	2.739675029	0.229755593	11.92430179	8.84E-33	2.41E-30
Ccl9	3306.750615	0.405870944	0.114790432	3.535755889	0.00040661	0.00309589
Ccn1	29.8572727	-1.26116183	0.308913841	-4.082568224	4.45E-05	0.000441786
Ccn2	72.70470927	-2.104824532	0.275408169	-7.6425639	2.13E-14	9.18E-13
Ccn4	108.8814926	-1.087344712	0.187766206	-5.790950009	7.00E-09	1.44E-07
Ccna2	210.539364	1.993563795	0.227755508	8.753087064	2.08E-18	1.53E-16
Ccnb1	107.1957366	1.80448472	0.250197952	7.212228176	5.50E-13	1.97E-11
Ccnb2	113.3816684	2.176265856	0.243934188	8.921528672	4.60E-19	3.63E-17
Ccnd1	2094.110945	2.893661336	0.22227133	13.01859911	9.59E-39	4.23E-36
Ccnd2	484.5987425	-0.473539578	0.135590333	-3.49242875	0.000478649	0.003554225
Ccnd3	408.8167698	0.966981613	0.103559607	9.337440003	9.87E-21	9.26E-19
Ccne1	52.7919598	0.946035359	0.248793902	3.802486123	0.000143251	0.001240492
Ccne2	80.07545546	1.985685535	0.253672224	7.827760976	4.97E-15	2.35E-13
Ccnf	53.3250517	1.549973224	0.28136411	5.508780853	3.61E-08	6.63E-07
Ccnh	332.3551682	0.257508437	0.105338306	2.444584939	0.014501893	0.061195986
Ccnl1	1287.601572	-0.284145131	0.108948652	-2.608064672	0.009105575	0.042214199
Ccpg1	390.5397382	-0.423453594	0.101664329	-4.165213084	3.11E-05	0.000321053
Ccr1	1503.182186	0.84420802	0.118849123	7.103190997	1.22E-12	4.20E-11
Ccr5	1017.213232	-0.713576402	0.164510789	-4.337565991	1.44E-05	0.000162316
Ccr7	22.3263984	0.968846582	0.322448156	3.00465847	0.002658792	0.01538184
Ccrl2	195.6599941	-1.502598966	0.149012521	-10.08370948	6.52E-24	8.90E-22
Cct8	1628.55251	0.291022654	0.076666236	3.795968995	0.000147068	0.001270609
Cd14	1408.579497	0.272673037	0.118939264	2.29254014	0.021874494	0.085274341
Cd151	555.1019017	-0.726513958	0.116710081	-6.224946039	4.82E-10	1.19E-08
Cd163	287.6105689	2.202612	0.293973544	7.49255178	6.75E-14	2.70E-12
Cd164	1924.364452	0.801809466	0.126918611	6.317508964	2.66E-10	6.85E-09
Cd180	918.0032303	-0.613496867	0.101877241	-6.021922683	1.72E-09	3.90E-08
Cd19	98.05410054	0.785083211	0.252864295	3.10476104	0.001904328	0.011523488
Cd2	32.55319992	0.726965043	0.29960774	2.426389397	0.015249898	0.063801287
Cd200	84.60531818	-1.02310554	0.209813758	-4.876255727	1.08E-06	1.55E-05
Cd200r3	18.12386803	-0.840149553	0.315543442	-2.662547975	0.007755151	0.037433759
Cd200r4	511.9457667	0.477662962	0.121324243	3.937077612	8.25E-05	0.00076406
Cd209c	7.984786511	0.948087216	0.380072913	2.494487726	0.012613909	0.054948311
Cd209f	65.05199113	3.394433349	0.316149208	10.73680801	6.84E-27	1.18E-24
Cd209g	4.986310794	1.439465861	0.379633228	3.791727795	0.000149603	0.001288061
Cd22	275.143433	-0.979081426	0.149459735	-6.550803979	5.72E-11	1.59E-09
Cd244a	122.274046	1.611203953	0.195181112	8.254917391	1.52E-16	8.71E-15
Cd274	372.2721155	-1.194220697	0.264230627	-4.519614974	6.20E-06	7.55E-05
Cd276	56.27376023	-0.761914809	0.272748215	-2.793473123	0.005214536	0.027096449
Cd28	426.1048857	1.286574454	0.113960282	11.28967419	1.48E-29	3.21E-27
Cd300a	796.1630884	-0.552249378	0.114379981	-4.828199601	1.38E-06	1.93E-05
Cd300c2	986.1695638	-0.210250092	0.100805316	-2.0857044	0.037005408	0.127605734

Cd300e	4.296236301	0.907132487	0.378049804	2.399505241	0.016417245	0.067666463
Cd300lb	2462.951813	-0.426421708	0.099872837	-4.269646503	1.96E-05	0.000213526
Cd300ld	5273.272489	0.235678635	0.100776562	2.338625465	0.019354826	0.07716734
Cd300lf	103.1245928	1.097348052	0.326361715	3.362367583	0.000772772	0.005339312
Cd300lg	36.36694573	1.03439845	0.264488209	3.910943524	9.19E-05	0.000840494
Cd320	59.9908929	-0.471805309	0.228477427	-2.064997472	0.038923239	0.132423726
Cd33	1205.719645	1.19829488	0.117065281	10.2361253	1.37E-24	1.99E-22
Cd34	39.70298975	0.617785054	0.240594897	2.56773964	0.010236401	0.046241967
Cd36	17790.90463	-0.941097483	0.15095464	-6.234306422	4.54E-10	1.12E-08
Cd37	808.6109224	-0.350513718	0.103135189	-3.398585116	0.000677354	0.004763378
Cd38	514.0703428	0.776328255	0.336123129	2.309654377	0.020907297	0.082199581
Cd40	184.06797	0.310869721	0.128929654	2.411157649	0.015901973	0.065922904
Cd48	1769.869306	-0.291162277	0.12167698	-2.392911756	0.016715261	0.068630894
Cd52	4508.025054	-0.684616023	0.090307425	-7.580949421	3.43E-14	1.44E-12
Cd59a	107.7702851	1.441806798	0.172368577	8.364673099	6.03E-17	3.66E-15
Cd5l	706.3991744	-1.473110205	0.270552368	-5.444824661	5.19E-08	9.28E-07
Cd63	11052.98032	-0.428297144	0.109987851	-3.894040468	9.86E-05	0.000893966
Cd63-ps	112.1616248	-0.427381123	0.201460308	-2.121416016	0.033886809	0.119847042
Cd68	10667.58313	-0.239171981	0.118861687	-2.012187334	0.044200193	0.144877502
Cd74	1514.804705	-1.556187256	0.26956814	-5.772890124	7.79E-09	1.60E-07
Cd80	46.21396149	0.68177598	0.210580843	3.237597351	0.001205408	0.007844561
Cd81	1574.491511	0.759236051	0.074403452	10.20431222	1.90E-24	2.71E-22
Cd82	164.7876322	-0.347074399	0.151838659	-2.285810486	0.022265348	0.086518922
Cd83	147.0492165	-0.414532583	0.139719992	-2.966880951	0.003008374	0.017075141
Cd84	4696.207437	-0.566401842	0.070705209	-8.010751272	1.14E-15	5.88E-14
Cd86	403.8646945	-0.341105029	0.158772832	-2.148384104	0.031683255	0.113776167
Cd9	4335.185274	-0.324903364	0.146301407	-2.220780854	0.026365808	0.098745116
Cd93	7675.276359	0.188976054	0.081484025	2.319179157	0.020385324	0.080590769
Cd99l2	223.0916177	0.30780184	0.126768187	2.428068479	0.015179477	0.063565757
Cdc20	121.4077135	1.075908662	0.205557797	5.234093183	1.66E-07	2.71E-06
Cdc25a	182.9249573	0.725740281	0.152187472	4.768725526	1.85E-06	2.52E-05
Cdc25b	155.5953239	1.333865667	0.177410227	7.51853876	5.54E-14	2.25E-12
Cdc25c	8.777946104	0.893112621	0.361770536	2.468726813	0.013559469	0.058105926
Cdc27	324.4684807	0.267769351	0.128276349	2.087441313	0.036848261	0.127209995
Cdc34	247.5086834	0.218221019	0.108173493	2.017324321	0.043661679	0.143627974
Cdc42	6199.044753	0.320577754	0.085584535	3.745743934	0.00017986	0.001514679
Cdc42bpa	144.3777762	-0.378047734	0.13469366	-2.806722569	0.005004832	0.026197252
Cdc42bpg	138.446457	0.626453071	0.146691938	4.270535105	1.95E-05	0.000212831
Cdc42ep3	229.4487184	0.435105507	0.158335681	2.747994034	0.00599611	0.030441009
Cdc42se1	1012.472795	0.295761352	0.077142549	3.83395878	0.000126097	0.001111834
Cdc42se2	856.7149145	-0.173031153	0.072931434	-2.372518193	0.017667293	0.071715733
Cdc45	52.08949134	1.421264414	0.246509307	5.765560874	8.14E-09	1.66E-07
Cdc6	56.8165905	1.713890057	0.227720342	7.526293167	5.22E-14	2.14E-12
Cdc7	28.96342729	1.339080445	0.267858514	4.999208077	5.76E-07	8.61E-06

Cdca2	101.3557857	1.950494474	0.255753683	7.626457042	2.41E-14	1.03E-12
Cdca3	105.5349658	2.052932233	0.250199067	8.205195391	2.30E-16	1.29E-14
Cdca5	25.87183877	1.235219524	0.288639654	4.279451932	1.87E-05	0.000206277
Cdca7	27.7094866	0.974265513	0.283457664	3.43707593	0.000588031	0.004234442
Cdca7l	111.8580499	0.415555406	0.184022729	2.258174352	0.023934792	0.091606585
Cdca8	192.3414664	1.536219963	0.209633329	7.328128462	2.33E-13	8.80E-12
Cdh11	92.59336322	-1.323343332	0.172375184	-7.67711049	1.63E-14	7.16E-13
Cdh2	38.15413183	-1.341228346	0.298696365	-4.490273407	7.11E-06	8.57E-05
Cdh3	9.359009345	-0.814543309	0.369349424	-2.205346095	0.027429818	0.101622184
Cdip1	184.0200105	-0.486713509	0.125789205	-3.869278841	0.000109158	0.000980329
Cdipt	536.0803082	-0.208256783	0.083412236	-2.496717434	0.01253488	0.054683412
Cdk1	425.2124211	1.847453901	0.183397507	10.07349519	7.24E-24	9.61E-22
Cdk10	128.5687014	-0.444250446	0.140198668	-3.168720875	0.001531114	0.009634139
Cdk11b	678.0425306	-0.206603301	0.099846504	-2.069209153	0.038526464	0.13131198
Cdk14	693.027905	0.42517717	0.128382782	3.311792784	0.000927002	0.006255178
Cdk18	159.943665	-1.204475331	0.1773793	-6.790393975	1.12E-11	3.38E-10
Cdk19	517.4421901	-0.659052682	0.104754053	-6.291428929	3.15E-10	8.01E-09
Cdk2	103.3478173	0.612512155	0.166148654	3.686530952	0.000227332	0.001867306
Cdk2ap2	558.9292185	-0.69812195	0.08961845	-7.789935567	6.70E-15	3.10E-13
Cdk4	563.7376742	0.361660498	0.113204281	3.194759891	0.001399472	0.008880287
Cdk5r1	45.84724322	1.278677615	0.227553664	5.61923545	1.92E-08	3.68E-07
Cdk6	1091.25414	0.461115842	0.098826777	4.665899821	3.07E-06	3.99E-05
Cdkn1b	464.8160936	-0.284043676	0.106665799	-2.662931138	0.007746326	0.037403192
Cdkn1c	100.4554207	-0.89299919	0.234640452	-3.805819421	0.000141335	0.001226024
Cdkn2aipnl	399.140496	0.379934874	0.110713601	3.431691044	0.00059983	0.004304953
Cdkn2c	90.52379322	1.400041361	0.233137339	6.005221489	1.91E-09	4.29E-08
Cdkn2d	147.5573407	0.85918782	0.164005214	5.238783581	1.62E-07	2.65E-06
Cdkn3	60.19268163	2.253245895	0.279685261	8.056362659	7.86E-16	4.14E-14
Cdo1	17.85530726	-1.979353901	0.358626131	-5.519268486	3.40E-08	6.30E-07
Cdon	21.74841572	-0.674551904	0.293529031	-2.298075599	0.021557487	0.084270176
Cdr2l	28.21701673	-0.625452863	0.267338258	-2.33955614	0.01930667	0.077016266
Cds1	239.4935757	0.352066665	0.122823072	2.866453824	0.004150987	0.022407865
Cds2	728.6502979	-0.573393736	0.109627019	-5.230405269	1.69E-07	2.76E-06
Cdt1	344.7537907	0.720898861	0.125139445	5.760764407	8.37E-09	1.71E-07
Ceacam1	111.1399991	0.5651009	0.162586368	3.475696695	0.000509528	0.003733925
Cebpa	1650.312229	-0.47008384	0.09545098	-4.924871795	8.44E-07	1.23E-05
Cebpb	1721.069329	0.307695412	0.106452666	2.890443459	0.003846987	0.021016286
Cebpd	498.730251	0.941028887	0.121552232	7.741765563	9.80E-15	4.43E-13
Cebpg	1003.747159	-0.318752464	0.081455238	-3.913222443	9.11E-05	0.000834387
Cebpzos	206.9856265	0.509605934	0.130515131	3.904573605	9.44E-05	0.000859027
Cela1	111.149829	2.353766141	0.196167795	11.99873882	3.61E-33	1.02E-30
Celf4	42.1493393	-0.846243663	0.221974443	-3.81234727	0.000137653	0.001199355
Cemip	4.242923	-1.620564565	0.378826553	-4.277853681	1.89E-05	0.000207312
Cemip2	601.6207358	0.613489844	0.098522965	6.226871541	4.76E-10	1.17E-08

Cenpa	306.6432665	0.346028065	0.145036759	2.385795621	0.017042219	0.069707893
Cenpb	666.7183377	0.219384706	0.093202791	2.353842669	0.018580473	0.074733688
Cenpc1	184.5265777	0.328317262	0.127375744	2.577549325	0.009950368	0.045250052
Cenpe	331.4864151	1.55719745	0.182214025	8.54598022	1.27E-17	8.54E-16
Cenpf	314.5524712	2.093811278	0.180395308	11.60679457	3.81E-31	9.68E-29
Cenph	77.82648993	1.674987633	0.239851258	6.983443185	2.88E-12	9.63E-11
Cenpi	41.9405332	2.084181819	0.288472554	7.224887734	5.02E-13	1.80E-11
Cenpk	56.37385084	1.434339173	0.252627372	5.677687102	1.37E-08	2.70E-07
Cenpl	143.0916108	0.45202487	0.171715142	2.632411248	0.008478117	0.04003496
Cenpm	36.1312932	1.576086145	0.29651034	5.315450878	1.06E-07	1.80E-06
Cenpn	49.05843769	1.55297445	0.247512493	6.274327528	3.51E-10	8.86E-09
Cenpo	65.9910339	0.442445843	0.200554415	2.2061137	0.02737604	0.101515253
Cenpp	23.7349173	1.594201566	0.31162411	5.115783775	3.12E-07	4.87E-06
Cenpq	90.09775975	1.612490367	0.210896191	7.645896109	2.07E-14	8.97E-13
Cenps	37.7220375	1.538985843	0.301012445	5.112698392	3.18E-07	4.93E-06
Cenpu	40.76225977	0.986699953	0.25127468	3.926778264	8.61E-05	0.000792135
Cenpw	86.32730342	1.150824072	0.203197736	5.663567389	1.48E-08	2.91E-07
Cenpx	207.6753802	0.441460113	0.144397954	3.057246304	0.002233806	0.013203124
Cep19	62.76289675	0.406495526	0.206224078	1.971135138	0.048708421	0.155194749
Cep250	195.2965475	0.347596292	0.140395384	2.475838456	0.013292371	0.057255628
Cep41	68.67891839	0.927682088	0.207960394	4.460859453	8.16E-06	9.73E-05
Cep55	49.47325623	1.872115572	0.279407376	6.700308333	2.08E-11	6.06E-10
Cep57	346.74425	0.25144856	0.111421094	2.256741079	0.024024258	0.091878707
Cep63	133.3741762	-0.551542806	0.141159562	-3.907229496	9.34E-05	0.000850158
Cep68	124.3319806	0.834596556	0.181620912	4.595266838	4.32E-06	5.48E-05
Cep70	150.1115057	-0.392687103	0.142599167	-2.753782582	0.005891089	0.030009359
Cep78	51.52361293	0.633296202	0.204490722	3.096943445	0.001955272	0.011788977
Cep83	283.5384673	0.283216029	0.133062714	2.12844019	0.033300604	0.118114432
Cerk	1607.628137	-0.424736656	0.080043562	-5.306318784	1.12E-07	1.89E-06
Cers4	113.1949433	0.638817588	0.165411134	3.86199873	0.000112463	0.001004604
Cers6	914.5436439	-0.791939473	0.090352684	-8.764980021	1.87E-18	1.39E-16
Ces2g	12.83221103	-1.606932637	0.340580026	-4.718223368	2.38E-06	3.16E-05
Cetn3	1016.997223	0.339699764	0.110703725	3.068548622	0.002151013	0.012824893
Cfap161	3.962243834	-0.93029276	0.381239387	-2.440180085	0.014679942	0.061795761
Cfap45	11.92578639	1.196850326	0.343291265	3.48639901	0.00048957	0.003612529
Cfdp1	714.8161632	0.238315838	0.09304753	2.561226908	0.010430321	0.046991241
Cfl1	2027.399564	0.314877296	0.103599493	3.039371018	0.002370727	0.013901145
Cflar	892.2138932	0.414387388	0.1043993	3.969254464	7.21E-05	0.000676656
Cfp	4095.354146	1.203854404	0.135804492	8.864614022	7.68E-19	5.91E-17
Cgas	145.992572	1.001640666	0.16184619	6.188843051	6.06E-10	1.47E-08
Cggbp1	1143.984116	0.145521465	0.069825367	2.084077328	0.037153134	0.127909401
Cgnl1	1171.495692	0.230065985	0.112427746	2.04634526	0.040722414	0.136923828
Ch25h	11.87585361	0.693120771	0.340102915	2.037973626	0.041552571	0.138758217
Chaf1a	94.1132308	1.578266135	0.229750908	6.869466373	6.44E-12	2.04E-10

Chaf1b	48.2191867	1.044882211	0.261457631	3.996372968	6.43E-05	0.000611308
Chchd1	481.8882986	0.219004906	0.105801743	2.069955553	0.038456507	0.131133106
Chchd10	152.5679174	0.427047133	0.168523194	2.534055535	0.011275086	0.050194369
Chchd2	5674.753392	0.242294588	0.093281252	2.597462874	0.009391527	0.043192966
Chchd5	87.13097064	-0.350601026	0.162299312	-2.160212642	0.030756212	0.111138568
Chd3	1016.353328	0.20777728	0.092165825	2.254385281	0.02417194	0.092349366
Chek1	32.02621486	1.649919523	0.314949645	5.238677202	1.62E-07	2.65E-06
Chek2	94.53273056	0.545641821	0.178513519	3.05658543	0.002238737	0.013214017
Chid1	138.831541	0.328625293	0.143689547	2.287050801	0.022192858	0.086304281
Chil1	10.29565623	-1.920686709	0.367244889	-5.229988947	1.70E-07	2.77E-06
Chkb	286.9805257	-0.202045854	0.102313276	-1.974776504	0.048293517	0.154287898
Chm	597.9736872	0.197856846	0.093792458	2.109517664	0.034899923	0.122369893
Chml	165.3519805	0.322832384	0.153033904	2.109548119	0.034897297	0.122369893
Chmp3	1649.232705	0.261114881	0.083363171	3.132257051	0.001734679	0.010686507
Chmp5	1642.861379	0.158029252	0.079534819	1.986919111	0.046931361	0.151760511
Chmp7	205.572555	-0.307934756	0.125053276	-2.462428535	0.013799967	0.058917811
Chn2	55.56997038	-0.590510834	0.200868673	-2.939785616	0.003284394	0.018377667
Chordc1	727.8920816	0.295569058	0.09192932	3.215177236	0.00130364	0.008371299
Chpf	45.13099816	-1.203500597	0.213991886	-5.624047805	1.87E-08	3.58E-07
Chpf2	272.1407106	-0.231656776	0.106918395	-2.166669047	0.030260098	0.109885033
Chst11	204.1376461	-0.709032052	0.182921447	-3.876155937	0.00010612	0.000955905
Chst14	151.4902187	0.367678201	0.144348238	2.547160988	0.010860332	0.048680108
Chst15	86.33282054	-0.669217148	0.206537548	-3.240171854	0.001194577	0.007780822
Chst2	11.87462912	-1.190010245	0.347161713	-3.42782686	0.000608433	0.004356278
Chsy1	391.9224872	-0.326719242	0.129121834	-2.530317551	0.011395934	0.050597271
Chtf18	16.96938396	0.891750249	0.335776323	2.655786569	0.007912367	0.037984988
Ciao2a	859.3330658	0.423397156	0.103364542	4.09615471	4.20E-05	0.000419985
Ciapin1	273.0735833	0.30949159	0.115451071	2.68071649	0.007346473	0.035760142
Ciart	6.632855191	0.783905455	0.376005872	2.084822373	0.037085427	0.127758618
Cib1	272.8602378	0.368162032	0.112030968	3.286252346	0.0010153	0.006762809
Cib2	42.20230571	-1.041398676	0.220034005	-4.732898798	2.21E-06	2.95E-05
Cic	907.9913051	-0.621609814	0.092574486	-6.714699075	1.88E-11	5.52E-10
Cip2a	135.2929311	1.370439004	0.218747621	6.264932138	3.73E-10	9.39E-09
Cipc	286.958452	-0.472268626	0.13798384	-3.422637223	0.000620168	0.004427621
Cirbp	204.6242434	-0.324968477	0.145513793	-2.233248611	0.025532554	0.096369981
Cisd2	898.7404205	-0.316216317	0.083653934	-3.780053145	0.000156795	0.001340212
Cit	91.10657256	2.302717463	0.235682523	9.770420967	1.51E-22	1.74E-20
Ciz1	294.7423125	-0.460824933	0.11573386	-3.981764131	6.84E-05	0.000647266
Ckap2	67.6943767	1.33703207	0.273987179	4.879907432	1.06E-06	1.52E-05
Ckap2l	190.1068495	1.716856564	0.187609167	9.151240272	5.63E-20	4.80E-18
Ckap5	325.3760928	0.279112977	0.126945658	2.19868076	0.02790063	0.102998442
Cklf	259.9331152	0.250956086	0.106094293	2.365406085	0.018010311	0.072851949
Cks1b	330.310248	1.011709596	0.193665473	5.224006029	1.75E-07	2.84E-06
Clca3a2	2.508704251	-1.132429694	0.363440018	-3.11586407	0.001834068	0.011179471

Clcf1	60.00156308	-0.482490779	0.236197735	-2.042740922	0.041078092	0.137541035
Clcn1	2.111621884	-0.773559906	0.359919448	-2.149258426	0.03161392	0.113635952
Clcn5	824.1938575	0.832165599	0.107366629	7.750691328	9.14E-15	4.14E-13
Clcn7	680.3884714	-0.561465731	0.097491985	-5.759096293	8.46E-09	1.72E-07
Clec10a	212.2318739	0.876731424	0.322889472	2.715267915	0.006622217	0.032928965
Clec12a	1217.565675	-1.06577469	0.12376122	-8.611539978	7.21E-18	4.96E-16
Clec1a	32.11203354	-1.418280788	0.29324642	-4.836481175	1.32E-06	1.86E-05
Clec1b	109.8864553	-2.771966334	0.241022489	-11.50086178	1.31E-30	3.21E-28
Clec2d	58.93562135	-0.581694812	0.232028052	-2.507002091	0.012175999	0.053453042
Clec2i	66.92820274	-0.701424749	0.203894277	-3.440139466	0.000581414	0.004192834
Clec4a2	1186.247245	0.234660864	0.10983371	2.136510404	0.032637834	0.116313785
Clec4a3	1294.370597	-0.422225286	0.115741524	-3.648001772	0.000264288	0.002135793
Clec4b1	147.5262956	-0.725084009	0.160867564	-4.50733505	6.56E-06	7.96E-05
Clec4d	6844.548022	0.352042702	0.078248651	4.499025835	6.83E-06	8.24E-05
Clec4e	644.8007754	1.025027893	0.124032218	8.264206769	1.41E-16	8.12E-15
Clec4n	3830.524638	0.714410606	0.127464524	5.604779946	2.09E-08	3.96E-07
Clec5a	437.0083879	1.145114783	0.126899409	9.023799207	1.82E-19	1.50E-17
Clec7a	10230.82948	-1.222737899	0.124436842	-9.826172729	8.69E-23	1.02E-20
Clec9a	24.7428215	-2.673713043	0.324381911	-8.242485027	1.69E-16	9.55E-15
Clic1	1252.202091	0.438249021	0.102935798	4.257498646	2.07E-05	0.000223996
Clic4	2581.590721	-0.549070462	0.104299422	-5.264367247	1.41E-07	2.34E-06
Clip1	1112.072948	-0.340045394	0.100480344	-3.384198144	0.000713865	0.004985094
Clip3	10.5592849	-0.740262748	0.369328346	-2.004348587	0.045032733	0.147042258
Clk1	789.0010111	-0.279012935	0.103990347	-2.683065735	0.007295065	0.035579149
Clk4	409.8025844	-0.646202243	0.116141907	-5.56390247	2.64E-08	4.97E-07
Clspn	111.1034791	2.031732394	0.217036686	9.36123947	7.88E-21	7.44E-19
Clta	3804.988911	0.524098425	0.092469201	5.667816072	1.45E-08	2.85E-07
Cltb	261.6802498	0.396148878	0.135993874	2.912990612	0.003579854	0.019751053
Cltc	6033.403149	0.159303569	0.072047799	2.211081695	0.02703018	0.100655562
Clybl	165.2289875	0.376380944	0.14219167	2.646997149	0.008121004	0.038763328
Cmah	71.72746456	1.165136554	0.243010183	4.79459971	1.63E-06	2.24E-05
Cmbl	42.78238986	-1.052959458	0.246882423	-4.265024003	2.00E-05	0.000217837
Cmc2	119.1650805	1.067690784	0.201880298	5.288731953	1.23E-07	2.06E-06
Cmtm7	161.4104888	0.388591807	0.150935387	2.574557327	0.010036845	0.045560477
Cnksr3	51.51160527	-0.6896795	0.201850846	-3.416777848	0.00063367	0.004504728
Cnn1	8.980241495	-1.244868667	0.363734995	-3.422460537	0.000620571	0.004428393
Cnn2	263.8625868	-0.340443244	0.127154493	-2.677398459	0.007419634	0.036081155
Cnn3	136.8412978	-0.499456673	0.15606496	-3.200312692	0.001372786	0.008729404
Cnnm2	54.62255004	0.649268341	0.196538825	3.303511876	0.000954819	0.006408306
Cnnm3	182.467537	-0.392643498	0.133827458	-2.93395319	0.003346747	0.018694203
Cnnm4	70.11634208	0.368560155	0.177170407	2.080257996	0.037501875	0.128742466
Cnot6	1341.592069	0.165864306	0.083920029	1.976456738	0.048103071	0.154009805
Cnot6l	1845.746201	-0.257950165	0.073332861	-3.517524893	0.000435591	0.003289845
Cnot9	249.5344045	0.382277622	0.135489865	2.82144809	0.004780737	0.025200044

Cnp	353.5406433	0.444224861	0.102313327	4.341808403	1.41E-05	0.000159812
Cnppd1	1062.77473	-0.384567188	0.089137678	-4.31430567	1.60E-05	0.000178905
Cnpy4	234.3672074	0.578819514	0.117345834	4.932595352	8.11E-07	1.19E-05
Cnr2	42.28841403	-0.542071539	0.255622369	-2.120595085	0.033955892	0.11995705
Cnrip1	184.8900836	0.689272112	0.175690953	3.923207768	8.74E-05	0.000803479
Coa5	720.6903416	-0.180497047	0.0813275	-2.21938515	0.026460533	0.099025741
Col15a1	23.28763685	-0.953242674	0.298343573	-3.195117172	0.001397741	0.008873053
Col1a1	1451.882199	-1.370983695	0.15612857	-8.781119926	1.62E-18	1.22E-16
Col1a2	2511.055177	-1.228301965	0.148281648	-8.283573739	1.20E-16	6.93E-15
Col24a1	15.88673879	-0.73423173	0.32148016	-2.283909932	0.022376825	0.08689726
Col4a1	127.9702851	-1.458079331	0.176482768	-8.261879334	1.43E-16	8.25E-15
Col4a2	67.16332543	-1.398915163	0.186182782	-7.513665608	5.75E-14	2.33E-12
Col4a3bp	698.1251872	0.425301966	0.083543774	5.09076793	3.57E-07	5.49E-06
Col4a5	17.72411676	-1.235942953	0.300725118	-4.109876029	3.96E-05	0.000398982
Col4a6	2.152190305	-0.797579314	0.364204962	-2.189918857	0.028530122	0.104836323
Col5a1	104.8873232	-1.118880826	0.187744336	-5.959598316	2.53E-09	5.55E-08
Col5a2	124.0137959	-1.382767708	0.170976121	-8.087490231	6.09E-16	3.24E-14
Col5a3	7.873978971	-1.168618721	0.37875957	-3.085384007	0.002032895	0.012193307
Col6a1	94.94249564	-1.000389104	0.173959919	-5.750687352	8.89E-09	1.80E-07
Col6a2	104.6755626	-1.33846455	0.196727411	-6.803650521	1.02E-11	3.11E-10
Col6a3	66.692547	-1.026016873	0.199200908	-5.150663638	2.60E-07	4.11E-06
Colgalt1	2551.098358	-0.363426925	0.076115292	-4.774689996	1.80E-06	2.45E-05
Commd1	215.8888273	0.299939562	0.13250383	2.263629372	0.02359692	0.09066026
Commd10	455.7769564	0.410191505	0.096954009	4.230784351	2.33E-05	0.000248386
Commd5	140.9539931	0.295262275	0.146923409	2.009633975	0.044469943	0.145564288
Commd6	247.2481842	0.294834562	0.108300276	2.72238052	0.006481347	0.03238287
Commd8	846.4571788	0.406883518	0.079170705	5.139319122	2.76E-07	4.34E-06
Commd9	98.5643294	0.484465976	0.19346846	2.504108294	0.012276045	0.053740399
Comt	1428.721524	0.212552113	0.082381585	2.580092559	0.009877384	0.045013634
Comtd1	195.7547329	-0.578579359	0.154259993	-3.750676672	0.000176358	0.001488529
Cop1	344.7477748	-0.425143622	0.098237628	-4.327706506	1.51E-05	0.000169244
Coprs	283.0113302	0.954113368	0.138176591	6.905029023	5.02E-12	1.62E-10
Coq10a	98.21143268	-0.369831564	0.159123525	-2.324179055	0.020115899	0.079741226
Coq7	284.0683161	0.311797418	0.157692427	1.977250418	0.048013331	0.153966418
Coro1b	1497.733478	-0.319330071	0.078763987	-4.054264957	5.03E-05	0.000491472
Coro2a	116.0697248	-1.362862285	0.197202541	-6.910977316	4.81E-12	1.57E-10
Coro2b	7.577591807	1.183816896	0.371492175	3.186653653	0.00143929	0.009104692
Cox14	480.7919351	0.530940767	0.104145884	5.098048475	3.43E-07	5.30E-06
Cox5a	2335.944466	0.475652188	0.099173279	4.796172839	1.62E-06	2.23E-05
Cox6b1	1761.202327	0.1697007	0.086215529	1.96833102	0.049029963	0.156020495
Cox7a1	27.63351204	1.63195527	0.281915427	5.788811509	7.09E-09	1.46E-07
Cox7a2l	1165.368187	-0.229062812	0.086912886	-2.635544881	0.008400232	0.03972894
Cox7b	1423.957169	0.23326687	0.091307168	2.554748728	0.010626448	0.047768408
Cox7b2	1.853661031	0.918832596	0.340222851	2.700678672	0.006919816	

Cp	204.1786553	-0.644754415	0.325877288	-1.978519029	0.047870184	0.153764523
Cpd	966.5994122	0.29681909	0.105453367	2.814695227	0.004882351	0.025627648
Cpe	69.45192772	-0.650812191	0.208355959	-3.12355929	0.001786779	0.010949035
Cpeb1	114.5482981	-1.26458177	0.197588674	-6.400072138	1.55E-10	4.12E-09
Cpeb2	219.6208375	-0.295694478	0.120524052	-2.453406373	0.014151037	0.060023916
Cped1	130.1567077	-1.246323861	0.197085195	-6.323782282	2.55E-10	6.59E-09
Cpne2	633.0798536	0.357219521	0.094671681	3.773245802	0.000161137	0.001373191
Cpne3	1919.676764	0.209212468	0.067740914	3.08842109	0.002012231	0.012108081
Cpped1	257.4483051	0.279421285	0.115326941	2.422862183	0.015398767	0.064262876
Cpq	539.8787736	0.241876022	0.092821823	2.605809867	0.009165733	0.042388335
Cpsf2	772.859987	0.339210491	0.091770471	3.69629235	0.000218771	0.001809866
Cptp	69.98506653	0.485061455	0.180707328	2.684237887	0.007269536	0.035512346
Cpxm1	4.664877906	-0.836791988	0.381083112	-2.195825426	0.028104441	0.103601903
Crabp1	10.98090617	2.167147106	0.372898596	5.811625815	6.19E-09	1.29E-07
Cracr2b	110.2733108	-0.822543774	0.210834823	-3.901365824	9.57E-05	0.000868911
Cramp1l	303.3687462	-0.220309281	0.11063385	-1.991337022	0.046443848	0.150666413
Crat	425.4009824	-0.295675533	0.100330761	-2.947007779	0.003208651	0.018021044
Creb3l1	132.8118558	-0.604189377	0.174724319	-3.457958109	0.000544286	0.003967023
Creb3l2	271.1111343	-0.321767724	0.110749125	-2.905374856	0.003668135	0.020163993
Creb5	538.766303	-0.346144029	0.16004722	-2.162761893	0.030559498	0.110654054
Crebbp	1584.498642	-0.231800857	0.089402755	-2.592770845	0.009520618	0.043692942
Crebrf	1299.879881	-0.414852293	0.119192554	-3.480521898	0.000500438	0.003679604
Creg1	13969.58117	-0.926203656	0.113892609	-8.132254239	4.21E-16	2.29E-14
Creg2	93.27014472	-0.941567929	0.17753513	-5.303558408	1.14E-07	1.92E-06
Crem	152.8817697	0.487235623	0.142085199	3.429179297	0.000605409	0.004338768
Crim1	520.0861447	-0.226448223	0.11078467	-2.044039346	0.04094966	0.137259266
Crip1	2403.375419	-0.875981811	0.079346629	-11.03993735	2.45E-28	4.60E-26
Crip2	19.15086521	-1.157765461	0.289457115	-3.999782359	6.34E-05	0.000604099
Crlf2	1545.176932	-0.537069043	0.091981909	-5.838855122	5.26E-09	1.10E-07
Cryab	173.6375826	-1.462806808	0.177247222	-8.25291812	1.55E-16	8.82E-15
Cryba4	11.20110376	1.163589012	0.36031747	3.229343867	0.001240746	0.008032734
Crybg1	387.5977605	-0.394107551	0.110367428	-3.570868316	0.0003558	0.002748063
Cryl1	540.0203162	0.472239305	0.106085888	4.451480896	8.53E-06	0.00010117
Cryz	90.5212344	0.501847467	0.171785091	2.921368004	0.003484979	0.019298554
Cryzl1	455.8326054	0.295379595	0.106350822	2.777407722	0.00547944	0.028238307
Csad	230.9882611	-0.56707235	0.127560782	-4.44550699	8.77E-06	0.000103811
Cse1l	313.8214397	0.380416398	0.121569032	3.129221253	0.001752703	0.01078426
Csf1	254.5815098	-1.284770312	0.180188982	-7.130126935	1.00E-12	3.48E-11
Csf1r	9143.017798	-0.190678953	0.08789577	-2.16937577	0.030054167	0.109418458
Csf2ra	2027.712846	-0.555266902	0.151313846	-3.669637082	0.000242895	0.001979971
Csf2rb	2514.859254	-0.749606486	0.147692454	-5.075455556	3.87E-07	5.94E-06
Csf2rb2	914.3208868	-0.985097061	0.166138446	-5.929374471	3.04E-09	6.60E-08
Csf3	1.89813765	-0.747351452	0.35207011	-2.122734736	0.033776087	
Csf3r	230.0934532	-1.101046435	0.16398161	-6.714450677	1.89E-11	5.52E-10

Csnk1e	575.8127273	-0.343001987	0.104667572	-3.277060714	0.001048938	0.00694372
Csrnp2	23.97509254	-0.731745342	0.268823692	-2.722026978	0.006488285	0.032402558
Csrp2	84.4696272	-0.459408858	0.180468966	-2.545639118	0.010907789	0.048878229
Cst3	8204.663375	-0.342814408	0.072747275	-4.712402071	2.45E-06	3.23E-05
Csta2	46.0752153	2.564817439	0.32830456	7.812311346	5.61E-15	2.62E-13
Cstb	6327.161161	-0.192944054	0.093593571	-2.061509705	0.039254439	0.133308748
Cstf2t	302.3019101	0.247152712	0.112064899	2.205442686	0.027423045	0.101622184
Ctdnep1	408.015121	-0.228679241	0.11642989	-1.964093939	0.049519199	0.157179722
Ctdp1	150.9479304	0.263908666	0.132830904	1.986801706	0.046944376	0.15176524
Ctdsp2	816.2239632	-0.546198931	0.107969582	-5.058822331	4.22E-07	6.46E-06
Cthrc1	28.02104232	-1.121481813	0.29967616	-3.742312411	0.000182335	0.001532939
Ctla2a	305.154182	2.806280366	0.166012682	16.90401199	4.20E-64	4.51E-61
Ctla2b	1103.968187	2.256996895	0.14302843	15.78005785	4.27E-56	4.00E-53
Ctnnb1	5584.955662	-0.386803662	0.091374814	-4.233154044	2.30E-05	0.000245957
Ctnnbip1	546.9504374	0.490955493	0.119980226	4.091970059	4.28E-05	0.000427069
Ctnnd1	899.701036	0.37742205	0.139805859	2.699615398	0.006941968	0.034246584
Ctns	214.3305778	0.282103156	0.133768183	2.108895771	0.034953579	0.122500786
Ctsb	62270.27117	-0.195768767	0.069934916	-2.799299362	0.005121363	0.026676952
Ctsc	3945.272855	0.479110768	0.144128066	3.324201734	0.00088672	0.006015828
Ctsd	52493.91412	-0.371416789	0.103821419	-3.577458236	0.000346952	0.002685252
Ctsf	80.38933343	0.49819096	0.177803134	2.801924509	0.005079876	0.026534528
Ctsh	2716.211546	-0.559316376	0.070648817	-7.916854094	2.44E-15	1.21E-13
Ctsk	47.28640056	-0.76820846	0.229163554	-3.352227907	0.00080164	0.005508338
Ctsl	16354.20733	0.375475801	0.125780896	2.985157622	0.002834322	0.01619135
Ctss	56675.78709	-0.907413872	0.091499756	-9.917117927	3.51E-23	4.31E-21
Ctsz	3615.58456	-0.411818109	0.085875932	-4.795500908	1.62E-06	2.24E-05
Cttn	56.07811689	-0.619693795	0.209762946	-2.954257677	0.003134222	0.017696753
Ctxn1	8.730594595	-1.532366983	0.369437153	-4.147842117	3.36E-05	0.000342866
Cul4a	443.09028	0.260088469	0.102865566	2.528430829	0.011457366	0.050764893
Cuta	474.9137061	0.357922881	0.106376779	3.364671182	0.000766349	0.005302261
Cutc	102.3673854	-0.360794623	0.151617578	-2.379635834	0.017329755	0.07070898
Cux1	1026.941726	0.192504198	0.076061712	2.530894885	0.011377194	0.050529017
Cwc15	1410.761523	0.25946268	0.087064469	2.980121331	0.002881342	0.016391321
Cwf19l2	551.8680793	-0.254468377	0.100245919	-2.538441251	0.01113475	0.049687537
Cxcl1	100.8568342	1.21375486	0.193423541	6.275114477	3.49E-10	8.83E-09
Cxcl10	8.948319107	-0.816509229	0.378950195	-2.154661061	0.031188367	0.112510535
Cxcl12	103.2309675	-1.275217646	0.167484921	-7.613925115	2.66E-14	1.13E-12
Cxcl13	1229.049336	1.402653669	0.348776557	4.021639763	5.78E-05	0.000557046
Cxcl16	1512.220749	-0.925386029	0.104709749	-8.837630115	9.78E-19	7.45E-17
Cxcl2	239.4574971	1.594592835	0.190366394	8.376440849	5.46E-17	3.36E-15
Cxcl3	13.23025827	1.030701124	0.37012663	2.784725657	0.005357303	0.027713563
Cxcr4	857.7776723	-0.751478888	0.236671087	-3.175203605	0.001497314	0.00943333
Cyb561d1	70.12251147	-0.596719611	0.185707259	-3.21322718	0.001312524	0.008413948
Cyb5b	744.2347597	0.280150178	0.079963351	3.503482204	0.000459217	0.003441503

Cyb5r1	1067.551357	0.288072328	0.107851688	2.671004352	0.007562467	0.036680652
Cyb5r4	358.7424305	-0.221568113	0.098114561	-2.258259235	0.023929502	0.091606585
Cyba	5034.409513	-0.402185943	0.086172337	-4.66722799	3.05E-06	3.97E-05
Cybb	25761.3548	-0.798631951	0.073085256	-10.9274017	8.53E-28	1.54E-25
Cyfip2	138.3232036	-0.294333708	0.148308165	-1.984608929	0.047187998	0.152290385
Cyhr1	279.1628943	-0.241645945	0.110881852	-2.179310142	0.029308636	0.107276757
Cyld	646.4962327	-0.192150492	0.094425156	-2.034950223	0.041855882	0.13946075
Cyp11a1	25.2491668	-1.165844884	0.337609697	-3.453232813	0.000553911	0.004023494
Cyp1a1	3.619094884	-1.486624341	0.369516225	-4.023163907	5.74E-05	0.00055452
Cyp1b1	346.2611258	-1.607025824	0.175264102	-9.169167017	4.77E-20	4.09E-18
Cyp20a1	221.1025604	0.24671693	0.119931096	2.057155644	0.039671257	0.134207969
Cyp27a1	281.4013023	-0.828461657	0.150179872	-5.516462674	3.46E-08	6.38E-07
Cyp4f13	108.0829539	-0.541882982	0.180151184	-3.00793461	0.002630297	0.015240491
Cyp4v3	1057.394657	-0.81248408	0.078225543	-10.38642943	2.86E-25	4.52E-23
Cyren	279.2290529	0.813141364	0.135925954	5.982237695	2.20E-09	4.90E-08
Cysltr1	675.0655797	1.062611124	0.161265891	6.589187053	4.42E-11	1.25E-09
Cystm1	94.71134905	1.06405491	0.210842156	5.046689576	4.50E-07	6.84E-06
Cyth3	464.9589073	-0.419498188	0.090584512	-4.631014483	3.64E-06	4.65E-05
Cyth4	1504.998277	-0.314373063	0.065945445	-4.767168731	1.87E-06	2.54E-05
D10Wsu102e	237.9410294	-0.460484553	0.116889499	-3.939486069	8.17E-05	0.000757833
D130058E05Rik	24.94082431	2.591576738	0.338013909	7.667071299	1.76E-14	7.72E-13
D530018E20Rik	46.36798594	-0.662878199	0.225324957	-2.941876516	0.0032623	0.018281305
D5Ertd605e	7.25445675	1.29784823	0.379151885	3.423029876	0.000619273	0.004423334
D6Wsu163e	226.5453747	0.734798825	0.141122456	5.20681715	1.92E-07	3.10E-06
D730003I15Rik	50.4574382	-0.552068215	0.229789414	-2.402496297	0.016283599	0.067222115
D830025C05Rik	6.229503105	-0.75245289	0.375942955	-2.001508154	0.045337656	0.147877027
D830050J10Rik	146.2799558	-0.630989011	0.156969535	-4.019818319	5.82E-05	0.00056065
D930019O06Rik	10.99106844	-0.940581813	0.342536698	-2.745930053	0.006033963	0.030602122
D930048N14Rik	12.05192238	-1.322636977	0.354623829	-3.729690081	0.000191715	0.001601934
Daam1	247.747677	-0.416903469	0.110918757	-3.758638143	0.000170841	0.001448478
Dab2	5754.62703	2.662216181	0.101941466	26.11514519	2.45E-150	3.68E-146
Dab2ip	176.6894489	1.090985209	0.129861706	8.401131062	4.42E-17	2.74E-15
Dag1	924.2228354	-0.223313651	0.093712818	-2.382957365	0.017174183	0.070188717
Daglb	1199.60574	-1.66161747	0.113292519	-14.66661246	1.05E-48	6.50E-46
Dalrd3	188.3494042	-0.430481819	0.11947723	-3.603044863	0.000314511	0.002478736
Dapk1	3110.581483	-0.355051276	0.092294408	-3.84694244	0.000119601	0.001061415
Dars	353.1642943	0.256075254	0.101208644	2.530171773	0.01140067	0.050603328
Daxx	354.2829001	-0.303459516	0.108110444	-2.806939882	0.005001457	0.02618872
Dazap1	546.409658	0.268983612	0.100436898	2.678135408	0.007403328	0.03602521
Dbf4	338.8552101	0.865173418	0.124244797	6.963457954	3.32E-12	1.10E-10
Dbn1	34.88747125	-0.790979448	0.269309256	-2.937067443	0.00331332	0.018532612
Dbnl	730.102088	0.239420739	0.085267396	2.807881432	0.004986858	0.026123414
Dbp	203.5207174	0.497019002	0.195506496	2.542212213	0.011015327	0.049257156
Dcaf11	271.8651858	-0.312149825	0.110220511	-2.832048432	0.004625084	0.024534691

Dcaf17	332.4496771	-0.232456876	0.099067167	-2.346457285	0.018952838	0.075907459
Dcaf5	315.8068371	0.228566495	0.099495023	2.297265615	0.021603622	0.08442853
Dcaf6	232.8378894	-0.233387217	0.11833872	-1.972196561	0.048587172	0.154907094
Dcaf8	688.1117884	-0.17672788	0.084812859	-2.083739218	0.037183895	0.127975542
Dcbld2	225.4641436	-0.697495747	0.117430256	-5.939659603	2.86E-09	6.23E-08
Dchs1	29.0161972	-0.877445268	0.281822432	-3.113468509	0.001849022	0.011261477
Dck	279.2645261	1.153359263	0.129670345	8.894549174	5.87E-19	4.61E-17
Dclk1	142.512885	-1.418571514	0.199112383	-7.124476618	1.04E-12	3.61E-11
Dcn	365.8474258	-1.115999589	0.182248859	-6.123492882	9.15E-10	2.17E-08
Dcps	158.9928716	0.518130201	0.130719491	3.963679759	7.38E-05	0.000690502
Dcstamp	134.342856	-0.888014763	0.206585258	-4.298538873	1.72E-05	0.000190979
Dctn2	673.2395322	-0.317865162	0.084993665	-3.739868892	0.000184116	0.001546288
Dctn5	407.5936245	0.323012375	0.111933107	2.885762615	0.003904665	0.021300369
Dctn6	796.8288717	0.263804793	0.091660069	2.878077609	0.004001067	0.021707886
Dctpp1	165.762426	0.556517398	0.16619638	3.348553066	0.000812347	0.005571707
Dcxr	219.0330073	0.75250965	0.114524451	6.570733507	5.01E-11	1.40E-09
Ddah2	108.8637102	0.783615147	0.185488762	4.22459635	2.39E-05	0.000253688
Ddhd1	470.4862808	-0.927113194	0.16886041	-5.490411822	4.01E-08	7.30E-07
Ddhd2	343.4646633	0.233418276	0.111708848	2.089523612	0.036660615	0.126737122
Ddi2	1536.097791	-0.288140048	0.082539987	-3.490914624	0.00048137	0.003569131
Ddias	28.89614286	0.9608665	0.266947043	3.599464858	0.000318873	0.002505405
Ddit3	182.560763	-0.633506342	0.145832065	-4.344081275	1.40E-05	0.000158406
Ddit4l	14.26921692	-2.172557859	0.347919979	-6.244418222	4.25E-10	1.06E-08
Ddr1	134.9175647	-1.144764964	0.189152626	-6.052070162	1.43E-09	3.30E-08
Ddr2	69.48558923	-0.745517712	0.188805016	-3.948611782	7.86E-05	0.000732238
Ddrgk1	524.8532709	-0.226313998	0.10044569	-2.253098144	0.024252961	0.092611754
Ddx11	20.31059054	0.817026727	0.30624817	2.667858319	0.007633644	0.036954225
Ddx17	1620.419465	-0.260457091	0.067377357	-3.865647205	0.000110795	0.000991473
Ddx21	1449.617714	0.191175212	0.09726696	1.965469183	0.049359959	0.156820155
Ddx39	319.5565645	0.434880424	0.119679239	3.633716471	0.000279368	0.002241964
Ddx58	253.0555156	0.507879906	0.171592494	2.959802587	0.003078362	0.017432824
Def6	86.70243782	0.470217802	0.179723088	2.616346104	0.008887642	0.041493019
Defb42	9.020859191	-0.743647793	0.375412477	-1.980881931	0.047604516	0.153239161
Dennd1b	831.5944728	-0.516391335	0.119895231	-4.307021478	1.65E-05	0.000184212
Dennd2c	58.48193072	1.238767766	0.210719555	5.878750856	4.13E-09	8.80E-08
Dennd3	27.16700799	-1.68706158	0.280098114	-6.023109397	1.71E-09	3.89E-08
Dennd4a	518.5634004	0.854970889	0.140587354	6.081420999	1.19E-09	2.79E-08
Dennd4b	382.7453816	-0.428885703	0.094050299	-4.560173736	5.11E-06	6.35E-05
Denr	1123.702834	0.278102521	0.101448573	2.741315242	0.006119377	0.030993413
Depdc1b	2.760200723	1.14000504	0.356478189	3.197965754	0.001384007	0.008793309
Dera	298.8467013	0.462812485	0.135498076	3.41563881	0.000636326	0.004515056
Derl1	1610.498442	0.291635569	0.082102246	3.552102201	0.000382166	0.002926108
Des	38.81233284	-2.009559626	0.268781492	-7.476555079	7.63E-14	3.04E-12
Desi2	407.5483327	0.203946416	0.097878053	2.083678724	0.037189401	0.127975542

Dgkd	722.8148979	0.277364798	0.105482392	2.629489094	0.008551327	0.040329909
Dgkg	86.82943967	-1.230658112	0.218302098	-5.637408545	1.73E-08	3.33E-07
Dguok	384.5688195	-0.427451451	0.114355194	-3.737927718	0.000185543	0.001554689
Dhcr24	155.7903025	0.506747848	0.161605066	3.135717593	0.001714342	0.01057859
Dhdh	100.1400271	-0.388646154	0.177914349	-2.184456483	0.028928722	0.106093188
Dhfr	124.188384	1.956621175	0.202283228	9.67268119	3.94E-22	4.28E-20
Dhps	146.0678729	-0.287093598	0.137430586	-2.089008028	0.036707	0.126839041
Dhrs1	908.1356587	0.479890969	0.078814009	6.088904413	1.14E-09	2.68E-08
Dhrs3	2107.585933	-0.746490793	0.086565319	-8.623439488	6.50E-18	4.51E-16
Dhrs7	719.4044586	-0.288186257	0.098341007	-2.930479009	0.003384399	0.018840995
Dhx29	191.8138928	0.362449149	0.130454718	2.778352174	0.005463537	0.028175703
Dhx34	79.25079911	-0.927256803	0.19130937	-4.846896954	1.25E-06	1.77E-05
Dhx9	892.9403184	0.312505961	0.074929068	4.170690591	3.04E-05	0.000314513
Diaph1	2134.021636	-0.310902535	0.096094955	-3.235367936	0.001214861	0.007895805
Diaph2	1369.121897	0.395646254	0.089476388	4.421795082	9.79E-06	0.000114851
Diaph3	126.6508262	1.039660137	0.198197978	5.245563792	1.56E-07	2.57E-06
Dip2a	190.0619374	0.433705611	0.131068204	3.309007044	0.000936275	0.006300752
Dis3l	65.97396081	0.433197968	0.20397345	2.123795855	0.033687218	0.119372864
Disp1	68.5813851	-0.582184317	0.194104821	-2.999329512	0.002705745	0.015586363
Dkk2	295.3590789	-1.446502467	0.317808338	-4.551493127	5.33E-06	6.60E-05
Dlc1	64.30040615	-1.274173813	0.196157731	-6.495659429	8.27E-11	2.26E-09
Dleu2	524.7530348	-0.605765942	0.130178155	-4.653360925	3.27E-06	4.22E-05
Dlg3	130.7633671	-1.254776822	0.194534464	-6.450151806	1.12E-10	2.99E-09
Dlgap4	558.8343558	-0.389097698	0.093067226	-4.180824048	2.90E-05	0.000302698
Dlgap5	35.10467207	2.119478869	0.30719189	6.899527431	5.22E-12	1.68E-10
Dlk1	40.17226235	0.667213707	0.270900284	2.46294945	0.013779934	0.058849025
Dlst	509.7687369	0.218068351	0.101765743	2.142846364	0.032125437	0.115074388
Dlx2	6.740255721	-0.972338399	0.373479807	-2.603456412	0.0092289	0.042641042
Dmac1	461.2754356	0.50662396	0.119873265	4.226329862	2.38E-05	0.000252277
Dmc1	1.665541157	0.764147973	0.344227097	2.219894891	0.026425903	
Dmd	8.957322609	-1.58384081	0.366747775	-4.318610545	1.57E-05	0.000175845
Dmpk	489.265791	2.413809187	0.132494896	18.21812965	3.71E-74	5.06E-71
Dmwd	136.1241192	1.256939189	0.184172459	6.824794505	8.81E-12	2.73E-10
Dmxl1	744.2869912	0.22784903	0.114791398	1.984896374	0.047156002	0.152219858
Dmxl2	473.5027816	-0.543589746	0.130651139	-4.160620041	3.17E-05	0.00032668
Dnaaf3	55.26834932	-0.577588709	0.21997036	-2.625756986	0.008645652	0.040619448
Dnaic1	2.445477543	0.904249634	0.367797975	2.458549791	0.013949943	0.059406015
Dnajb11	557.5614331	0.357906591	0.10483697	3.41393489	0.000640319	0.00454124
Dnajb12	238.2889393	-0.387870721	0.116434065	-3.331247795	0.000864576	0.005884214
Dnajb14	1832.51758	-0.513398591	0.094339398	-5.442038039	5.27E-08	9.39E-07
Dnajb2	74.3868832	-0.492803111	0.191769689	-2.569765401	0.010176741	0.046059276
Dnajb4	362.2068418	-0.339666858	0.109267542	-3.10857966	0.00187989	0.011393683
Dnajb5	34.80295578	0.695407401	0.24317658	2.859680811	0.004240676	0.022842722
Dnajc10	688.1808457	0.222098498	0.086451441	2.5690549	0.01019763	0.046122916

Dnajc19	363.5173442	0.262104983	0.106645151	2.457729969	0.013981826	0.059440585
Dnajc28	63.0921404	-0.542742488	0.191276579	-2.837474884	0.004547192	0.024189903
Dnajc3	1853.620983	0.190952146	0.074102818	2.576854042	0.009970404	0.045299987
Dnajc8	1878.218267	0.182319419	0.072914035	2.500470844	0.012402834	0.054201902
Dnajc9	172.2600295	0.874189297	0.181287924	4.822104417	1.42E-06	1.98E-05
Dnase1l1	926.3985136	-0.561326967	0.098772799	-5.683011652	1.32E-08	2.62E-07
Dner	2.814272192	0.826528981	0.363791711	2.271984093	0.023087471	0.08912617
Dnmt1	422.4785473	0.402091535	0.128812261	3.121531539	0.00179913	0.011011232
Dnmt3a	1179.769406	-1.065396866	0.125089	-8.517110727	1.64E-17	1.08E-15
Dnmt3aos	43.33061985	-1.525749881	0.24082164	-6.335601237	2.36E-10	6.13E-09
Dnph1	41.98351299	1.145810389	0.281285132	4.073483658	4.63E-05	0.000457597
Dock4	72.25168402	0.576107125	0.224022655	2.571646712	0.010121612	0.045875878
Dock6	85.54799554	0.726873329	0.215302598	3.376054615	0.000735333	0.005118342
Dock7	429.0712727	0.352139435	0.103693324	3.395970171	0.000683858	0.004801257
Dok1	490.828764	-0.316092927	0.095449564	-3.311622534	0.000927566	0.006256173
Dok2	678.6007842	0.350806015	0.101807768	3.44576865	0.000569438	0.0041243
Dok3	329.1300341	0.448215578	0.102670534	4.365571689	1.27E-05	0.000145693
Dok7	10.20497204	-0.943081676	0.37639883	-2.505538279	0.012226516	0.053617559
Donson	85.07773283	0.460861392	0.181507323	2.539078773	0.01111448	0.049641367
Dop1b	216.436159	-0.492606881	0.121504919	-4.054213492	5.03E-05	0.000491472
Dot1l	607.4492402	-0.482829504	0.148839369	-3.243963654	0.001178788	0.007684652
Dpep2	1392.571353	-0.711333866	0.120824575	-5.887327673	3.92E-09	8.41E-08
Dph6	228.8258784	0.28981036	0.134858407	2.148997353	0.03163461	0.113683091
Dph7	19.4059106	0.590712785	0.291695603	2.025100067	0.04285709	0.141789319
Dppa2	2.196971192	0.900936295	0.336919477	2.674040403	0.007494342	0.036385504
Dppa4	2.345080589	1.128306555	0.357620098	3.155042349	0.001604748	0.010026001
Dpy19l1	909.6606879	0.217042985	0.095209333	2.27963981	0.02262906	0.087637239
Dpy30	262.5728077	0.573394688	0.126189022	4.54393479	5.52E-06	6.83E-05
Dpysl2	834.3506927	0.310901863	0.0967855	3.21227727	0.001316872	0.008430796
Dpysl3	148.0911456	-0.607211838	0.173239284	-3.505047038	0.000456527	0.003423836
Dr1	522.4313826	0.261383012	0.09582537	2.727701561	0.006377728	0.031956783
Dram1	544.9777516	-0.654028867	0.095007138	-6.883997151	5.82E-12	1.85E-10
Dram2	953.5233685	-0.19317674	0.0743103	-2.599595754	0.009333364	0.043017748
Dscc1	27.97961401	1.14307269	0.296413334	3.856347056	0.000115094	0.001024446
Dse	700.4312323	0.3860949	0.106105484	3.638783657	0.000273929	0.002204209
Dsn1	26.95696832	1.248439175	0.301638639	4.138856948	3.49E-05	0.000355363
Dtl	65.29845674	2.164729812	0.243657815	8.884302798	6.43E-19	4.98E-17
Dtna	22.09752062	-1.014293596	0.30311824	-3.346197827	0.000819279	0.00561412
Dtnbp1	760.4527102	-0.397701221	0.095650685	-4.157850208	3.21E-05	0.000330212
Dtx4	417.5510203	-0.691518653	0.114771152	-6.025195707	1.69E-09	3.85E-08
Dtymk	145.3207475	0.623092761	0.173206093	3.597406709	0.000321406	0.002521346
Dubr	255.6197113	-0.375410006	0.12849805	-2.92152297	0.003483246	0.019296076
Dus3l	75.01937832	-0.469005927	0.199290239	-2.353381334	0.018603545	0.074787944
Dusp19	47.76407284	0.496365084	0.224558869	2.210400716	0.027077364	0.100706321

Dusp2	38.96102513	-0.79298737	0.25301476	-3.134154583	0.0017235	0.010626364
Dusp23	291.508283	0.245376665	0.124466536	1.971426803	0.048675078	0.155121447
Dusp28	46.40555604	-0.585117797	0.240374898	-2.434188438	0.014925223	0.062722716
Dusp6	1606.34603	1.861750297	0.098129632	18.97235588	2.89E-80	4.81E-77
Dusp7	1073.140097	0.893553307	0.071292527	12.53361802	4.89E-36	1.67E-33
Dut	248.9976917	0.892414012	0.162149221	5.503658942	3.72E-08	6.80E-07
Dync2li1	26.11800145	0.543069413	0.276499595	1.964087556	0.049519939	0.157179722
Dynll2	763.349951	0.308226957	0.103164618	2.987719643	0.002810673	0.01606504
Dynlt1a	7.350202432	1.01171508	0.375585385	2.693701941	0.007066332	0.034745888
Dysf	1.808787239	-0.750160036	0.335066465	-2.238839497	0.025166361	
Dzip1l	8.041264714	-1.193208183	0.369429983	-3.229862861	0.001238496	0.008021627
E130102H24Rik	37.3464593	-1.159662642	0.237590133	-4.880937722	1.06E-06	1.52E-05
E230020D15Rik	37.03106037	-0.626724814	0.264066248	-2.373362058	0.017626976	0.071590806
E230029C05Rik	53.77533968	-0.791042705	0.27300736	-2.897514208	0.003761327	0.020615864
E230032D23Rik	81.72813701	-0.449329074	0.191642453	-2.344621807	0.019046388	0.076189581
E2f1	157.0641226	0.821989463	0.15514296	5.298271122	1.17E-07	1.97E-06
E2f2	68.39714574	1.934552274	0.249566208	7.751659529	9.07E-15	4.12E-13
E2f4	235.135696	0.255498852	0.129498862	1.972981445	0.048497676	0.154733765
E2f7	34.77412208	1.639245441	0.286986571	5.711923854	1.12E-08	2.24E-07
E2f8	50.35099957	1.70733047	0.236015404	7.233978957	4.69E-13	1.70E-11
E330009J07Rik	59.28090855	-0.556247442	0.206098497	-2.698939834	0.006956075	0.034293633
E330013P04Rik	1.640793154	-0.705336311	0.331314741	-2.128901082	0.033262445	
E330034L11Rik	26.12409009	-0.799262925	0.259998454	-3.074106455	0.002111341	0.012613412
E430024P14Rik	6.158690565	1.093858777	0.377331545	2.898932756	0.003744352	0.020537825
Eaf1	295.1031847	0.431055743	0.152172388	2.832680417	0.004615951	0.024494899
Ear2	8.43763515	-0.953221059	0.374815218	-2.54317598	0.010984989	0.049165442
Ebf1	117.2286307	0.653548329	0.319872586	2.043152051	0.041037389	0.137435414
Ebi3	427.1340904	0.516492097	0.114204165	4.522532927	6.11E-06	7.46E-05
Ebpl	146.422363	0.292016999	0.135627928	2.153074255	0.031312843	0.112870707
Ece1	126.0623888	1.215786744	0.156802742	7.753606413	8.93E-15	4.07E-13
Ece2	81.41437891	0.40895295	0.196044323	2.086022914	0.036976547	0.12753552
Echdc2	6.225944484	-0.737883532	0.375007697	-1.967649031	0.049108434	0.156237072
Echdc3	8.72336837	-0.821586415	0.368047448	-2.232283962	0.025596201	0.096513113
Echs1	805.5764452	-0.402606321	0.082296949	-4.892117207	9.98E-07	1.44E-05
Ecpas	567.8849577	-0.222015422	0.105022208	-2.113985466	0.034516509	0.121451171
Ecscr	5.591800392	-1.030577781	0.379431856	-2.716107689	0.006605443	0.032867334
Ect2	91.50294579	1.42174675	0.245902099	5.78175932	7.39E-09	1.52E-07
Eda2r	28.44119396	-1.230492635	0.25937664	-4.744038003	2.09E-06	2.82E-05
Edem1	1114.226839	0.381325653	0.085359244	4.467303529	7.92E-06	9.46E-05
Edem2	350.2104218	-0.665766858	0.121320311	-5.487678469	4.07E-08	7.40E-07
Edem3	407.0014792	0.232612254	0.094548959	2.460230717	0.013884772	0.059195676
Edil3	225.6346218	1.667679157	0.207219087	8.047903216	8.42E-16	4.42E-14
Ednrb	2641.88095	0.698486837	0.126519838	5.520769313	3.38E-08	6.25E-07
Eea1	2872.839813	0.552474595	0.113399001	4.87195294	1.11E-06	1.58E-05

Eef2	5502.42368	-0.151922904	0.060148182	-2.52581041	0.011543176	0.051067224
Eepd1	591.934101	-0.871774924	0.112611642	-7.741428079	9.83E-15	4.43E-13
Efcab11	8.470008082	1.579662492	0.377619725	4.183209691	2.87E-05	0.000300163
Efemp1	8.059144866	0.836009066	0.376582453	2.219989433	0.026419485	0.098896785
Efna5	42.48029179	-0.645298475	0.224537477	-2.873900986	0.00405436	0.021973199
Efnb1	64.15222854	-0.505685624	0.204922179	-2.467695914	0.013598578	0.058256885
Efr3b	59.56898306	-1.39959986	0.258293815	-5.41863482	6.01E-08	1.05E-06
Egfl7	11.31064546	1.763148394	0.363036371	4.856671489	1.19E-06	1.70E-05
Egln1	446.6229984	0.461684629	0.102719454	4.494617259	6.97E-06	8.41E-05
Egln3	79.34979769	0.84848684	0.242998982	3.491730026	0.000479903	0.003560012
Egr1	150.5975417	-1.439208718	0.155001901	-9.285103656	1.62E-20	1.45E-18
Egr2	638.4311419	-1.762629958	0.178112662	-9.89615188	4.33E-23	5.28E-21
Ehd1	1400.845207	1.206429355	0.114062076	10.57695417	3.81E-26	6.43E-24
Eid1	1738.1989	0.436823215	0.086800471	5.032498228	4.84E-07	7.32E-06
Eif1b	829.4631053	0.174521088	0.086469295	2.018301276	0.043559894	0.143481854
Eif2a	435.6738197	-0.191097615	0.095803999	-1.994672633	0.046078596	0.14981134
Eif2ak2	215.9458155	0.825016776	0.157362389	5.242782489	1.58E-07	2.60E-06
Eif2ak4	200.1420337	-0.34018932	0.122999963	-2.765767658	0.005678898	0.029106292
Eif2b3	94.23587713	0.374174398	0.1559906	2.398698367	0.016453462	0.067759908
Eif2b5	257.1634657	0.258216724	0.126270832	2.044943545	0.040860424	0.137179505
Eif2s1	328.4471903	0.241899719	0.101151177	2.391467158	0.016781185	0.06886389
Eif2s2	1760.929463	0.25831427	0.084028839	3.074114475	0.002111284	0.012613412
Eif3a	4711.74355	0.197103802	0.095187452	2.070691031	0.038387679	0.130928159
Eif3f	1230.532852	-0.3884113	0.0871333	-4.457667729	8.29E-06	9.85E-05
Eif3k	1157.025903	-0.347449142	0.080416168	-4.320637863	1.56E-05	0.000174497
Eif4b	724.5377126	-0.175805303	0.085574832	-2.054404303	0.039936577	0.134831993
Eif4e	634.6079703	0.622903399	0.110357209	5.644428711	1.66E-08	3.21E-07
Eif4ebp1	312.8670114	0.291572174	0.11923289	2.44540055	0.014469135	0.061079692
Eif4ebp2	326.0620395	0.354970085	0.152399452	2.329208405	0.019848028	0.078924047
Eif4g1	3346.754381	0.222881436	0.085449604	2.608337845	0.009098311	0.04220408
Eif4g2	3789.209164	0.32360267	0.079472034	4.07190622	4.66E-05	0.000460379
Eif5	2019.901391	0.23908736	0.064936285	3.681876146	0.000231524	0.00189966
Elavl1	397.4594875	0.253053348	0.092187702	2.744979454	0.006051469	0.030670175
Eldr	6.158828806	1.439318764	0.380152135	3.78616515	0.00015299	0.001312705
Elf4	387.2041316	-0.224239027	0.093422783	-2.40026063	0.016383402	0.067545526
Elk1	172.3309253	0.253051854	0.124439671	2.033530402	0.041998965	0.139720344
Elk3	359.7578432	0.758412172	0.115249446	6.58061445	4.69E-11	1.32E-09
Elk4	526.739804	-0.359160637	0.087836137	-4.088984868	4.33E-05	0.00043183
Ell2	613.1485678	0.286806709	0.13168941	2.177902608	0.029413288	0.107607318
Elmo1	400.1900153	0.708056292	0.106433439	6.652573636	2.88E-11	8.33E-10
Eloc	693.0759254	0.409275606	0.115053891	3.557251317	0.000374756	0.002879193
Elovl5	1285.329186	-0.323329278	0.092629094	-3.490580153	0.000481973	0.003571837
Elovl6	71.28491836	0.376647608	0.190915083	1.97285412	0.048512185	0.154733765
Emc2	1107.606534	0.413638495	0.070884375	5.835397354	5.37E-09	1.13E-07

Emc6	473.7502419	0.229599997	0.112304525	2.044441186	0.040909982	0.137253767
Eme1	19.76718063	1.21972464	0.33654702	3.624232476	0.000289821	0.002313576
Emilin1	183.8306958	0.36217081	0.157336929	2.301880501	0.021341912	0.083536274
Emilin2	2624.918425	0.888375741	0.090198316	9.849138911	6.91E-23	8.17E-21
Eml1	73.42517335	-0.558283525	0.21473495	-2.599872651	0.009325836	0.042996259
Eml4	401.2690414	-0.375635731	0.125150683	-3.001467686	0.002686816	0.01550213
Emp1	1788.972834	0.978897945	0.144114932	6.792481051	1.10E-11	3.34E-10
Emp3	2644.629313	0.268355985	0.089319135	3.004462409	0.002660506	0.015383713
Emx2	7.464530995	-1.240134481	0.368853257	-3.362135097	0.000773423	0.005341351
Enah	130.0644768	-0.80599201	0.153519396	-5.250098899	1.52E-07	2.52E-06
Enc1	598.3534708	-1.046875346	0.108877065	-9.615205445	6.90E-22	7.19E-20
Endov	22.7202488	-0.657254013	0.307460575	-2.137685498	0.032542277	0.116055598
Eng	166.5604839	-0.596516209	0.167637057	-3.558379142	0.00037315	0.002868785
Eno1	219.8001832	0.438250462	0.139836242	3.134026307	0.001724254	0.010626644
Eno2	16.58248829	-1.209615821	0.35267791	-3.429803191	0.000604019	0.004331298
Eno3	52.16743585	0.564034131	0.211751897	2.663655624	0.007729665	0.037334754
Enpp1	316.6322579	0.261325248	0.124746335	2.094853106	0.036184053	0.125581424
Enpp2	72.26929381	-1.747592858	0.237068319	-7.371684519	1.68E-13	6.43E-12
Enpp3	16.32948689	-0.912795785	0.326748367	-2.793574127	0.005212908	0.027096449
Enpp4	238.8318271	-0.746120583	0.193667616	-3.852583094	0.000116878	0.001039095
Ensa	291.0614864	-0.239953223	0.111547968	-2.151121412	0.031466618	0.113296433
Entpd1	1183.883119	-0.282460742	0.111340589	-2.536907212	0.011183659	0.049876129
Entpd5	100.8435938	0.391908986	0.166870982	2.348574821	0.018845413	0.07559472
Entpd7	285.862414	0.411239364	0.134067852	3.067397271	0.002159317	0.012864178
Epas1	836.5392302	-0.553051546	0.171683403	-3.221345437	0.001275903	0.008221327
Epb41l1	365.7768009	-0.484097785	0.10732087	-4.510751607	6.46E-06	7.84E-05
Epb41l4a	4.362867626	-0.845058461	0.379714148	-2.225512182	0.026046874	0.097888885
Epb41l4aos	248.4965184	-0.423337864	0.154956719	-2.731974883	0.006295594	0.031640315
Epg5	123.1741079	0.302332433	0.139590497	2.165852539	0.030322457	0.110048153
Epha2	13.53597562	0.79462236	0.335804922	2.366321363	0.017965842	0.072691667
Epha4	128.9713486	-1.164089651	0.169850886	-6.853597756	7.20E-12	2.26E-10
Ephx1	523.6501492	-1.202817759	0.09778139	-12.30109077	8.94E-35	2.79E-32
Ephx2	5.04719415	0.955668069	0.381208588	2.50694265	0.012178047	0.053453042
Epm2a	53.20561957	0.411241735	0.19797158	2.077276625	0.037776035	0.129459913
Epn1	609.5398825	0.206520023	0.093823778	2.201148031	0.027725546	0.102431627
Epn2	197.7019059	0.791331403	0.130739599	6.052729301	1.42E-09	3.29E-08
Epop	13.41320208	0.945586985	0.337396054	2.802602387	0.005069212	0.026497272
Epor	9.985545849	1.320774374	0.36602906	3.608386651	0.000308107	0.002436445
Eprs	1507.101198	-0.286921124	0.093603422	-3.065284565	0.00217463	0.012945129
Eps15	850.1558196	0.677821445	0.101820136	6.65704713	2.79E-11	8.11E-10
Eps15l1	149.1576965	-0.465608829	0.14199634	-3.27901993	0.001041683	0.006901781
Eps8	485.6064984	0.530220697	0.183510368	2.889322843	0.003860725	0.02108366
Eps8l1	50.63801762	-1.106992533	0.283678013	-3.902285278	9.53E-05	0.000866141
Epsti1	658.1040164	0.295143126	0.093705476	3.149689193	0.001634442	0.01018185

Erbb3	23.52245227	0.775794104	0.293411878	2.644044639	0.008192185	0.03907248
Ercc6	90.58635113	-0.490856215	0.195433663	-2.511625715	0.012017647	0.05285722
Ercc6l	44.1404447	1.46799647	0.266025377	5.518257261	3.42E-08	6.33E-07
Erf	250.6877393	-0.359398344	0.126473776	-2.841682711	0.004487613	0.023968751
Erg28	316.8878711	0.230363542	0.117178213	1.965924686	0.049307311	0.156770088
Ergic1	854.0863335	0.184499983	0.086128359	2.142151391	0.032181302	0.115206296
Ergic3	696.371816	-0.237317819	0.088130974	-2.6927856	0.007085782	0.034807309
Eri1	571.8993162	0.384469853	0.107209235	3.586163572	0.000335579	0.002612047
Eri2	50.18494251	0.718811476	0.215418954	3.336807016	0.000847468	0.005783514
Erlec1	318.6090199	-0.320314858	0.10598454	-3.022279084	0.002508791	0.014615462
Erlin1	250.380929	0.425270983	0.118139229	3.599744016	0.000318531	0.002504028
Ermp1	163.6580935	0.382392113	0.142838948	2.677085747	0.007426563	0.03610315
Ero1l	195.8858589	0.539344242	0.133819009	4.030400798	5.57E-05	0.000540853
Esco1	222.2119032	-0.306167539	0.121766191	-2.514388736	0.011923892	0.052506411
Esco2	32.3630788	2.194517056	0.299575875	7.325413168	2.38E-13	8.96E-12
Esd	3335.940736	-0.484770388	0.146584392	-3.307107822	0.000942646	0.006337943
Espl1	93.86560959	1.062693187	0.199767433	5.319651801	1.04E-07	1.77E-06
Espn	30.56235695	-0.765050466	0.251757227	-3.038842121	0.002374893	0.013916445
Esyt1	2410.849256	-0.494776112	0.111299826	-4.445434742	8.77E-06	0.000103811
Etfrf1	163.7102107	-0.283270858	0.134946654	-2.099132139	0.035805254	0.124728284
Ethe1	260.1065695	0.328381576	0.117095996	2.804379205	0.005041357	0.026360854
Ets2	658.4573928	1.340656079	0.112230975	11.9455086	6.85E-33	1.90E-30
Etv1	174.8155692	2.273043358	0.181326647	12.53562777	4.77E-36	1.66E-33
Etv3	486.9417505	-0.374976797	0.110254872	-3.400999784	0.000671399	0.004728147
Etv4	19.49178527	-0.65222028	0.326873865	-1.995327101	0.046007216	0.149703012
Etv5	225.5944977	1.347896527	0.131586402	10.24343322	1.27E-24	1.86E-22
Etv6	573.0340537	0.242495697	0.094534751	2.565148743	0.010313158	0.046547238
Eva1b	78.9345766	0.523548375	0.230282047	2.273509303	0.022995506	0.088850041
Evc2	9.687318939	-0.753142082	0.345954565	-2.176997091	0.029480785	0.107775428
Evi5	1194.273609	0.511546612	0.093844159	5.451022378	5.01E-08	8.97E-07
Evi5l	36.36330801	0.470535809	0.22944121	2.050790301	0.040287372	0.135771974
Evl	865.8661397	-1.06397271	0.079133356	-13.44531253	3.28E-41	1.59E-38
Exo1	36.52048087	1.147333056	0.300122047	3.822888284	0.000131898	0.001158213
Exoc3l2	4.148535325	0.780265229	0.380946134	2.048229811	0.040537487	0.13643664
Exoc3l4	30.21585559	-1.332523306	0.264622452	-5.03556405	4.76E-07	7.23E-06
Exoc5	1286.159251	0.212569218	0.086957743	2.444511659	0.014504839	0.061195986
Extl1	2.648095185	-0.760227577	0.364566113	-2.085294133	0.03704261	0.127674524
Extl2	177.0504324	0.290797948	0.125457443	2.317901132	0.020454695	0.080758644
Extl3	170.029489	-0.52966133	0.132487516	-3.997820669	6.39E-05	0.000608353
Eya3	360.8303082	-0.200505815	0.099452408	-2.01609815	0.043789714	0.143954486
Ezh1	370.3974068	-0.549424409	0.102601866	-5.354916346	8.56E-08	1.47E-06
Ezh2	373.1272066	0.981966773	0.130480843	7.52575436	5.24E-14	2.14E-12
Ezr	669.5532263	0.249993296	0.088142803	2.836230387	0.00456495	0.024267166
F10	1435.02504	-0.651630302	0.222200159	-2.932627524	0.003361069	0.018750764

F11r	53.34217188	-0.558147404	0.215039287	-2.595560143	0.009443687	0.043406253
F13a1	4576.192039	3.202120089	0.144760637	22.12010228	2.02E-108	5.06E-105
F2r	59.9145717	-0.610942967	0.275606907	-2.216718631	0.026642325	0.099597347
F3	40.69900927	-0.562075977	0.275945511	-2.036909299	0.041659132	0.138928858
F630028O10Rik	1028.357884	0.554548515	0.158710882	3.494079988	0.000475698	0.003535813
F7	941.8137775	-0.789242444	0.128692238	-6.132789818	8.64E-10	2.05E-08
F830208F22Rik	20.24805767	-0.706254618	0.295834931	-2.387326665	0.016971404	0.069511424
Fabp4	4195.806964	-1.382685815	0.227945437	-6.065863096	1.31E-09	3.06E-08
Fabp5	3220.116662	-0.804233365	0.123669039	-6.503110016	7.87E-11	2.15E-09
Fads1	499.0976143	-0.199388285	0.093907871	-2.123232937	0.033734337	0.119483408
Fads3	20.38718965	-0.602611731	0.297383619	-2.026378362	0.042726027	0.141480468
Fads6	18.00111306	1.057164054	0.325012536	3.25268701	0.001143193	0.007474127
Faim	380.2531654	0.24283293	0.097204182	2.498173688	0.012483501	0.054522673
Fam102b	1107.405106	0.503296151	0.093682438	5.372363913	7.77E-08	1.34E-06
Fam107b	128.7697574	0.473962856	0.17075189	2.775740032	0.005507623	0.028354338
Fam110a	92.64583102	0.644533178	0.170646028	3.777018346	0.000158717	0.001355244
Fam111a	2471.609603	0.611116793	0.11210469	5.451304443	5.00E-08	8.97E-07
Fam114a2	620.089408	0.250021657	0.091176961	2.742158277	0.006103692	0.030924412
Fam122b	133.7433125	0.524269855	0.141291348	3.710558811	0.000206802	0.001717477
Fam124a	3.974474966	-1.006129814	0.377649139	-2.664191995	0.00771735	0.037287275
Fam129b	2748.565748	-0.50259185	0.139092092	-3.613374742	0.000302237	0.002393858
Fam13b	217.6077627	-0.461782536	0.148376489	-3.112235221	0.001856765	0.011289657
Fam162a	316.9559736	0.476291902	0.116609564	4.084501187	4.42E-05	0.000438415
Fam167b	31.8797771	1.527461094	0.274715106	5.560164184	2.70E-08	5.06E-07
Fam168a	511.9756831	-0.287973077	0.110113852	-2.615230261	0.008916732	0.041549357
Fam174b	11.76946113	-0.906321903	0.361741215	-2.505442744	0.01222982	0.053617559
Fam193a	555.8791977	-0.30214372	0.104174151	-2.900371326	0.003727208	0.020458747
Fam193b	189.6563876	-0.453763184	0.130213508	-3.484762765	0.000492574	0.003632458
Fam20a	62.36011691	0.52836334	0.197805915	2.671120026	0.007559861	0.036679868
Fam20c	1769.57221	-0.621958573	0.094714793	-6.566646566	5.15E-11	1.44E-09
Fam214a	162.8150314	-1.0681731	0.149706797	-7.135100906	9.67E-13	3.36E-11
Fam222b	248.436955	0.227125901	0.113222279	2.006017738	0.044854356	0.146619323
Fam234a	857.7324381	-0.214812732	0.088287046	-2.433117223	0.014969454	0.062855789
Fam241a	333.4895094	-0.977521222	0.14117179	-6.924338239	4.38E-12	1.44E-10
Fam43a	460.8177863	0.606845117	0.155579231	3.90055351	9.60E-05	0.000871305
Fam45a	258.554404	0.322132709	0.118898299	2.709313004	0.00674227	0.033404175
Fam49a	261.0387785	-0.476194739	0.1349021	-3.529928284	0.000415672	0.00315688
Fam49b	1434.62513	0.583929058	0.103092848	5.664108342	1.48E-08	2.91E-07
Fam57a	25.38595419	0.92673234	0.278509938	3.327465961	0.000876397	0.00595298
Fam72a	29.78951561	0.930499239	0.261663985	3.556084489	0.000376423	0.002888027
Fam76a	402.542946	0.227939086	0.104310691	2.185193905	0.028874632	0.105946603
Fam78a	50.82770141	0.486024713	0.209922515	2.315257667	0.020598835	0.081242238
Fam78b	79.97077987	-0.811723542	0.18251785	-4.447365235	8.69E-06	0.000103046
Fam83d	35.19049805	1.417371092	0.286551354	4.946307433	7.56E-07	1.11E-05

Fam83f	5.865932689	-1.302453766	0.381083778	-3.417762294	0.000631382	0.004494579
Fam8a1	365.5328083	-0.288777129	0.103556132	-2.78860483	0.005293561	0.027446644
Fam91a1	885.7120658	0.400364228	0.091690554	4.366471924	1.26E-05	0.000145316
Fanca	18.63692263	0.879648331	0.314914448	2.793293026	0.005217441	0.027102157
Fancd2	24.07291656	1.560448075	0.283858271	5.497278867	3.86E-08	7.04E-07
Fance	245.4322182	-0.572580571	0.128038928	-4.471925684	7.75E-06	9.28E-05
Fancg	52.38131748	0.703932684	0.222799311	3.159492189	0.001580443	0.009903012
Fanci	9.323977522	1.377094964	0.373800774	3.684034549	0.000229571	0.001884668
Fancl	89.45206741	0.359073716	0.176746852	2.031570648	0.042197141	0.140135481
Fancm	67.35189836	0.519831853	0.192020416	2.707169702	0.006785956	0.033576275
Far1	978.0312145	0.305259542	0.099871364	3.056527222	0.002239171	0.013214017
Farp1	23.8409598	-0.996136213	0.298961173	-3.331991917	0.000862268	0.005871166
Farsa	782.3525169	0.330446436	0.09893526	3.340026952	0.000837703	0.005724683
Farsb	609.2639485	0.365158014	0.113465614	3.218226233	0.00128986	0.008297014
Fasn	205.6590408	0.477153356	0.132530628	3.600325165	0.000317819	0.002501058
Fastkd3	104.9192614	0.625040094	0.172981463	3.613335683	0.000302283	0.002393858
Fat1	51.25889431	-1.449719163	0.249155149	-5.818539846	5.94E-09	1.24E-07
Fblim1	1521.266788	-0.466887106	0.090151851	-5.178896536	2.23E-07	3.57E-06
Fbln1	4.17025453	-1.055563765	0.38137416	-2.767790467	0.005643773	0.028965834
Fbln2	255.3635483	-1.134708441	0.112617291	-10.07579233	7.07E-24	9.51E-22
Fbn1	63.37766489	-1.275692108	0.190102198	-6.710559471	1.94E-11	5.66E-10
Fbn2	9.547678843	-1.151311994	0.363750754	-3.16511232	0.001550231	0.009738098
Fbrs	444.4067974	-0.249589844	0.094027871	-2.654424077	0.007944392	0.038077765
Fbxl12	119.9556892	-0.562854309	0.150346792	-3.743706824	0.000181325	0.001525307
Fbxl17	259.5126584	-0.295775756	0.109508032	-2.700950337	0.006914166	0.034145794
Fbxl20	299.5295631	-0.305214414	0.124301681	-2.455432719	0.014071509	0.059741108
Fbxl4	47.41323488	0.612787224	0.233059666	2.629314779	0.008555713	0.040334562
Fbxo21	280.9409343	0.375263181	0.132738316	2.827090117	0.004697311	0.024849726
Fbxo22	318.4492862	0.338843286	0.10766021	3.147340021	0.001647632	0.010247003
Fbxo31	148.4356667	-0.340743337	0.135180976	-2.520645639	0.011713975	0.05174908
Fbxo32	355.155012	-2.011223379	0.135037808	-14.8937798	3.62E-50	2.86E-47
Fbxo33	621.6184838	0.390578015	0.087274834	4.475265061	7.63E-06	9.15E-05
Fbxo40	2.085474138	0.691398489	0.348739063	1.982566801	0.047415839	0.152772799
Fbxo5	101.6493689	1.262549231	0.203206749	6.213126474	5.19E-10	1.27E-08
Fbxo6	273.064905	-0.225564684	0.114766259	-1.965426824	0.049364857	0.156820155
Fbxw5	115.6780584	-0.409437678	0.152490172	-2.685010276	0.007252758	0.035453464
Fcer1g	9398.380356	0.213609054	0.063680427	3.354391022	0.000795399	0.005479442
Fcgr1	300.8543501	0.483102169	0.181929419	2.655437322	0.007920565	0.037994127
Fcgr2b	4138.250672	0.810779258	0.13393192	6.053667091	1.42E-09	3.28E-08
Fcgr4	303.7826513	0.70826264	0.281506494	2.515972649	0.011870439	0.052317088
Fcgrt	625.6694082	0.854610166	0.131633312	6.492354827	8.45E-11	2.30E-09
Fchsd2	199.5173726	0.668215911	0.193319438	3.456537632	0.000547163	0.003984732
Fcna	1032.83712	0.698488401	0.117785982	5.930148831	3.03E-09	6.58E-08
Fcrls	204.5039602	0.957972276	0.158193991	6.055680561	1.40E-09	3.24E-08

Fen1	153.5056328	0.932535618	0.182756455	5.102613851	3.35E-07	5.19E-06
Fer	359.3594265	0.257810791	0.101178124	2.548088274	0.010831506	0.048579919
Fermt2	84.76976995	-0.940540261	0.208165532	-4.518232451	6.24E-06	7.60E-05
Fermt3	1544.083903	0.225893589	0.076493116	2.953123093	0.003145765	0.017740885
Fes	635.9948501	-0.236844693	0.089353957	-2.650634632	0.00803407	0.038446202
Fez2	514.1960073	0.246934354	0.084658913	2.91681459	0.003536259	0.019546461
Ffar4	7.989032687	-1.466816611	0.376725691	-3.893593264	9.88E-05	0.000895075
Fgd2	129.7845531	1.409888283	0.200114816	7.045396801	1.85E-12	6.26E-11
Fgd6	255.735365	-0.292186379	0.128546714	-2.272997653	0.023026321	0.088928209
Fgf11	4.160368214	0.772675118	0.380658789	2.029836538	0.042373158	0.14054284
Fgf13	12.12894768	-1.492864437	0.367639713	-4.060672409	4.89E-05	0.000480425
Fgf2	34.13568876	-0.689607927	0.259459777	-2.657860633	0.00786384	0.037805947
Fgf7	4.542701112	1.434213126	0.379727846	3.776950101	0.000158761	0.001355244
Fgfr2	11.27695626	-1.139127644	0.347061456	-3.2822073	0.001029979	0.006836628
Fgl2	160.0996575	-0.956632957	0.23342501	-4.098245329	4.16E-05	0.000417323
Fgr	353.7664474	-0.949815166	0.144451426	-6.575325656	4.85E-11	1.37E-09
Fh1	332.6754496	0.515761553	0.140104108	3.681273581	0.000232072	0.001902076
Fhl1	12.56334592	-1.306153604	0.34257927	-3.812704729	0.000137454	0.001199289
Fhod1	150.8513066	0.415097496	0.145912409	2.844840261	0.00444337	0.023773416
Fibp	299.8199177	-0.25408971	0.106073254	-2.395417314	0.016601458	0.068275715
Fignl1	50.15143958	1.593500272	0.270609913	5.888550987	3.90E-09	8.36E-08
Fignl2	65.66657181	-0.427842929	0.189418306	-2.258720068	0.023900803	0.091590641
Filip1l	420.4460812	0.368140307	0.186242341	1.976673541	0.048078544	0.153972408
Firre	114.5460765	-0.43599863	0.153113371	-2.847554244	0.004405659	0.023612756
Fis1	500.0459975	-0.326278013	0.101578138	-3.212088926	0.001317736	0.008430796
Fkbp10	54.11257218	-0.875619142	0.233103622	-3.756351512	0.000172408	0.001459297
Fkbp11	37.27131895	-0.68574149	0.238910003	-2.870292078	0.004100928	0.022190784
Fkbp15	1014.377539	0.759724229	0.080037961	9.492048759	2.27E-21	2.24E-19
Fkbp3	674.6501977	0.408055639	0.087807839	4.647143626	3.37E-06	4.33E-05
Fkbp5	61.67196075	0.665438952	0.204576554	3.252762545	0.00114289	0.007474127
Fli1	2075.695319	0.440410714	0.071489233	6.160518139	7.25E-10	1.74E-08
Flna	1728.294262	-0.495951867	0.083210548	-5.960204288	2.52E-09	5.54E-08
Flnb	565.8884293	-0.500422492	0.130292907	-3.840750065	0.000122659	0.00108791
Flnc	27.09999395	-1.041107773	0.30548363	-3.408064031	0.000654255	0.004620429
Flot1	581.029607	0.506775694	0.098760619	5.13135395	2.88E-07	4.51E-06
Flrt1	2.839260697	-0.775016875	0.373083009	-2.077330934	0.037771026	0.129459913
Flrt2	879.7182717	-1.06935638	0.140345728	-7.619443749	2.55E-14	1.09E-12
Flrt3	42.55061232	-0.606412263	0.270032011	-2.245705092	0.024722904	0.094023471
Flt1	15.21613694	-1.001684802	0.347280161	-2.884370933	0.003921965	0.02136113
Flvcr1	711.4548305	-0.463227047	0.128988233	-3.59123493	0.000329115	0.002569732
Fmn1	412.6734868	-0.26078356	0.112036764	-2.32766059	0.019930133	0.079124738
Fmnl3	156.7467281	-0.938494266	0.164590106	-5.702008989	1.18E-08	2.36E-07
Fmod	4.340153565	-1.251593698	0.381297179	-3.282462515	0.001029047	0.006836628
Fn1	14058.18473	-0.537653181	0.206575422	-2.602696751	0.009249373	0.042722479

Fnbp1	1285.702074	0.249322179	0.064959539	3.838114984	0.000123982	0.001095998
Fnbp1l	247.1645557	-0.37320881	0.13151732	-2.837716061	0.004543758	0.024180206
Fndc3a	1336.50377	0.184846041	0.076964179	2.401715241	0.016318406	0.067296047
Fndc3b	1850.499109	0.376170522	0.086676684	4.339927473	1.43E-05	0.00016104
Fndc4	4.744858923	-1.097902782	0.380842601	-2.882825555	0.003941256	0.021436921
Fnip2	782.0293258	-0.436869675	0.104601065	-4.176531817	2.96E-05	0.000307823
Fnta	715.2654262	0.403246235	0.083206173	4.846349992	1.26E-06	1.78E-05
Folr2	629.8452593	1.40116433	0.145396814	9.63682968	5.59E-22	5.87E-20
Fos	230.6784183	0.467220647	0.124323635	3.758099954	0.000171208	0.001450777
Fosl2	806.8190105	0.504913574	0.14164779	3.564570797	0.000364452	0.002810551
Foxf2	10.03557718	-0.912433734	0.349951893	-2.6073119	0.009125619	0.042291918
Foxg1	4.189487729	1.625366817	0.376889726	4.312579258	1.61E-05	0.00018004
Foxj2	251.4969471	-0.340932623	0.111310347	-3.062901439	0.002192022	0.013027992
Foxm1	79.06727564	1.472903598	0.253574475	5.808564122	6.30E-09	1.31E-07
Foxn3	504.3368833	-0.271275806	0.091825922	-2.954239946	0.003134402	0.017696753
Foxo1	291.7922312	-0.314560345	0.112748277	-2.789934829	0.005271865	0.027347004
Foxo3	666.011854	-0.517716094	0.081138907	-6.380614563	1.76E-10	4.64E-09
Foxo4	112.4574343	-0.428717562	0.162643235	-2.635938484	0.008390494	0.039708655
Foxp1	2184.280802	-0.241604594	0.088265174	-2.737258471	0.00619536	0.03129376
Foxq1	38.90053425	-1.636645803	0.273898579	-5.975371648	2.30E-09	5.08E-08
Foxs1	10.26572145	-1.137493371	0.352600183	-3.22601469	0.001255269	0.008104446
Fpr1	638.8414625	2.222134448	0.255590459	8.694121274	3.50E-18	2.50E-16
Frat2	35.02495012	-0.567468385	0.264744456	-2.143457109	0.032076412	0.114940475
Frmd4b	1385.733388	0.403613037	0.090362603	4.466593763	7.95E-06	9.49E-05
Frmd6	82.06551884	-0.865255894	0.190038609	-4.553053192	5.29E-06	6.56E-05
Fsbp	5.083772899	0.994982035	0.381390409	2.608828146	0.009085286	0.042185298
Fscn1	376.5999073	-1.007748987	0.138071365	-7.298754412	2.90E-13	1.08E-11
Fstl1	257.3437027	-1.154093184	0.126394783	-9.13086094	6.80E-20	5.73E-18
Fstl3	14.49514344	-0.680311536	0.313686549	-2.168762217	0.030100741	0.109557675
Fth1	112654.2089	-0.485751172	0.070475362	-6.892496256	5.48E-12	1.75E-10
Fth-ps2	4.262264936	-0.808052731	0.381385715	-2.118728362	0.03411343	0.120428192
Ftsj3	496.7138417	0.271578622	0.115808213	2.345072204	0.019023395	0.076139995
Fuca1	1319.603969	-0.469944644	0.097183304	-4.835652074	1.33E-06	1.87E-05
Fuca2	748.7048426	0.467349044	0.103025621	4.536240961	5.73E-06	7.06E-05
Fundc1	712.1200489	0.216712096	0.086980593	2.491499408	0.012720518	0.05526833
Fundc2	841.3237285	0.302351826	0.092536867	3.267366137	0.001085532	0.007154404
Fus	3041.594598	0.593586892	0.11427988	5.194150462	2.06E-07	3.31E-06
Fut8	115.5333229	0.567307675	0.151692543	3.739852102	0.000184129	0.001546288
Fxyd2	32.73737087	1.090089105	0.258894475	4.210553763	2.55E-05	0.000268844
Fxyd6	5.092020031	-0.864231606	0.379387305	-2.27796659	0.022728568	0.087954519
Fyb	5149.827149	-0.254773037	0.088725433	-2.871476958	0.004085586	0.022134435
Fyttd1	528.8069821	0.259782272	0.110505558	2.35085254	0.018730455	0.075217538
Fzd2	22.75429651	-0.84012053	0.295645162	-2.84165154	0.004488052	0.023968751
Fzd3	6.779811029	-0.810582922	0.368224006	-2.20133101	0.027712599	0.102429194

Fzd4	72.7222334	-0.498171473	0.202991521	-2.454149175	0.014121838	0.059917
Fzd7	292.30998	-0.655617643	0.147018994	-4.459407768	8.22E-06	9.79E-05
Fzr1	239.860242	0.440699939	0.141346144	3.117877347	0.001821586	0.011120307
G2e3	269.6911247	0.450273531	0.12651486	3.55905646	0.00037219	0.002864332
G3bp1	1147.817849	0.288278814	0.084504613	3.411397386	0.000646308	0.004572913
G3bp2	2111.9266	0.295144446	0.088242502	3.344697171	0.000823725	0.005639432
G6pc3	117.4042805	-0.355514366	0.168497246	-2.109912028	0.034865934	0.122369893
G6pdx	881.650805	-0.222198491	0.09517603	-2.33460558	0.019564035	0.077918649
Gaa	326.9362211	-0.363892137	0.107740225	-3.377495611	0.000731491	0.005096327
Gab2	878.4457906	-0.34952638	0.112029046	-3.119962129	0.001808743	0.01105654
Gab3	368.0650439	0.893616659	0.102652436	8.705265015	3.17E-18	2.29E-16
Gabarapl1	1272.965291	-0.937829497	0.096298902	-9.73873512	2.06E-22	2.31E-20
Gabbr2	109.126717	-1.439782915	0.187113843	-7.69468944	1.42E-14	6.30E-13
Gabpa	201.8593861	0.41372063	0.120369797	3.437080077	0.000588022	0.004234442
Gad1-ps	6.407042061	0.953864875	0.380950951	2.503904697	0.012283112	0.053741299
Gadd45a	109.6215807	-1.289963902	0.186744431	-6.907643233	4.93E-12	1.59E-10
Gadd45b	113.3280843	-1.417436192	0.205307862	-6.903954756	5.06E-12	1.63E-10
Gadd45g	125.7945993	-0.889131083	0.179623593	-4.949968266	7.42E-07	1.10E-05
Galc	587.4145185	-0.320397019	0.097213467	-3.295808999	0.000981387	0.006565088
Galk1	245.8531993	0.646433578	0.125773296	5.139672727	2.75E-07	4.33E-06
Galk2	423.5786818	0.463342485	0.107940374	4.292578099	1.77E-05	0.000195601
Galm	113.8293342	-0.534990962	0.169482028	-3.156623557	0.001596073	0.009988432
Galnt3	6.407183798	1.402846008	0.379859407	3.693066383	0.000221566	0.001827951
Galnt4	172.1607971	0.283946879	0.12867816	2.206643912	0.027338947	0.101452911
Galnt7	1442.140917	-0.330687615	0.08420386	-3.927226319	8.59E-05	0.000791146
Galnt9	156.1379353	-1.435555624	0.190401899	-7.53960769	4.71E-14	1.94E-12
Gamt	116.3063805	0.370056573	0.151737464	2.438795031	0.014736324	0.061998322
Ganc	225.1238435	-0.356571707	0.147553706	-2.416555417	0.015668141	0.065115423
Gapdh	166.8441319	0.793494066	0.166535606	4.764711193	1.89E-06	2.56E-05
Gapt	42.83630588	1.06989933	0.237632184	4.50233344	6.72E-06	8.13E-05
Gas2l3	1174.497501	-0.532968545	0.106943029	-4.983667955	6.24E-07	9.29E-06
Gas6	1659.546111	2.33094093	0.091909708	25.36120473	6.76E-142	3.38E-138
Gas7	1056.466871	-0.253627399	0.121681414	-2.084356108	0.037127787	0.127851469
Gask1b	352.9816381	-0.891740297	0.141589234	-6.298079795	3.01E-10	7.70E-09
Gata2	53.06828011	-1.324295964	0.225516572	-5.872277814	4.30E-09	9.11E-08
Gata3	14.46612798	-0.815866764	0.327186202	-2.493585488	0.012646013	0.055024272
Gata4	6.082493387	-0.983394375	0.376833972	-2.609622402	0.009064221	0.04211355
Gata6	283.2771831	-0.656139875	0.159436251	-4.115374451	3.87E-05	0.000390113
Gatad2b	1226.798186	-0.214204169	0.081292078	-2.634994386	0.008413867	0.039769105
Gatd3a	327.7826815	0.266900771	0.126886976	2.103452841	0.035426204	0.123810209
Gba	487.5858714	0.279438397	0.117005509	2.388249911	0.016928826	0.069374901
Gbe1	1215.368636	0.387646371	0.110879912	3.496091965	0.000472126	0.003512739
Gbp2	333.9130563	-1.303234954	0.38057951	-3.424343456	0.000616287	0.004404669
Gbp2b	8.881393006	-1.237700523	0.378805361	-3.267378584	0.001085484	0.007154404

Gbp3	136.9018723	-0.789731603	0.35486394	-2.225449011	0.02605111	0.097888885
Gca	70.26140456	-0.469009663	0.181890306	-2.578530291	0.00992216	0.045149138
Gcat	35.58343748	0.807362332	0.25724365	3.138512195	0.001698079	0.010510456
Gcnt1	167.4651545	0.532775914	0.207453786	2.568166743	0.010223796	0.046211259
Gcsh	255.8601663	0.384642644	0.126410403	3.04280846	0.002343815	0.0137612
Gda	5721.137178	1.447830442	0.261418646	5.538359502	3.05E-08	5.67E-07
Gdf15	315.3097896	1.104770699	0.159931311	6.907782403	4.92E-12	1.59E-10
Gdi1	159.3799388	-0.321680055	0.136058255	-2.364281801	0.018065065	0.073053742
Gen1	11.64054665	1.334043555	0.352391296	3.785688157	0.000153284	0.001314474
Gfm1	218.39779	0.263138553	0.113704615	2.314229309	0.020655147	0.08144293
Gfra2	16.94766624	-0.747124657	0.305936385	-2.442091536	0.014602444	0.061504033
Gfra4	7.675722007	1.45651389	0.37583468	3.875411097	0.000106445	0.000958259
Ggact	38.09663332	0.833632642	0.263365789	3.165303455	0.001549213	0.009735778
Ggt5	36.37324489	0.539688633	0.248239141	2.174067436	0.029700071	0.108407952
Ggta1	176.240922	0.623585122	0.163234596	3.820177453	0.000133356	0.001168273
Ghdc	158.6289243	0.371706267	0.165835152	2.241420241	0.024998867	0.094929886
Gigyf1	624.0515092	-0.256023031	0.103387411	-2.476346294	0.013273476	0.057207083
Gins1	68.66907982	1.127651811	0.218240269	5.167019893	2.38E-07	3.80E-06
Gins2	109.5552563	1.441822355	0.206991359	6.965616167	3.27E-12	1.09E-10
Gins3	61.546325	0.641928512	0.200056235	3.208740344	0.001333178	0.008520872
Gins4	217.7893379	0.293810236	0.140407813	2.092549056	0.036389428	0.126060977
Gipc1	159.6475447	0.345758881	0.138023032	2.505081036	0.012242334	0.053656749
Gja1	51.87503001	-0.6662415	0.209085728	-3.186451351	0.001440297	0.009104692
Gjb3	69.13902755	-1.22232828	0.204468647	-5.978071927	2.26E-09	5.01E-08
Gjb5	11.78326064	-1.180535788	0.335534987	-3.518368678	0.000434209	0.003282705
Gk	224.1382415	0.450933625	0.118892388	3.792788032	0.000148965	0.001283308
Glg1	3146.071125	0.160731103	0.077682054	2.069089261	0.038537711	0.131320489
Glipr1	3036.179867	-0.956394064	0.121238979	-7.888503146	3.06E-15	1.49E-13
Glipr1l1	30.30617623	1.333938532	0.273601784	4.875474543	1.09E-06	1.55E-05
Glipr2	69.89550892	-0.75763852	0.200399649	-3.780637962	0.000156427	0.001338369
Glmn	104.503818	0.453580176	0.147408154	3.077035865	0.002090701	0.012515856
Glrp1	11.07035814	0.71127293	0.340125833	2.091205257	0.036509668	0.126331702
Glrx	530.2258442	1.086680158	0.127230647	8.541025152	1.33E-17	8.87E-16
Glrx2	418.9489805	0.217257479	0.10032345	2.165570251	0.030344042	0.110099863
Glrx5	475.595918	0.252502601	0.098022269	2.575971797	0.00999588	0.045396003
Gltp	1996.482627	-0.174409928	0.079714319	-2.187937249	0.028674174	0.10528831
Glud1	1803.308746	0.210778583	0.081636736	2.581908503	0.009825563	0.044831932
Gm10093	4.055142972	1.12389214	0.380351689	2.954876163	0.003127946	0.017680257
Gm10110	7.314392418	-0.728119003	0.367689008	-1.980257739	0.047674575	0.153300267
Gm10263	43.37242396	1.526023019	0.250674003	6.087679624	1.15E-09	2.69E-08
Gm10313	3.146595221	1.10880993	0.374358605	2.961892462	0.003057546	0.017321474
Gm10425	3.629963095	-0.900754629	0.381138613	-2.363325565	0.018111751	0.073194668
Gm10478	53.11279133	-0.539554445	0.225471583	-2.393004204	0.01671105	0.068630894
Gm10521	84.44317115	-0.566710367	0.218158123	-2.597704631	0.009384918	0.043175803

Gm10603	2.714710132	-1.16156473	0.362266831	-3.206378916	0.001344169	0.008569218
Gm11110	15.37752203	1.291514598	0.322560588	4.003944205	6.23E-05	0.000595074
Gm11450	14.43441033	-0.641402991	0.322677759	-1.987750857	0.046839251	0.151588666
Gm11696	23.74876888	-0.567957937	0.276616273	-2.053234003	0.040049888	0.135123352
Gm11725	2.279507034	-0.780884583	0.354532467	-2.202575664	0.027624671	0.102134376
Gm11942	91.02715387	4.260017352	0.288691882	14.75627689	2.80E-49	1.91E-46
Gm11953	38.07493146	0.525259368	0.243117835	2.160513515	0.030732939	0.11109865
Gm12216	19.77434219	-1.067232093	0.308937342	-3.454526047	0.000551261	0.004008127
Gm12258	36.30422994	-0.607108419	0.237991995	-2.550961508	0.010742619	0.048253362
Gm12351	1.510308766	-0.616283566	0.313904159	-1.963285761	0.049612979	
Gm12708	32.22710535	-1.077256335	0.254463037	-4.233449174	2.30E-05	0.00024581
Gm12992	4.359028439	-0.856962394	0.381189151	-2.248129023	0.024567964	0.09357726
Gm13293	4.279158132	-1.146625907	0.381365758	-3.006630471	0.002641607	0.015300112
Gm13340	34.68749326	-0.592752813	0.266420453	-2.22487728	0.026089478	0.097930163
Gm13986	173.1806348	1.173291424	0.301495689	3.891569487	9.96E-05	0.000901487
Gm14085	4.067500098	-0.749607614	0.380771768	-1.968653345	0.048992912	0.155968739
Gm14124	1.09279576	0.602442559	0.300767556	2.003017105	0.045175453	
Gm14279	2.857560402	-0.917586047	0.372300929	-2.464635396	0.013715273	0.058648835
Gm14328	55.79446793	-0.708282865	0.231333107	-3.061744492	0.002200512	0.013047445
Gm14461	36.86523938	-0.901450084	0.286632806	-3.144964799	0.001661068	0.010317736
Gm14636	110.8683752	-1.040431747	0.17904343	-5.811057948	6.21E-09	1.29E-07
Gm15270	1.491502548	0.806505862	0.329523987	2.447487572	0.014385609	
Gm15344	1.518196098	-0.721542358	0.334682219	-2.155902877	0.03109125	
Gm15420	2.08757284	-0.696003758	0.345674353	-2.013466579	0.044065567	0.144563094
Gm15513	106.4332377	-1.58598207	0.171216963	-9.262996134	1.99E-20	1.77E-18
Gm15564	11.85331999	-0.992187186	0.33622117	-2.950995577	0.003167515	0.017836732
Gm15672	3.498599241	-0.847588914	0.376408431	-2.251779835	0.024336189	0.092858679
Gm15706	25.35728003	0.841604186	0.272668522	3.086546915	0.00202496	0.012158466
Gm15740	4.024394447	-0.775917675	0.380929085	-2.036908456	0.041659217	0.138928858
Gm15747	3.587280493	-0.984260231	0.377333666	-2.608461207	0.009095032	0.04220408
Gm15832	51.03717113	-1.416482081	0.226507797	-6.253568746	4.01E-10	1.01E-08
Gm15931	63.87138108	-0.825597777	0.213067791	-3.874812679	0.000106707	0.000960041
Gm16014	22.07099461	0.764620334	0.341075066	2.241794872	0.024974633	0.094908664
Gm16104	24.19294698	1.330301738	0.327893041	4.05712099	4.97E-05	0.000486663
Gm16287	43.40035044	-1.027495875	0.22516575	-4.56328672	5.04E-06	6.27E-05
Gm16556	11.79510514	-1.109392205	0.335303252	-3.308623462	0.000937558	0.006306561
Gm16565	6.234839964	-0.761693448	0.37755157	-2.017455382	0.043648013	0.143614498
Gm16587	26.25896602	-0.601859967	0.262464895	-2.293106542	0.021841872	0.085182165
Gm17324	0.932998858	-0.573404536	0.286760873	-1.999591264	0.045544418	
Gm19552	8.721025739	-0.777805541	0.361799671	-2.149823796	0.031569155	0.113583867
Gm20342	61.03547014	-0.670898467	0.19128296	-3.50736139	0.000452574	0.003399289
Gm20492	39.35077816	-0.682953643	0.250936501	-2.721619376	0.006496292	0.032431753
Gm20619	119.1950166	0.483915601	0.14576244	3.319892296	0.000900522	0.006092936
Gm20632	34.08175141	-0.582551582	0.25795163	-2.258375267	0.023922273	0.091606585

Gm20658	57.78885692	-1.271680945	0.243660296	-5.219073294	1.80E-07	2.91E-06
Gm20667	16.04283859	1.221693386	0.342782891	3.56404423	0.000365184	0.002814752
Gm20682	5.608029612	-0.939731313	0.378721966	-2.481322439	0.013089591	0.056560751
Gm20696	32.83669501	-0.533916603	0.26461356	-2.017722001	0.043620222	0.143602831
Gm20703	27.30579073	-0.739725017	0.257164113	-2.876470623	0.004021496	0.02180296
Gm20712	72.93118982	0.42939158	0.18933472	2.267896661	0.023335506	0.08981686
Gm21092	56.96194893	-0.464068294	0.217071535	-2.137858819	0.032528203	0.116032981
Gm21986	5.462735014	-0.761708524	0.377630351	-2.017074425	0.043687747	0.143682231
Gm23127	13.52567806	-0.661758092	0.321802021	-2.056413721	0.039742655	0.134291089
Gm23318	5.596108024	-0.766039946	0.378922469	-2.021627136	0.043214886	0.142689945
Gm23722	68.58459037	-1.143159855	0.204837357	-5.580817253	2.39E-08	4.52E-07
Gm25311	1.178059648	0.624422601	0.304277067	2.052151377	0.040154953	
Gm26497	14.33031504	-0.626149278	0.313885655	-1.99483241	0.046061161	0.14981134
Gm26532	66.65138099	-0.605056684	0.228420542	-2.648871594	0.008076101	0.038598104
Gm26652	4.635817305	-1.01181111	0.381041722	-2.655381422	0.007921878	0.037994127
Gm26674	7.243854423	1.034947879	0.379158264	2.729593358	0.006341249	0.031816491
Gm26714	22.3884923	-0.935735881	0.296985681	-3.150777764	0.001628363	0.010148193
Gm26917	1061.78618	-0.910706165	0.116388259	-7.82472537	5.09E-15	2.40E-13
Gm26930	20.04399881	-1.092135532	0.305023191	-3.580499992	0.000342937	0.002656924
Gm27010	41.55899338	-0.68253348	0.241350719	-2.827973678	0.004684366	0.024817951
Gm2830	10.00711941	-0.777741073	0.344056144	-2.260506281	0.023789847	0.091298095
Gm28438	12.41622648	0.791438132	0.349702113	2.263178005	0.023624718	0.090743832
Gm28557	22.98961546	-0.70279691	0.290964825	-2.41540162	0.015717868	0.065303999
Gm29291	6.671090952	-0.945151635	0.375400952	-2.517712408	0.011811972	0.05209
Gm29358	9.328474169	-0.714897375	0.360968641	-1.980497176	0.04764769	0.153279505
Gm2a	1501.787353	-0.993527274	0.082281853	-12.07468284	1.44E-33	4.23E-31
Gm30211	6.488011164	0.805723957	0.378254401	2.130111256	0.033162429	0.117763506
Gm32051	4.902567975	0.746945128	0.380624741	1.962418751	0.049713751	0.157694836
Gm33142	15.30315122	-0.788091887	0.330320465	-2.385840331	0.017040148	0.069707893
Gm33973	4.618108332	0.79407296	0.381369076	2.082164002	0.037327492	0.128362437
Gm36371	13.27978179	-0.843888262	0.320833703	-2.630298044	0.008531004	0.040246708
Gm36955	13.62511519	-1.123391408	0.324667341	-3.460130621	0.000539913	0.003938979
Gm36989	3.428892898	-0.755407137	0.378909294	-1.993635812	0.046191869	0.150076071
Gm37010	132.0899936	-0.685956639	0.223531171	-3.068729237	0.002149713	0.012822237
Gm37033	220.1278589	-0.901843888	0.201921837	-4.466301915	7.96E-06	9.49E-05
Gm37124	17.23531612	-0.87648144	0.315855044	-2.77494837	0.005521047	0.028403961
Gm37125	15.44896452	0.765893709	0.311602124	2.45792198	0.013974353	0.059440585
Gm37159	6.219282255	-0.888178228	0.379220541	-2.34211529	0.019174791	0.076605389
Gm37168	36.54875088	-2.121339524	0.29951371	-7.08261242	1.41E-12	4.86E-11
Gm37199	28.75591387	-1.497358674	0.311193456	-4.811665046	1.50E-06	2.08E-05
Gm37336	1.879486508	-0.728654114	0.347374471	-2.097604098	0.035940133	
Gm37399	65.09894993	-0.528198117	0.231532701	-2.281311081	0.022530045	0.087298836
Gm37420	79.33081281	-0.914802346	0.2193718	-4.170100015	3.04E-05	0.000314895
Gm37423	36.60038551	-0.725434486	0.234993115	-3.087045702	0.002021565	0.012149631

Gm37452	7.001351356	-0.968395964	0.37708971	-2.568078471	0.0102264	0.046211259
Gm37472	79.43600552	-0.972124611	0.208379809	-4.665157424	3.08E-06	4.00E-05
Gm37494	322.9656175	-0.224639007	0.109163777	-2.057816365	0.039607764	0.134053613
Gm37534	11.43017879	-0.706289626	0.332930239	-2.121434296	0.033885272	0.119847042
Gm37589	16.27738086	-0.758005338	0.323650368	-2.342049979	0.019178147	0.076605389
Gm37598	11.95700222	-0.909005999	0.335495539	-2.709442876	0.006739631	0.033402127
Gm37642	88.47111629	-0.70790086	0.244344647	-2.897140851	0.003765806	0.020632879
Gm37666	10.23920351	-0.714833599	0.351353625	-2.034513233	0.041899876	0.13954537
Gm37696	28.72306576	0.792097026	0.291442658	2.717848619	0.006570789	0.032736068
Gm37718	22.86307031	-0.793513136	0.295772833	-2.682846587	0.007299847	0.035590904
Gm37728	26.84608335	-0.927356955	0.262549093	-3.532127815	0.00041223	0.003133909
Gm37786	2.370094417	0.727390235	0.366256393	1.986013754	0.047031797	0.151916955
Gm37795	13.93514492	-1.211179221	0.326678986	-3.707551675	0.000209273	0.001736073
Gm37899	4.549983894	-1.125488378	0.381229423	-2.952260003	0.003154572	0.017783869
Gm37933	14.35615047	-0.73917812	0.330641637	-2.235586922	0.025378842	0.095910421
Gm38091	2.03503766	-0.807535688	0.351972013	-2.294317894	0.021772245	0.084954781
Gm38120	29.71089198	-0.561915759	0.278842051	-2.015175817	0.04388623	0.14424018
Gm38137	1.477602338	0.656891631	0.333118135	1.971947974	0.048615546	
Gm38236	17.54525209	-0.869068201	0.301171375	-2.885626834	0.00390635	0.021301816
Gm38248	47.53166742	0.618225111	0.217528881	2.842036927	0.00448263	0.023956847
Gm38335	60.23903798	-1.276194224	0.198666529	-6.423800881	1.33E-10	3.55E-09
Gm38340	6.131782292	-0.982317967	0.379692554	-2.587140458	0.009677613	0.044285963
Gm38366	30.62094738	-0.542823759	0.261629099	-2.074783581	0.038006597	0.12987856
Gm38377	17.84180268	-0.889556514	0.312076681	-2.850442114	0.00436585	0.023441254
Gm38832	84.02257516	1.276558341	0.204378036	6.246064222	4.21E-10	1.05E-08
Gm38843	41.78308287	1.698903337	0.256345828	6.62738829	3.42E-11	9.84E-10
Gm42418	640241.1806	-0.305924098	0.101575764	-3.011782403	0.002597187	0.015089425
Gm42466	38.05276211	-0.676374414	0.243703037	-2.77540412	0.005513315	0.02837391
Gm42482	4.229334014	-1.040478602	0.381268929	-2.728988708	0.006352888	0.031842949
Gm42550	7.7084529	-1.19373018	0.377779134	-3.159862665	0.001578435	0.00989456
Gm42551	25.98426542	-0.994884217	0.282257015	-3.524745766	0.000423889	0.00321603
Gm42572	11.81820366	-0.793646246	0.332655888	-2.385787458	0.017042597	0.069707893
Gm42576	57.73895827	-0.592974972	0.229815953	-2.580216753	0.009873832	0.045011116
Gm42603	9.781452831	-0.845067796	0.358439588	-2.357629634	0.018392032	0.074076551
Gm42636	24.88970473	-0.773222819	0.270184991	-2.861827434	0.004212062	0.022713047
Gm42639	2.632980316	-0.973856086	0.362119062	-2.689325665	0.007159653	0.035078328
Gm42659	66.51769949	-0.422857094	0.205635541	-2.056342461	0.039749518	0.134291089
Gm42715	57.74278525	-0.419124997	0.201587257	-2.079124462	0.037605912	0.129053722
Gm42722	4.313966581	0.872651746	0.380212568	2.295168071	0.021723494	0.084808657
Gm42772	2.879098385	-0.852996509	0.363816766	-2.344577243	0.019048664	0.076189581
Gm42820	27.30353763	-0.545376913	0.2628801	-2.074622285	0.038021555	0.129886063
Gm4285	48.9889598	-0.940233123	0.215557589	-4.361865083	1.29E-05	0.000147815
Gm42868	2.515394181	-1.025397687	0.366248733	-2.79973033	0.005114531	0.026650616
Gm42879	4.351789552	-0.786009474	0.38116516	-2.062123077	0.03919602	0.133200786

Gm42908	18.04849383	-0.60008795	0.302273449	-1.985248628	0.047116816	0.152152681
Gm42918	4.933724308	-0.801049834	0.380973314	-2.102640275	0.035497227	0.123971813
Gm43071	71.78431274	-0.550807539	0.213264464	-2.582744111	0.009801799	0.044750715
Gm43088	19.34098517	-1.014911925	0.316361323	-3.208078389	0.001336251	0.00853324
Gm43275	9.870238945	-0.926499444	0.351462805	-2.636123737	0.008385915	0.039699502
Gm43290	21.74139953	-0.692419955	0.30477167	-2.271930183	0.023090728	0.08912617
Gm43300	4.936910137	-0.829820058	0.381388586	-2.175786295	0.029571244	0.108027181
Gm43312	1.501791369	-0.725659328	0.33405973	-2.172244254	0.029837244	
Gm43323	104.1232167	-0.527839163	0.223409463	-2.36265356	0.018144623	0.073296461
Gm43336	125.3977026	-0.62572095	0.197029362	-3.175775139	0.001494367	0.009422677
Gm43343	21.48917409	-1.865592994	0.32490056	-5.74204303	9.35E-09	1.88E-07
Gm43429	2.804332617	0.731208256	0.370089734	1.97575936	0.048182038	0.154139384
Gm43430	42.45030033	-0.662363667	0.266215026	-2.488077687	0.012843567	0.055690094
Gm43445	41.70464459	-1.089043616	0.253569061	-4.294859993	1.75E-05	0.000193886
Gm43489	14.96619075	-1.022844082	0.333896851	-3.063353487	0.002188714	0.01301348
Gm43511	12.95816781	-0.764384805	0.3479499	-2.196824328	0.028032995	0.10336392
Gm43544	17.70845087	-0.81719444	0.295002865	-2.770123743	0.0056035	0.028778824
Gm43609	21.77382841	-0.653211564	0.309642341	-2.109567967	0.034895586	0.122369893
Gm43628	16.75498172	-0.651972387	0.319910204	-2.037985597	0.041551373	0.138758217
Gm43652	21.96683281	-1.344765961	0.293456346	-4.582507684	4.59E-06	5.77E-05
Gm43696	31.82855689	-0.565871334	0.261364164	-2.165068564	0.030382435	0.110212521
Gm43719	7.323799097	-1.594457842	0.377971266	-4.218463112	2.46E-05	0.000260135
Gm43788	49.60716667	-0.561226717	0.231690835	-2.422308665	0.015422245	0.064318515
Gm43792	8.159057324	-1.581695502	0.369920183	-4.275775089	1.90E-05	0.00020895
Gm43793	7.986677376	-1.334231362	0.366654644	-3.638932127	0.000273771	0.00220412
Gm43794	22.47578728	-2.195573881	0.33823762	-6.49121728	8.51E-11	2.31E-09
Gm43795	8.418131929	-1.163782428	0.374073321	-3.111107807	0.001863869	0.011315161
Gm43813	92.0352008	-0.616063056	0.204759382	-3.008717104	0.002623533	0.015213044
Gm43814	43.9539836	-1.38853397	0.22924291	-6.057042166	1.39E-09	3.23E-08
Gm43914	7.475703817	-1.858832316	0.377094654	-4.929352084	8.25E-07	1.21E-05
Gm44206	22.85351765	-0.952555142	0.27847442	-3.420619901	0.000624786	0.004452119
Gm44250	723.3368073	0.480427959	0.099113304	4.847260063	1.25E-06	1.77E-05
Gm44291	16.22194202	-0.656347195	0.307732678	-2.132848544	0.032937153	0.117213149
Gm44423	30.97633743	-0.650442911	0.274530452	-2.369292387	0.017822158	0.072266178
Gm44620	3.313349942	-0.76110626	0.35893558	-2.120453646	0.033967807	0.11995705
Gm44640	15.53044526	-0.663319462	0.327031658	-2.028303519	0.04252928	0.14095338
Gm44647	3.678066897	-0.80563224	0.378781878	-2.12690281	0.033428159	0.11851084
Gm44694	119.6981333	-0.576138269	0.15862625	-3.632048735	0.00028118	0.002255301
Gm44751	65.07051455	0.879084502	0.234256629	3.752655822	0.000174971	0.001478485
Gm44829	72.68513734	-0.840165477	0.182834621	-4.59522092	4.32E-06	5.48E-05
Gm44860	16.08939394	-0.858891608	0.339068836	-2.533089207	0.011306218	0.050276903
Gm44974	13.77237675	-1.546267132	0.364078639	-4.247069078	2.17E-05	0.000232829
Gm45120	5.238611666	-0.749476577	0.379543722	-1.97467784	0.048304719	0.154287898
Gm45153	14.69567989	-1.14889165	0.327713717	-3.505778344	0.000455274	0.00341615

Gm45191	35.52325811	-0.591682877	0.273595554	-2.162618759	0.030570514	0.110654054
Gm45223	105.8404924	-0.544157455	0.224157837	-2.427563821	0.015200612	0.063630568
Gm45828	82.51639535	-0.907532265	0.221249884	-4.10184289	4.10E-05	0.000411828
Gm45837	998.4667101	0.698775098	0.103366261	6.7601855	1.38E-11	4.10E-10
Gm45902	460.4934688	0.466307617	0.1057976	4.407544359	1.05E-05	0.000121909
Gm4673	31.71355521	-0.839688783	0.255465873	-3.286892196	0.001012996	0.006750458
Gm47854	23.66142934	0.672768815	0.270422506	2.48784328	0.012852035	0.055710715
Gm4841	7.816675157	-1.570762621	0.378003454	-4.155418703	3.25E-05	0.000333515
Gm49342	481.3267139	0.492318409	0.113272652	4.346313078	1.38E-05	0.000157159
Gm5113	20.88101813	-0.580796901	0.285517208	-2.034192286	0.041932212	0.139611234
Gm5150	192.1705802	1.122032841	0.182643465	6.143295843	8.08E-10	1.93E-08
Gm5523	5.368928914	1.029408445	0.37917967	2.714830269	0.006630975	0.032943729
Gm553	1.84500617	-0.73664793	0.347448813	-2.120162459	0.033992347	
Gm5547	60.54732855	-1.261727361	0.202971432	-6.216280548	5.09E-10	1.25E-08
Gm5617	95.85010516	-0.329215862	0.161593756	-2.03730559	0.041619428	0.13888316
Gm5644	5.651401371	-1.056004372	0.377770667	-2.795358306	0.005184222	0.02696694
Gm5863	5.659977482	0.767675046	0.380442117	2.017849793	0.043606907	0.143602831
Gm6166	27.26804326	-0.865888065	0.272387096	-3.178887979	0.001478412	0.009333837
Gm6209	11.0184892	-0.969449921	0.353992101	-2.738620209	0.00616976	0.03120098
Gm6345	4.648757733	-0.80478335	0.380067615	-2.117474151	0.034219627	0.120746283
Gm6377	83.5401297	-0.370503307	0.166236004	-2.228778948	0.025828616	0.097291678
Gm6946	4.98699282	0.797808558	0.380711608	2.095571924	0.036120182	0.125478974
Gm7308	24.15790575	-0.692078883	0.287476599	-2.407426847	0.01606538	0.066453463
Gm7863	2.0157289	-0.879282428	0.357675572	-2.458323956	0.013958719	0.059426526
Gm7967	4.167533416	1.03477307	0.379596952	2.725978344	0.00641112	0.032091956
Gm807	1.307830478	0.690049943	0.313674451	2.199892087	0.027814552	
Gm8093	7.315127549	1.398253831	0.373029524	3.748373098	0.000177985	0.001499734
Gm8995	1434.866591	-0.553215174	0.251596406	-2.198819854	0.027890734	0.102991203
Gm9403	29.67072595	-0.513124418	0.253726923	-2.022349112	0.043140298	0.142537749
Gm9725	1.573506001	0.813752776	0.32976525	2.467672913	0.013599452	
Gm973	7.50397059	-1.389625744	0.38138754	-3.643605517	0.000268845	0.002171454
Gm9812	5.13938913	-1.052376867	0.380552605	-2.76539131	0.005685455	0.029120007
Gmds	89.4835263	0.901693678	0.172432172	5.229265912	1.70E-07	2.77E-06
Gmfb	950.0873615	0.246328947	0.078347871	3.144041352	0.001666319	0.01034179
Gmfg	203.5152843	0.689640645	0.126071298	5.470243098	4.49E-08	8.13E-07
Gmfg-ps	9.522348211	0.761128425	0.357113176	2.131336719	0.033061412	0.117488186
Gmnn	226.5394688	0.926677911	0.167975058	5.516758994	3.45E-08	6.37E-07
Gna11	190.850302	-0.477795207	0.130055895	-3.673768154	0.000239	0.001951397
Gnai1	11.02211348	-1.144903578	0.35870214	-3.191794672	0.001413918	0.008960586
Gnai3	1520.369798	0.509640067	0.095042718	5.36222109	8.22E-08	1.41E-06
Gnaq	3221.035934	0.396126672	0.071395529	5.548340049	2.88E-08	5.39E-07
Gnas	4055.093918	-0.448239752	0.104934271	-4.271623999	1.94E-05	0.000211949
Gnb4	136.6871812	-0.579613486	0.142522129	-4.066831522	4.77E-05	0.000470206
Gng2	730.2477172	0.458767679	0.113151681	4.054448645	5.03E-05	0.000491472

Gngt2	599.9659518	-0.657399809	0.089516937	-7.34385953	2.08E-13	7.88E-12
Gnl1	260.5272961	-0.508875422	0.114603931	-4.440296403	8.98E-06	0.000106154
Gnl3	674.6514055	0.363257979	0.112726924	3.222459776	0.00127095	0.008192933
Gnpda1	93.21784551	0.741231107	0.165709889	4.473065027	7.71E-06	9.24E-05
Gns	2699.675764	-0.242530342	0.077319438	-3.136731829	0.001708423	0.010550743
Golga4	1051.129277	-0.222729395	0.105856499	-2.104069163	0.035372414	0.123651018
Golgb1	799.7759449	-0.26529204	0.119999624	-2.210773931	0.027051496	0.10068497
Golim4	671.0574627	-0.297967705	0.111648837	-2.668793631	0.007612421	0.03688718
Golph3	776.6309735	0.199687215	0.082588343	2.417861989	0.015611997	0.064936041
Golph3l	401.852322	-0.227450715	0.099611794	-2.28337133	0.022408505	0.086962614
Gon4l	163.5109202	-0.318755969	0.138050478	-2.308981271	0.020944621	0.0823032
Gopc	345.0766736	0.342892005	0.101689513	3.371950515	0.000746379	0.005185603
Gorasp1	58.56827204	0.492606719	0.208442987	2.363268373	0.018114546	0.073194668
Got1	512.5211202	-0.321223103	0.092219855	-3.483231495	0.0004954	0.003649713
Gpaa1	485.2505392	0.361218659	0.138976608	2.599132782	0.009345961	0.043049369
Gpalpp1	387.0057791	0.214611453	0.107621895	1.994124461	0.046138455	0.149967466
Gpat3	9.309447727	1.365291198	0.367536244	3.714711728	0.000203435	0.001694204
Gpatch11	227.3216893	0.265814707	0.120424999	2.207305038	0.027292756	0.10130655
Gpatch4	261.9613439	0.282568402	0.132723473	2.129000956	0.033254181	0.118005557
Gpc1	327.1249261	-1.358257263	0.138441292	-9.811070419	1.01E-22	1.17E-20
Gpcpd1	421.4965644	-0.2754836	0.119280759	-2.309539291	0.020913674	0.082203118
Gpd1l	279.5022064	0.226711452	0.110233054	2.056655815	0.039719345	0.134279841
Gpi1	1178.854778	0.218803841	0.081293147	2.691541044	0.007112275	0.034881494
Gpm6a	9.983109107	-0.77722584	0.351030746	-2.214124684	0.026820203	0.100047424
Gpnmb	31557.83894	-0.673718498	0.132005828	-5.103702692	3.33E-07	5.16E-06
Gpr108	383.3637246	-0.262327424	0.099526729	-2.635748474	0.008395194	0.03971837
Gpr137b	219.3795585	-0.500608009	0.139697835	-3.583505861	0.000339013	0.002633599
Gpr137b-ps	91.73795116	-0.4024078	0.173160585	-2.323899518	0.02013088	0.079773995
Gpr146	469.763744	-0.251908231	0.115532049	-2.180418616	0.029226445	0.107054251
Gpr155	74.53503223	-0.896067932	0.201009975	-4.457828191	8.28E-06	9.85E-05
Gpr157	115.0605884	-1.079941558	0.171887009	-6.282857341	3.32E-10	8.41E-09
Gpr160	83.26059797	0.595971585	0.195199986	3.053133343	0.002264653	0.013348643
Gpr162	11.03124208	-0.740782894	0.342024573	-2.165876235	0.030320646	0.110048153
Gpr171	16.97744199	0.997218832	0.353568537	2.820439963	0.004795785	0.02524655
Gpr176	26.84474352	-1.572035557	0.286596285	-5.485191683	4.13E-08	7.49E-07
Gpr18	39.58667294	-0.737077655	0.238322603	-3.092772762	0.001982959	0.011946314
Gpr183	520.4382305	1.162783683	0.135873954	8.557811448	1.15E-17	7.78E-16
Gpr34	234.8041035	1.424023244	0.130374298	10.92257654	8.99E-28	1.61E-25
Gpr35	167.2351936	-0.468201119	0.230278163	-2.033198077	0.042032515	0.139739025
Gpr39	28.33633214	-1.411906943	0.260343428	-5.42324788	5.85E-08	1.03E-06
Gpr65	1231.408446	-0.420122788	0.104562037	-4.017928487	5.87E-05	0.00056444
Gpr68	60.92521007	-0.840344065	0.228857861	-3.671903865	0.00024075	0.001964277
Gpr82	24.63045042	0.624046273	0.31752795	1.965327059	0.049376396	0.156823613
Gpr84	60.36291038	2.415753246	0.254419028	9.495175194	2.20E-21	2.18E-19

Gprc5b	115.1246426	-0.559695199	0.19933184	-2.807856484	0.004987245	0.026123414
Gprc5c	20.44603972	-0.817653272	0.288364191	-2.835488236	0.00457557	0.024315007
Gprin3	7.489353134	-0.821485745	0.367769401	-2.233697916	0.025502956	0.096282481
Gpsm3	867.4299983	-0.19140688	0.081998825	-2.334263695	0.019581919	0.077969183
Gpx1	4692.778834	-0.817002006	0.065055494	-12.55853967	3.57E-36	1.28E-33
Gpx3	247.0665478	-0.831415978	0.155772169	-5.337384602	9.43E-08	1.61E-06
Gpx8	34.71632697	-0.936738778	0.275284244	-3.402805642	0.000666977	0.00470363
Gramd1b	404.8912368	0.779227523	0.118984005	6.549010709	5.79E-11	1.60E-09
Gramd2	1.512065737	0.663360981	0.33384063	1.987058859	0.046915875	
Grap	161.081756	0.878166685	0.145802644	6.022981896	1.71E-09	3.89E-08
Grb10	168.3578679	-0.734157756	0.154155209	-4.762458293	1.91E-06	2.59E-05
Grb14	3.045366411	0.973793681	0.375629012	2.59243469	0.009529927	0.04371333
Grb2	3940.657536	-0.382462094	0.079344863	-4.820250238	1.43E-06	2.00E-05
Greb1l	2.387465513	-0.995278318	0.355345861	-2.800872127	0.005096471	0.026602692
Grem1	24.30186628	-1.714096892	0.315404556	-5.434597748	5.49E-08	9.75E-07
Grem2	7.368167105	-1.000929479	0.377606352	-2.650722038	0.008031991	0.038446202
Grhpr	46.6771825	0.613482874	0.222927865	2.751934457	0.005924438	0.030138317
Grina	1089.47333	-0.317029448	0.095622142	-3.315439733	0.000914991	0.006179687
Gripap1	356.9613457	-0.350377913	0.102700673	-3.411641854	0.000645729	0.004570969
Grk2	1180.016678	0.255002924	0.079928966	3.190369374	0.001420911	0.0090011
Grk3	35.51555778	-0.860622189	0.269618922	-3.191994774	0.001412939	0.008958164
Grk5	201.2375057	-0.373972371	0.12150239	-3.077901347	0.002084639	0.012488694
Grk6	356.2496415	0.236655732	0.115880096	2.042246594	0.041127077	0.137612941
Grn	11827.31749	0.593273512	0.097437807	6.088740399	1.14E-09	2.68E-08
Gsap	147.057806	-0.515924823	0.151208954	-3.411999157	0.000644883	0.004569292
Gsdmd	346.485119	-0.258721208	0.105523086	-2.451797203	0.014214475	0.060250559
Gsg1	18.04648195	0.954897765	0.307766787	3.10266671	0.001917855	0.011600665
Gskip	300.9843313	-0.281983394	0.108290182	-2.603960843	0.009215328	0.04260457
Gsta3	314.7936682	-0.658364845	0.283741719	-2.320296248	0.020324857	0.080415272
Gstcd	92.470925	0.537362753	0.194147507	2.767806607	0.005643493	0.028965834
Gstm1	1804.97444	-0.796453225	0.091261619	-8.727143251	2.61E-18	1.91E-16
Gstm5	35.55967053	1.014767948	0.242982507	4.176300436	2.96E-05	0.000307922
Gstt3	101.6240892	0.399554781	0.170190351	2.347693503	0.018890058	0.075716641
Gtf2e2	228.3321802	0.662555268	0.123015161	5.385964321	7.21E-08	1.25E-06
Gtf2h1	550.8413081	0.465189093	0.112824732	4.123112778	3.74E-05	0.000378503
Gtf2h5	803.3717058	0.388527873	0.096798604	4.013775593	5.98E-05	0.000573734
Gtf2i	917.3531129	0.188170334	0.075343302	2.497505804	0.012507042	0.05459371
Gtf3c2	595.7594531	-0.191649224	0.094168665	-2.035169815	0.041833789	0.139418093
Gtpbp6	123.280464	-0.417638774	0.168792953	-2.474266649	0.013351001	0.057425758
Gtse1	40.77402432	1.492927553	0.263820361	5.6588792	1.52E-08	2.99E-07
Gulp1	8.397297959	-0.876377881	0.35988974	-2.435128827	0.014886489	0.062594994
Gusb	2340.35619	0.226296934	0.08623069	2.624320098	0.008682215	0.040729601
Gxylt1	579.5453777	0.394647407	0.102059537	3.866835177	0.000110257	0.000987247
Gyg	1044.540876	-0.353259794	0.109478072	-3.226763031	0.001251991	0.008088088

Gys1	60.58431719	0.633530051	0.196922832	3.217148793	0.001294714	0.008321104
H13	719.2279539	-0.206124731	0.076545823	-2.692827932	0.007084882	0.034807309
H19	28.60076847	-0.756545464	0.27909298	-2.710729107	0.006713545	0.033305841
H1fx	6.554883395	-0.799627008	0.377996187	-2.115436707	0.034392744	0.121129291
H2-Aa	3013.617188	-1.755576974	0.257606984	-6.814943238	9.43E-12	2.89E-10
H2-Ab1	2659.054609	-1.83182116	0.245516504	-7.461091735	8.58E-14	3.40E-12
H2afj	506.7564099	-0.455044663	0.09874101	-4.608466746	4.06E-06	5.17E-05
H2afv	510.2119505	0.581824939	0.13197112	4.40872928	1.04E-05	0.000121338
H2afx	216.1923248	0.515346706	0.182682702	2.820993445	0.004787518	0.025226926
H2afy	1494.156132	0.176762495	0.076993546	2.295809249	0.02168679	0.084709439
H2-D1	11427.88999	-0.801728728	0.094259645	-8.50553522	1.81E-17	1.17E-15
H2-DMa	801.0507371	-0.844868386	0.146989251	-5.747824299	9.04E-09	1.83E-07
H2-DMb1	366.1382076	-0.699487175	0.160536473	-4.357185392	1.32E-05	0.000150579
H2-Eb1	1848.424445	-1.738011016	0.256091981	-6.786667074	1.15E-11	3.46E-10
H2-K1	7122.543043	-0.503437242	0.128756745	-3.909987325	9.23E-05	0.000842367
H2-K2	12.40383824	-1.079715511	0.348188552	-3.100950635	0.001929004	0.011649324
H2-M2	131.4791083	-1.716006559	0.235299594	-7.292858147	3.03E-13	1.12E-11
H2-Q4	75.99627479	-0.759274186	0.225856182	-3.361759584	0.000774475	0.005346159
H2-T22	228.8833209	-0.473543053	0.125801618	-3.764204783	0.00016708	0.001417393
H2-T24	31.66264083	-0.618583254	0.292938439	-2.111649315	0.034716539	0.121983401
H3f3a	285.4626087	0.277988471	0.113454168	2.450227035	0.014276616	0.060419961
H60b	3.850934163	1.482116187	0.378631128	3.9144066	9.06E-05	0.00083081
H6pd	336.6332934	-0.490912253	0.126886392	-3.868911766	0.000109322	0.000981219
Hacd2	129.2100976	0.592258721	0.154230292	3.840093367	0.000122988	0.001089536
Hadha	735.8821735	-0.273058612	0.080488594	-3.392513113	0.000692546	0.004854293
Hadhb	201.9090891	-0.280214513	0.125388363	-2.234772883	0.025432263	0.096063924
Hagh	221.9676242	0.421468356	0.126803677	3.323786553	0.000888041	0.006022068
Hal	493.3134703	2.588790341	0.169610889	15.26311407	1.35E-52	1.19E-49
Hand2	49.09612733	-1.289809764	0.214386685	-6.016277397	1.78E-09	4.02E-08
Hand2os1	6.555563758	-0.952261441	0.380997666	-2.499389168	0.012440761	0.054351818
Haspin	35.62002161	0.69506208	0.26171185	2.655829609	0.007911358	0.037984988
Haus1	53.96341609	0.425865602	0.210192749	2.026071805	0.042757427	0.141521991
Haus4	143.6687063	0.832313733	0.169502261	4.910340003	9.09E-07	1.32E-05
Haus6	127.4228875	0.420593957	0.143618194	2.928556234	0.003405402	0.018948785
Haus8	444.2286767	-0.539524543	0.108136001	-4.989314725	6.06E-07	9.05E-06
Havcr2	23.35012193	1.365786514	0.308233753	4.43100894	9.38E-06	0.00011057
Hba-a1	12.01989146	-2.196502309	0.374530703	-5.864678897	4.50E-09	9.48E-08
Hba-a2	8.274328576	-2.311841232	0.380402412	-6.077356918	1.22E-09	2.86E-08
Hbb-bs	62.96252029	-1.227512923	0.362917959	-3.382342741	0.000718704	0.005014223
Hbegf	97.4964367	-0.423244831	0.195864218	-2.160909403	0.030702338	0.111024095
Hcar2	63.87032847	1.442781186	0.259436779	5.561205272	2.68E-08	5.04E-07
Hccs	176.2307894	0.335351736	0.137061854	2.446718224	0.01441635	0.060908265
Hcfc1r1	356.6650861	-0.229306083	0.109645362	-2.091343207	0.036497309	0.126318063
Hck	581.899235	0.437995232	0.104286132	4.199937465	2.67E-05	0.000281172

Hcst	302.6112429	-0.686264687	0.13699562	-5.00939145	5.46E-07	8.20E-06
Hdac1	256.1131531	0.428575618	0.115921091	3.697132383	0.000218049	0.001806878
Hdac2	414.8830516	0.249848193	0.106289247	2.35064413	0.018740948	0.075239542
Hdac5	334.5752735	-0.513998288	0.106840418	-4.810897386	1.50E-06	2.08E-05
Hdac7	333.0265825	-0.245438011	0.117191168	-2.09433881	0.036229809	0.125711156
Hdac9	285.7536123	0.749127368	0.133937031	5.593131051	2.23E-08	4.23E-07
Hdgf	2161.074182	0.29079614	0.084807494	3.428896748	0.00060604	0.004341214
Hdgfl3	46.50230363	-0.752219476	0.21413425	-3.512840548	0.000443343	0.003339988
Hdx	1.50320742	-0.667263594	0.322670404	-2.06794173	0.038645503	
Hebp2	6.987999785	-0.843500161	0.371747695	-2.269012483	0.023267567	0.089670358
Hectd3	230.438397	0.308541075	0.126306282	2.442800705	0.014573783	0.061417792
Hecw2	3.144764452	0.960406138	0.375865827	2.555183444	0.010613185	0.047753395
Heg1	236.815743	-0.348553785	0.126965799	-2.745257279	0.006046348	0.030654574
Hells	189.1805321	1.400256743	0.177140555	7.904777899	2.68E-15	1.32E-13
Herc1	1509.567727	-0.355440508	0.112629374	-3.155841996	0.001600355	0.010006888
Herpud1	520.1915613	0.743163499	0.114920782	6.46674595	1.00E-10	2.70E-09
Hexa	4527.99769	-0.370179309	0.077497281	-4.776674777	1.78E-06	2.43E-05
Hexb	1524.66639	-0.496517188	0.095182776	-5.216460478	1.82E-07	2.95E-06
Hexim1	492.419237	0.413977479	0.108294051	3.822716707	0.000131989	0.001158236
Hexim2	13.45481926	0.752522678	0.326132685	2.307412633	0.021031828	0.082559413
Hey1	25.8685672	-2.33669503	0.325004567	-7.189729836	6.49E-13	2.30E-11
Hfe	844.1823201	0.656557937	0.095681426	6.861916306	6.79E-12	2.14E-10
Hgf	64.05004777	-1.50164144	0.203372984	-7.383682009	1.54E-13	5.91E-12
Hgsnat	6142.945022	0.599111344	0.109627637	5.464966338	4.63E-08	8.34E-07
Hibch	51.94482098	0.729992985	0.218534716	3.340398262	0.000836583	0.005719638
Hic1	23.66200558	-0.565209187	0.277055297	-2.040059127	0.041344437	0.138216967
Higd1a	83.91365212	0.577155549	0.166404438	3.468390366	0.000523586	0.00382917
Hikeshi	309.3124336	0.389858739	0.11376888	3.426760813	0.000610827	0.00437133
Hilpda	685.1262837	0.941255625	0.167756131	5.610856765	2.01E-08	3.83E-07
Hipk2	1086.411694	-0.573574753	0.112064448	-5.118257942	3.08E-07	4.82E-06
Hirip3	110.7420702	0.84072718	0.191999659	4.378795176	1.19E-05	0.000137973
Hist1h1b	20.03737707	1.557473522	0.344111944	4.526066438	6.01E-06	7.36E-05
Hist1h1c	755.8284844	-0.404957223	0.144678249	-2.79901938	0.005125806	0.02669083
Hist1h1d	3.902406883	0.800837348	0.377514226	2.121343497	0.033892906	0.119847042
Hist1h1e	143.8592392	0.668310153	0.236437956	2.826577271	0.004704839	0.024878619
Hist1h2ab	5.40289119	1.099346812	0.380554589	2.888801878	0.003867126	0.021110937
Hist1h2ae	11.63479397	1.106115452	0.340629036	3.247272943	0.001165166	0.007605761
Hist1h2ag	13.39200075	0.953779193	0.360923871	2.642604912	0.008227097	0.039199778
Hist1h2an	3.563597141	0.903820788	0.376232922	2.402290535	0.016292762	0.067227244
Hist1h2bk	6.667461803	0.751161528	0.375601776	1.999888117	0.045512347	0.148414556
Hist1h3a	5.014681391	0.75932804	0.380681248	1.994655748	0.046080439	0.14981134
Hist1h4d	11.83592941	0.855183254	0.346735044	2.466388295	0.013648329	0.058403329
Hist2h2be	143.9608503	-1.121141003	0.14565719	-7.697120901	1.39E-14	6.20E-13
Hist3h2a	478.0245013	-0.241762328	0.091308211	-2.647761088	0.008102677	0.038688154

Hivep3	619.6899683	-0.271977379	0.107278604	-2.53524345	0.01123692	0.050054159
Hjurp	240.5313494	0.695873587	0.134320999	5.180676088	2.21E-07	3.54E-06
Hk1	512.6746023	0.231073194	0.091399338	2.528171424	0.011465835	0.050777532
Hk2	616.3564981	0.447952196	0.099025224	4.523617108	6.08E-06	7.44E-05
Hk3	772.4388329	-0.577289328	0.114363943	-5.047826369	4.47E-07	6.81E-06
Hlcs	117.9782773	-0.556341219	0.146429038	-3.799391348	0.000145052	0.001254636
Hmbs	113.7977314	0.468318466	0.154243236	3.036233403	0.002395538	0.014015534
Hmg20b	452.6377143	-0.262478198	0.117109253	-2.241310489	0.02500597	0.094931594
Hmga2	259.3265393	0.516242524	0.19348365	2.668145471	0.007627123	0.036934569
Hmgb1-ps5	17.31962426	-1.106827173	0.307782779	-3.59613094	0.000322985	0.002529818
Hmgb2	134.8338284	0.988475526	0.160288678	6.166845583	6.97E-10	1.68E-08
Hmgb3	41.81473749	1.167716039	0.274815835	4.249085709	2.15E-05	0.000231573
Hmgcl	394.392679	-0.313450973	0.112617954	-2.783312633	0.005380693	0.027824969
Hmgn5	220.4978082	0.620788877	0.157023722	3.95347193	7.70E-05	0.000717962
Hmmr	183.5460111	1.823164664	0.219327452	8.312523808	9.37E-17	5.54E-15
Hmox1	8932.915448	0.662093071	0.22077424	2.998959797	0.002709031	0.015594331
Hmox2	1621.712345	0.19793124	0.079919528	2.476631735	0.013262867	0.057194208
Hnmt	201.4836969	0.269986449	0.125598819	2.14959385	0.031587355	0.113622109
Hnrnpa2b1	2604.009628	0.376889059	0.09644098	3.907976262	9.31E-05	0.00084805
Hnrnpab	2606.953372	0.4506107	0.088409344	5.096867362	3.45E-07	5.33E-06
Hnrnpd	599.9238553	0.304717862	0.091863301	3.317079376	0.000909638	0.00615184
Hnrnph3	282.465826	-0.243378907	0.102771626	-2.368152743	0.017877154	0.072430466
Hnrnpm	1982.630735	0.330060282	0.089363781	3.693445805	0.000221236	0.001826229
Hnrnpu	2009.466401	0.189890124	0.076195058	2.492158022	0.012696953	0.055197908
Hoga1	10.46425585	0.941293069	0.363928701	2.5864766	0.009696275	0.044336382
Hook3	1616.507697	0.257654115	0.10778526	2.390439228	0.016828234	0.069038083
Hormad2	3.631320683	1.358388889	0.367434542	3.696954785	0.000218201	0.001807144
Hoxc8	13.21470768	-0.998035809	0.338564408	-2.94784622	0.003199962	0.017999187
Hoxc9	4.508645689	-0.766484623	0.380930895	-2.012135619	0.044205643	0.144877502
Hoxd8	4.880684502	-0.817409309	0.379659753	-2.153004903	0.031318293	0.112870707
Hp	507.8633837	-1.526428632	0.34749064	-4.392718698	1.12E-05	0.000129924
Hpcal1	1046.052044	-0.374431848	0.091620439	-4.086772036	4.37E-05	0.000435011
Hpgd	148.6243242	0.662299249	0.225956774	2.931088274	0.003377768	0.018829927
Hpgds	1246.812221	0.37230094	0.100414549	3.707639426	0.0002092	0.001736073
Hr	18.54030689	-1.220790025	0.31646904	-3.857533823	0.000114537	0.001020205
Hras	152.5803761	0.419362705	0.172505875	2.431005343	0.015056993	0.063170335
Hs3st3a1	21.9201375	-1.828700685	0.32383711	-5.646976916	1.63E-08	3.17E-07
Hs6st1	494.7657727	-0.284386074	0.114032277	-2.493908589	0.012634508	0.054990156
Hs6st2	39.52217855	-0.904784395	0.235903293	-3.835403843	0.000125358	0.001105967
Hsd11b1	26.93960482	0.611792777	0.287271239	2.129669436	0.033198914	0.1178373
Hsd17b11	1184.601615	-0.261884121	0.089356867	-2.930766592	0.003381267	0.018835441
Hsd17b12	1256.482208	0.522909676	0.092491512	5.65359635	1.57E-08	3.07E-07
Hsd17b4	535.7375908	0.432925598	0.091270513	4.743323808	2.10E-06	2.83E-05
Hsd17b7	182.5593267	0.462243721	0.150629103	3.068754397	0.002149532	0.012822237

Hsdl2	194.9783067	-0.289113253	0.122454585	-2.360983488	0.018226542	0.073567972
Hsp90b1	5176.101246	0.292749648	0.080474988	3.637771874	0.000275007	0.002211698
Hspa5	7503.303837	0.407152365	0.078650319	5.176741447	2.26E-07	3.61E-06
Hspa9	1553.042861	0.187126246	0.090225558	2.07398271	0.038080917	0.130059244
Hspb1	34.68942103	-0.933442526	0.304818724	-3.062287363	0.002196525	0.013034103
Hspb7	6.295117507	-0.850157391	0.380136112	-2.236455219	0.025321968	0.095791975
Hspbp1	276.9248421	0.263715386	0.133350827	1.97760592	0.047973181	0.153963543
Hspg2	61.35455347	-2.16191842	0.236680904	-9.134317071	6.58E-20	5.58E-18
Hsph1	305.5731611	-0.30201633	0.130734504	-2.310150114	0.020879845	0.082135137
Htr1b	4.49740189	-0.923786723	0.380977622	-2.424779488	0.015317688	0.064013517
Htra1	8.974753212	-0.725099261	0.349489338	-2.074739288	0.038010704	0.12987856
Htra2	326.3261038	-0.324714175	0.123583113	-2.627496319	0.008601577	0.040490549
Htt	224.0663334	-0.240756899	0.117301494	-2.052462339	0.040124751	0.135284688
Hus1	71.06858295	0.536013491	0.183338569	2.92362646	0.003459797	0.019201615
Hvcn1	797.5553829	-0.818543583	0.103228039	-7.92946946	2.20E-15	1.11E-13
Hyal1	58.56334634	0.461087532	0.218830986	2.107048643	0.035113363	0.122945927
Hyal2	125.8445163	0.465911544	0.144076258	3.233784317	0.001221617	0.007932844
Hyi	345.6283385	-0.761165702	0.123400015	-6.168278865	6.90E-10	1.67E-08
I830077J02Rik	57.81477502	0.830669002	0.206540245	4.021826355	5.77E-05	0.000556962
Iah1	284.240426	-0.753701382	0.108194553	-6.966167542	3.26E-12	1.09E-10
Iars	422.0392065	0.347502357	0.108446885	3.204355355	0.001353653	0.008618697
Ica1	17.0964767	0.998899737	0.322119456	3.101022672	0.001928535	0.011649324
Icosl	33.69385929	1.148110174	0.262512108	4.373551312	1.22E-05	0.000141114
Id1	312.4299531	1.251928015	0.190998818	6.554637514	5.58E-11	1.55E-09
Id2	3756.184907	-0.654974678	0.108390684	-6.042721136	1.52E-09	3.48E-08
Idh1	3364.435848	-0.242885448	0.093887869	-2.586973709	0.009682298	0.044285963
Idh2	729.9224458	0.259493596	0.096574305	2.686983824	0.007210044	0.035278072
Idh3a	245.4955797	0.417542648	0.135087149	3.090913174	0.00199542	0.012011738
Idh3g	622.3575327	-0.22759935	0.086302641	-2.637223454	0.008358775	0.039609024
Idnk	183.1105867	-0.466889164	0.133272102	-3.503277544	0.00045957	0.003441503
Ier3	1101.518738	0.943080937	0.14169121	6.655888811	2.82E-11	8.16E-10
Ier5	1591.778888	-0.262891519	0.099369186	-2.64560402	0.008154521	0.038910937
Ier5l	87.37181763	-1.098028995	0.204281135	-5.375087608	7.65E-08	1.32E-06
Iffo2	187.1724039	0.482854229	0.135946089	3.551806689	0.000382596	0.002927823
Ifi202b	6.557897566	1.995404235	0.380992194	5.237388758	1.63E-07	2.67E-06
Ifi203-ps	17.02621506	0.768728619	0.314583224	2.44364149	0.014539867	0.061309296
Ifi27	1319.249413	0.694322872	0.084797771	8.187984936	2.66E-16	1.48E-14
Ifi27l2a	196.7258594	1.863783201	0.144454977	12.90217371	4.38E-38	1.77E-35
Ifit1	25.44420494	0.678993446	0.305961877	2.21920931	0.026472488	0.099045781
Ifitm10	7.847166863	-0.765346126	0.371735533	-2.05884576	0.039509016	0.133809932
Ifitm2	2060.196415	0.509386206	0.124413571	4.094297775	4.23E-05	0.000423083
Ifitm3	3714.608435	1.327590837	0.226270974	5.867260886	4.43E-09	9.35E-08
Ifitm6	55.69801285	2.380583327	0.293572518	8.109012866	5.10E-16	2.75E-14
Ift172	77.28538608	-0.650245995	0.18156238	-3.581391673	0.000341769	0.002649238

Ift43	127.8974199	-0.45067485	0.152694605	-2.951478531	0.003162565	0.017815547
Igf1r	551.0243127	-0.532351612	0.14622699	-3.640583798	0.000272021	0.002194738
Igf2bp1	12.17288755	1.547379546	0.361839004	4.276431032	1.90E-05	0.000208488
Igf2bp2	237.7237635	-0.991371629	0.199491048	-4.969504329	6.71E-07	9.93E-06
Igf2bp3	7.364966574	1.271113626	0.37853941	3.357942643	0.000785249	0.005414594
Igf2r	1170.315577	-0.531343706	0.115713931	-4.591873246	4.39E-06	5.54E-05
Igfbp2	1.920657763	-0.850852663	0.341778368	-2.489486586	0.012792775	
Igfbp4	4208.235004	2.133975544	0.145516101	14.66487573	1.08E-48	6.50E-46
Igfbp7	189.439899	-1.769679595	0.154585492	-11.44790222	2.41E-30	5.74E-28
Igsf3	53.98283111	-0.657238164	0.294966345	-2.228180178	0.025868502	0.097393029
Igsf6	2271.086094	-0.300166224	0.085256078	-3.520760402	0.000430311	0.003259809
Igtp	177.5593621	-0.468794529	0.227898014	-2.057036478	0.039682717	0.134216483
Ikbip	288.0772623	0.227229085	0.107577559	2.112235007	0.034666297	0.121835392
Ikbkb	724.1495862	-0.209539368	0.079764537	-2.626974026	0.008614791	0.040501932
Ikbkg	397.4932974	-0.376325805	0.141431492	-2.660834579	0.007794724	0.037552304
Ikzf2	122.7146791	-0.489881498	0.170815907	-2.867891565	0.004132171	0.022330391
Il10ra	385.1146358	-0.318222798	0.114419808	-2.781186258	0.005416065	0.027978965
Il11ra1	980.6945059	-1.230718618	0.112119079	-10.97688836	4.94E-28	9.15E-26
Il16	275.5716415	0.500032613	0.121756413	4.106827734	4.01E-05	0.000404011
Il17ra	581.1517055	0.836853097	0.099523129	8.408629255	4.15E-17	2.58E-15
Il17rc	4.66074586	-0.849892653	0.381259015	-2.229173923	0.025802335	0.097226104
Il18	167.0564458	0.67624179	0.18872901	3.58313643	0.000339493	0.00263432
Il18bp	194.7094621	-1.049377833	0.305155342	-3.438831601	0.000584231	0.004211118
Il1r1	151.6601548	-1.073039911	0.152829654	-7.021149929	2.20E-12	7.42E-11
Il21r	283.9606451	1.236563463	0.167250821	7.393467242	1.43E-13	5.55E-12
Il27ra	4.870331007	-0.75601303	0.379614699	-1.991527281	0.046422949	0.150641851
Il2rg	595.7690646	-0.446988611	0.119551412	-3.73888191	0.000184841	0.001550532
Il33	8.075402965	1.075437011	0.37791415	2.845717771	0.004431145	0.023732402
Il3ra	137.3984522	-0.89783368	0.180569966	-4.972220453	6.62E-07	9.82E-06
Il4i1	38.34601806	-0.810046761	0.231467003	-3.499620907	0.00046592	0.003478639
Il4ra	718.3489315	0.957925133	0.112260488	8.533056951	1.43E-17	9.46E-16
Il6st	1078.614318	-0.664712517	0.102589811	-6.479322944	9.21E-11	2.50E-09
Il7	20.38759832	-0.746476215	0.282902591	-2.638633362	0.008324095	0.039517284
Il7r	3139.178316	-1.199547944	0.215906837	-5.555858992	2.76E-08	5.17E-07
Ilk	537.0812184	-0.369498619	0.095693529	-3.861270682	0.000112799	0.001007003
Ilrun	867.4582584	-0.159176748	0.078836398	-2.019076877	0.04347923	0.143279052
Ilvbl	274.3460337	-0.457148492	0.122099696	-3.744059222	0.000181071	0.001524022
Imp4	532.4906779	0.168764952	0.083120531	2.030364225	0.04231953	0.140444315
Impa1	532.5402592	0.294648191	0.100387695	2.935102654	0.003334374	0.018643422
Impad1	1727.203872	0.272598917	0.07237225	3.766622108	0.000165471	0.001404694
Impdh1	267.739404	0.774929313	0.117094343	6.617991058	3.64E-11	1.04E-09
Inafm1	130.2412233	-0.601801058	0.158796852	-3.789754326	0.000150796	0.001296849
Incenp	541.8108047	0.84684044	0.153625907	5.512354355	3.54E-08	6.51E-07
Inf2	134.2615112	-0.639957982	0.183205324	-3.49311891	0.000477414	0.003546807

Ing4	51.0062255	-0.525860529	0.238454629	-2.205285466	0.027434069	0.101622184
Inhba	48.26658452	-1.619287838	0.239770983	-6.75347709	1.44E-11	4.29E-10
Inhbb	2.125323817	-1.08450477	0.347844303	-3.117787935	0.001822139	0.011120307
Inka1	63.07735087	0.876957889	0.185927088	4.716676295	2.40E-06	3.18E-05
Inpp4b	94.14137117	2.194511001	0.263111154	8.34062322	7.39E-17	4.45E-15
Inpp5a	172.302204	-0.462504908	0.128180557	-3.608229808	0.000308293	0.002436445
Inpp5d	1261.468687	0.413214032	0.0852261	4.84844466	1.24E-06	1.76E-05
Inpp5f	355.8870692	-0.645084948	0.112277019	-5.74547625	9.17E-09	1.85E-07
Inppl1	1261.850141	-0.332204636	0.076056782	-4.367850264	1.25E-05	0.000144624
Insig1	254.356721	-0.289458206	0.139925873	-2.068653919	0.038578574	0.13142989
Insl6	65.55307915	-0.491181641	0.230918657	-2.127076472	0.03341373	0.118487676
Insr	309.1191416	-0.41744051	0.115179131	-3.624272102	0.000289776	0.002313576
Ints10	54.26097698	0.415957689	0.210416819	1.976827191	0.048061167	0.153966418
Ints6l	255.7003886	0.398880365	0.117947894	3.381835424	0.000720033	0.005021157
Ints7	167.1348212	0.442254135	0.150758939	2.933518483	0.003351437	0.018710943
Ints8	78.81194229	0.361991799	0.183890901	1.968513919	0.049008936	0.155986661
Invs	83.25703514	-0.343389119	0.163811267	-2.096248483	0.036060154	0.125354351
Ip6k1	534.4204359	-0.31164194	0.091075081	-3.421813493	0.00062205	0.004434727
Ipcef1	44.99712761	-1.618185542	0.247918228	-6.52709386	6.71E-11	1.84E-09
Ipmk	392.8687072	0.304558351	0.096878885	3.143702069	0.001668252	0.010349507
Ipo11	236.7517961	0.362257714	0.112184562	3.22912268	0.001241706	0.008034912
Ipo5	614.0435623	-0.254405981	0.109949811	-2.313837365	0.020676645	0.081484877
Ipo9	195.3160774	0.357795049	0.125729238	2.845758506	0.004430578	0.023732402
Ippk	60.24664897	0.424953239	0.193872686	2.19191908	0.02838535	0.104381022
Iqgap1	5982.55505	-0.268128207	0.082120289	-3.265066513	0.001094384	0.007203253
Iqgap3	103.303505	1.758477408	0.196798724	8.935410612	4.06E-19	3.22E-17
Iqsec1	501.3467117	-0.654770578	0.123992702	-5.280718691	1.29E-07	2.15E-06
Iqsec2	282.5710288	-0.262231167	0.115156542	-2.277171256	0.022776001	0.088092641
Irak2	137.1384126	-0.429615654	0.13962754	-3.07686903	0.002091872	0.012517034
Irf1	1247.170425	-1.01489279	0.274867278	-3.692301228	0.000222234	0.001832454
Irf2	1595.950637	-0.346289364	0.122454039	-2.827912958	0.004685254	0.024817951
Irf5	865.5104099	-0.418861226	0.099074504	-4.227739806	2.36E-05	0.000251167
Irf8	2696.64651	-0.439645829	0.089364407	-4.919697257	8.67E-07	1.26E-05
Irf9	476.0993891	0.39552516	0.144508624	2.73703498	0.00619957	0.031304491
Irs2	651.0902033	-0.375274682	0.110777039	-3.387657643	0.000704922	0.004931825
Isca1	190.8373083	0.316857226	0.121142885	2.615566117	0.008907967	0.04154191
Isca2	255.1909429	0.313497846	0.120304398	2.605871873	0.009164073	0.042388335
Itch	590.2920358	-0.34760931	0.127315767	-2.730292702	0.006327812	0.031759688
Itga11	1.458264335	-0.624066152	0.294585715	-2.118453546	0.034136675	
Itga3	24.46605638	-0.561133607	0.273182355	-2.054062415	0.039969651	0.134882967
Itga4	2049.661337	0.313897808	0.083134176	3.775797446	0.000159497	0.00135998
Itga5	44.33583121	-0.840426084	0.268822433	-3.126324221	0.001770063	0.010873243
Itga6	7421.604163	0.450020005	0.087653082	5.134103596	2.83E-07	4.45E-06
Itga8	4.667951977	1.598773785	0.375333856	4.25960451	2.05E-05	0.000222378

Itga9	319.3889419	0.640206926	0.137743008	4.647836115	3.35E-06	4.32E-05
Itgam	10506.48218	0.594763628	0.12600584	4.720127486	2.36E-06	3.13E-05
Itgax	1447.266344	-1.722692556	0.237723329	-7.246628094	4.27E-13	1.56E-11
Itgb1bp1	272.7507331	0.27143977	0.120806533	2.246896443	0.024646646	0.093828569
Itgb2	7634.401401	-0.425394889	0.109408702	-3.88812663	0.000101021	0.000913266
Itgb3bp	44.59345312	0.58973171	0.239438187	2.462981016	0.013778721	0.058849025
Itgb5	713.6260213	-0.971758089	0.103193799	-9.416826413	4.65E-21	4.50E-19
Itm2b	25653.40567	-0.157920454	0.065096278	-2.425952121	0.015268284	0.063860408
Itpa	241.0711526	0.438251495	0.131887085	3.322929571	0.000890774	0.006032421
Itpk1	92.43378019	-0.343561022	0.170007601	-2.020856836	0.043294587	0.142827406
Itpkb	778.6065451	-0.673245305	0.096791196	-6.955646092	3.51E-12	1.16E-10
Itpr2	672.6255926	-0.248749867	0.102901551	-2.417357799	0.015633641	0.06500805
Itpr3	45.73330031	0.465330148	0.223576455	2.081302111	0.037406262	0.128515516
Itsn1	3510.175323	0.570894862	0.09173524	6.223288452	4.87E-10	1.20E-08
Ivl	7.190310137	-1.461119785	0.371705496	-3.930853325	8.46E-05	0.000780741
Jag1	583.2455521	-0.467699021	0.15412688	-3.034506501	0.002409296	0.014079556
Jak2	360.9854171	-0.451554096	0.105270258	-4.289474587	1.79E-05	0.000197625
Jam2	68.20610186	1.748948661	0.21480265	8.142118635	3.88E-16	2.12E-14
Jam3	10.62666365	-0.825778225	0.350221323	-2.35787535	0.018379863	0.074047412
Jarid2	415.3789236	0.473417451	0.113478409	4.171872489	3.02E-05	0.000313102
Jdp2	164.1824215	0.915190825	0.146438654	6.249653362	4.11E-10	1.03E-08
Jmjd1c	1182.311468	-0.213961158	0.103835482	-2.060578467	0.039343273	0.133572446
Jmjd7	13.2554048	0.771451367	0.333405667	2.313851992	0.020675842	0.081484877
Jmy	246.9003475	-0.274167045	0.131000669	-2.092867515	0.036360983	0.126020616
Jpt1	371.3460738	0.318437546	0.11049726	2.881859206	0.003953363	0.021482051
Jpt2	229.9549124	1.00566535	0.160968723	6.247582347	4.17E-10	1.04E-08
Jund	719.0236077	-0.383928138	0.095287373	-4.029160704	5.60E-05	0.00054301
Jup	113.9711418	-0.551360323	0.158765995	-3.472785999	0.000515086	0.00377068
Kank2	347.8312181	0.959818891	0.097197915	9.874891787	5.35E-23	6.47E-21
Kansl3	449.2275844	-0.20704786	0.098964833	-2.092135704	0.036426378	0.126159856
Kat2a	104.6410205	0.321083259	0.157029609	2.044730677	0.040881418	0.137219287
Kat5	122.3898563	-0.332454112	0.154667574	-2.149475185	0.031596751	0.113624445
Kbtbd7	93.41716688	-0.424603124	0.165692458	-2.562597769	0.010389233	0.046862405
Kcnab1	14.24325149	-1.43102779	0.339195735	-4.218884977	2.46E-05	0.000259832
Kcnab2	428.3127533	0.386445423	0.121395883	3.183348684	0.001455822	0.009198955
Kcnb1	8.947557653	-0.769065983	0.367858653	-2.090656226	0.036558892	0.126443717
Kcne3	27.10342435	-1.267288893	0.260263062	-4.86926144	1.12E-06	1.60E-05
Kcng2	7.391618058	-0.966815032	0.366189533	-2.640203896	0.008285616	0.039398682
Kcnj16	3.72648323	-1.092256009	0.37965864	-2.876942322	0.00401549	0.021778264
Kcnj2	263.9083677	-1.344348876	0.329913743	-4.074849575	4.60E-05	0.000456095
Kcnn1	3.312410658	0.778353645	0.371024151	2.097851699	0.035918248	0.125034829
Kcnn4	335.4753922	-0.573059019	0.138554052	-4.135996103	3.53E-05	0.000359089
Kcnq1	26.83886493	-1.153091508	0.264737976	-4.355595396	1.33E-05	0.000151454
Kctd11	163.4335026	-0.459041132	0.125126215	-3.668624759	0.000243859	0.001986747

Kctd12	2073.777278	0.414171087	0.12264045	3.377116491	0.0007325	0.005100989
Kctd15	14.49821039	-0.822744791	0.331391341	-2.482698515	0.013039139	0.056407715
Kctd4	3.118033497	0.819013633	0.377232084	2.171113405	0.029922601	0.109018809
Kdelr3	37.66864831	-1.092329216	0.257677282	-4.239136678	2.24E-05	0.000240349
Kdm2a	854.6226271	-0.212312298	0.095932298	-2.21314721	0.026887498	0.100223717
Kdm5a	1076.523179	-0.175897654	0.088178904	-1.994781589	0.046066706	0.14981134
Kdm7a	1393.646578	-0.450410913	0.107250944	-4.199598596	2.67E-05	0.000281396
Kdsr	239.4878685	0.48391529	0.120872116	4.003531236	6.24E-05	0.000595735
Khdrbs3	8.466286199	-0.88369117	0.36156745	-2.444056204	0.014523164	0.061256076
Kif11	109.3272797	1.821165632	0.232146509	7.844897779	4.33E-15	2.08E-13
Kif14	48.67045581	1.963068084	0.278326785	7.053105171	1.75E-12	5.94E-11
Kif15	139.3806323	2.03784481	0.23592218	8.637783894	5.73E-18	4.02E-16
Kif16b	224.6233266	0.253214235	0.128239961	1.97453456	0.048320992	0.154287898
Kif18a	40.66539693	1.360580597	0.260547197	5.222012028	1.77E-07	2.87E-06
Kif18b	21.10143169	1.667196021	0.318990589	5.226473999	1.73E-07	2.81E-06
Kif1b	1151.631454	-0.24008386	0.102000546	-2.353750744	0.018585069	0.074733688
Kif20a	102.0860068	1.634152714	0.237078946	6.892863086	5.47E-12	1.75E-10
Kif20b	218.9803978	1.39394566	0.193416243	7.206973108	5.72E-13	2.03E-11
Kif22	74.98295226	1.571667138	0.254047935	6.186498381	6.15E-10	1.49E-08
Kif23	276.6854223	2.131939868	0.201968324	10.55581304	4.77E-26	7.96E-24
Kif26b	23.93979498	-0.935440496	0.302956989	-3.087700661	0.002017115	0.012132606
Kif2c	52.70163601	1.579067598	0.281462117	5.610231365	2.02E-08	3.84E-07
Kif4	37.54924121	1.586383647	0.288970782	5.489771788	4.02E-08	7.32E-07
Kif5b	2946.365812	0.19298127	0.08970504	2.151286822	0.031453568	0.113276624
Kif5c	29.50646478	-0.619801439	0.249660333	-2.482578756	0.013043523	0.056410418
Kifc1	7.322614857	1.361589392	0.36933247	3.686622493	0.00022725	0.001867306
Kifc2	12.28647629	-0.725444199	0.330399776	-2.195655845	0.028116586	0.103621219
Kifc3	393.4730748	0.595760893	0.098782077	6.031062628	1.63E-09	3.71E-08
Kifc5b	6.092361614	0.985303064	0.375047171	2.627144372	0.008610479	0.040494348
Kitl	477.0588626	-0.94909173	0.135850495	-6.986295703	2.82E-12	9.45E-11
Klc1	831.6188932	0.344482437	0.091886957	3.748980785	0.000177555	0.001496946
Klc2	53.19621119	-0.428439435	0.206400855	-2.075763858	0.037915797	0.129701928
Klc4	647.8744682	-0.414370403	0.105137006	-3.941242171	8.11E-05	0.000753237
Klf13	1298.179163	-0.238273594	0.111773863	-2.131746973	0.033027653	0.117445776
Klf2	1093.159141	1.545787559	0.163398251	9.460245416	3.07E-21	3.01E-19
Klf3	1371.832655	0.406704087	0.12077465	3.367462342	0.000758634	0.005258577
Klf4	135.8770025	0.629127227	0.171602346	3.666192466	0.000246189	0.002003554
Klf6	3699.025158	-0.205861581	0.102263103	-2.013058238	0.044108503	0.144638261
Klf8	28.99715529	-0.984539098	0.272003345	-3.619584521	0.000295076	0.002347711
Klf9	1323.992029	-1.024955091	0.122939544	-8.337065969	7.62E-17	4.57E-15
Klhdc8a	1.582562704	-0.692700233	0.328567476	-2.108243461	0.035009936	
Klhl12	134.5112996	-0.369135971	0.14878132	-2.481063964	0.013099086	0.056569206
Klhl21	339.4113045	-0.368602621	0.10891466	-3.384325126	0.000713535	0.004985094
Klhl24	750.4507613	-0.501541898	0.104235383	-4.811628094	1.50E-06	2.08E-05

Klhl26	88.20767161	-0.415094356	0.172008199	-2.413224244	0.015812088	0.065604645
Klhl5	611.9986127	0.471824641	0.088162283	5.351774325	8.71E-08	1.49E-06
Klra2	156.7617436	2.009889646	0.305355217	6.582136269	4.64E-11	1.31E-09
Klra4	2.310156927	0.896579202	0.336135924	2.667311458	0.007646078	0.037002479
Klrb1a	22.95557465	-0.918071674	0.299355943	-3.066822948	0.00216347	0.012883805
Klrg2	3.244512022	1.117696887	0.372418199	3.001187615	0.002689288	0.015510433
Kmt2a	1200.182814	-0.493125918	0.137099689	-3.596841997	0.000322104	0.002525504
Kmt2c	1084.372172	-0.295350393	0.10953361	-2.696436213	0.007008582	0.034473218
Kmt2d	399.5056479	-0.278755385	0.125362922	-2.223587169	0.026176233	0.098182135
Kmt5a	1230.894496	0.235285069	0.090206983	2.608280007	0.009099848	0.04220408
Kmt5b	370.5706438	-0.272669193	0.134462661	-2.027843203	0.042576253	0.141077906
Knl1	99.83377655	1.707670809	0.250403275	6.819682413	9.12E-12	2.81E-10
Knstrn	96.05776502	1.671885437	0.227000398	7.365121153	1.77E-13	6.74E-12
Kntc1	34.94446772	2.205726885	0.280655124	7.859207601	3.87E-15	1.87E-13
Kpna1	548.4718016	0.403559123	0.110231081	3.661028456	0.000251205	0.002038849
Kpnb1	901.8483658	0.21667759	0.080642671	2.686885087	0.007212176	0.035278072
Kras	1254.424236	0.840010229	0.085229892	9.855817146	6.47E-23	7.70E-21
Kremen1	107.5009113	0.340364577	0.161025349	2.113732897	0.034538087	0.121470137
Krt222	8.028871013	0.865405642	0.371754933	2.327892831	0.019917795	0.079096679
Ksr2	201.8917516	-1.456809144	0.145205381	-10.03274901	1.09E-23	1.42E-21
Ktn1	750.0718311	-0.203169911	0.1025032	-1.982083598	0.047469885	0.152904178
Kxd1	280.8476978	-0.290945042	0.110126826	-2.641908904	0.008244023	0.039250552
L2hgdh	19.99892478	0.759776093	0.288718784	2.63154368	0.008499794	0.040124696
Lacc1	387.7985811	-0.644706956	0.1198914	-5.377424539	7.56E-08	1.31E-06
Lactb2	178.0285756	0.294889825	0.123414892	2.389418494	0.016875068	0.069205133
Lage3	243.8471769	0.363096885	0.118565109	3.062426109	0.002195507	0.013034103
Lair1	1124.873287	-0.368608	0.093669621	-3.935192621	8.31E-05	0.000769132
Lama3	88.06202628	0.763451495	0.241715201	3.158475312	0.001585967	0.009933478
Lama4	31.66468343	-0.983060366	0.250734062	-3.92072923	8.83E-05	0.000810299
Lamb1	86.32151674	-0.889844513	0.176803018	-5.032971277	4.83E-07	7.32E-06
Lamp2	7434.352309	0.190446919	0.067063176	2.839813577	0.004513991	0.024071757
Lamtor4	1569.664138	-0.283587734	0.075947311	-3.734006225	0.000188458	0.001577349
Large1	305.8421975	-0.814727194	0.119261604	-6.831429106	8.41E-12	2.62E-10
Larp4	460.8690151	0.26841499	0.107459271	2.497829995	0.01249561	0.054559681
Lars2	103.2451333	-0.416226162	0.175053044	-2.377714503	0.017420308	0.070994788
Las1l	161.5303706	0.295970937	0.133389809	2.21884219	0.026497463	0.099114512
Lasp1	2135.922416	-0.299414142	0.118193684	-2.533249933	0.011301034	0.050276903
Lat	15.87363822	-0.894107997	0.31151233	-2.870217035	0.004101901	0.022190784
Lats2	599.6159754	0.298897678	0.083909986	3.562122854	0.000367868	0.002833982
Lbh	330.9584631	0.50089261	0.151339014	3.309738827	0.000933831	0.006287122
Lbp	66.00622308	1.077547594	0.254477625	4.234351037	2.29E-05	0.000245169
Lbr	405.5792807	0.420715333	0.105930658	3.97161068	7.14E-05	0.000671677
Lbx2	14.14066206	0.758749848	0.325870308	2.328379816	0.019891944	0.079035851
Lca5	28.42334429	0.593752035	0.273113734	2.174010172	0.029704371	0.108407952

Lcorl	323.1732334	0.268799381	0.122814921	2.188654111	0.02862199	0.105122418
Lcp1	6393.403175	0.305987289	0.072095618	4.244187075	2.19E-05	0.000235504
Lcp2	913.0699586	0.606624142	0.128450404	4.722633211	2.33E-06	3.09E-05
Ldha	2675.555963	0.599234238	0.101309423	5.914891432	3.32E-09	7.17E-08
Ldhb	151.9112882	-0.897870999	0.15961793	-5.625126197	1.85E-08	3.57E-07
Ldlrad3	368.9475371	-0.384285535	0.115273	-3.333699431	0.000856992	0.005840545
Lefty1	15.77744204	1.220514025	0.326805972	3.734674794	0.000187958	0.001574043
Lemd2	307.8342465	-0.198070666	0.098876381	-2.003215167	0.045154198	0.14737474
Leng9	33.01485958	-0.500057041	0.242512721	-2.061982728	0.03920938	0.133215139
Letmd1	392.5721293	-0.460415233	0.09932563	-4.63541216	3.56E-06	4.56E-05
Lfng	401.0177251	0.420862041	0.129392129	3.252609291	0.001143506	0.007474127
Lgals1	3629.423898	0.811361131	0.123364725	6.576929772	4.80E-11	1.35E-09
Lgals3	24168.22242	-1.17975981	0.116880038	-10.09376652	5.89E-24	8.10E-22
Lgals3bp	1270.398885	0.280771478	0.130334755	2.154233363	0.031221876	0.112604347
Lgals9	850.387889	1.070814064	0.101579462	10.54163944	5.55E-26	9.16E-24
Lgmn	8001.128589	0.372578592	0.09988492	3.730078499	0.00019142	0.001600358
Lgr4	30.30630932	0.543672846	0.253585619	2.143941949	0.032037538	0.114883474
Lhfpl2	561.0452444	-0.622124588	0.148941008	-4.176986556	2.95E-05	0.000307421
Lhpp	51.47865036	0.665067468	0.226035457	2.942314797	0.003257686	0.018269092
Lifr	71.79807549	0.603534294	0.198773805	3.036286863	0.002395114	0.014015534
Lig1	611.9067439	1.435740675	0.154577121	9.288183559	1.57E-20	1.42E-18
Lilra5	27.94537834	0.563529151	0.270035765	2.086868571	0.036900017	0.127330087
Lima1	949.1419207	0.16539077	0.077455919	2.135288979	0.032737413	0.11660946
Limd1	906.7529743	-0.217289436	0.077097623	-2.818367515	0.004826853	0.025380721
Lin54	195.4258294	0.51156826	0.122932686	4.161368932	3.16E-05	0.000325834
Lin9	103.9714898	0.531726072	0.169422762	3.138457114	0.001698398	0.010510456
Lipa	13026.48832	-0.647096726	0.11091586	-5.834122628	5.41E-09	1.13E-07
Lipe	185.998873	-0.58084838	0.151385959	-3.836870892	0.000124612	0.001100678
Lipo3	190.989326	0.284418951	0.137043521	2.075391451	0.03795027	0.129760697
Lipt2	67.69373111	-0.694231347	0.25106684	-2.765125598	0.005690088	0.029133795
Litaf	3477.474324	-0.358094924	0.078920721	-4.537400571	5.70E-06	7.03E-05
Llgl1	131.9637381	0.299888541	0.147129224	2.03826632	0.041523306	0.13872223
Lmbr1l	49.4711463	-0.493089667	0.213961747	-2.304569274	0.021190708	0.082987725
Lmln	39.35759805	0.549339596	0.22569027	2.434042003	0.014931262	0.062727196
Lmna	803.0943162	-0.257906948	0.130173161	-1.981260547	0.047562062	0.153135351
Lmnb1	363.1788178	1.48499117	0.182999788	8.114715231	4.87E-16	2.64E-14
Lmnb2	141.781112	0.926198743	0.1941816	4.769755446	1.84E-06	2.51E-05
Lmo2	1225.56117	0.197844336	0.077643933	2.548097817	0.01083121	0.048579919
Lmo4	900.4079976	-0.498370684	0.09278765	-5.371088515	7.83E-08	1.35E-06
Lmod1	3.131788909	-0.918927911	0.351558045	-2.613872515	0.008952243	0.041680181
Lockd	38.09099501	1.763200804	0.279637534	6.305308083	2.88E-10	7.37E-09
Lonp2	450.5809133	-0.240468273	0.090086689	-2.669298621	0.007600984	0.036843658
Lonrf1	232.2467879	0.680494814	0.129277933	5.263812598	1.41E-07	2.34E-06
Lox	117.0794413	-0.806500777	0.163249013	-4.940310283	7.80E-07	1.15E-05

Loxl1	24.92247877	-0.597522372	0.284504654	-2.100220026	0.035709491	0.124553713
Loxl2	96.13191241	-0.831819603	0.198777955	-4.184667269	2.86E-05	0.000298451
Lpar6	2418.130357	-0.411525005	0.132234667	-3.112081083	0.001857735	0.011289657
Lpcat2	434.0012661	0.715361255	0.149341365	4.790107908	1.67E-06	2.29E-05
Lpgat1	722.4920497	0.292489058	0.096155738	3.041826363	0.002351475	0.013795381
Lpin1	772.4286808	-0.792500628	0.090401336	-8.766470325	1.84E-18	1.38E-16
Lpl	38786.86491	-1.556609863	0.212055313	-7.34058411	2.13E-13	8.06E-12
Lpp	1030.21389	-0.290159386	0.138167854	-2.100049885	0.035724453	0.124553713
Lratd2	228.799962	-0.266380109	0.132027202	-2.017615354	0.043631337	0.143602831
Lrch2	7.138810455	-1.165654467	0.373794944	-3.118432945	0.001818155	0.011105028
Lrfn1	23.08896758	1.018732289	0.286269537	3.558647213	0.00037277	0.002867328
Lrp12	406.069713	-0.343450678	0.111244576	-3.087347623	0.002019513	0.012142159
Lrp3	5.370497354	-0.935465724	0.380076221	-2.461258221	0.013845068	0.059076751
Lrp5	85.5254688	0.485486139	0.185864904	2.612037716	0.009000432	0.041856055
Lrp6	1115.109601	0.294066191	0.109457176	2.686586689	0.007218621	0.035298094
Lrp8	45.5487022	1.132381074	0.240511162	4.7082267	2.50E-06	3.29E-05
Lrpap1	769.5480337	-0.497083806	0.08551826	-5.812604315	6.15E-09	1.28E-07
Lrpprc	548.7370335	0.383179512	0.109724477	3.492197223	0.000479064	0.003555549
Lrr1	22.54881224	1.958631993	0.337225929	5.808070575	6.32E-09	1.31E-07
Lrrc17	15.27422699	0.709114641	0.334309561	2.121131801	0.033910712	0.119881758
Lrrc20	110.2938102	-0.58824789	0.16556318	-3.553011553	0.000380848	0.0029175
Lrrc25	90.96272462	0.512400851	0.171006206	2.996387454	0.002731991	0.015696395
Lrrc32	121.1738395	-1.286980743	0.221951516	-5.79847691	6.69E-09	1.38E-07
Lrrc45	65.27605073	-0.434334976	0.191086424	-2.272976626	0.023027589	0.088928209
Lrrc56	31.31882121	-0.787622037	0.246358489	-3.19705662	0.001388377	0.008817338
Lrrc58	1088.349858	0.251003578	0.097979966	2.561784697	0.010413585	0.046946275
Lrrc61	107.0072038	-0.52906798	0.171415088	-3.086472641	0.002025466	0.012158466
Lrrc75b	2.337993776	-0.725631911	0.365813828	-1.983609848	0.04729935	0.152533368
Lrrc8b	138.7311582	0.515865291	0.166289099	3.102219538	0.001920755	0.011613523
Lrrfip1	944.2354188	0.391708698	0.089723886	4.365712592	1.27E-05	0.000145693
Lrrk1	261.1812456	-0.290770012	0.116860799	-2.488174086	0.012840086	0.055690094
Lrrn4	13.99797916	-1.697284222	0.345587837	-4.911296177	9.05E-07	1.31E-05
Lsm10	270.4673561	0.332990161	0.11429443	2.913441721	0.003574686	0.019729793
Lsm12	323.7941395	0.255833844	0.097303484	2.629236221	0.008557689	0.040334562
Lsm2	116.0091996	0.417092713	0.17388206	2.398710447	0.016452919	0.067759908
Lsm4	422.5813224	0.248646478	0.113494228	2.190829276	0.028464149	0.104619517
Lsm5	38.23662312	0.512079325	0.250331034	2.045608632	0.040794892	0.137020801
Lsm6	415.9296427	0.434657835	0.119785518	3.628634253	0.000284925	0.002282895
Lsm7	34.42640045	0.614361763	0.238166336	2.579549124	0.009892939	0.045062291
Lsm8	293.5318716	0.322944639	0.123673334	2.611271393	0.009020628	0.041936976
Lst1	704.20646	0.449652133	0.125101669	3.594293627	0.000325273	0.002545031
Ltbp1	479.0879352	0.653328904	0.264269409	2.472207834	0.013428142	0.057691421
Ltbp2	116.2250943	-1.990245148	0.194458591	-10.23480187	1.38E-24	2.00E-22
Ltbp3	137.3767361	-1.221504062	0.166912739	-7.318219496	2.51E-13	9.43E-12

Ltbr	743.491113	0.30231932	0.090858033	3.327381301	0.000876663	0.00595298
Ltc4s	84.16611894	0.959006464	0.182214761	5.263055852	1.42E-07	2.35E-06
Ltn1	376.9113592	0.208298524	0.096330725	2.162326967	0.030592983	0.110708678
Ly6a	247.9869221	0.621932774	0.315575974	1.9707862	0.048748336	0.155256001
Ly6i	36.59394654	-0.793477899	0.3767584	-2.106065585	0.035198656	0.123187087
Ly75	37.152168	-1.06977635	0.248224187	-4.309718411	1.63E-05	0.00018225
Lyl1	405.7325856	0.347762397	0.106771696	3.257065414	0.001125705	0.007377054
Lyn	2530.990768	0.634928008	0.095872299	6.622642965	3.53E-11	1.01E-09
Lypla1	451.9341909	0.219110901	0.110937974	1.975075743	0.048259553	0.154287898
Lyrm9	116.0525893	0.387316889	0.150755078	2.56917971	0.010193958	0.046120206
Lyst	579.6963611	-0.719464067	0.149534976	-4.811343035	1.50E-06	2.08E-05
Lyve1	17.69535465	3.181787785	0.369365424	8.614200405	7.04E-18	4.87E-16
Lyz1	21428.45611	0.493118261	0.101995714	4.834695915	1.33E-06	1.87E-05
Lyz2	180969.9526	0.274711841	0.060353019	4.55174978	5.32E-06	6.59E-05
Lyzl4	57.22804231	-0.771244784	0.284089989	-2.71479043	0.006631772	0.032943729
Lzts2	23.8572636	-0.705735939	0.275487833	-2.561768082	0.010414083	0.046946275
Macf1	2631.999257	-0.31714671	0.123348327	-2.57114724	0.010136222	0.045928226
Maco1	472.5131114	0.616679146	0.108374208	5.690275906	1.27E-08	2.52E-07
Mad1l1	79.96587447	0.470386881	0.191915987	2.451004157	0.014245831	0.060357759
Mad2l1	298.8000285	1.228087978	0.191412988	6.415907243	1.40E-10	3.73E-09
Madd	268.3917194	0.322563967	0.109394227	2.948637944	0.003191777	0.017959877
Maf	11954.72486	1.411078519	0.071516058	19.73093254	1.17E-86	2.19E-83
Maf1	510.4694526	-0.269987536	0.086035275	-3.138102799	0.001700452	0.010518832
Mafg	1674.300225	0.293972644	0.081932077	3.588004283	0.000333219	0.002599072
Mafk	87.53169453	-0.356629381	0.16137844	-2.209894835	0.027112462	0.100753847
Mag	35.86035685	-0.568434938	0.231839157	-2.451850436	0.014212372	0.060250559
Magea8	2.422546013	1.17271071	0.360521573	3.252817025	0.001142671	0.007474127
Magohb	107.6269754	0.410104248	0.150786979	2.719759033	0.00653295	0.032593081
Magt1	2158.497398	0.301028847	0.086015038	3.499723453	0.000465741	0.003478639
Malt1	199.9761857	-0.60278128	0.144121015	-4.182466258	2.88E-05	0.000300937
Mamdc2	260.1074411	-3.594053666	0.317272623	-11.32796658	9.54E-30	2.11E-27
Maml2	292.2147755	0.420271831	0.126731815	3.316229869	0.000912407	0.006165015
Man1a	814.4928203	1.089204856	0.135428182	8.042675042	8.79E-16	4.60E-14
Man2a1	1638.910494	0.500308941	0.111469721	4.488294558	7.18E-06	8.64E-05
Man2a2	556.3420819	-0.715336073	0.114775529	-6.232478985	4.59E-10	1.14E-08
Man2b1	6027.517176	-0.270903961	0.083806667	-3.232487014	0.001227177	0.007962061
Man2c1os	9.870055533	-0.71341372	0.346763169	-2.057351482	0.039652429	0.13417452
Manea	332.1485338	0.394693529	0.106225665	3.715613644	0.000202711	0.001689109
Manf	769.8385029	0.333448378	0.111742878	2.984068291	0.002844433	0.016226223
Mansc1	10.08082198	0.99972738	0.361754	2.763555841	0.005717531	0.029254342
Maoa	250.6290139	-0.551366608	0.130438305	-4.227029845	2.37E-05	0.000251672
Maob	6.97546337	-0.894505645	0.371211524	-2.409692551	0.015965969	0.066133395
Map1a	37.60025332	-0.673720792	0.242320267	-2.780290729	0.005431025	0.028046592
Map1b	87.55139397	-0.689505241	0.201748355	-3.417649884	0.000631643	0.004494579

Map1lc3a	246.1060552	-0.525749332	0.120463287	-4.364394708	1.27E-05	0.000146255
Map1s	217.0137887	0.238640886	0.11381065	2.096823856	0.036009171	0.125206123
Map2k1	810.1749898	0.284930935	0.081907656	3.478685024	0.00050388	0.003699478
Map2k5	100.559166	-0.325999416	0.156954805	-2.077027309	0.037799039	0.129479611
Map3k1	864.7712385	-0.236193809	0.118893562	-1.986598808	0.046966873	0.151772582
Map3k14	111.8269867	-0.338325488	0.154301824	-2.192621442	0.028334665	0.10426916
Map3k5	296.7947557	0.243279049	0.124100203	1.960343689	0.049955633	0.158227983
Map3k8	132.5066159	-0.614239731	0.148177483	-4.145297385	3.39E-05	0.000346225
Map4	504.7649437	-0.478057612	0.089432099	-5.345481321	9.02E-08	1.54E-06
Map4k1	118.9221488	-1.196294712	0.160519145	-7.452660608	9.15E-14	3.60E-12
Map4k3	371.1435351	-0.755560679	0.105320767	-7.173900274	7.29E-13	2.56E-11
Map6	19.62703763	-0.614872551	0.298207899	-2.061892234	0.039217997	0.133215139
Mapk1ip1	96.37366859	-0.384522941	0.169766978	-2.265004329	0.023512414	0.09037884
Mapk3	1446.714711	-0.37752572	0.078344314	-4.818801775	1.44E-06	2.01E-05
Mapkap1	270.8883485	0.298613499	0.113662402	2.627196808	0.008609152	0.040494348
Mapkapk2	1076.648982	-0.251220028	0.082079061	-3.060707853	0.002208144	0.013087529
Mapkapk3	301.7138229	-0.52758739	0.127019386	-4.15359738	3.27E-05	0.000335528
Mapre1	2269.251215	0.285946091	0.081640933	3.502484356	0.000460941	0.003449935
Mapre2	627.6915587	-0.379884527	0.084341129	-4.504143229	6.66E-06	8.07E-05
Mapre3	71.64342011	-0.514190723	0.178278965	-2.88419176	0.003924197	0.02136113
March1	752.3161435	-0.652597296	0.132588417	-4.921978201	8.57E-07	1.25E-05
March6	349.2008135	-0.305493947	0.104656246	-2.919022588	0.003511308	0.01943718
March7	1110.648829	-0.193139885	0.074175636	-2.603818409	0.009219159	0.042609152
March8	907.6207636	0.361143848	0.093140142	3.877424257	0.000105568	0.00095208
Marcksl1	514.1537773	1.836775663	0.150880335	12.17372475	4.29E-34	1.29E-31
Marco	5448.841922	2.250157832	0.300964997	7.476476846	7.63E-14	3.04E-12
Mark1	7.201695784	-1.023073095	0.373984009	-2.735606522	0.006226543	0.031387533
Mark2	305.1654114	-0.244348275	0.109860941	-2.224159676	0.026137703	0.098062127
Marveld2	19.04949651	-0.709346009	0.338499221	-2.09556172	0.036121088	0.125478974
Mast4	106.688662	-0.661805331	0.169997858	-3.893021593	9.90E-05	0.000896646
Mastl	41.1518482	1.930511156	0.290422472	6.647251309	2.99E-11	8.62E-10
Matk	97.00035639	-1.544972171	0.226885671	-6.809474425	9.80E-12	2.99E-10
Matr3	1460.525406	-0.139350709	0.068121399	-2.045623131	0.040793464	0.137020801
Mbd6	242.8098854	-0.419515893	0.133642342	-3.139094143	0.00169471	0.01049629
Mblac2	166.4398411	-0.281104299	0.131654179	-2.135171863	0.032746974	0.11660946
Mbnl1	6579.489634	-0.254559201	0.07907545	-3.219193849	0.001285515	0.008272612
Mbnl2	2622.225328	-0.208226655	0.06787725	-3.067694314	0.002157172	0.012856503
Mboat1	27.25351941	1.38281127	0.276718126	4.997183568	5.82E-07	8.70E-06
Mboat7	616.656291	-0.354874066	0.101449243	-3.498045492	0.000468681	0.003490596
Mbtd1	817.4664811	-0.55670293	0.077047966	-7.225407242	5.00E-13	1.80E-11
Mcam	3.185101876	-0.979971707	0.374723426	-2.615186666	0.00891787	0.041549357
Mcc	12.58225568	-0.698613291	0.340022864	-2.054606809	0.039916998	0.134796216
Mccc2	24.2378726	0.666812247	0.286357111	2.328603764	0.019880066	0.079009576
Mcee	442.7963851	0.323929016	0.097995908	3.305536149	0.000947949	0.00636789

Mcf2l	94.59898234	-1.982811249	0.197344728	-10.04744981	9.43E-24	1.24E-21
Mcfd2	799.0686929	0.362078411	0.089593534	4.041345324	5.31E-05	0.00051722
Mcm10	60.71381467	2.020182857	0.268898131	7.512818523	5.79E-14	2.34E-12
Mcm2	154.2161638	1.64509489	0.208263643	7.899097821	2.81E-15	1.37E-13
Mcm3	657.2787955	1.054129851	0.126940515	8.304124552	1.01E-16	5.92E-15
Mcm4	332.2745946	1.128873368	0.136028382	8.298807575	1.05E-16	6.14E-15
Mcm5	210.0967477	1.76431392	0.184887112	9.542655002	1.39E-21	1.39E-19
Mcm6	440.2232049	1.107133708	0.156602119	7.069723668	1.55E-12	5.31E-11
Mcm7	415.8391981	1.38385778	0.150698919	9.182931047	4.20E-20	3.62E-18
Mcm8	26.14641959	0.945472038	0.287798269	3.285190144	0.001019136	0.006785346
Mcoln2	73.88372161	-0.398401591	0.177745558	-2.241415177	0.024999194	0.094929886
Mcrip1	230.6190002	-0.290159886	0.127436254	-2.276902183	0.022792067	0.088132068
Mcu	245.0269883	0.46115782	0.134977855	3.416544288	0.000634213	0.004506459
Mcub	404.0649233	-0.339193466	0.096961776	-3.49821837	0.000468377	0.003490596
Mcur1	1140.339176	-0.666218314	0.081143482	-8.210373748	2.21E-16	1.24E-14
Mdga1	58.22370783	0.886708182	0.211191033	4.198607145	2.69E-05	0.000282037
Mdk	65.7489525	-0.690896471	0.229017135	-3.01678942	0.002554672	0.014859676
Mdm1	40.16745805	0.610990358	0.258025317	2.36794732	0.017887083	0.072451133
Mecom	1.964039107	0.671859702	0.33164781	2.025822818	0.042782945	
Mecp2	480.3758608	-0.468321732	0.118044493	-3.967332318	7.27E-05	0.000680857
Med13l	1295.865864	-0.212385781	0.094060486	-2.257970258	0.023947514	0.091631907
Med15	894.7550305	-0.173448211	0.075555466	-2.295640811	0.021696427	0.084725028
Med18	32.40114297	0.577650392	0.24671041	2.341410693	0.019211021	0.076716285
Med21	219.7199305	0.380141249	0.124817951	3.045565525	0.002322432	0.013667738
Med28	875.2759605	0.27135689	0.088773196	3.056743488	0.002237557	0.013214017
Med30	42.12674344	0.614849067	0.240906189	2.552234421	0.010703448	0.048091808
Med7	241.1642909	0.285588712	0.127209179	2.245032271	0.024766061	0.094139888
Med8	572.4188694	0.271601907	0.103695812	2.619217705	0.008813168	0.041227935
Mef2a	5014.713694	0.452331167	0.076971223	5.876626964	4.19E-09	8.89E-08
Meg3	90.13957293	-1.394430601	0.234655774	-5.942451702	2.81E-09	6.13E-08
Megf8	76.59321828	-0.385789612	0.192417117	-2.004965139	0.044966774	0.146858842
Megf9	132.5189027	0.820887393	0.1753137	4.682391586	2.84E-06	3.71E-05
Meis2	67.76178833	-1.05636875	0.209278421	-5.047671644	4.47E-07	6.81E-06
Meis3	62.75344684	-0.442279423	0.187857082	-2.354339899	0.018555635	0.074655339
Melk	40.79457257	1.579140338	0.295512179	5.343740293	9.10E-08	1.56E-06
Memo1	135.2727123	0.436276829	0.159197043	2.740483246	0.006134891	0.031040563
Mertk	2472.122254	-0.250903908	0.064534505	-3.887903175	0.000101114	0.000913556
Met	320.1682418	-0.821667879	0.18105273	-4.538279414	5.67E-06	7.01E-05
Metap1	350.8187928	0.479912945	0.104473155	4.593648446	4.36E-06	5.51E-05
Metap2	1854.325792	0.287753937	0.108267284	2.657810619	0.007865008	0.037805947
Metrn	33.48686902	1.255864618	0.262416304	4.785772074	1.70E-06	2.34E-05
Mettl16	267.4294098	0.309182871	0.133773496	2.311241623	0.020819512	0.081983318
Mettl27	44.68122207	-0.874464922	0.218673373	-3.998954735	6.36E-05	0.00060583
Mettl7a1	178.2385454	-0.35265245	0.157710376	-2.236076404	0.025346767	0.095837473

Mex3a	39.4043289	-0.612361994	0.243502246	-2.514810452	0.01190964	0.052459043
Mex3b	119.3812527	0.452066442	0.199275167	2.268553822	0.023295472	0.089731816
Mfap5	5.251550551	-0.799338741	0.381055676	-2.097695409	0.035932061	0.125053904
Mfge8	1775.502543	-1.307644646	0.13924981	-9.390638656	5.96E-21	5.70E-19
Mfng	46.26777691	0.480493803	0.217660857	2.207534275	0.027276755	0.101272208
Mfsd1	4250.24731	0.719930353	0.083014018	8.672394979	4.23E-18	3.00E-16
Mfsd12	1088.161557	-1.22026973	0.130791131	-9.329911895	1.06E-20	9.88E-19
Mfsd6	805.0826886	0.773041321	0.104563659	7.393020954	1.44E-13	5.55E-12
Mgat2	863.4049415	0.349637637	0.078105389	4.476485451	7.59E-06	9.11E-05
Mgat4b	998.7456417	-0.239105545	0.086860413	-2.752756241	0.005909588	0.03008317
Mgll	70.37908576	-2.880997957	0.248902097	-11.57482395	5.53E-31	1.38E-28
Mgmt	96.56082103	0.354914033	0.179019927	1.98253926	0.047418918	0.152772799
Mgrn1	615.2276168	-0.265414306	0.091293034	-2.907278832	0.003645881	0.020078433
Mgst1	3817.806519	-0.487991228	0.068199514	-7.155347571	8.35E-13	2.91E-11
Mia2	1830.637129	0.206776655	0.075195985	2.749836364	0.005962503	0.030301146
Mib2	106.5031191	-0.420787564	0.15641	-2.690285554	0.00713909	0.035000433
Mical1	165.519455	-1.105052151	0.175576733	-6.293841622	3.10E-10	7.90E-09
Mical2	108.6291585	-1.119536921	0.181574002	-6.16573359	7.02E-10	1.69E-08
Micall2	30.42050665	0.666473714	0.253873612	2.625218541	0.008659337	0.040647691
Micu1	273.6430389	0.257383256	0.121980864	2.110029782	0.034855791	0.122369893
Mid1ip1	1304.869613	1.132034677	0.076998463	14.70204261	6.25E-49	4.08E-46
Midn	927.1947056	0.278690378	0.107687278	2.587960101	0.009654616	0.044213252
Mier1	2156.893915	-0.476650984	0.118920149	-4.008159979	6.12E-05	0.000586417
Mif	565.8012822	0.579438534	0.10177484	5.693337722	1.25E-08	2.48E-07
Mif4gd	528.113047	-0.218325962	0.089092139	-2.450563693	0.014263273	0.06039756
Milr1	684.225652	0.29447745	0.107964002	2.727552195	0.006380616	0.031960583
Mindy1	1188.561189	-0.242685573	0.100607472	-2.412202272	0.015856482	0.06575248
Mindy2	2746.810641	0.204946012	0.070110237	2.923196695	0.003464576	0.019213928
Mink1	94.01674855	-0.391221736	0.179188528	-2.183296778	0.029013962	0.1063798
Mir100hg	34.44623918	-0.681137691	0.277428985	-2.455178541	0.014081463	0.05976259
Mir17hg	34.00369748	-0.53498639	0.240707334	-2.222559577	0.026245513	0.098354776
Mis12	366.0812541	0.306919597	0.106891976	2.871306239	0.004087793	0.022138401
Mis18bp1	190.6151248	1.231529317	0.17395274	7.079677609	1.44E-12	4.95E-11
Mitd1	221.0896599	0.306075145	0.138872277	2.204004655	0.027524017	0.101887745
Mitf	1910.341164	-1.023620254	0.117943093	-8.678933407	3.99E-18	2.84E-16
Mki67	471.4416765	1.872355486	0.274490089	6.821213458	9.03E-12	2.79E-10
Mkrn1	812.6265557	-0.39608686	0.093836993	-4.221009725	2.43E-05	0.000257577
Mks1	55.24115029	-0.405984225	0.194697701	-2.085202977	0.03705088	0.127674524
Mlf1	9.273281916	1.143774683	0.366302385	3.122487679	0.001793296	0.010984488
Mlh3	121.4404468	0.443265385	0.16427601	2.69829652	0.006969533	0.034337421
Mllt6	493.1042577	-0.363155954	0.119385531	-3.041875766	0.002351089	0.013795381
Mlph	38.26801588	0.767928375	0.24522672	3.131503671	0.001739136	0.010709567
Mlxip	344.9216856	-0.706274402	0.10935646	-6.458460743	1.06E-10	2.84E-09
Mlxipl	121.6127963	1.670721345	0.165508697	10.09446259	5.85E-24	8.10E-22

Mmd	479.3777209	-0.324346636	0.111555394	-2.907493975	0.003643374	0.020071993
Mmgt1	357.4489854	0.252776348	0.103258542	2.447994555	0.014365383	0.060727126
Mmp12	12753.96845	-1.214191441	0.262883694	-4.618740039	3.86E-06	4.93E-05
Mmp14	1428.876286	-0.301593869	0.139074122	-2.168583655	0.030114307	0.109557675
Mmp2	20.75657268	-0.621458663	0.305543796	-2.033942992	0.041957344	0.139643791
Mmp23	19.87153456	-1.815754209	0.321654429	-5.645046492	1.65E-08	3.20E-07
Mmp27	141.3817564	-0.540533755	0.213391263	-2.533064129	0.011307026	0.050276903
Mmp8	1778.65914	-1.324769917	0.327747855	-4.042039935	5.30E-05	0.000516025
Mmp9	538.7573427	0.6661289	0.300713835	2.215158803	0.026749166	0.099856901
Mms22l	97.4336664	0.957071629	0.208984801	4.579623124	4.66E-06	5.84E-05
Mn1	5.23752143	-1.067788541	0.378146757	-2.823741101	0.004746671	0.025038064
Mns1	26.90513353	0.70613051	0.267776334	2.637016119	0.008363886	0.039609024
Mnt	761.2635309	-0.401668436	0.114296438	-3.514269069	0.000440966	0.003325415
Mob1a	1905.268589	0.309080633	0.077797896	3.972866242	7.10E-05	0.000668984
Mob3a	260.6576875	-0.303007719	0.109306252	-2.772098703	0.005569615	0.028624385
Mob4	834.5648794	0.250117557	0.089279118	2.801523605	0.005086192	0.026558275
Mocos	304.7257763	-0.372348942	0.135034641	-2.757432759	0.005825719	0.029736929
Mocs1	129.003676	-0.289101844	0.146958526	-1.96723423	0.049156213	0.156355934
Mon2	871.3873743	0.198520443	0.089862256	2.209163804	0.027163249	0.100900714
Morc3	782.8551956	0.240295373	0.101222518	2.373931976	0.017599793	0.071572282
Morrbid	129.5476355	1.161747446	0.156434591	7.42641021	1.12E-13	4.36E-12
Mospd1	144.3452333	0.364412861	0.135388059	2.691617448	0.007110646	0.034881494
Mospd2	572.8827783	0.43476754	0.096017304	4.52801238	5.95E-06	7.31E-05
Mospd3	156.7531053	0.295291244	0.13544097	2.180220982	0.029241085	0.107081738
Mov10	52.61744237	-0.58078072	0.214520573	-2.707342758	0.006782419	0.033569843
Mpeg1	9710.324952	-0.525303858	0.073077677	-7.188294465	6.56E-13	2.32E-11
Mphosph10	503.4905337	0.355059649	0.103140517	3.442484672	0.000576397	0.004160646
Mphosph9	64.0940426	0.454275126	0.204382545	2.222670861	0.026238002	0.098354776
Mpi	65.13427988	0.594437458	0.189029589	3.14467941	0.001662689	0.010323532
Mpp1	1787.454844	0.273014899	0.092811435	2.941608434	0.003265125	0.018290307
Mpp6	804.9672647	-0.292146793	0.105917384	-2.758251595	0.005811145	0.029672629
Mprip	513.3173031	-0.357151415	0.086576752	-4.125257732	3.70E-05	0.000375499
Mpv17	414.0158033	0.256985706	0.09304029	2.762090539	0.005743255	0.029365938
Mr1	172.9196933	0.3702283	0.140900999	2.627577539	0.008599524	0.040490549
Mras	277.1073306	-1.253338873	0.141612408	-8.850487699	8.71E-19	6.67E-17
Mrc1	3814.062247	1.84573027	0.229896439	8.028529172	9.86E-16	5.12E-14
Mrgpre	82.65203158	-1.63529705	0.210388515	-7.772748667	7.68E-15	3.52E-13
Mrnip	14.7627859	0.661230981	0.315152905	2.098127517	0.035893882	0.124979001
Mroh1	388.4908819	-0.242609257	0.105103594	-2.308286967	0.020983181	0.08243314
Mrpl12	318.6637939	0.393414166	0.123460675	3.186554475	0.001439784	0.009104692
Mrpl16	212.8150179	0.276383246	0.128849719	2.145004645	0.031952476	0.114660641
Mrpl18	562.260703	0.355813479	0.118065755	3.013689104	0.002580921	0.015000731
Mrpl27	300.6669287	0.272969513	0.118395821	2.305567135	0.021134831	0.082837938
Mrpl28	214.7906097	0.349054851	0.137285707	2.542543273	0.011004897	0.049225185

Mrpl3	486.1821177	0.470568954	0.093730539	5.020444335	5.16E-07	7.77E-06
Mrpl33	961.2858274	0.700460343	0.080385279	8.713788755	2.94E-18	2.13E-16
Mrpl34	424.6743159	0.248504561	0.094754324	2.622619758	0.00872566	0.04086953
Mrpl38	117.8252806	-0.306657183	0.153655399	-1.995746226	0.045961553	0.14961931
Mrpl41	193.3943139	0.2755378	0.12824863	2.148465839	0.031676767	0.113776167
Mrpl42	304.9999634	0.280146806	0.123518497	2.268055502	0.023325824	0.089802627
Mrpl49	146.4529583	0.525954289	0.145261071	3.620751825	0.000293748	0.00233861
Mrpl51	242.4475683	0.279362113	0.135651636	2.059408358	0.039455136	0.133748187
Mrpl57	279.6981375	0.309995832	0.122573465	2.529061503	0.011436798	0.050703703
Mrps16	298.9653345	0.368820334	0.106114704	3.475676027	0.000509567	0.003733925
Mrps18c	336.1225614	0.357640297	0.107430313	3.329044525	0.000871445	0.005922903
Mrps25	302.2525911	0.358243795	0.123621768	2.897902206	0.003756677	0.0205979
Mrtfb	461.4989121	-0.585532478	0.107059679	-5.46921572	4.52E-08	8.16E-07
Ms4a4a	41.33898758	0.607475471	0.286461283	2.120619806	0.03395381	0.11995705
Ms4a6b	383.8754933	3.44868013	0.185687387	18.57250613	5.36E-77	8.05E-74
Ms4a6c	2607.59196	1.309103478	0.131493432	9.95565677	2.38E-23	3.00E-21
Ms4a6d	2625.634486	0.772630036	0.092294551	8.371350524	5.70E-17	3.47E-15
Ms4a7	1458.11832	0.317387706	0.114531101	2.771192322	0.005585143	0.028694364
Ms4a8a	338.4598132	-0.969115289	0.186873803	-5.185934439	2.15E-07	3.45E-06
Msantd3	48.11540908	-0.517735331	0.218742299	-2.366873409	0.017939068	0.072602909
Msantd4	513.1411001	0.405300054	0.096489299	4.200466355	2.66E-05	0.000280713
Msh3	110.4119282	0.561675207	0.165854727	3.386549286	0.000707776	0.004947177
Msh6	216.3738945	0.520102429	0.136036436	3.823258267	0.0001317	0.001157153
Msn	5646.073187	0.396983167	0.078880293	5.032729355	4.84E-07	7.32E-06
Msr1	8341.383858	0.806214811	0.088481165	9.111711061	8.11E-20	6.80E-18
Msra	163.9860444	-0.320973926	0.147850805	-2.170931199	0.029936374	0.109042514
Msrb1	1531.352458	-0.193243576	0.091915503	-2.102404589	0.03551785	0.124014976
Msrb2	97.57515685	0.729929164	0.165692609	4.405321201	1.06E-05	0.000123071
Msrb3	30.17970786	-1.057509695	0.26898185	-3.931528077	8.44E-05	0.000779511
Mst1r	31.57371269	-0.901986348	0.285101865	-3.163733594	0.001557593	0.00978025
Mt1	3371.829634	0.745204865	0.092842771	8.026525478	1.00E-15	5.19E-14
Mt2	243.4279311	1.061309786	0.147722628	7.184476736	6.75E-13	2.38E-11
Mtap	506.8893602	0.290102558	0.106165674	2.732545714	0.006284695	0.03159612
Mtbp	28.52674968	0.509844531	0.253615638	2.010304001	0.044399024	0.145416008
Mtch2	1096.650659	0.297867706	0.104880547	2.840066298	0.004510416	0.024062499
Mtdh	3727.48553	-0.265639812	0.08229652	-3.227837741	0.001247297	0.008064708
Mtf2	397.3795949	-0.271060915	0.103747427	-2.612700129	0.008983008	0.041787974
Mtfmt	45.41197649	0.495013581	0.236054369	2.097032066	0.035990737	0.125177219
Mtfr2	47.73966711	0.925399825	0.256473122	3.608174675	0.000308359	0.002436445
Mthfd1l	94.44285842	0.4320851	0.193116057	2.237437461	0.025257762	0.095597287
Mthfd2l	54.75645546	0.517618655	0.219192997	2.361474413	0.018202428	0.073490405
Mtmr1	168.403819	0.271248841	0.132621228	2.045289773	0.040826298	0.137095605
Mtmr10	154.9879673	0.28723866	0.138018052	2.081167333	0.037418593	0.128528455
Mtmr14	238.6177207	-0.305549042	0.114818007	-2.661159608	0.007787203	0.037540174

Mtmr2	262.7806001	0.324929573	0.114995477	2.825585682	0.004719426	0.02493818
Mtr	167.723522	0.681593056	0.146736908	4.645000798	3.40E-06	4.37E-05
mt-Rnr2	297937.5351	-0.256502372	0.124303498	-2.063516936	0.03906354	0.132780646
Mtrr	78.05643491	0.485423634	0.177634108	2.732716366	0.00628144	0.031590338
Mtss1	2104.248333	0.346301593	0.082479792	4.198623524	2.69E-05	0.000282037
mt-Ti	47.15808878	-0.494613734	0.223348191	-2.214541031	0.026791583	0.099990374
mt-Tl2	5.49657072	1.000291206	0.379650191	2.634770716	0.008419413	0.039782789
Mturn	80.20420808	-0.517951864	0.205070658	-2.52572391	0.011546018	0.051067224
Mtus1	864.476636	1.362052282	0.119299335	11.41709872	3.44E-30	7.93E-28
Mtx2	399.716306	0.334318462	0.104223966	3.207692769	0.001338044	0.008541055
Mvb12b	451.6532336	0.715876106	0.103472793	6.918495994	4.56E-12	1.49E-10
Mvp	325.8440989	-0.27861916	0.101739025	-2.738567227	0.006170754	0.03120098
Mxd3	15.64655745	1.606759727	0.337927035	4.754753425	1.99E-06	2.68E-05
Mxd4	1596.785578	-0.376480062	0.082544295	-4.56094587	5.09E-06	6.34E-05
Mxra8	103.0082573	-1.224787844	0.198712307	-6.163623486	7.11E-10	1.71E-08
Mybl1	23.02201462	0.745517274	0.284672552	2.618858998	0.008822441	0.04125845
Mybl2	10.29203798	0.876825643	0.37335676	2.348492744	0.018849566	0.07559472
Myc	50.2491474	0.619010468	0.221984253	2.788533235	0.005294731	0.027446644
Mycbp	124.3337387	0.478570186	0.152889668	3.130166955	0.00174707	0.01075401
Mycbp2	1335.696843	-0.422939433	0.122475616	-3.453254173	0.000553867	0.004023494
Mycl	2.118588841	-0.697676965	0.34329039	-2.032323027	0.042120965	0.140002064
Mydgf	283.3050352	0.30170121	0.11007212	2.740941204	0.006126347	0.031018248
Myef2	151.5621907	0.365516673	0.144986049	2.521047192	0.011700616	0.051705283
Myh10	66.47780615	-1.360375908	0.187442902	-7.257548258	3.94E-13	1.45E-11
Myh11	3.977099486	-0.89725184	0.377885993	-2.374398248	0.01757758	0.071510972
Myh15	8.692659137	-1.540014694	0.36993988	-4.162878285	3.14E-05	0.000323909
Myh9	2799.204884	-0.283457787	0.08073227	-3.511084055	0.000446283	0.003358763
Myl9	16.53971581	-1.322111386	0.338145043	-3.90989433	9.23E-05	0.000842367
Mylip	400.0767292	-0.285741642	0.105853054	-2.699418041	0.006946086	0.034255641
Mylpf	9.738702003	0.716560607	0.350535019	2.044191214	0.040934661	0.137259266
Myo10	461.1819488	-0.978093017	0.122444883	-7.988026854	1.37E-15	7.05E-14
Myo18a	560.3282825	-0.303660971	0.087852249	-3.456496251	0.000547247	0.003984732
Myo1d	48.22628365	-0.587926963	0.231396694	-2.540775116	0.011060704	0.049425073
Myo1e	1079.356501	-1.181816824	0.144603482	-8.172810269	3.01E-16	1.66E-14
Myo1f	3211.473465	-0.572728308	0.093899932	-6.099347428	1.07E-09	2.52E-08
Myo5a	3773.307968	-0.368782243	0.093867247	-3.928763798	8.54E-05	0.000786734
Myo7a	343.4578943	0.608324199	0.098271603	6.19023378	6.01E-10	1.46E-08
Myo9a	799.1934825	-0.267228705	0.089169083	-2.996876247	0.002727614	0.015683259
Myo9b	441.2864922	-0.603377174	0.096978592	-6.221756391	4.92E-10	1.21E-08
Myof	4120.556694	-1.081370775	0.098745932	-10.95104129	6.57E-28	1.20E-25
Myom1	47.05170223	-0.822679323	0.245355929	-3.353003639	0.000799397	0.005497958
Myoz1	19.0548878	0.621676599	0.302831648	2.052878563	0.040084357	0.135209247
Mypop	10.88859363	-0.681390514	0.340123921	-2.003359576	0.045138707	0.147356228
Myrf	67.95041595	-1.067134572	0.216046402	-4.939376728	7.84E-07	1.15E-05

Mysm1	423.3714952	-0.217213963	0.102737802	-2.114255489	0.034493452	0.121426984
N4bp3	24.2713824	-1.446489144	0.276057382	-5.239813303	1.61E-07	2.64E-06
Naa15	931.9366551	0.243298088	0.087107176	2.793088912	0.005220734	0.027104897
Naa40	110.8070353	0.530038658	0.153117836	3.461638892	0.000536897	0.003918878
Naa60	914.4356414	0.566848137	0.084180716	6.733705336	1.65E-11	4.89E-10
Naa80	136.3521623	0.328037594	0.142281831	2.305548015	0.021135901	0.082837938
Naaa	217.7150341	-0.651329084	0.14138713	-4.606707003	4.09E-06	5.21E-05
Nab1	1343.937871	0.64798051	0.083273868	7.781318724	7.18E-15	3.30E-13
Nab2	268.9708819	-0.301212244	0.106895396	-2.817822429	0.004835054	0.025414941
Nabp1	418.142398	-0.267301996	0.119428148	-2.238182535	0.025209154	0.095549262
Nacc2	582.8245555	0.315003049	0.08126006	3.876480619	0.000105978	0.000955205
Naga	150.2486397	-0.454516091	0.160015934	-2.840442687	0.004505097	0.02404267
Nagpa	677.4302094	0.208355749	0.091032818	2.288798186	0.022091081	0.085953034
Naip1	355.68295	-0.313348589	0.12665728	-2.473987976	0.013361419	0.057437646
Naip2	521.2585734	-0.35714083	0.111571289	-3.20101017	0.001369467	0.008711993
Naip5	223.2631107	-0.494193013	0.143346423	-3.447543379	0.00056571	0.004101258
Nanos1	34.7661802	1.208117837	0.275160011	4.390601066	1.13E-05	0.000130993
Nap1l1	1666.149988	0.237211295	0.086741242	2.734700225	0.006243711	0.031442742
Napsa	260.093175	-0.570945483	0.139763385	-4.085086256	4.41E-05	0.000437731
Nasp	851.6938203	0.364094463	0.11309986	3.219229992	0.001285353	0.008272612
Nat9	67.87455215	-0.43997785	0.203273977	-2.164457337	0.030429268	0.110355732
Nav1	1271.475999	-0.177772253	0.078672239	-2.259656705	0.023842565	0.091440168
Nav2	333.7876405	-0.2799165	0.128860412	-2.172245895	0.029837121	0.108760182
Naxd	206.3559149	-0.306902406	0.117717121	-2.607117833	0.009130793	0.042291918
Nbea	140.6707802	-0.747702975	0.157743153	-4.740002727	2.14E-06	2.87E-05
Nbl1	31.2903499	-1.185902679	0.259115931	-4.576726237	4.72E-06	5.92E-05
Ncam1	286.1918998	-0.834509894	0.162726224	-5.128306134	2.92E-07	4.58E-06
Ncapd2	177.4486799	1.879516208	0.204644224	9.184311045	4.14E-20	3.61E-18
Ncapd3	115.541922	0.477903445	0.152170024	3.14058861	0.001686087	0.010449801
Ncapg	102.3638426	1.732987285	0.237256332	7.304282561	2.79E-13	1.04E-11
Ncapg2	247.1058194	0.930284703	0.139682048	6.660016201	2.74E-11	7.96E-10
Ncaph	78.67368737	2.099149653	0.233872409	8.97561907	2.82E-19	2.29E-17
Ncbp2	385.6608893	0.318777119	0.098628809	3.232089302	0.001228886	0.007969706
Nceh1	2879.210952	-0.253698375	0.109033954	-2.326783226	0.019976806	0.079268091
Ncf1	2699.701766	0.407046026	0.095017668	4.2838983	1.84E-05	0.000202349
Ncf2	1898.822184	-0.331759305	0.081868332	-4.052352053	5.07E-05	0.000494756
Ncf4	887.5582425	-0.354422339	0.085087219	-4.165400428	3.11E-05	0.00032101
Nck2	260.1169334	-0.331053262	0.153158822	-2.161503058	0.030656501	0.110911791
Nckap1	79.60113784	-0.93922351	0.205576394	-4.568732301	4.91E-06	6.14E-05
Nckap5	3.844131827	-1.118950793	0.374864031	-2.984951074	0.002836237	0.016196121
Ncoa4	43.87644203	-0.550402792	0.219776952	-2.504369941	0.01226697	0.053740399
Ncoa7	868.2916132	-0.209654772	0.103112684	-2.033258806	0.042026383	0.139739025
Ncor2	347.1675073	-0.325001055	0.097166197	-3.344795477	0.000823433	0.005639432
Ndc1	171.0984978	0.677755807	0.20723272	3.270505776	0.001073553	0.007084792

Ndc80	98.53452815	1.91030537	0.224559715	8.506892555	1.79E-17	1.17E-15
Nde1	294.7743219	0.289947285	0.136139319	2.129783564	0.033189486	0.117831706
Ndn	13.95235851	-1.095254626	0.328988653	-3.329156235	0.000871095	0.005922903
Ndor1	78.44827516	-0.49125566	0.172658164	-2.845250103	0.004437656	0.023758798
Ndrg1	44.05888464	-0.670702105	0.217899806	-3.078029835	0.002083741	0.012488296
Ndrg2	9.650077812	-0.759353075	0.354914538	-2.139537812	0.032392137	0.115744664
Ndst1	578.1334417	0.314731857	0.105495696	2.983362071	0.002851005	0.016255712
Ndufa6	1476.65467	-0.147372456	0.065748137	-2.241469683	0.024995667	0.094929886
Ndufab1	315.1080317	0.342286349	0.141628471	2.416790534	0.015658025	0.065091407
Ndufaf4	292.0817424	0.403306521	0.114464623	3.523416326	0.000426022	0.003228942
Ndufaf8	370.5143157	0.218572509	0.110602074	1.976206246	0.048131423	0.154043135
Ndufb3	668.3859155	0.197391932	0.093941611	2.101219372	0.035621714	0.124290876
Ndufb6	482.4800607	0.314928199	0.103979215	3.028761061	0.002455588	0.014322196
Ndufb8	1849.685656	0.161793017	0.081697265	1.980396978	0.047658939	0.153282833
Ndufc2	1023.301904	0.271430707	0.084480962	3.212921606	0.001313921	0.008419308
Ndufs4	417.367259	0.201036938	0.100100324	2.008354522	0.044605632	0.145933446
Ndufv1	388.1551959	-0.212244084	0.107485876	-1.974623014	0.048310945	0.154287898
Ndufv3	657.9607708	0.410217557	0.104698756	3.9180748	8.93E-05	0.000818771
Neat1	5129.039397	-0.50801705	0.148304563	-3.425498449	0.000613672	0.004389601
Necap2	882.904831	0.256809622	0.091293826	2.813000965	0.00490815	0.025736063
Nectin2	100.2761214	-0.363048184	0.162864837	-2.229137924	0.025804729	0.097226104
Nectin4	35.14286662	0.673905127	0.250752918	2.687526561	0.007198338	0.035233351
Nedd1	253.6113176	0.265924215	0.125318829	2.121981329	0.033839306	0.119742151
Nedd4	467.6058071	-0.762600292	0.118986857	-6.409113649	1.46E-10	3.89E-09
Nedd4l	244.6312654	-0.269068585	0.135138475	-1.991058322	0.046474476	0.150685113
Nedd8	2360.753526	0.252795356	0.091920314	2.750157661	0.00595666	0.030290386
Neil2	72.37411232	-0.583010458	0.187555553	-3.108468128	0.001880599	0.011393683
Neil3	24.40472504	2.662575483	0.340016795	7.830717549	4.85E-15	2.30E-13
Nek2	45.15124856	2.117547779	0.285065019	7.428297537	1.10E-13	4.31E-12
Nelfcd	166.5812513	0.327219384	0.147865755	2.212949066	0.026901157	0.100249731
Nes	47.76824991	-1.446831668	0.235821181	-6.135291409	8.50E-10	2.02E-08
Net1	76.51698896	-0.656387917	0.200438564	-3.274758626	0.001057523	0.006990863
Neto2	51.16294807	-0.868983684	0.225465502	-3.854175818	0.00011612	0.001032967
Neu1	792.4984978	0.291935839	0.077397604	3.771897638	0.000162011	0.00137985
Neurl1b	63.09066764	0.983022006	0.215345124	4.564867713	5.00E-06	6.24E-05
Neurl3	766.8437806	-0.479834378	0.119534224	-4.014200786	5.96E-05	0.000573067
Neurl4	82.37012749	-0.495603893	0.167512297	-2.958612004	0.003090279	0.017487111
Nexn	42.25597059	-0.725494458	0.242463448	-2.992180734	0.002769922	0.015871792
Nfam1	998.5004068	-0.360054794	0.070312391	-5.120787214	3.04E-07	4.76E-06
Nfat5	365.5821093	-0.293225399	0.114779318	-2.554688457	0.010628288	0.047768408
Nfatc2	220.7683206	1.413073486	0.127593452	11.07481197	1.66E-28	3.24E-26
Nfatc2ip	96.29510946	0.378788847	0.177293331	2.136509279	0.032637925	0.116313785
Nfatc3	1091.044022	0.442620786	0.105906161	4.179367691	2.92E-05	0.000304431
Nfe2l2	4003.594	-0.169633837	0.075814518	-2.237484878	0.025254667	0.095597287

Nfia	960.0576703	0.248819556	0.106304794	2.34062403	0.019251543	0.076837208
Nfib	299.7770485	-0.73592526	0.145391598	-5.061676667	4.16E-07	6.37E-06
Nfic	1872.415806	0.446680846	0.077685041	5.749895199	8.93E-09	1.81E-07
Nfil3	228.8110608	-0.98302625	0.180925635	-5.433316555	5.53E-08	9.81E-07
Nfkbia	4261.668986	0.378711118	0.077208501	4.905044335	9.34E-07	1.35E-05
Nfkbib	258.7714375	-0.24378274	0.120602548	-2.021373048	0.043241163	0.142740927
Nfkbid	184.9489178	-0.79577877	0.147818738	-5.383476969	7.31E-08	1.27E-06
Nfu1	401.8519819	0.200975443	0.098219818	2.046180164	0.040738648	0.136923828
Nfxl1	235.3641367	0.860521711	0.135732319	6.339843882	2.30E-10	5.98E-09
Ngfr	10.82570345	-1.077991395	0.358894954	-3.00364043	0.002667704	0.015403707
Nhlrc1	12.57702696	0.665659212	0.330570324	2.013668997	0.044044297	0.144563094
Nhsl1	10.71077555	-0.986386463	0.354569074	-2.781930337	0.005403664	0.027924512
Nhsl2	144.1875363	1.693816274	0.18201812	9.305756319	1.33E-20	1.22E-18
Nid1	146.3923813	-0.573232597	0.165887543	-3.455549389	0.000549173	0.003996815
Nin	732.9367382	0.262350675	0.101534249	2.583863828	0.009770036	0.044619273
Nip7	204.9886753	0.300552879	0.128166511	2.345018808	0.01902612	0.076139995
Nipa1	46.02355634	0.613384171	0.220093899	2.786920368	0.005321155	0.027555065
Nipa2	773.9437631	0.302402737	0.088501299	3.416929914	0.000633316	0.004504346
Nipal1	35.9176875	0.925746684	0.239402437	3.866905846	0.000110225	0.000987247
Nipsnap3b	711.9639219	0.374023946	0.093987942	3.979488605	6.91E-05	0.000652627
Nit2	122.9338482	-0.583913989	0.150324088	-3.884367409	0.000102597	0.000925838
Nkiras2	532.9267911	0.238845663	0.097494658	2.44983333	0.014292235	0.060451966
Nktr	1319.900961	-0.215281598	0.108942366	-1.976105409	0.04814284	0.154046823
Nlk	351.3854943	0.489431575	0.112978005	4.332096106	1.48E-05	0.000166153
Nlrc3	132.2332295	0.579178726	0.142430836	4.066385771	4.77E-05	0.000470797
Nlrp3	321.7280112	0.735433306	0.163232479	4.505434896	6.62E-06	8.02E-05
Nlrx1	48.56059051	-0.525511595	0.210775593	-2.493227929	0.012658756	0.055063753
Nmd3	313.5647211	0.27707079	0.107732655	2.571836641	0.010116061	0.045864571
Nme4	108.7576833	1.520617204	0.182859324	8.315776142	9.12E-17	5.41E-15
Nmral1	72.93625142	0.467743055	0.214581089	2.179796257	0.029272568	0.107170876
Nnmt	53.56576842	-0.803540929	0.269415938	-2.982529298	0.002858773	0.016281444
Nnt	492.563774	0.562897515	0.096700411	5.821045712	5.85E-09	1.22E-07
Nol7	709.6449542	0.447119956	0.093363875	4.789003843	1.68E-06	2.30E-05
Nolc1	382.4851415	0.279960224	0.128069061	2.18600982	0.028814887	0.105753242
Nop10	833.4551708	0.243724316	0.096290487	2.531135974	0.011369376	0.050509246
Nop2	114.4515986	0.308021252	0.148562062	2.073350677	0.038139656	0.130211504
Nop53	786.5079088	-0.350664617	0.089664026	-3.91087297	9.20E-05	0.000840494
Nop56	126.2460824	0.401617903	0.152162128	2.639407766	0.008305102	0.039466328
Nop9	229.0478954	0.303001197	0.148427475	2.041409096	0.041210181	0.137860274
Npas4	1.851062088	-0.796149883	0.351125227	-2.267424331	0.023364317	
Npc1	1763.712525	-0.294564387	0.082808899	-3.557158581	0.000374888	0.002879193
Npepps	1171.014978	-0.313029673	0.099214568	-3.155077733	0.001604553	0.010026001
Nphp3	29.21832154	0.720577006	0.25548696	2.820406195	0.004796289	0.02524655
Nphs2	5.090225241	-0.752424868	0.381149738	-1.974092574	0.048371218	0.154382574

Npl	66.18755445	1.554773503	0.226029147	6.878641649	6.04E-12	1.92E-10
Npnt	15.00204642	-1.636733797	0.330741392	-4.948681466	7.47E-07	1.10E-05
Nptn	4184.810381	-0.252586014	0.096543706	-2.616286689	0.008889189	0.041493019
Nqo1	90.35419909	-1.082619943	0.269109547	-4.022971142	5.75E-05	0.000554618
Nqo2	310.5925408	0.698550434	0.150375097	4.645386435	3.39E-06	4.36E-05
Nr1d1	67.05944908	0.493808534	0.251767667	1.961365966	0.049836347	0.157983537
Nr1h2	335.7259825	-0.214296577	0.105493269	-2.03137677	0.042216789	0.140135481
Nr1h3	104.7014196	-0.861446507	0.173109685	-4.976304525	6.48E-07	9.62E-06
Nr1h4	3.602559043	-0.864969637	0.366811359	-2.35807757	0.018369854	0.07402696
Nr1h5	1.393893549	0.692781102	0.313424263	2.21036207	0.027080044	
Nras	1642.364708	0.210502683	0.087223807	2.413362715	0.015806081	0.065604645
Nrcam	7.185237968	-1.327192982	0.380297046	-3.489885068	0.000483228	0.003577606
Nrde2	168.8903375	-0.290721898	0.141628347	-2.052709816	0.040100729	0.135234078
Nrep	10.00717561	-0.727498395	0.345749797	-2.104118069	0.035368149	0.123651018
Nrm	68.71364799	1.412454545	0.230872652	6.117894565	9.48E-10	2.25E-08
Nrp1	5415.192042	0.513855312	0.091462939	5.618180609	1.93E-08	3.68E-07
Nrros	2782.010681	0.388398772	0.071592134	5.425159882	5.79E-08	1.02E-06
Nsd2	404.8244614	0.480042723	0.10415177	4.609069266	4.04E-06	5.16E-05
Nsd3	1119.303546	-0.280977493	0.085078038	-3.30258547	0.000957979	0.006423766
Nsg1	13.61560609	-0.973865468	0.328343552	-2.965995414	0.00301705	0.017117908
Nsl1	20.17522157	1.054220673	0.332515711	3.170438686	0.00152209	0.009581375
Nsmce1	179.2247039	0.499596802	0.142481509	3.506397464	0.000454216	0.003409918
Nsmce2	269.7556572	0.334892704	0.124923359	2.680785285	0.007344963	0.035760142
Nsmf	68.49721942	-0.747639891	0.178139883	-4.196925908	2.71E-05	0.000283741
Nsun3	117.6028567	0.394959126	0.148737103	2.655417637	0.007921027	0.037994127
Nt5dc2	418.445531	1.561973365	0.137000879	11.40119232	4.12E-30	9.24E-28
Nt5dc3	107.1387563	-0.691131956	0.167136869	-4.135125656	3.55E-05	0.000360209
Nt5e	158.9067551	1.554305274	0.194688502	7.983549422	1.42E-15	7.28E-14
Ntpcr	310.7691057	0.758881841	0.109504222	6.930160574	4.20E-12	1.38E-10
Nuak2	139.6272009	-0.518114751	0.146332031	-3.540679002	0.000399099	0.003041786
Nub1	743.8431336	-0.48844793	0.088920039	-5.493114193	3.95E-08	7.20E-07
Nucb2	487.3726111	0.615405314	0.116133854	5.299103518	1.16E-07	1.96E-06
Nucks1	2599.36156	0.361771632	0.115997216	3.118795826	0.001815917	0.011095877
Nudcd1	61.91279189	0.521353823	0.207983196	2.506711281	0.01218602	0.0534724
Nudcd2	407.1709714	0.379233152	0.105471723	3.595590752	0.000323656	0.002533704
Nudt11	1.353308854	-0.7409137	0.312739846	-2.369105535	0.017831165	
Nudt13	165.8077723	-0.396424928	0.136125819	-2.912194996	0.003588985	0.019794156
Nudt16	353.9183812	-0.553213439	0.127141966	-4.351147442	1.35E-05	0.000154317
Nudt16l1	407.5553808	0.256427102	0.107723325	2.380423207	0.017292765	0.070596443
Nudt2	63.13392781	0.452720407	0.202766494	2.232718035	0.025567544	0.096429288
Nudt4	398.025063	0.512361971	0.115354011	4.441648497	8.93E-06	0.000105572
Nudt5	129.8940212	0.479685019	0.142430375	3.367856175	0.000757551	0.005253497
Nudt9	268.3089242	-0.455144265	0.106520228	-4.272843525	1.93E-05	0.0002111
Nuf2	51.78274526	2.387197414	0.294469948	8.106760745	5.20E-16	2.80E-14

Nup107	96.88763046	0.515463849	0.192087418	2.683485747	0.007285908	0.035548559
Nup155	236.2917249	0.265904752	0.113246595	2.348015421	0.01887374	0.075671444
Nup205	228.4764909	0.437079439	0.134044824	3.260696134	0.001111391	0.007293473
Nup214	315.3977595	-0.252986274	0.097876117	-2.584760029	0.009744679	0.044532544
Nup35	93.2669217	0.346789804	0.162112637	2.139190444	0.032420248	0.115813059
Nup37	82.30068531	0.471892954	0.175414156	2.690164611	0.007141678	0.035001687
Nup62	628.616388	0.252997817	0.111536646	2.268293204	0.023311342	0.089769902
Nup93	294.8910668	0.501905101	0.136831411	3.668054713	0.000244403	0.0019901
Nupr1	147.7765789	0.779914516	0.151618934	5.14391241	2.69E-07	4.24E-06
Nus1	1474.586838	0.432688164	0.088274011	4.901648377	9.50E-07	1.37E-05
Nusap1	156.9996365	2.1432192	0.304555603	7.037201683	1.96E-12	6.63E-11
Nxf1	400.1406194	-0.311478654	0.103968039	-2.995907761	0.002736292	0.015715091
Nxn	132.9326826	-0.742188645	0.167962406	-4.418778362	9.93E-06	0.000116375
Nxpe5	1215.79734	1.820699645	0.277613864	6.55838876	5.44E-11	1.51E-09
Nxph3	1.876833611	-0.725158624	0.34968285	-2.073760905	0.038101522	
Nxt1	135.7625322	0.560463498	0.166472865	3.366695827	0.000760745	0.005268347
Nxt2	339.4933216	0.371494666	0.117783011	3.15405984	0.001610161	0.010055631
Oas1a	26.33289607	1.013827067	0.275377092	3.681595515	0.000231779	0.001900714
Oas1g	5.342406835	0.78258047	0.379823008	2.060381947	0.039362041	0.133572446
Oas2	48.69722411	1.091685936	0.238338996	4.58039161	4.64E-06	5.83E-05
Oat	676.7864621	0.274310441	0.085534739	3.207006215	0.001341241	0.008557825
Oaz1	981.7299773	0.18339423	0.092686478	1.9786514	0.047855268	0.153749521
Oaz2	199.7470137	0.268520128	0.136003427	1.974362955	0.048340487	0.154317314
Obsl1	12.17652069	-0.847108556	0.330311748	-2.564572896	0.010330288	0.046610532
Ocrl	294.679757	0.343516424	0.100997035	3.401252554	0.000670778	0.004725995
Ocstamp	22.63236947	-0.686802041	0.308631054	-2.225317354	0.026059941	0.097892751
Odf2	337.0087307	-0.21057432	0.098226104	-2.143771466	0.032051203	0.114905016
Oga	976.6013903	-0.305377274	0.081357828	-3.753508205	0.000174377	0.001474295
Ogfrl1	858.7953183	0.930422431	0.085799555	10.84414047	2.13E-27	3.71E-25
Ogn	3.801451357	-0.943992351	0.381279742	-2.475852367	0.013291853	0.057255628
Ogt	603.9110079	-0.792575091	0.107058569	-7.403191505	1.33E-13	5.17E-12
Oip5	16.71611174	2.053064238	0.346324658	5.92814919	3.06E-09	6.64E-08
Oip5os1	1352.845221	0.212607596	0.069418809	3.062679972	0.002193645	0.013032476
Oit3	48.34001195	-0.507397093	0.256647332	-1.977020721	0.048039288	0.153966418
Olfml3	20.41897812	-0.616940258	0.283448383	-2.1765524	0.02951398	0.107844241
Olfr56	2.224693836	0.983046475	0.360595791	2.726172902	0.006407342	0.032083746
Olr1	98.97054113	-0.67089941	0.242671894	-2.764635816	0.005698638	0.029167619
Opn3	45.29208469	0.523113725	0.213004133	2.455885325	0.0140538	0.059695831
Optn	159.3945002	-0.504789358	0.143789844	-3.510605092	0.000447088	0.003363133
Orai1	264.3974905	0.536357654	0.113309626	4.733557729	2.21E-06	2.95E-05
Orai2	123.9790644	0.519143541	0.153924185	3.372722359	0.00074429	0.005173485
Orc1	15.29430609	0.889947925	0.340352353	2.614784114	0.008928387	0.041585445
Orc6	114.6105445	0.531980422	0.166790845	3.18950613	0.001425161	0.009022736
Osbpl1a	268.2916256	0.351234798	0.107796308	3.258319371	0.001120742	0.007347739

Osbpl3	143.9505945	-0.380588529	0.162378569	-2.343834728	0.019086627	0.076300749
Osbpl5	44.6298454	-0.555966879	0.222290869	-2.501078345	0.012381578	0.054124774
Oscp1	30.77190353	1.084720487	0.262243625	4.136308314	3.53E-05	0.000358844
Osgep	157.0512827	-0.313166232	0.128725123	-2.43282916	0.014981368	0.062888219
Osm	118.8729273	0.695042181	0.163628063	4.247695475	2.16E-05	0.000232346
Osmr	20.18067355	-0.877876401	0.30474078	-2.880731622	0.003967533	0.0215392
Ost4	443.1865045	0.384139407	0.184902113	2.07752849	0.037752808	0.129439433
Ostc	986.2470756	0.382567064	0.111044689	3.445163099	0.000570715	0.004131558
Ostf1	2308.872496	0.340253047	0.085978111	3.957438045	7.58E-05	0.000707907
Otof	2.067361484	-0.774452694	0.345781757	-2.239715309	0.025109411	0.095228219
Otud1	149.1607746	-0.544128208	0.151668829	-3.587607371	0.000333726	0.002600328
Otud5	306.2677955	-0.539732447	0.114960221	-4.694949607	2.67E-06	3.50E-05
Otulin	441.099099	-0.291441871	0.092597976	-3.147389206	0.001647355	0.010247003
Otulinl	853.2790915	0.357878833	0.088733793	4.033174081	5.50E-05	0.000535199
Oxa1l	309.1985264	-0.239707969	0.110179516	-2.17561283	0.029584223	0.108032384
P2rx4	1927.725577	-0.603330526	0.075902634	-7.948742931	1.88E-15	9.55E-14
P2rx7	710.4057053	-0.257031518	0.10446851	-2.460373161	0.013879262	0.059188999
P2ry1	62.15152511	-1.439684757	0.230377867	-6.249232072	4.12E-10	1.03E-08
P2ry10	23.32984263	1.052060011	0.311890294	3.373173292	0.000743072	0.005167413
P2ry10b	219.822683	-0.4193752	0.148838802	-2.817646964	0.004837697	0.025419929
P2ry12	161.2595179	2.305205552	0.167283467	13.7802354	3.35E-43	1.73E-40
P2ry13	165.7140883	2.033018333	0.164805902	12.33583451	5.81E-35	1.89E-32
P2ry14	266.6684683	-0.732689847	0.150044559	-4.883148385	1.04E-06	1.50E-05
P3h3	12.07837576	-0.786111557	0.334718481	-2.348575304	0.018845388	0.07559472
P3h4	56.94795447	-1.119682348	0.215100021	-5.205403242	1.94E-07	3.12E-06
P4ha1	727.919746	0.68422969	0.085935821	7.962101005	1.69E-15	8.60E-14
P4ha2	107.6683093	1.111747981	0.183953052	6.043650655	1.51E-09	3.46E-08
P4ha3	10.13554281	-1.126149353	0.356775342	-3.156466324	0.001596933	0.009989653
Pa2g4	1356.962837	0.408910663	0.109361236	3.739082306	0.000184693	0.001550163
Pabpn1	169.670509	-0.323420956	0.144666349	-2.235633632	0.02537578	0.095910421
Pacs2	401.0631988	-0.519768898	0.109419596	-4.75023594	2.03E-06	2.74E-05
Pacsin2	165.3557127	0.303758056	0.143993226	2.109530188	0.034898843	0.122369893
Padi2	63.14682673	-0.53063054	0.215171792	-2.466078539	0.013660138	0.058437198
Padi4	333.162804	1.83101163	0.185524434	9.869382669	5.65E-23	6.78E-21
Pafah1b2	560.9754018	0.174120615	0.081414695	2.13868779	0.032460962	0.115903321
Paics	781.2093829	0.331342943	0.100922698	3.283135984	0.001026591	0.0068259
Paip1	772.9740558	0.289457542	0.076796659	3.769142405	0.000163809	0.001392939
Pak3	18.4774113	-0.775191691	0.302132607	-2.565733298	0.010295796	0.046482854
Palb2	38.43767081	0.48254035	0.241488638	1.998190697	0.04569599	0.14885863
Pald1	48.22076551	0.627959622	0.233647091	2.687641515	0.00719586	0.035232717
Palld	232.0763346	-0.476652867	0.152593822	-3.123670812	0.001786102	0.010949035
Pam	342.7323517	0.389188025	0.104779466	3.714353961	0.000203723	0.001694959
Pan3	682.8447648	-0.295042554	0.094874722	-3.109812055	0.001872064	0.011351138
Pank3	1194.909007	0.300734255	0.082961682	3.624977784	0.000288986	0.002313576

Paox	187.5357117	1.260644679	0.150759182	8.361976103	6.17E-17	3.73E-15
Papola	2017.390093	0.156835401	0.067196185	2.33399263	0.019596108	0.078004984
Pappa	25.54005636	-1.316826033	0.306717542	-4.29328569	1.76E-05	0.000195122
Paqr7	328.1320799	0.248153527	0.122255423	2.029795662	0.042377314	0.14054284
Park7	465.8921918	0.447648911	0.09749695	4.591414513	4.40E-06	5.55E-05
Parl	399.4320948	0.224272791	0.106797377	2.099984082	0.035730241	0.124553713
Parp1	442.699522	0.283489411	0.102893204	2.755181092	0.005865966	0.029901681
Parp2	448.5700897	-0.428341811	0.107893038	-3.970059784	7.19E-05	0.000674795
Parp3	195.2539746	0.882089167	0.16201121	5.444618097	5.19E-08	9.28E-07
Parpbp	24.92337749	1.655422812	0.311787257	5.309462705	1.10E-07	1.86E-06
Parva	51.44192521	-0.9608647	0.233695649	-4.111607146	3.93E-05	0.000396268
Parvg	69.58002158	-1.182708553	0.218178801	-5.420822506	5.93E-08	1.04E-06
Pask	17.49981484	1.230367473	0.328022462	3.75086348	0.000176227	0.001488257
Patj	9.60412797	-0.991111865	0.350948054	-2.824098475	0.004741381	0.025028223
Pawr	8.264532716	-0.861558724	0.368430849	-2.338454357	0.019363691	0.07718218
Paxbp1	329.8345742	-0.410728171	0.114484645	-3.587626731	0.000333702	0.002600328
Paxip1	157.8571198	0.377344892	0.154165806	2.447656198	0.014378879	0.06076706
Pbk	101.3215961	2.116269389	0.23036289	9.186676664	4.05E-20	3.56E-18
Pbld2	1.202621151	-0.651714552	0.299684555	-2.174668469	0.029654969	
Pbrm1	2763.044193	0.158384929	0.070773131	2.237924595	0.025225973	0.095549262
Pbx1	384.6776708	-0.455700869	0.161366566	-2.82401045	0.004742684	0.025028223
Pbxip1	1084.753024	-0.560444592	0.09743071	-5.7522376	8.81E-09	1.79E-07
Pcbd2	418.7352072	0.434101621	0.101959195	4.257601477	2.07E-05	0.000223996
Pcbp1	1993.780462	0.239978622	0.082253124	2.917562388	0.003527791	0.019508246
Pcdh19	9.959660088	1.707000954	0.363561774	4.695215715	2.66E-06	3.50E-05
Pcdh7	145.5721941	-0.799534657	0.168940976	-4.732627182	2.22E-06	2.95E-05
Pcdhb22	21.95292934	0.572337975	0.286392255	1.998440826	0.045668889	0.148828018
Pced1a	107.1674179	-0.577175949	0.164796296	-3.502359972	0.000461156	0.003449935
Pcf11	791.2326526	-0.23249633	0.08439743	-2.754779741	0.005873166	0.029928217
Pcgf2	26.38723861	0.543215129	0.273319973	1.98746957	0.046870385	0.15165672
Pcgf6	43.04717015	0.691950817	0.23637291	2.927369365	0.003418426	0.01900012
Pclaf	412.4718005	1.666576341	0.25381066	6.566218856	5.16E-11	1.44E-09
Pcna-ps2	21.03285146	-0.788948397	0.296380536	-2.66194403	0.00776908	0.037488932
Pcolce2	23.05234563	-1.418766229	0.304727871	-4.655846629	3.23E-06	4.17E-05
Pcsk6	2.96346479	-0.837940305	0.35402089	-2.366923333	0.017936648	0.072602909
Pctp	179.4486572	0.357382543	0.142830378	2.502146594	0.01234428	0.053993183
Pcyox1	754.6409739	0.214973156	0.097318919	2.20895543	0.027177741	0.100929562
Pcyox1l	34.15630086	1.001197724	0.276282738	3.623815705	0.000290288	0.00231475
Pcyt1a	751.449104	-0.320116892	0.101901928	-3.141421344	0.0016813	0.010426142
Pcyt1b	11.15369872	0.958673847	0.359001587	2.670388882	0.007576344	0.03673609
Pdap1	1215.586793	0.20706548	0.09693438	2.136140746	0.032667943	0.116393121
Pdcl3	303.7808487	0.362542891	0.141196961	2.567639477	0.010239359	0.046241967
Pde10a	38.39868329	0.519657567	0.23685217	2.194016487	0.028234224	0.104003683
Pde1b	102.8112509	-0.570623471	0.166639037	-3.424308513	0.000616366	0.004404669

Pde1c	58.47222789	-1.177771492	0.289680419	-4.065761493	4.79E-05	0.000471749
Pde3a	4.554734858	-0.94539684	0.381198203	-2.480066361	0.013135793	0.056711407
Pde4b	516.7796237	0.523785545	0.176259936	2.971665345	0.002961893	0.016824048
Pde4dip	192.2530433	-0.416643503	0.138972848	-2.998020901	0.00271739	0.015630463
Pde7a	168.5904926	0.407204218	0.125824412	3.23628946	0.001210945	0.007874378
Pde8a	438.0267614	0.612452943	0.103013706	5.945353923	2.76E-09	6.03E-08
Pdgfa	290.747683	-0.344480624	0.12038342	-2.861528803	0.004216032	0.02272629
Pdgfb	187.291331	-0.448282732	0.21188806	-2.115658293	0.03437388	0.121091272
Pdgfc	62.40564868	-0.686328883	0.207358606	-3.309864463	0.000933412	0.006287122
Pdgfra	120.6926222	-0.923330782	0.19182578	-4.813382132	1.48E-06	2.06E-05
Pdgfrb	4.899318024	-1.119842646	0.380641878	-2.94198487	0.003261159	0.018281305
Pdha1	898.3126191	0.217116899	0.082611473	2.628168853	0.008584589	0.040435948
Pdhx	151.4009406	0.481804457	0.146066816	3.298520975	0.000971956	0.006508767
Pdia4	397.33986	0.735192155	0.143751122	5.114340274	3.15E-07	4.90E-06
Pdia6	2255.503339	0.40754395	0.09294906	4.384594636	1.16E-05	0.000134556
Pdik1l	281.4658315	0.256532118	0.107685333	2.382238239	0.017207761	0.070306798
Pdk1	460.2610387	1.337341997	0.12812384	10.43788572	1.66E-25	2.72E-23
Pdk2	44.23282529	-0.581465608	0.23011414	-2.52685736	0.011508824	0.050947762
Pdlim1	221.9739206	-0.287661026	0.123866315	-2.322350717	0.020214059	0.080040206
Pdlim2	253.3192253	0.543355881	0.133817029	4.060438984	4.90E-05	0.000480425
Pdlim7	77.20786517	-0.791930512	0.188057423	-4.211110098	2.54E-05	0.000268372
Pdp1	194.3102709	-0.303151066	0.137863252	-2.198925829	0.027883197	0.102988711
Pdrg1	389.0229762	-0.256611418	0.113537665	-2.260143523	0.023812345	0.091347612
Pds5a	478.7296202	0.242910816	0.102137015	2.378283869	0.01739343	0.07091095
Pdss1	38.08406737	0.670769428	0.239999943	2.794873283	0.005192006	0.026998072
Pdxk	714.3543131	0.201945006	0.092062436	2.193565738	0.028266644	0.104097552
Pdzd11	180.8872864	0.378780692	0.145645175	2.600708817	0.009303138	0.042917981
Pea15a	1468.41299	-0.247026456	0.109066353	-2.264919014	0.02351765	0.09037884
Peak1os	26.43593122	0.851672404	0.284941476	2.988937998	0.002799489	0.016010647
Peg10	2.780039683	-1.031764079	0.37309185	-2.765442558	0.005684562	0.029120007
Peg3	17.39872058	-1.073774394	0.32157919	-3.339066794	0.000840603	0.00573928
Pelp1	231.0326578	0.505990874	0.118358775	4.275060064	1.91E-05	0.000209412
Pepd	449.6867853	0.322868948	0.105740463	3.053409637	0.002262569	0.013341598
Per1	109.9358219	-0.421290458	0.174856254	-2.409353097	0.015980828	0.06617668
Per3	118.921543	0.873772697	0.165209816	5.288866711	1.23E-07	2.06E-06
Perp	3.291870869	-0.906069583	0.37351653	-2.425781749	0.015275454	0.063872592
Pex11a	194.0509052	-0.552446367	0.127929935	-4.318351038	1.57E-05	0.00017592
Pex11b	220.381623	0.230781411	0.114175089	2.021293902	0.04324935	0.142740927
Pex6	98.00070602	-0.348318062	0.164694058	-2.114940069	0.034435056	0.121249854
Pex7	240.0175083	0.296069176	0.122838136	2.410238274	0.015942105	0.06605278
Pf4	20733.41008	2.536196674	0.113534322	22.33858997	1.56E-110	4.68E-107
Pfdn1	381.7951231	0.469256796	0.126090491	3.721587494	0.000197974	0.001651473
Pfdn5	1208.029101	-0.375729169	0.066633423	-5.638749362	1.71E-08	3.31E-07
Pfkfb3	263.710791	0.304011313	0.122370669	2.484347887	0.012978894	0.056195692

Pfkfb4	186.1883238	-0.437380736	0.132951931	-3.289765957	0.001002707	0.006687836
Pfkp	387.5718952	-0.225370062	0.107690091	-2.092765081	0.03637013	0.126023216
Pfn1	460.2481362	0.562940997	0.285036917	1.974975742	0.048270901	0.154287898
Pfn2	66.70747078	-1.16969426	0.203550133	-5.746467687	9.11E-09	1.84E-07
Pgap1	678.7388802	-1.011330605	0.090498999	-11.17504744	5.40E-29	1.11E-26
Pgap2	824.8755707	-0.282572696	0.090533905	-3.121180894	0.001801273	0.011016438
Pgd	3837.271814	-0.367171775	0.116419937	-3.153856506	0.001611283	0.010058454
Pgm1	253.9375943	0.575360865	0.135692436	4.240183787	2.23E-05	0.000239401
Pgm2l1	1150.725708	1.26877993	0.123276746	10.29212704	7.65E-25	1.18E-22
Pgm3	82.84205466	0.500561727	0.189577393	2.64040833	0.008280619	0.039387401
Pgpep1	250.0003844	0.381102417	0.116439796	3.272956768	0.001064288	0.007026733
Pgrmc1	214.7277862	-0.368631719	0.132861755	-2.774551019	0.005527796	0.028428937
Pgs1	370.6638354	0.437948503	0.114535732	3.82368452	0.000131472	0.00115583
Phc1	63.22743648	-0.522495841	0.196292079	-2.661828455	0.007771748	0.037489751
Phf10	526.4048847	0.314890812	0.089926639	3.501641085	0.000462402	0.003457533
Phf19	26.19574887	2.549092818	0.321802301	7.92130077	2.35E-15	1.18E-13
Phf20	364.1627981	0.327384201	0.107391183	3.04852029	0.002299714	0.013539351
Phf21a	401.0433434	-0.266683595	0.108610642	-2.455409435	0.014072421	0.059741108
Phf5a	484.4455327	0.266450545	0.107486278	2.478926145	0.013177859	0.056863894
Phf7	58.55088683	-0.451256372	0.196505719	-2.296403254	0.021652835	0.084598828
Phip	1272.033308	-0.343269266	0.089920499	-3.817475102	0.000134824	0.001179772
Phka2	177.9869068	-0.634987879	0.143098432	-4.437420248	9.10E-06	0.000107497
Phlda3	69.2005699	-0.555711087	0.219992394	-2.526046817	0.011535411	0.051050401
Phldb2	181.2511724	-1.193339304	0.140873522	-8.470997867	2.43E-17	1.55E-15
Phlpp1	227.8449985	-1.306822201	0.153858219	-8.493678213	2.00E-17	1.29E-15
Phtf2	139.18622	0.367989452	0.147550942	2.493982404	0.012631881	0.054990156
Phxr4	15.04877193	-0.712766065	0.348608298	-2.044604415	0.040893874	0.137230404
Pi4k2a	1043.818942	-0.325845951	0.085266257	-3.821511136	0.000132636	0.001162661
Pianp	249.3490731	-2.602297567	0.173213561	-15.02363648	5.14E-51	4.29E-48
Pid1	2664.862084	1.096982482	0.080056212	13.70265283	9.79E-43	4.90E-40
Piezo2	17.4944803	-1.184301139	0.323086324	-3.665587344	0.000246772	0.002007209
Pif1	11.27131305	1.051724251	0.345440436	3.044589286	0.002329983	0.01370143
Pigs	578.1821873	0.203954817	0.096727019	2.108560965	0.034982495	0.122573501
Pigt	207.3595815	0.360816687	0.124114445	2.907128887	0.003647629	0.020080691
Pigv	80.44406209	-0.402014876	0.183677816	-2.188695867	0.028618953	0.105122418
Pigz	39.41749221	0.510749772	0.226893892	2.251051222	0.024382293	0.092987313
Pik3cb	431.2785257	-0.545494061	0.120384446	-4.531266943	5.86E-06	7.20E-05
Pik3cg	682.5928202	1.064303363	0.102166577	10.4173341	2.07E-25	3.30E-23
Pik3r5	518.383469	-0.615021418	0.085811795	-7.167096558	7.66E-13	2.68E-11
Pik3r6	153.6482705	-0.550747108	0.14277282	-3.857506697	0.00011455	0.001020205
Pilra	633.9944334	1.928633124	0.107139017	18.00122095	1.91E-72	2.20E-69
Pilrb1	64.917005	2.029201923	0.238311043	8.5149303	1.67E-17	1.10E-15
Pilrb2	99.53609327	1.466518963	0.194290981	7.548054764	4.42E-14	1.83E-12
Pim1	695.4677466	-0.532416643	0.147335981	-3.613622685	0.000301948	0.002393734

Pim3	203.5169543	-0.400934193	0.146691069	-2.733187487	0.006272462	0.031566342
Pimreg	44.17917156	2.40258702	0.308394639	7.790625106	6.67E-15	3.09E-13
Pink1	606.1849974	-0.731372953	0.092515657	-7.905396486	2.67E-15	1.31E-13
Pip4k2c	420.5290098	-0.215938273	0.102580189	-2.10506799	0.03528539	0.123433065
Pip5k1a	827.0958277	-0.166244347	0.080014105	-2.077688013	0.037738104	0.129418584
Pip5k1c	568.4791085	-0.706526922	0.097576208	-7.240770417	4.46E-13	1.62E-11
Pirb	1834.640157	-0.484726593	0.127985894	-3.787343896	0.000152266	0.001307243
Pitpnb	253.6595275	0.233665226	0.111095621	2.103280245	0.035441279	0.123834058
Pitpnc1	2234.12103	-0.265901975	0.093781987	-2.83532032	0.004577976	0.02431918
Pitpnm1	204.1628881	-0.618314818	0.137362652	-4.501331396	6.75E-06	8.16E-05
Piwil2	13.21193894	1.039029368	0.329582975	3.152557766	0.001618468	0.010090714
Pkd1	157.7844175	-0.38276278	0.147953429	-2.587049058	0.009680181	0.044285963
Pkdcc	155.7898822	-0.702267655	0.169689951	-4.138534144	3.50E-05	0.000355622
Pkhd1l1	1.356747736	-0.64110737	0.297541995	-2.154678605	0.031186993	
Pkib	625.8963888	-0.801705321	0.103802809	-7.723348979	1.13E-14	5.08E-13
Pkm	3627.208508	0.304246101	0.108037397	2.816118392	0.004860775	0.025523321
Pkmyt1	59.49362633	1.403963326	0.216877675	6.473526265	9.57E-11	2.59E-09
Pkp2	12.18620462	-0.974653398	0.342265491	-2.847653132	0.00440429	0.023612756
Pla2g15	1760.419284	-0.369082111	0.084925274	-4.345963141	1.39E-05	0.000157291
Pla2g2d	48.36573824	-4.112791033	0.294479332	-13.96631474	2.50E-44	1.39E-41
Pla2g4a	410.9235386	0.312290527	0.109585107	2.849753359	0.004375314	0.02348367
Plac8	140.4448142	0.59120465	0.239880568	2.46457916	0.013717426	0.058648835
Platr3	4.070173963	1.334413032	0.379535067	3.515914995	0.000438241	0.003308193
Plb1	1.13566691	0.598662267	0.29975112	1.997197765	0.045803704	
Plbd1	42.42831926	-0.900506733	0.23822856	-3.780011647	0.000156821	0.001340212
Plcb4	26.93595543	0.7596601	0.283167195	2.682726366	0.007302471	0.035592136
Plcg2	686.973859	0.176206912	0.086486881	2.037383126	0.041611663	0.13888316
Pld1	263.6880974	0.260406336	0.112898476	2.306553153	0.021079744	0.082725867
Pld3	2437.761512	-0.959772877	0.084154073	-11.40494856	3.95E-30	8.98E-28
Pld4	1257.617746	0.622871227	0.133915208	4.651235919	3.30E-06	4.26E-05
Plec	912.1353396	0.354047219	0.1003195	3.529196419	0.000416824	0.003164022
Plek	7528.521369	0.251475881	0.070202819	3.582133643	0.000340799	0.002643088
Plekha1	631.5664642	-0.596957239	0.082772599	-7.212015151	5.51E-13	1.97E-11
Plekha3	528.7214326	0.425435251	0.093129285	4.568222012	4.92E-06	6.15E-05
Plekha8	77.28173666	0.39606063	0.194754126	2.033644362	0.041987466	0.139713059
Plekhf2	338.3201198	-0.30434502	0.09816028	-3.100490531	0.001932004	0.011662744
Plekhg1	122.8566607	-0.726458585	0.169761318	-4.279293974	1.87E-05	0.000206277
Plekhg3	109.4257101	0.390449489	0.148853072	2.623052936	0.008714573	0.040830346
Plekhh2	21.26900464	-0.733903389	0.294743959	-2.489969233	0.012775415	0.055490784
Plekhj1	165.7293409	-0.276713214	0.128580297	-2.152065438	0.031392201	0.113096504
Plekhm1	788.0453047	-0.387637674	0.097586184	-3.972259796	7.12E-05	0.000670269
Plekhm2	778.2368401	-0.874829715	0.08452151	-10.35037965	4.17E-25	6.52E-23
Plekhn1	44.60259171	-0.753119539	0.25761229	-2.923461223	0.003461633	0.019204707
Plekho1	879.7242969	-0.471375528	0.092561737	-5.09255274	3.53E-07	5.45E-06

Plekho2	981.065427	-0.590101448	0.070477515	-8.372903779	5.62E-17	3.44E-15
Plgrkt	525.4999165	-0.215641385	0.098322865	-2.193196723	0.028293209	0.104169819
Plin3	782.4918601	-0.342211053	0.104503037	-3.274651752	0.001057923	0.006990863
Plk1	278.0904055	1.664397217	0.278205173	5.982624981	2.20E-09	4.90E-08
Plk2	306.4386503	-0.838789979	0.17231773	-4.867694005	1.13E-06	1.61E-05
Plk3	92.52646104	0.472424437	0.161451277	2.926111487	0.003432279	0.019070053
Plk4	98.78096885	0.980898803	0.194897755	5.032889177	4.83E-07	7.32E-06
Plod1	648.7657532	1.00711283	0.134824306	7.469816539	8.03E-14	3.19E-12
Plod2	113.8689644	-0.536901313	0.169511005	-3.167353731	0.001538331	0.009675494
Plod3	745.2009487	-0.28541006	0.105481779	-2.705775946	0.006814501	0.033684193
Plpp2	110.506692	0.321792547	0.145518569	2.211350409	0.027011581	0.100611268
Plpp3	147.7211444	-0.44706079	0.20112904	-2.222756046	0.026232254	0.098354776
Plscr2	44.91491732	-1.666507352	0.247133626	-6.743345218	1.55E-11	4.59E-10
Plscr4	176.7820203	-0.366163625	0.128978757	-2.838945209	0.004526293	0.024095806
Pltp	2900.040295	-0.376758428	0.120559762	-3.125076078	0.001777591	0.010906946
Plxdc1	32.24842554	-1.022207303	0.259018827	-3.946459469	7.93E-05	0.000737957
Plxdc2	844.0849133	-1.246181045	0.121101483	-10.29038631	7.79E-25	1.19E-22
Plxna1	1303.63472	-1.256916038	0.098181537	-12.80195931	1.60E-37	6.31E-35
Plxna2	55.6302894	-0.454205463	0.209581253	-2.167204636	0.030219254	0.109820843
Plxna4	89.11667743	-0.367170728	0.178285487	-2.059453828	0.039450784	0.133748187
Plxnc1	1234.906261	-0.434279947	0.102790669	-4.224896608	2.39E-05	0.000253529
Plxnd1	230.7857288	-1.384951665	0.13745945	-10.07534707	7.10E-24	9.51E-22
Pmepa1	413.3393415	0.213642845	0.107873022	1.980503009	0.047647036	0.153279505
Pmf1	177.1690234	1.20420284	0.177238108	6.794265954	1.09E-11	3.31E-10
Pmp22	5005.434234	0.470858769	0.073422994	6.412960626	1.43E-10	3.80E-09
Pmpca	246.728847	0.325594613	0.134324232	2.423945474	0.01535291	0.064125001
Pnisr	1116.809504	-0.294035784	0.107397376	-2.737830242	0.006184599	0.031249925
Pnkp	196.1208927	0.536898233	0.156409709	3.432640053	0.000597735	0.004291967
Pno1	320.708261	0.299514191	0.143903157	2.081359416	0.03740102	0.128515516
Pnpla2	419.5474001	-0.759872066	0.101318306	-7.499849686	6.39E-14	2.56E-12
Pnpla6	133.4513949	-0.284340499	0.136929646	-2.076544472	0.037843622	0.129543623
Pnpla7	537.4921144	-0.416922746	0.110990467	-3.756383371	0.000172387	0.001459297
Pnrc1	842.182349	-0.239137473	0.114383487	-2.090664302	0.036558167	0.126443717
Poc1a	25.29748399	0.752743114	0.286779848	2.624811747	0.008669689	0.040683558
Poc1b	274.4781456	0.40453903	0.106206165	3.808997612	0.000139531	0.001214586
Pogk	215.2378109	-0.440508363	0.159498857	-2.761827712	0.00574788	0.029379576
Poglut3	264.1833689	0.576074824	0.125071947	4.605947525	4.11E-06	5.22E-05
Pola1	321.1172942	1.023831061	0.150057886	6.822907384	8.92E-12	2.76E-10
Pola2	45.21440766	0.667585951	0.224576151	2.972648463	0.002952424	0.016776609
Pold2	61.99077032	0.557086882	0.204980041	2.717761597	0.006572518	0.032736068
Pole	31.41349954	1.681422425	0.286496402	5.868912879	4.39E-09	9.27E-08
Pole2	51.52398222	0.559436252	0.21770131	2.569742235	0.010177421	0.046059276
Pole3	308.6548961	0.275003867	0.108131753	2.54322953	0.010983305	0.049165442
Pole4	329.7953791	0.340330029	0.127611729	2.666918103	0.007655032	0.037033872

Polg	246.0627031	-0.290204361	0.107464937	-2.700456246	0.006924444	0.034171371
Polq	18.60757944	0.765986025	0.314018883	2.43929925	0.014715776	0.061929237
Polr2a	645.4759245	-0.264761667	0.082457264	-3.210895595	0.00132322	0.008460828
Polr2d	69.44413345	0.48132187	0.18928203	2.542882011	0.010994235	0.049192153
Pom121	241.445692	0.272753437	0.10988721	2.482121788	0.013060263	0.056466542
Pomk	803.7394431	1.022775325	0.121267733	8.434026893	3.34E-17	2.11E-15
Pon3	1084.109982	0.398803646	0.095113997	4.192901772	2.75E-05	0.00028842
Pop5	139.96695	0.383688127	0.158407112	2.422164774	0.015428353	0.064318515
Postn	144.1558434	-2.857980933	0.338278162	-8.448611991	2.95E-17	1.87E-15
Pou3f1	4.318883654	-1.196636422	0.381374167	-3.137696585	0.00170281	0.010524743
Pparg	53.46332007	-1.950898553	0.224191759	-8.701919131	3.26E-18	2.34E-16
Ppat	124.2207734	0.341079934	0.149350426	2.28375602	0.022385874	0.08689726
Ppbp	35.85642696	1.732784328	0.342959716	5.05244275	4.36E-07	6.67E-06
Ppfia4	250.8496685	-0.650457184	0.129797004	-5.011342054	5.41E-07	8.14E-06
Ppfibp2	486.8056796	-0.806512531	0.124700427	-6.46760038	9.96E-11	2.69E-09
Ppia	346.4813648	0.273438943	0.108274461	2.525424204	0.011555871	0.051095743
Ppic	142.7973685	-1.145361049	0.20651616	-5.546108596	2.92E-08	5.45E-07
Ppig	2156.531024	0.219364522	0.103997541	2.109324114	0.034916615	0.122399822
Ppil1	121.1863622	0.61560989	0.170012367	3.620971236	0.000293499	0.002337867
Ppip5k2	722.5562066	0.174213554	0.087238029	1.996990949	0.045826166	0.149210952
Ppm1b	382.4107687	-0.199463733	0.097650255	-2.042634015	0.041088681	0.137541779
Ppm1m	221.2199524	0.335662284	0.117504616	2.856588059	0.004282212	0.023033389
Ppp1r12a	1153.501711	-0.275038146	0.119282441	-2.305772278	0.02112336	0.082832052
Ppp1r14b	195.3123507	0.565439575	0.146053447	3.87145655	0.000108187	0.000972775
Ppp1r18	1623.374698	0.158103468	0.075429856	2.096032999	0.036079264	0.125391735
Ppp1r1a	4.975018372	-0.857052958	0.381379806	-2.247242631	0.024624525	0.093768142
Ppp1r2	351.4641374	0.325927954	0.122938755	2.651140838	0.008022038	0.038413123
Ppp1r21	438.9547201	0.356018587	0.118479713	3.004890688	0.002656763	0.015376029
Ppp1r3b	67.64131566	1.698686719	0.217097578	7.824530964	5.10E-15	2.40E-13
Ppp1r9b	883.6857922	-0.364754311	0.089725262	-4.065235382	4.80E-05	0.000472504
Ppp2ca	1315.163342	0.152127089	0.068235026	2.229457478	0.025783482	0.097194852
Ppp2r2a	1319.540503	0.25779748	0.100169776	2.57360544	0.010064497	0.045658378
Ppp2r3d	114.8387828	-0.470968461	0.154075326	-3.056741606	0.002237571	0.013214017
Ppp4r1l-ps	186.2505302	-0.483962484	0.140514538	-3.444216454	0.000572717	0.00414006
Ppt1	1118.787748	0.532824662	0.088090514	6.048604292	1.46E-09	3.36E-08
Ppt2	267.8952478	-0.261228968	0.105560353	-2.474688275	0.013335251	0.057390914
Pqlc1	770.9584732	-0.568904197	0.115563146	-4.922886038	8.53E-07	1.24E-05
Pqlc2	134.5660182	0.569204051	0.157055528	3.624221686	0.000289833	0.002313576
Pqlc3	789.220807	-0.326256254	0.094152013	-3.465207427	0.000529823	0.003872893
Prag1	59.48563357	0.428335547	0.19803331	2.162946967	0.030545259	0.110654054
Prc1	237.6906376	1.966988813	0.211421284	9.303646163	1.36E-20	1.23E-18
Prcp	426.1536015	-0.616260539	0.130188435	-4.73360432	2.21E-06	2.95E-05
Prdm1	257.3716899	-1.457173562	0.156502698	-9.310852648	1.27E-20	1.17E-18
Prdm2	414.9639081	-0.279639544	0.133897184	-2.088464718	0.036755935	0.126978894

Prdx1	31601.05953	-0.715892592	0.151211407	-4.734382188	2.20E-06	2.94E-05
Prdx2	981.1398652	0.173367699	0.088355162	1.962168302	0.049742893	0.15775393
Prdx4	262.1715526	0.580159262	0.146676903	3.955355283	7.64E-05	0.000712772
Prelid1	488.6062934	0.468446602	0.109173401	4.290849218	1.78E-05	0.00019655
Prelid3b	556.9355763	0.258650611	0.100167333	2.582185263	0.009817687	0.044809618
Prex1	632.5703408	-0.204943933	0.086071402	-2.381092067	0.017261397	0.070487561
Prim1	35.18854182	1.02377144	0.270311542	3.78737598	0.000152247	0.001307243
Prim2	99.4308985	0.415622282	0.180213542	2.306276635	0.02109518	0.082764801
Prkaa1	298.0110266	0.270894375	0.11738335	2.307775133	0.021011647	0.082501774
Prkaa2	12.5824686	0.657550952	0.330606068	1.988925839	0.046709389	0.151233615
Prkab1	151.4852454	0.309443056	0.13897266	2.226646994	0.025970876	0.097680434
Prkacb	1879.299182	0.679727956	0.083340753	8.156009305	3.46E-16	1.90E-14
Prkag1	384.9298477	0.51329139	0.116667185	4.39962096	1.08E-05	0.000125957
Prkag2	252.8327406	0.269636838	0.126661085	2.128805684	0.03327034	0.118034988
Prkar2a	1038.472247	0.300209296	0.077614208	3.867968284	0.000109746	0.000984433
Prkar2b	348.8999713	0.329205553	0.138702319	2.37346827	0.017621907	0.071589594
Prkcb	2925.136944	-0.684565186	0.079678303	-8.5916135	8.58E-18	5.85E-16
Prkcd	3022.60328	-0.348909696	0.063990732	-5.452503612	4.97E-08	8.92E-07
Prkx	464.1780398	-0.318842005	0.086470257	-3.687302623	0.000226644	0.001863695
Prl2c2	3.041473581	-0.99427434	0.374111833	-2.657692841	0.007867756	0.037807051
Prmt2	524.491189	0.445484694	0.116330554	3.829472821	0.000128418	0.001130305
Prmt7	101.0899963	0.317620417	0.155501628	2.042553643	0.041096644	0.137541779
Prnp	283.9409393	-0.680124431	0.115434684	-5.891855096	3.82E-09	8.21E-08
Prox1	46.01866661	-0.707329553	0.23592005	-2.99817481	0.002716018	0.015628561
Prps1	162.5609689	0.748204344	0.155900562	4.799240831	1.59E-06	2.20E-05
Prps2	258.8894277	0.373140895	0.122004764	3.058412492	0.002225131	0.013177796
Prr11	36.85111853	1.767227663	0.294524214	6.000279695	1.97E-09	4.41E-08
Prr13	2007.620396	-0.436349531	0.072055614	-6.055732625	1.40E-09	3.24E-08
Prr14l	253.8787623	-0.348270981	0.113727127	-3.062338674	0.002196148	0.013034103
Prr15	35.77474614	-1.000182773	0.242995996	-4.116046316	3.85E-05	0.000389279
Prr5l	118.7171884	-0.374123845	0.179293375	-2.086657387	0.036919116	0.127366706
Prr9	2.892399107	-1.060534262	0.354344069	-2.992950508	0.002762946	0.015837862
Prrc2b	358.8808956	-0.316231258	0.103434159	-3.057319361	0.002233262	0.013203124
Prrx2	12.2842871	-1.119036022	0.34078853	-3.283666915	0.001024659	0.006816369
Prss23	32.65649123	-1.288578152	0.299729025	-4.299143713	1.71E-05	0.000190599
Prune2	65.52617074	1.274489562	0.206104817	6.183696132	6.26E-10	1.52E-08
Prxl2a	91.95862703	-0.519554342	0.166943891	-3.112149464	0.001857304	0.011289657
Prxl2b	200.6633673	1.267325925	0.130069897	9.743422191	1.97E-22	2.24E-20
Psap	27367.54648	-0.774296725	0.080613557	-9.60504354	7.61E-22	7.86E-20
Psd3	131.39379	1.288500897	0.152790423	8.433126057	3.37E-17	2.11E-15
Psd4	102.0286843	0.324343646	0.157434997	2.060175003	0.039381814	0.133572446
Psen1	640.8178676	-0.293846689	0.083185214	-3.532438928	0.000411745	0.00313181
Psen2	1006.176557	-0.626059206	0.092578421	-6.762474418	1.36E-11	4.05E-10
Psip1	459.9142916	0.398801814	0.108148886	3.687525854	0.000226445	0.001863082

Psma1	1396.797887	0.234242434	0.070837256	3.306768867	0.000943787	0.006342775
Psma2	1658.167479	0.190176681	0.090686714	2.097073234	0.035987093	0.125177219
Psmb10	491.095542	-0.550800429	0.127931835	-4.305421146	1.67E-05	0.000185411
Psmb6	1147.03378	-0.228991469	0.086108378	-2.659340185	0.007829386	0.037683003
Psmb8	892.5864471	-0.321165882	0.132256447	-2.428357104	0.015167401	0.063544719
Psmb9	388.1837336	-0.360730611	0.170854465	-2.11133266	0.034743729	0.12205036
Psmc1	1457.726908	0.211130725	0.077760408	2.715144253	0.006624691	0.032930353
Psmc3ip	11.92837322	0.923661784	0.339214067	2.722946582	0.006470252	0.032344794
Psmd12	461.8882352	0.270660425	0.092631819	2.921894737	0.003479091	0.019287299
Psmd14	528.5970645	0.337359209	0.103479014	3.260170319	0.001113453	0.007303144
Psmd3	716.5648036	0.311363858	0.106106998	2.934432838	0.003341579	0.018676749
Psmd4	886.1495521	0.312004707	0.08647391	3.608079092	0.000308472	0.002436445
Psme1	1252.427252	-0.35758806	0.107040267	-3.340687295	0.000835713	0.005717602
Psme2	210.44795	-0.309940254	0.144590783	-2.143568544	0.032067473	0.114935891
Psme3	266.875817	0.283334215	0.109452739	2.588644345	0.009635455	0.044138974
Psmg2	117.9065839	0.447025503	0.188497674	2.371517339	0.017715215	0.071890816
Pstpip1	187.7627267	0.63244384	0.132228529	4.78296055	1.73E-06	2.36E-05
Ptafr	1820.795944	-1.384645181	0.099869187	-13.86458846	1.04E-43	5.56E-41
Ptbp3	4214.111468	0.39010828	0.111593573	3.495795234	0.000472651	0.003514905
Ptchd1	21.89176737	-1.621989839	0.314958267	-5.149856382	2.61E-07	4.12E-06
Ptdss1	540.7129085	0.255378292	0.097963428	2.606873777	0.009137304	0.042309014
Pter	39.08059363	0.655378516	0.230800204	2.839592447	0.00451712	0.024072595
Ptger4	208.7341532	-0.517070596	0.131212393	-3.94071462	8.12E-05	0.000754429
Ptges	49.57324099	0.42268333	0.215378042	1.962518212	0.049702182	0.157691469
Ptges3l	29.93807738	1.260261631	0.289190817	4.357889528	1.31E-05	0.000150325
Ptgfr	1.372950791	0.733836492	0.319840831	2.294380268	0.021768666	
Ptgir	216.3528266	-0.506381947	0.120686741	-4.195837447	2.72E-05	0.000284908
Ptgis	643.4145129	-0.627934839	0.153599895	-4.08812024	4.35E-05	0.000433065
Ptgs1	1418.599938	0.857070805	0.155677002	5.505442656	3.68E-08	6.75E-07
Ptgs2	142.8433573	-1.113684181	0.210469027	-5.291439785	1.21E-07	2.03E-06
Ptgs2os2	6.821761776	0.816028246	0.372133298	2.192838561	0.028319013	0.10423925
Ptk2b	1008.282473	-0.825354555	0.10389498	-7.944123504	1.96E-15	9.88E-14
Ptma	1798.868118	0.276741816	0.10587741	2.613794727	0.008954282	0.041680181
Ptms	626.6919551	-0.670254979	0.122473907	-5.472634912	4.43E-08	8.03E-07
Ptp4a3	113.9741066	0.642605004	0.156665032	4.101776875	4.10E-05	0.000411828
Ptpn1	3887.651185	-0.242088978	0.074673662	-3.241959356	0.00118711	0.007735542
Ptpn14	51.75372477	-0.804300026	0.224671428	-3.579894575	0.000343733	0.002661713
Ptpn18	1967.92843	0.586650341	0.090196814	6.504113806	7.82E-11	2.14E-09
Ptpn22	217.3114973	-0.371905506	0.141731111	-2.62402166	0.008689826	0.040739837
Ptpn6	1797.934159	-0.358530902	0.117814476	-3.043182068	0.002340907	0.013754888
Ptprc	3377.100581	-0.285009652	0.098817569	-2.884200185	0.003924092	0.02136113
Ptprd	27.28122715	-1.468365764	0.30372447	-4.83453231	1.33E-06	1.87E-05
Ptpre	2963.588799	-0.185894531	0.091614597	-2.029092926	0.042448826	0.140748903
Ptprf	82.5590533	-0.661048772	0.182636775	-3.619472435	0.000295204	0.002347711

Ptprg	25.07094912	-0.743627919	0.333821624	-2.227620577	0.025905827	0.097484642
Ptprn	41.06244186	-0.592264062	0.259293152	-2.284148492	0.022362805	0.086875129
Ptpro	543.2271508	-0.213042846	0.106543121	-1.999592681	0.045544265	0.148486375
Ptprs	175.7793801	-0.556063262	0.127619299	-4.357203551	1.32E-05	0.000150579
Ptprv	6.712314097	-0.95163507	0.377937416	-2.517969986	0.011803338	0.052082532
Pttg1	323.6897814	0.334133354	0.097813039	3.416041012	0.000635387	0.004510525
Pttg1ip	1437.433251	-0.201742794	0.06838182	-2.950240184	0.00317527	0.017873696
Purg	27.22491866	-0.586334325	0.279190799	-2.100120518	0.035718241	0.124553713
Pvr	172.7645716	0.467555171	0.133253149	3.508773899	0.000450177	0.003382981
Pwwp2b	114.6439364	-0.385759423	0.170885225	-2.257418239	0.023981953	0.091740293
Pwwp3a	234.218412	0.341665687	0.125504516	2.722337804	0.006482184	0.03238287
Pwwp3b	18.02565559	-1.03711888	0.342418582	-3.02880432	0.002455237	0.014322196
Pxdn	28.92227231	-0.72898806	0.258142096	-2.823979787	0.004743138	0.025028223
Pxk	533.6807517	0.703643921	0.090164861	7.803970583	6.00E-15	2.79E-13
Pxylp1	27.78637786	0.677227855	0.307160337	2.20480242	0.027467962	0.101705331
Pycr2	294.5433043	0.297144823	0.106584632	2.787876814	0.005305471	0.027483331
Pygb	339.5023347	0.48945661	0.099079337	4.940047274	7.81E-07	1.15E-05
Pygl	1672.147321	0.408779798	0.08670747	4.714470394	2.42E-06	3.21E-05
Pygo2	383.4811576	-0.243835056	0.092521424	-2.635444267	0.008402722	0.03972894
Qk	3725.17539	0.30738139	0.066309286	4.635570779	3.56E-06	4.56E-05
R3hdm2	965.6672541	-0.21606445	0.096951888	-2.228573934	0.025842267	0.09731867
Rab10	2506.945182	0.196088983	0.079789257	2.457586291	0.01398742	0.059447526
Rab11a	2977.224246	0.826076221	0.091654866	9.012900816	2.01E-19	1.65E-17
Rab11b	1014.417721	-0.30384678	0.081862779	-3.711659705	0.000205905	0.00171097
Rab14	5142.346542	0.215863834	0.063702694	3.388613908	0.000702468	0.004916952
Rab19	42.79372788	-0.901749674	0.22390217	-4.027427123	5.64E-05	0.000545618
Rab1a	2509.734579	0.520519035	0.091258993	5.703756058	1.17E-08	2.34E-07
Rab20	298.3323988	0.330319454	0.148458864	2.224989771	0.026081925	0.097926306
Rab24	556.8398605	0.211019315	0.089447785	2.359134043	0.018317638	0.073856204
Rab27a	112.104664	0.598326068	0.209193498	2.860156147	0.004234325	0.0228167
Rab27b	13.25880991	-0.937886904	0.369767349	-2.53642434	0.011199094	0.049930126
Rab29	411.8485233	0.209888589	0.095524685	2.197218325	0.028004857	0.103285548
Rab31	2103.0656	0.35513618	0.088618807	4.007458382	6.14E-05	0.000587414
Rab32	3383.283791	1.226034419	0.095975856	12.77440459	2.28E-37	8.77E-35
Rab3gap1	652.8544946	0.236473996	0.083032113	2.847982403	0.004399736	0.023597868
Rab3il1	485.9165478	0.999964474	0.126221583	7.922293898	2.33E-15	1.17E-13
Rab40c	349.2539943	-0.2430518	0.101105724	-2.403937098	0.016219564	0.067017344
Rab43	48.74315899	-0.598817827	0.229750427	-2.606383957	0.009150382	0.042343444
Rab5c	3047.753186	0.39858297	0.073037642	5.457226699	4.84E-08	8.69E-07
Rab5if	1274.267297	0.374047123	0.096715865	3.867484648	0.000109964	0.000985798
Rab6b	193.3817273	-0.483411631	0.140775875	-3.433909621	0.000594943	0.00427396
Rab7b	2877.502978	-0.431877599	0.119155304	-3.624493283	0.000289529	0.002313576
Rab8b	1656.134467	0.18315547	0.074068956	2.472769691	0.013407051	0.057617302
Rab9	275.8432126	-0.362248548	0.106355229	-3.406024802	0.000659162	0.004652889

Rabac1	767.2982685	-0.339192034	0.094655465	-3.583438453	0.000339101	0.002633599
Rabep1	1040.651637	-0.351595777	0.102338272	-3.435623536	0.000591192	0.004251086
Rabgap1l	219.121743	-0.310332445	0.15078827	-2.058067544	0.03958365	0.134002219
Rabggta	93.47308472	0.418908992	0.157552815	2.658848033	0.007840832	0.037725991
Rac1	2980.592875	0.157348509	0.078399842	2.007000325	0.044749628	0.146370688
Rac2	1311.163393	0.170720346	0.079602223	2.144668068	0.031979396	0.114728066
Rac3	9.832968334	1.164599202	0.362368682	3.213851695	0.001309673	0.008401098
Racgap1	219.9898256	1.716431016	0.200591687	8.556840232	1.16E-17	7.81E-16
Rad18	94.45286735	0.540040821	0.170030463	3.176141563	0.001492481	0.009414738
Rad21	1747.449726	0.41115321	0.087254398	4.712120205	2.45E-06	3.23E-05
Rad23a	299.0534429	0.225665241	0.109625992	2.058501244	0.039542042	0.133891567
Rad51	73.15047781	1.969446745	0.266722149	7.38388901	1.54E-13	5.91E-12
Rad51ap1	59.78451268	2.073754772	0.265261955	7.817761779	5.38E-15	2.52E-13
Rad51c	12.23339596	0.999804	0.343833673	2.907812928	0.00363966	0.020058897
Rad54b	17.22577198	1.31753728	0.353713873	3.724867418	0.000195418	0.001631055
Rad54l	72.5745462	1.907992383	0.229808009	8.302549551	1.02E-16	5.97E-15
Radx	105.1330688	0.562583724	0.196050824	2.86958102	0.00411016	0.02222745
Raf1	283.0092925	-0.378534095	0.171295612	-2.209829493	0.027116998	0.100753847
Ralb	545.8946774	0.496966162	0.095108524	5.225253627	1.74E-07	2.82E-06
Ralgds	258.9781691	0.560065739	0.170981258	3.275597256	0.001054388	0.00697672
Ralgps2	255.9574751	0.88510703	0.301038469	2.940179153	0.003280225	0.018361186
Ramp1	21.18730327	-1.892336318	0.297541387	-6.35990957	2.02E-10	5.30E-09
Ramp3	83.26265411	0.590322829	0.17474219	3.378250155	0.000729487	0.005084723
Ran	992.6652808	0.636364837	0.1162099	5.47599505	4.35E-08	7.89E-07
Ranbp1	729.5780636	0.522827107	0.108246913	4.82994937	1.37E-06	1.91E-05
Ranbp10	259.1110994	-0.25993022	0.123809973	-2.099428785	0.035779119	0.124695134
Ranbp9	456.6033833	-0.315741474	0.101323212	-3.116181062	0.001832097	0.011171998
Rangap1	566.6221009	0.608768501	0.130608748	4.661008623	3.15E-06	4.07E-05
Rap1a	1664.716022	0.303229978	0.074024281	4.096358286	4.20E-05	0.000419985
Rap1b	5404.357433	0.466235635	0.079310931	5.878579765	4.14E-09	8.80E-08
Rap1gap2	152.5139247	-0.305819927	0.151921563	-2.013011984	0.044113369	0.144638261
Rap1gds1	850.9036886	0.449395135	0.074621951	6.022291414	1.72E-09	3.90E-08
Rap2b	2660.154527	0.453726203	0.070172978	6.46582507	1.01E-10	2.71E-09
Rap2c	707.778416	0.339709927	0.087796446	3.869290179	0.000109153	0.000980329
Rapgef3	8.614761322	-1.036618956	0.365138327	-2.838976022	0.004525856	0.024095806
Rapgef5	43.45462867	0.930406177	0.229677292	4.050928008	5.10E-05	0.000497453
Raph1	1029.996403	0.298416846	0.086827506	3.436892982	0.000588428	0.004235271
Rara	129.7015978	0.35524621	0.162767907	2.182532277	0.029070272	0.106560227
Rarg	300.5341259	-0.490528627	0.160204481	-3.061890805	0.002199437	0.013046224
Rasa1	2207.92771	-0.319263124	0.096599475	-3.305019229	0.000949699	0.006376792
Rasa2	355.0130327	0.507028342	0.111823055	4.534202199	5.78E-06	7.11E-05
Rasa3	1602.770237	-0.475567372	0.098030775	-4.851204883	1.23E-06	1.74E-05
Rasa4	521.7851486	-0.916565879	0.098336014	-9.320754844	1.16E-20	1.07E-18
Rasal2	356.8450251	0.416284471	0.119806228	3.474647989	0.000511524	0.003746434

Rasal3	58.97207855	-0.42074676	0.201821538	-2.084746578	0.03709231	0.127758618
Rasgef1a	53.40385336	-0.562001571	0.214879037	-2.615432284	0.008911459	0.041545283
Rasgrp2	406.4679446	0.444157599	0.120889948	3.674065584	0.000238721	0.001950186
Rasgrp4	100.0759073	1.443313326	0.19762476	7.303302101	2.81E-13	1.05E-11
Rassf3	1260.22602	-0.290185766	0.084075749	-3.451479991	0.000557521	0.00404776
Rassf4	2365.979865	-0.233764737	0.118987944	-1.964608586	0.049459557	0.157054502
Rassf5	532.0128206	0.380906744	0.089340314	4.263548286	2.01E-05	0.000218804
Rassf7	15.31229	0.955405275	0.339597925	2.813342503	0.00490294	0.025717727
Rassf8	529.6483715	-0.476283563	0.156108112	-3.050985358	0.002280917	0.013433956
Raver2	1.941444253	-0.719061613	0.345884141	-2.078908884	0.037625726	
Rbbp4	1488.065148	0.336878421	0.085885252	3.922424557	8.77E-05	0.000805602
Rbbp5	315.5773906	0.350050583	0.13180677	2.655786076	0.007912379	0.037984988
Rbbp7	667.0953692	0.300189097	0.104705201	2.866993161	0.00414392	0.022385818
Rbbp8	375.7542802	0.484444732	0.127269188	3.806457312	0.000140972	0.001223575
Rbfox1	21.64192936	-2.084824937	0.319700543	-6.521180473	6.98E-11	1.91E-09
Rbks	56.66095492	0.539622884	0.210982044	2.557672086	0.010537541	0.047460046
Rbl1	167.8347651	0.659589118	0.16765375	3.934234203	8.35E-05	0.000771731
Rbm15	301.5759714	0.242428875	0.105632894	2.295013099	0.021732374	0.084821256
Rbm17	828.2024902	0.203793142	0.082502232	2.470153061	0.013505525	0.057957511
Rbm34	218.7040519	0.362226101	0.113953972	3.178705361	0.001479344	0.009335792
Rbm38	50.48588381	0.542347379	0.223740841	2.423998131	0.015350684	0.064125001
Rbm44	1.683311088	0.786024494	0.325037975	2.418254341	0.015595172	
Rbms1	6025.985786	0.284998194	0.070666777	4.032987029	5.51E-05	0.000535279
Rbms2	241.3010884	-0.265428733	0.11747498	-2.259449055	0.023855466	0.091466269
Rbms3	51.85208399	-1.049783335	0.244608346	-4.291690583	1.77E-05	0.000195951
Rbp1	54.60128159	-0.496080179	0.236805428	-2.094885165	0.036181202	0.125581424
Rbpj	983.5789729	1.243342215	0.1238977	10.03523241	1.07E-23	1.39E-21
Rbpj-ps3	16.29232836	1.097204399	0.31366231	3.498043483	0.000468685	0.003490596
Rbpms2	43.60083987	-0.761348031	0.242427722	-3.140515553	0.001686508	0.010449801
Rbsn	231.8933613	0.284522675	0.117250757	2.426616956	0.015240337	0.063779069
Rcan1	252.0623753	-0.362317724	0.146708137	-2.469649821	0.013524537	0.057989351
Rcan2	7.064447012	-1.340201669	0.371585151	-3.606714817	0.000310098	0.00244671
Rcan3	174.6075815	0.378001257	0.13228236	2.857533364	0.004269477	0.022973126
Rcc1	178.4903133	0.580326117	0.130842152	4.435314689	9.19E-06	0.000108468
Rcc2	785.2093571	0.349651059	0.10604798	3.297102496	0.000976878	0.006538811
Rcn1	191.1284944	-0.549309349	0.139629652	-3.934045098	8.35E-05	0.000771863
Rcn2	413.4765239	0.19588727	0.09657516	2.02834011	0.042525548	0.14095338
Rcn3	93.8574906	-0.803870051	0.168314966	-4.775986759	1.79E-06	2.44E-05
Rcsd1	320.8210756	0.339521105	0.135164858	2.511903684	0.012008186	0.052831089
Rd3	11.82889228	1.411831304	0.357910357	3.944650594	7.99E-05	0.000743064
Rdh10	86.69677096	0.607183823	0.167796999	3.618561877	0.000296245	0.002353491
Rdm1	65.52507111	-0.424508017	0.183113723	-2.318275276	0.020434365	0.08069961
Rdx	1995.57545	0.136616065	0.066932512	2.041101711	0.041240718	0.137931683
Recql	134.3997807	0.645898774	0.154231101	4.187863327	2.82E-05	0.000294485

Recql4	4.652672728	0.79866638	0.377342983	2.116552887	0.034297812	0.120936858
Reep1	5.594397756	-1.282599413	0.380496804	-3.370854629	0.000749354	0.005203866
Reep4	288.5722271	0.515427652	0.140918278	3.657635196	0.000254553	0.002063791
Reep5	1660.350514	0.309267899	0.092789425	3.333008021	0.000859125	0.005852421
Rel	532.9324207	-0.301072696	0.106412772	-2.829290969	0.004665126	0.024738357
Rela	525.5898084	-0.218942353	0.099239602	-2.20619943	0.027370039	0.101515253
Relb	187.4021388	-0.345260409	0.13276836	-2.600472051	0.00930956	0.04293441
Relch	343.4289983	-0.255105686	0.116352033	-2.192533117	0.028341035	0.10426916
Rell1	658.1687107	-0.29671423	0.094558671	-3.13788495	0.001701717	0.010522316
Rem1	13.35415948	-1.541081495	0.344778395	-4.469773969	7.83E-06	9.37E-05
Reps1	193.4657507	0.287266379	0.120710289	2.379800268	0.017322024	0.070696659
Rest	1042.342594	-0.205796134	0.094984317	-2.166632769	0.030262867	0.109885033
Retreg2	612.5023724	-0.240076201	0.100295096	-2.393698309	0.016679462	0.068540166
Rexo1	200.5838172	-0.343625166	0.120789477	-2.844826992	0.004443555	0.023773416
Rexo2	866.0903327	0.195627812	0.079539063	2.459518704	0.013912345	0.059295293
Rfc2	277.2757396	-0.242948854	0.116925029	-2.077817341	0.037726186	0.129407286
Rfc3	174.4207742	0.542172525	0.155746479	3.481122204	0.000499318	0.003674968
Rfc4	44.6757115	0.970155687	0.222986458	4.350738133	1.36E-05	0.000154488
Rffl	271.2280426	-0.268678572	0.125643679	-2.13841695	0.032482918	0.115930094
Rfk	788.6404584	0.299684443	0.096319063	3.111372061	0.001862201	0.011309615
Rftn1	1179.88077	-0.487737657	0.090627107	-5.381807612	7.37E-08	1.28E-06
Rfx1	201.3355407	-0.290923971	0.116759245	-2.491656846	0.012714881	0.05525984
Rfx5	121.5307675	0.44052208	0.161197834	2.732803961	0.00627977	0.031590338
Rgl1	224.9081314	0.98091073	0.148655299	6.59855881	4.15E-11	1.18E-09
Rgl2	366.4044767	-0.237687567	0.114379843	-2.07805467	0.037704324	0.129361864
Rgmb	64.59138226	0.537294461	0.23159456	2.319978767	0.020342026	0.080440786
Rgs1	709.3795652	-0.54506864	0.272863312	-1.997588595	0.045761281	0.149032018
Rgs14	274.6524325	0.313865763	0.105305009	2.980539721	0.002877409	0.016375153
Rgs16	25.04654115	-1.096613445	0.277873285	-3.946451515	7.93E-05	0.000737957
Rgs18	890.1420568	0.70897357	0.102256167	6.933308669	4.11E-12	1.36E-10
Rgs2	3485.42441	-0.707563004	0.111410025	-6.350981476	2.14E-10	5.58E-09
Rgs7bp	15.89081437	2.576389493	0.35520342	7.253278958	4.07E-13	1.49E-11
Rhbdd3	104.1235584	-0.412411981	0.156632211	-2.632995981	0.008463535	0.03997868
Rhobtb1	251.7537456	1.5112798	0.168753888	8.9555258	3.38E-19	2.71E-17
Rhobtb2	62.10221503	0.435408778	0.194404593	2.239704169	0.025110135	0.095228219
Rhobtb3	39.30243012	-0.584159791	0.242064449	-2.413240743	0.015811372	0.065604645
Rhoc	2072.077584	-0.752663186	0.110192549	-6.830436287	8.47E-12	2.64E-10
Rhoj	142.1458183	-0.65218835	0.181020311	-3.602846255	0.000314752	0.002478736
Rhoq	1133.373258	0.37888232	0.103426242	3.663309352	0.000248978	0.002024055
Rhot2	139.5910793	0.347460338	0.137241337	2.531746968	0.011349586	0.050451196
Rhou	54.32148026	-0.51226284	0.239266505	-2.140971799	0.032276312	0.115405492
Rhov	37.73428138	-0.842802598	0.294767254	-2.859213788	0.004246925	0.022868173
Rian	9.410864636	-1.066328245	0.35542358	-3.000161793	0.002698362	0.01555679
Rif1	501.2378795	0.234096493	0.110524844	2.118044096	0.034171333	0.120604233

Rilpl2	1034.19022	-0.547728973	0.101552074	-5.393577433	6.91E-08	1.21E-06
Rims3	57.39575857	-0.828072186	0.198061855	-4.180876651	2.90E-05	0.000302698
Rin3	142.6223462	-1.410224628	0.186835903	-7.547931654	4.42E-14	1.83E-12
Ring1	97.2067887	-0.326877806	0.165767663	-1.971903324	0.048620644	0.154980884
Ripk1	343.2465361	0.361317191	0.140216381	2.576854346	0.009970395	0.045299987
Ripor2	74.83174354	1.260067641	0.26451414	4.763706175	1.90E-06	2.57E-05
Ripor3	1.567782201	-0.699379011	0.323842021	-2.159630208	0.030801308	
Ripply3	25.21003909	-0.710879117	0.294924306	-2.410378195	0.015935992	0.066045685
Rmdn3	279.55962	-0.386684304	0.128735677	-3.003707395	0.002667117	0.015403707
Rmi2	10.15925023	1.445891904	0.364481551	3.966982422	7.28E-05	0.000681432
Rn7sk	31.84078951	-2.44339435	0.29006956	-8.423477299	3.65E-17	2.29E-15
Rnase4	186.8711523	2.557426467	0.223696044	11.43259587	2.87E-30	6.74E-28
Rnasel	966.6615063	0.965403236	0.13509336	7.146193101	8.92E-13	3.11E-11
Rnd3	64.52817189	-0.616393087	0.194656347	-3.166570714	0.001542478	0.009697517
Rnf128	6838.119948	-0.760240366	0.232973915	-3.263199517	0.001101619	0.007247699
Rnf141	914.7950495	1.214506551	0.121383015	10.00557244	1.44E-23	1.85E-21
Rnf144b	820.0688817	-0.595892903	0.103863831	-5.73725135	9.62E-09	1.93E-07
Rnf145	638.1451436	0.555671141	0.090541649	6.137188235	8.40E-10	2.00E-08
Rnf149	1850.246132	-0.795468046	0.102463377	-7.76343774	8.27E-15	3.78E-13
Rnf157	120.478735	0.653560188	0.15255818	4.284006196	1.84E-05	0.000202349
Rnf166	646.7701734	-0.30029086	0.102643466	-2.925572102	0.003438235	0.019096074
Rnf167	200.9781876	-0.62535285	0.146668292	-4.263722177	2.01E-05	0.000218792
Rnf168	368.6208953	0.252975775	0.103181903	2.451745577	0.014216514	0.060250559
Rnf181	437.2842898	-0.199326784	0.09383593	-2.124205354	0.033652976	0.11927969
Rnf19a	292.2780839	0.26153217	0.129090075	2.025966529	0.042768215	0.141526482
Rnf19b	708.8139703	-0.244702013	0.110727296	-2.209952032	0.027108492	0.100753847
Rnf2	561.6313093	0.327510522	0.109332555	2.995544391	0.002739554	0.015727808
Rnf219	134.8170813	0.327999877	0.148895322	2.202889061	0.027602569	0.10210297
Rnf227	75.96925702	0.863157398	0.178494802	4.835756498	1.33E-06	1.87E-05
Rnf40	219.5254335	-0.320962947	0.115467176	-2.779689948	0.005441082	0.028088861
Rnf44	934.8590463	0.179141422	0.087933825	2.037229946	0.041627004	0.13888316
Rnf6	687.5301908	0.228116031	0.101593086	2.245389322	0.02474315	0.09407663
Rnf7	500.5304141	0.279455336	0.110729574	2.523764224	0.011610577	0.051322513
Rnpep	1030.472074	0.318646192	0.081945916	3.888493865	0.000100868	0.000912435
Rnpepl1	297.9344555	-0.289882097	0.111140351	-2.60825249	0.00910058	0.04220408
Rnps1	36.67823496	0.46969408	0.236026295	1.990007422	0.046590118	0.151010345
Rock1	3759.616576	-0.234918352	0.09813756	-2.393765974	0.016676385	0.068540166
Rora	631.6087857	-0.215161189	0.101831329	-2.11291742	0.034607837	0.121686927
Rp1	13.76644548	1.135627788	0.351694288	3.229019711	0.001242153	0.008034912
Rp2	634.8901788	0.243689185	0.094474962	2.579404964	0.009897069	0.045062291
Rp9	767.1526403	-0.237395575	0.079578854	-2.983148946	0.002852991	0.016260858
Rpa1	287.8186539	0.68929213	0.143001203	4.820184141	1.43E-06	2.00E-05
Rpa2	209.0134424	1.283559799	0.166286847	7.718949657	1.17E-14	5.24E-13
Rpe	335.8286288	0.432774172	0.113447323	3.81475877	0.000136316	0.001190742

Rpf2	300.1636747	0.290199073	0.105200209	2.758540831	0.005806005	0.029656473
Rpgrip1l	37.84301663	0.693616585	0.240686068	2.881831056	0.003953717	0.021482051
Rpl22l1	268.3996214	0.418852299	0.153474143	2.729139189	0.00634999	0.031839055
Rpl39l	43.35236983	1.998061072	0.262745537	7.60454808	2.86E-14	1.21E-12
Rpl4	5700.145408	-0.170514372	0.078925354	-2.160451141	0.030737762	0.11109865
Rplp1	6597.421753	0.137213464	0.069357225	1.978358627	0.047888264	0.15378968
Rplp2	1621.318858	-0.157677642	0.074511402	-2.116154532	0.034331667	0.120999371
Rpp14	207.2854857	0.270168868	0.136093255	1.985174571	0.047125052	0.152152681
Rpp25l	122.527454	0.351002264	0.148953521	2.35645496	0.018450305	0.074271386
Rpp38	79.77096618	0.514197031	0.181113243	2.839091291	0.004524221	0.024095806
Rps27	989.833143	-0.289563987	0.117221233	-2.470234966	0.013502433	0.057957511
Rps27a-ps1	15.64461136	-0.750058504	0.327734568	-2.288615781	0.022101686	0.085972007
Rps8	4133.581318	0.202163832	0.060058943	3.366090393	0.000762417	0.005277488
Rptor	114.2275232	-0.3600895	0.155797513	-2.31126603	0.020818165	0.081983318
Rraga	774.3794983	0.382864173	0.108811648	3.518595491	0.000433838	0.003282705
Rragc	2213.147179	0.271016809	0.071010585	3.816569154	0.00013532	0.001183421
Rras2	281.2413227	-0.781532645	0.137922754	-5.666451868	1.46E-08	2.87E-07
Rrbp1	3533.100355	-0.356935164	0.078049867	-4.573168111	4.80E-06	6.01E-05
Rrm1	336.773981	1.095297916	0.150205857	7.291978733	3.05E-13	1.13E-11
Rrm2	210.9330514	1.581890292	0.190223044	8.315976114	9.10E-17	5.41E-15
Rrnad1	108.6975385	-0.407277642	0.168670756	-2.414631034	0.015751156	0.065406088
Rrs1	253.2552392	0.374586248	0.138901662	2.696772981	0.007001498	0.034449668
Rsph9	23.33542372	-0.806690733	0.274079729	-2.943270325	0.003247647	0.018219603
Rspo1	3.877191431	-1.016797771	0.381047193	-2.66843003	0.007620665	0.03691521
Rsrp1	5036.169565	-0.425941873	0.08096362	-5.260904498	1.43E-07	2.38E-06
Rtkn	23.11601177	-1.205766061	0.294962791	-4.087858194	4.35E-05	0.000433267
Rtkn2	3.466822528	0.746777478	0.37966663	1.966929453	0.049191344	0.156434521
Rtl5	139.8594887	-0.79572414	0.151575	-5.249705685	1.52E-07	2.52E-06
Rtp4	242.1001487	1.256219211	0.347115054	3.619028325	0.000295711	0.002350497
Rufy3	720.5160567	-0.438023012	0.088639607	-4.941617246	7.75E-07	1.14E-05
Runx1	1378.574104	-0.403402496	0.13219404	-3.051593671	0.0022763	0.013412028
Runx2	50.89636907	-0.695037553	0.216385104	-3.21203973	0.001317961	0.008430796
Runx3	313.9851777	0.589448099	0.128562209	4.584925114	4.54E-06	5.71E-05
Rwdd1	144.789489	0.414881009	0.154691945	2.681981979	0.00731874	0.035651011
Rwdd2a	31.04049362	0.552207248	0.245777755	2.246774726	0.024654428	0.093834389
Rwdd4a	417.2560555	0.236363276	0.096749988	2.443031565	0.014564463	0.061395759
Ryr1	57.79475778	-1.515149241	0.209135717	-7.244813391	4.33E-13	1.58E-11
S100a1	839.9304116	-0.322272936	0.075866135	-4.247915578	2.16E-05	0.000232284
S100a10	1292.80648	0.243571982	0.083522704	2.916236797	0.003542815	0.019575488
S100a11	2220.847226	-0.260886896	0.119563117	-2.182001453	0.029109425	0.106677692
S100a13	483.3880349	0.415324336	0.102333554	4.058535255	4.94E-05	0.000484042
S100a4	1562.167215	0.987654137	0.233256617	4.234195585	2.29E-05	0.000245169
S100b	3.773654464	1.088620819	0.371260013	2.932232886	0.003365343	0.018767633
S100pbp	364.7283689	-0.291899226	0.096930791	-3.011419016	0.002600298	0.015101651

S1pr1	1411.923503	0.717233414	0.077426268	9.263437745	1.98E-20	1.77E-18
S1pr2	600.4250829	-0.415041149	0.106317856	-3.903776506	9.47E-05	0.00086134
S1pr5	6.001933653	-0.784922348	0.375699081	-2.089231484	0.036686891	0.126798749
Sac3d1	74.177184	0.535700954	0.223452215	2.39738485	0.01651257	0.067966029
Sae1	651.652875	0.65471016	0.12702543	5.154166048	2.55E-07	4.05E-06
Safb2	469.0717625	-0.435892373	0.104998429	-4.151418058	3.30E-05	0.000338473
Samd4	180.504122	-0.596169731	0.155601172	-3.831396148	0.000127418	0.001122162
Samd8	1003.133803	-0.421276679	0.096847548	-4.349895148	1.36E-05	0.000154965
Samhd1	2379.733326	-0.619946438	0.120753045	-5.13400252	2.84E-07	4.45E-06
Samsn1	651.268913	0.841379965	0.112970982	7.447752925	9.49E-14	3.73E-12
Sap25	24.4716733	-0.584070159	0.2869863	-2.035184813	0.041832281	0.139418093
Sapcd2	6.672852802	1.711192477	0.378196574	4.524611256	6.05E-06	7.41E-05
Sar1b	570.9803963	0.245449878	0.094591935	2.594828811	0.009463804	0.043485398
Sardh	43.51149088	-1.122917311	0.22408898	-5.011033163	5.41E-07	8.14E-06
Sarnp	333.942646	0.340398652	0.109836018	3.099153258	0.001940746	0.011710806
Sars	625.6642663	0.241733821	0.097256969	2.485516712	0.012936351	0.056027655
Sash1	3053.974916	0.128779974	0.062507318	2.060238356	0.03937576	0.133572446
Sass6	193.9773404	0.780178679	0.164719002	4.736421848	2.18E-06	2.91E-05
Sat2	11.55917945	0.838790873	0.336554417	2.492289006	0.012692272	0.055193544
Sbno1	1855.75197	-0.141490624	0.071570199	-1.97694886	0.048047411	0.153966418
Scamp1	1140.180151	0.587893318	0.092051647	6.386559461	1.70E-10	4.48E-09
Scamp5	268.5551268	0.744910928	0.110831282	6.72112524	1.80E-11	5.30E-10
Scand1	663.713309	-0.367901	0.087066322	-4.225525904	2.38E-05	0.000253
Scap	216.8609071	-0.402456461	0.118426287	-3.398371001	0.000677884	0.004764875
Scarb1	420.9016444	-0.386469526	0.097032205	-3.982899564	6.81E-05	0.000644993
Scarf1	103.3081287	0.8311607	0.183230569	4.536146491	5.73E-06	7.06E-05
Scarna17	8.18159427	0.967123413	0.359207412	2.692381561	0.007094373	0.03482671
Sccpdh	114.5965399	0.977146213	0.171161354	5.708918468	1.14E-08	2.28E-07
Scd1	171.2773539	0.714750521	0.231012728	3.093987629	0.001974857	0.011902282
Scml4	51.04800816	1.341496742	0.239116436	5.610223894	2.02E-08	3.84E-07
Scn3b	4.85598171	1.848528634	0.380604729	4.856819936	1.19E-06	1.70E-05
Sco2	243.2821595	0.315901688	0.120237007	2.627324939	0.008605911	0.040494348
Scoc	438.3131313	0.921344466	0.113885905	8.090065823	5.96E-16	3.18E-14
Scp2	206.8619846	0.299543122	0.129113979	2.319989858	0.020341426	0.080440786
Scrn1	3.617453199	-0.7647599	0.380553035	-2.009601367	0.044473396	0.145564288
Scube3	3.439642998	-1.430763206	0.375305723	-3.812260565	0.000137702	0.001199355
Sdc1	220.712752	-1.480547261	0.148546026	-9.966926065	2.13E-23	2.71E-21
Sdc2	40.94217129	-0.671185251	0.247823823	-2.708316103	0.006762558	0.033482582
Sdc3	1308.366519	0.667498881	0.092131305	7.24508222	4.32E-13	1.58E-11
Sdc4	339.6556587	0.382569404	0.117764493	3.24859721	0.001159756	0.00757374
Sdf2l1	349.6773139	0.366536062	0.112650447	3.253747059	0.001138936	0.007457251
Sdf4	1073.691503	-0.197288195	0.085477869	-2.308061685	0.020995706	0.082460758
Sdhaf3	58.61453606	0.433580024	0.196032274	2.211778782	0.026981954	0.100525864
Sdhb	1206.068983	-0.340144971	0.081794877	-4.158511928	3.20E-05	0.000329483

Sec11c	1126.599221	0.403722372	0.098485735	4.099297936	4.14E-05	0.000415707
Sec13	494.5972263	0.213495901	0.103935401	2.054121105	0.039963972	0.134882967
Sec14l1	970.3869601	0.690680177	0.083993653	8.22300435	1.98E-16	1.12E-14
Sec22a	104.9689301	0.431492947	0.153972004	2.802411705	0.00507221	0.026503709
Sec22c	158.5590255	-0.294362695	0.14104891	-2.086954772	0.036892223	0.127330087
Sec24b	408.6588916	-0.204578568	0.096394923	-2.122296095	0.033812881	0.119676865
Sec61b	518.7183796	0.222018169	0.100119719	2.217526895	0.026587107	0.099425048
Secisbp2l	1195.288294	0.320925606	0.091227008	3.517879341	0.00043501	0.003287108
Sel1l3	531.3117732	-0.661471697	0.132670833	-4.985810974	6.17E-07	9.20E-06
Selenof	1601.523172	0.264883672	0.126495187	2.094021746	0.036258043	0.12578004
Selenoh	129.8056455	0.752934989	0.166303115	4.527485775	5.97E-06	7.32E-05
Selenok	1797.832676	0.322390944	0.090441658	3.564628834	0.000364371	0.002810551
Selenoo	121.1738385	-0.656315246	0.165851586	-3.957244313	7.58E-05	0.000708041
Selenos	1283.917392	0.162532007	0.079509477	2.044184086	0.040935365	0.137259266
Selenow	130.7364265	-0.555239292	0.151730956	-3.659367266	0.000252839	0.002051001
Selp	202.878589	1.226254387	0.373441935	3.283654757	0.001024704	0.006816369
Selplg	799.5254313	-0.511423006	0.088629408	-5.770353373	7.91E-09	1.62E-07
Sem1	2512.373008	0.227680058	0.079928652	2.848541184	0.004392017	0.02356489
Sema3a	71.82158645	-0.605740673	0.202993894	-2.984033956	0.002844752	0.016226223
Sema3c	20.32978884	0.811446567	0.309660592	2.620438595	0.008781674	0.041093417
Sema4c	93.65778259	0.39871284	0.165992664	2.401990721	0.016306122	0.067263874
Sema4d	233.8659362	-1.532414008	0.129652702	-11.81937582	3.10E-32	8.31E-30
Sema6b	52.33819647	0.805049128	0.213521291	3.770345925	0.000163021	0.00138767
Sema6d	48.94525872	0.768464605	0.212558721	3.615304995	0.000299994	0.002380758
Senp2	504.5109741	-0.261045663	0.094696874	-2.756644996	0.005839771	0.029788393
Sephs1	170.1147815	0.356746604	0.127378524	2.800680939	0.005099491	0.026605487
Sephs2	380.4100778	0.554858706	0.119738661	4.633914414	3.59E-06	4.59E-05
Serbp1	6085.868546	0.193044446	0.079794684	2.419264501	0.015551927	0.06474002
Serinc1	2215.495817	-0.259546845	0.077376937	-3.354317889	0.000795609	0.005479442
Serinc2	25.89746844	-1.229866109	0.268227689	-4.585157164	4.54E-06	5.71E-05
Serinc3	10258.50237	0.601071733	0.08876149	6.771762547	1.27E-11	3.81E-10
Serp1	4578.269233	0.423683514	0.081027016	5.228916668	1.71E-07	2.78E-06
Serpinb10	262.6179956	2.02883627	0.194585624	10.42644483	1.88E-25	3.03E-23
Serpinb12	28.27809972	-0.712679657	0.265582163	-2.683462058	0.007286424	0.035548559
Serpinb1b	23.6665548	-0.742269372	0.314942074	-2.356844107	0.018430982	0.074213511
Serpinb2	2628.56943	0.637407226	0.274158346	2.324960142	0.020074092	0.079633067
Serpinb6a	2605.269197	0.310755076	0.081734685	3.801997592	0.000143534	0.001242224
Serpinb6b	56.33078082	-0.475833198	0.222259752	-2.14088783	0.032283085	0.115405492
Serpinb8	711.3887703	0.494446943	0.105847241	4.671325758	2.99E-06	3.90E-05
Serpinb9	299.7282511	-0.475068795	0.146377394	-3.24550658	0.001172419	0.007646452
Serpinb9b	60.83619785	-0.800367582	0.275704294	-2.902992808	0.003696151	0.020310561
Serpine1	455.7878503	-2.349931153	0.262509114	-8.951807863	3.50E-19	2.79E-17
Serpine2	9.091978312	-1.046065055	0.368369492	-2.839716855	0.004515359	0.024071757
Serpinf1	85.42040123	-0.964104568	0.222177678	-4.339340378	1.43E-05	0.000161253

Serpinh1	323.4261447	-1.15302848	0.158090868	-7.293454027	3.02E-13	1.12E-11
Sertad3	145.7617058	0.546226145	0.169744274	3.217935623	0.001291168	0.008301867
Sesn2	107.5346566	0.317055267	0.157746636	2.009901928	0.04444157	0.145523595
Sestd1	445.7107796	-0.488022084	0.104594628	-4.665842708	3.07E-06	3.99E-05
Setd4	24.72368553	-0.57579148	0.275298439	-2.091517414	0.036481707	0.12629319
Setd7	346.9039829	-0.342787361	0.133014067	-2.577076006	0.009964004	0.045298336
Setx	540.3105755	-0.342850273	0.114521537	-2.993762405	0.002755604	0.015813903
Sf1	1005.069052	0.166414936	0.077662355	2.142800531	0.032129119	0.115074388
Sf3b6	812.961277	0.21054678	0.085248107	2.469811788	0.013518416	0.057986499
Sfmbt1	643.8751256	0.616616581	0.125068322	4.930237899	8.21E-07	1.20E-05
Sfn	24.84805861	-0.62413662	0.279410098	-2.233765439	0.025498511	0.096282481
Sfpq	1710.690165	0.269276224	0.079387769	3.391910712	0.000694071	0.004862706
Sfr1	2471.264822	0.198299049	0.080929801	2.450259941	0.014275312	0.060419961
Sfswap	526.7724696	-0.193449936	0.096162839	-2.011691187	0.0442525	0.144999404
Sft2d1	455.3917412	0.480642093	0.171620269	2.800613793	0.005100552	0.026605487
Sft2d3	210.9646757	-0.295787493	0.121772736	-2.429012453	0.015140012	0.063465409
Sfxn1	940.9434199	0.480922692	0.111163682	4.32625733	1.52E-05	0.000170234
Sfxn5	91.63488599	0.627966261	0.174506116	3.598534402	0.000320016	0.002511754
Sgk1	628.5251458	-0.60836502	0.118208875	-5.146525782	2.65E-07	4.19E-06
Sgms1	691.9562998	-0.671447234	0.107358975	-6.254225477	3.99E-10	1.00E-08
Sgms2	134.8266333	0.425466796	0.155458828	2.73684551	0.006203142	0.03131199
Sgo1	75.47416606	1.384846356	0.216894024	6.384898638	1.72E-10	4.52E-09
Sgo2a	158.739058	1.793037205	0.187218892	9.577223665	9.97E-22	1.01E-19
Sgpl1	4684.530585	0.20961219	0.083985538	2.495812916	0.012566886	0.054775271
Sgpp1	903.1636113	0.453745784	0.085824114	5.286926545	1.24E-07	2.08E-06
Sgsh	319.3832083	0.494657693	0.116534438	4.244734028	2.19E-05	0.000235098
Sgsm2	38.30146202	-0.678640405	0.226240699	-2.999638918	0.002702998	0.015577533
Sh2d1b1	11.37834773	-1.59442321	0.347261906	-4.591414092	4.40E-06	5.55E-05
Sh2d3c	53.13338592	-0.451385991	0.219446017	-2.056934077	0.039692568	0.13421955
Sh3bgrl2	104.6734407	0.812168606	0.190839962	4.255757526	2.08E-05	0.000225421
Sh3bp5	2543.02822	1.613421944	0.109261314	14.76663497	2.40E-49	1.80E-46
Sh3gl1	335.6280674	0.859771311	0.114512033	7.508130728	6.00E-14	2.42E-12
Sh3glb2	199.5298545	-0.265459784	0.120950607	-2.194778434	0.028179495	0.103827568
Sh3kbp1	1289.605849	-0.439765381	0.080812832	-5.441776651	5.28E-08	9.39E-07
Sh3pxd2b	1371.936201	-0.331646244	0.08654158	-3.832218486	0.000126993	0.001119073
Sh3tc1	161.2913037	-0.516956387	0.157556507	-3.281085609	0.001034084	0.006860518
Shank2	1.950939053	-0.803981247	0.352481463	-2.28091781	0.022553311	
Shc1	324.8436287	-0.633529356	0.112110033	-5.650960378	1.60E-08	3.11E-07
Shcbp1	64.97575019	1.702090581	0.248310646	6.85468225	7.15E-12	2.24E-10
Shisa4	24.75092771	-1.501319223	0.281921731	-5.325305076	1.01E-07	1.72E-06
Shisa5	3246.563979	0.122137597	0.060065094	2.033420548	0.042010053	0.139726256
Shisa9	5.249260898	-0.75889605	0.380936486	-1.992185254	0.046350736	0.150461927
Shmt1	22.09630948	0.74989097	0.294647101	2.545047845	0.010926277	0.048946458
Shtn1	769.206911	-0.518751195	0.112927381	-4.593670648	4.36E-06	5.51E-05

Sigirr	3.086455138	0.760283515	0.369219019	2.059166716	0.03947827	0.133776434
Siglec1	84.51347974	2.191395559	0.224932042	9.742478373	1.99E-22	2.24E-20
Siglece	9.455484407	1.018316243	0.368086907	2.766510365	0.005665978	0.029059924
Siglecf	24.77582272	-1.922208344	0.337055168	-5.702948736	1.18E-08	2.35E-07
Sik1	44.27581769	-0.565439675	0.213497901	-2.648455425	0.008086051	0.038633356
Sike1	582.5823917	0.231271749	0.085752071	2.69698149	0.006997116	0.034439396
Sipa1	480.4670171	0.323876212	0.091460078	3.541175784	0.000398348	0.003039151
Sipa1l2	238.418622	0.366928798	0.124012271	2.958810396	0.003088291	0.01748245
Sirpa	6448.664773	-0.352757853	0.105746506	-3.335881875	0.000850293	0.005800157
Sirpb1b	60.28469964	0.496796521	0.243107103	2.043529439	0.041000056	0.1373717
Sirt1	206.4964327	-0.283898876	0.120320178	-2.359528393	0.018298181	0.073817418
Sirt5	23.95997975	0.604273958	0.273412001	2.210122295	0.027096676	0.100753175
Six4	17.95634354	-0.782654742	0.307099583	-2.548537301	0.010817572	0.048560963
Ska1	46.33274851	1.749998801	0.276474756	6.329687473	2.46E-10	6.36E-09
Ska2	49.58270374	1.342334859	0.259803522	5.166730793	2.38E-07	3.80E-06
Ska3	30.60983572	1.500718187	0.296329931	5.064348988	4.10E-07	6.29E-06
Sla	500.4033499	0.498281926	0.090353732	5.514790764	3.49E-08	6.43E-07
Slamf7	706.0614968	-2.137641218	0.240399504	-8.892036738	6.00E-19	4.69E-17
Slamf8	296.2361705	-1.578039486	0.195826262	-8.058364962	7.73E-16	4.09E-14
Slc10a6	30.31041955	1.092944817	0.300541364	3.636587001	0.000276274	0.002220701
Slc10a7	136.5852719	0.410187937	0.14239415	2.880651597	0.003968541	0.0215392
Slc11a1	1817.193388	-0.416241834	0.096891852	-4.295942597	1.74E-05	0.000193085
Slc11a2	364.406362	-0.376224529	0.108098771	-3.480377478	0.000500708	0.003679785
Slc12a4	130.228169	-0.36437275	0.141269164	-2.579280139	0.009900646	0.045064906
Slc12a5	53.34650911	0.957417672	0.268839181	3.561302586	0.00036902	0.002841393
Slc12a7	272.5012782	1.010662967	0.126623885	7.981613956	1.44E-15	7.37E-14
Slc13a3	29.9976187	1.550342244	0.289481364	5.355585669	8.53E-08	1.46E-06
Slc15a2	47.23954975	-0.91466066	0.309650884	-2.953844821	0.003138418	0.017712762
Slc15a3	1197.559659	-0.804735032	0.105768024	-7.608490737	2.77E-14	1.18E-12
Slc15a4	590.8566376	-0.236183477	0.082816306	-2.851895837	0.004345934	0.023351031
Slc16a10	386.5412844	-0.226661931	0.106895627	-2.120404146	0.033971977	0.11995705
Slc16a13	21.28338368	0.581696247	0.284001853	2.048212859	0.040539147	0.13643664
Slc16a2	34.19993901	-0.692017896	0.250046612	-2.767555582	0.005647841	0.028976805
Slc16a3	292.3487977	0.926487278	0.150374652	6.161193152	7.22E-10	1.74E-08
Slc16a6	733.1887649	1.095379658	0.099005393	11.06383828	1.88E-28	3.61E-26
Slc16a9	48.73849795	-0.599570513	0.241644664	-2.481207336	0.013093819	0.056562733
Slc17a5	740.0902576	0.210415677	0.086875868	2.422026755	0.015434214	0.06432137
Slc17a8	2.933319397	-0.808742205	0.367169434	-2.202640334	0.027620109	0.102134376
Slc17a9	50.81477929	0.45426558	0.2050138	2.215780496	0.026706539	0.099722575
Slc18a1	7.835903083	-1.51391175	0.378067786	-4.004339448	6.22E-05	0.000594459
Slc1a2	2.185066326	-0.826410637	0.356857436	-2.315800524	0.020569163	0.081146536
Slc1a5	206.6652963	-0.402473231	0.134852425	-2.984545737	0.002839997	0.016211427
Slc20a1	259.0163165	0.604873168	0.122780672	4.926452679	8.37E-07	1.22E-05
Slc22a17	205.1588773	-0.304957082	0.141501296	-2.15515398	0.031149787	0.112398377

Slc22a18	37.22579206	-0.580923775	0.28212028	-2.059135112	0.039481297	0.133776434
Slc22a4	52.48325206	-0.529138511	0.222392384	-2.379301405	0.017345487	0.070734706
Slc23a2	235.5381909	-0.760477802	0.110346503	-6.891725415	5.51E-12	1.76E-10
Slc25a10	136.1780429	0.563927	0.153974537	3.6624692	0.000249796	0.002028509
Slc25a11	949.0420893	0.232383004	0.094036119	2.471210084	0.013465669	0.057836087
Slc25a13	261.3890603	0.389017762	0.130033068	2.991683324	0.002774439	0.015885542
Slc25a15	119.8199312	0.510939719	0.168299252	3.03590011	0.002398188	0.014025568
Slc25a23	58.72357515	0.507737198	0.210984902	2.406509628	0.016105779	0.066583866
Slc25a24	1012.46483	0.349910622	0.097302102	3.596126039	0.000322991	0.002529818
Slc25a25	65.81467766	-0.769145642	0.20481118	-3.755388955	0.000173072	0.001464092
Slc25a3	5097.014605	0.176220137	0.075997731	2.318755227	0.020408312	0.080617936
Slc25a32	68.0374137	-0.57038263	0.192538875	-2.962428385	0.003052228	0.01729788
Slc25a36	562.8476868	0.257098773	0.088121769	2.917539865	0.003528045	0.019508246
Slc25a4	1417.39639	0.234965156	0.083100237	2.827490806	0.004691436	0.024833998
Slc25a42	9.096567357	0.731875408	0.362156897	2.020879388	0.043292252	0.142827406
Slc25a45	335.8552594	0.349648282	0.100778929	3.469458209	0.000521509	0.00381584
Slc25a51	934.378746	0.182758867	0.082321135	2.220072236	0.026413865	0.098896785
Slc26a2	567.92745	-0.257912073	0.09390217	-2.746603957	0.00602158	0.030549645
Slc26a6	36.69421021	-0.468535879	0.232083402	-2.018825457	0.043505364	0.143333699
Slc27a6	12.3306766	-1.561575559	0.359499901	-4.343744063	1.40E-05	0.000158529
Slc28a2	239.7918	0.686276404	0.165530833	4.145912821	3.38E-05	0.000345531
Slc29a3	859.8730559	-0.374721004	0.09584682	-3.909582022	9.25E-05	0.000842944
Slc2a4rg-ps	27.43784293	-0.669704105	0.260594056	-2.569913201	0.0101724	0.046059276
Slc2a6	112.0949716	-0.326368706	0.150591657	-2.167242937	0.030216335	0.109820843
Slc2a9	121.7519105	0.829295581	0.164901455	5.02903737	4.93E-07	7.44E-06
Slc30a1	549.8678719	-0.671138994	0.089223735	-7.521978281	5.40E-14	2.20E-12
Slc30a2	4.442126901	-1.132149426	0.377479726	-2.999232405	0.002706608	0.015586363
Slc30a5	293.5825109	0.499142932	0.108684889	4.592569737	4.38E-06	5.53E-05
Slc31a1	668.383091	0.723144151	0.120175625	6.017394544	1.77E-09	4.00E-08
Slc31a2	585.7044815	-0.23880785	0.10208442	-2.339317317	0.019319017	0.077045041
Slc35a3	434.049296	0.313144835	0.105258522	2.975006967	0.002929819	0.016654467
Slc35b1	659.0113057	0.23230827	0.105079422	2.210787468	0.027050558	0.10068497
Slc35b4	450.9840629	0.234279671	0.093380718	2.508865597	0.012111956	0.053225219
Slc35c2	238.6530629	0.449328526	0.130495535	3.443248285	0.000574771	0.004150911
Slc35g1	53.44332697	0.949221603	0.290988275	3.262061346	0.001106052	0.007267304
Slc35g2	4.455815164	0.758257385	0.381229783	1.988977299	0.046703709	0.151233615
Slc36a1	842.9969705	-0.330633703	0.104549914	-3.162448345	0.001564485	0.009819416
Slc36a2	57.66790272	-3.270928915	0.277477718	-11.78807776	4.50E-32	1.18E-29
Slc36a4	429.3950625	-0.42213155	0.105904155	-3.985977218	6.72E-05	0.000637494
Slc37a2	385.4117794	-0.477153302	0.133151739	-3.58353039	0.000338981	0.002633599
Slc38a1	824.2045867	0.532298411	0.078567219	6.775070049	1.24E-11	3.73E-10
Slc38a2	907.1580528	0.561931098	0.081731432	6.875336469	6.18E-12	1.96E-10
Slc38a6	320.1513484	0.297922294	0.125000187	2.383374792	0.017154719	0.070128267
Slc39a10	222.0511828	-0.602945334	0.141058055	-4.27444808	1.92E-05	0.000209738

Slc39a12	8.719021779	0.793906919	0.369363068	2.149394421	0.031603147	0.113624445
Slc39a4	42.96372485	-0.526772702	0.259875421	-2.027020103	0.042660357	0.14129419
Slc39a6	444.4159386	0.283060717	0.107115973	2.642563086	0.008228114	0.039199778
Slc39a8	42.1590509	1.925147167	0.26077497	7.382407764	1.55E-13	5.95E-12
Slc41a2	533.2479753	-0.382710239	0.099714443	-3.838062238	0.000124009	0.001095998
Slc43a3	50.68960171	1.520020104	0.252008233	6.031628754	1.62E-09	3.71E-08
Slc44a1	605.529323	-0.293375659	0.125220117	-2.342879614	0.019135556	0.076475977
Slc44a2	199.2187035	-0.448834339	0.145413754	-3.086601694	0.002024587	0.012158466
Slc45a4	345.4708098	0.286611711	0.104774903	2.735499665	0.006228565	0.031387533
Slc46a1	30.38420966	0.652644717	0.271000114	2.408282077	0.016027792	0.066334549
Slc46a3	500.486357	-0.634600405	0.09166877	-6.922754652	4.43E-12	1.45E-10
Slc49a4	331.4469029	-0.431065226	0.12201662	-3.532840243	0.000411121	0.003128646
Slc4a2	212.0883672	-0.328934657	0.144385841	-2.278164222	0.022716795	0.087931633
Slc4a3	5.771125135	-0.7616731	0.375915927	-2.02617938	0.042746406	0.141516725
Slc4a4	9.274814684	-0.794227963	0.35876265	-2.213797794	0.026842692	0.100106431
Slc4a8	3.083510632	0.859732345	0.375019744	2.292498887	0.021876872	0.085274341
Slc50a1	143.4156164	-0.333530908	0.155778522	-2.141058373	0.032269331	0.115405492
Slc5a3	92.49305797	-0.60895346	0.170325815	-3.575227043	0.000349924	0.002705467
Slc5a6	62.20491835	0.407146862	0.201634377	2.019233369	0.04346297	0.143275082
Slc5a7	2.75737043	-0.794644003	0.350781131	-2.265355608	0.023490866	0.09032217
Slc6a4	14.24566999	-1.154663891	0.348789037	-3.310493643	0.000931316	0.006278641
Slc6a8	222.9550531	-0.819233491	0.147916334	-5.538492397	3.05E-08	5.67E-07
Slc7a1	170.2723767	0.340288471	0.138361079	2.459423362	0.013916041	0.059295293
Slc7a11	879.9262171	-0.575760607	0.15768616	-3.651307186	0.000260909	0.002111899
Slc7a2	475.2134209	-0.574963405	0.118142743	-4.866684051	1.13E-06	1.62E-05
Slc7a4	14.99733006	1.120839389	0.32247196	3.475773173	0.000509383	0.003733925
Slc7a7	137.6784764	0.742382085	0.1564113	4.746345583	2.07E-06	2.79E-05
Slc7a8	1148.043653	1.239610266	0.111081728	11.15944349	6.44E-29	1.29E-26
Slc8b1	176.3408617	-0.347403395	0.174231455	-1.993918923	0.046160916	0.150007982
Slc9a3r1	337.35988	-0.372462157	0.109194904	-3.410984804	0.000647287	0.004577679
Slc9a3r2	216.221919	-2.051423535	0.139002133	-14.75821621	2.72E-49	1.91E-46
Slc9a4	34.47448548	-1.491950487	0.252028191	-5.919776193	3.22E-09	6.97E-08
Slc9a7	8.338647931	-0.906704597	0.364226313	-2.48939894	0.012795929	0.055531668
Slc9a8	108.7562603	-0.409365585	0.159812209	-2.561541365	0.010420883	0.046962821
Slc9a9	941.6322292	1.353131377	0.107661163	12.56842612	3.15E-36	1.15E-33
Slco3a1	156.0908217	-0.801007493	0.155088301	-5.164847951	2.41E-07	3.83E-06
Slf1	164.8072233	0.401872314	0.132354541	3.036331885	0.002394756	0.014015534
Slf2	678.888332	-0.179390013	0.086461413	-2.074798535	0.03800521	0.12987856
Slfn3	9.923476018	0.680228233	0.346040853	1.96574545	0.049328022	0.156802716
Slfn4	120.09224	0.704778399	0.151962303	4.637850198	3.52E-06	4.52E-05
Slfn5	230.7615796	0.488106996	0.183058119	2.666404519	0.007666738	0.037078549
Slfn9	93.01416663	1.83209668	0.228079212	8.032721036	9.53E-16	4.97E-14
Slit2	11.12394762	-0.839190188	0.339084066	-2.474873555	0.013328335	0.057377605
Slpi	3932.573104	0.861562106	0.130607245	6.596587398	4.21E-11	1.20E-09

Smad3	397.9942225	0.541926219	0.118386872	4.577587107	4.70E-06	5.90E-05
Smad4	784.8636967	-0.157969261	0.080519192	-1.961883333	0.049776069	0.15782579
Smad7	164.8290774	-0.582513199	0.16118261	-3.613995318	0.000301515	0.002391558
Smad9	4.747105019	-1.130428541	0.381273641	-2.964874618	0.003028063	0.017167414
Smagp	797.4978238	0.446762605	0.077470688	5.766859884	8.08E-09	1.65E-07
Smap2	1858.564941	-0.47490352	0.10498891	-4.523368442	6.09E-06	7.44E-05
Smarca1	4.270107891	-0.883011164	0.380981954	-2.31772438	0.020464305	0.080775336
Smarcb1	280.4545616	-0.352845831	0.122067418	-2.890581593	0.003845297	0.021014702
Smc1b	2.794096323	0.863862485	0.373964118	2.310014369	0.020887359	0.082142712
Smc2	879.1065113	1.282118814	0.16357541	7.838090189	4.57E-15	2.19E-13
Smc3	1049.297126	0.240541026	0.106874917	2.250678021	0.024405938	0.093053839
Smc4	1875.961314	0.561778461	0.132075455	4.253466028	2.10E-05	0.000227414
Smco4	92.57856776	0.445517091	0.19435613	2.292271874	0.02188996	0.085303201
Smg1	699.2195706	-0.299342242	0.141709146	-2.112370664	0.034654669	0.121823055
Smim10l1	654.7162698	0.341180194	0.104569041	3.262726613	0.001103459	0.007256622
Smim14	1242.12628	-0.366329801	0.100642107	-3.639925813	0.000272717	0.00219799
Smim15	960.8619063	0.416462189	0.087815412	4.742472648	2.11E-06	2.84E-05
Smim4	395.2777067	0.4051197	0.112530551	3.60008636	0.000318112	0.002502044
Smoc2	12.00599918	-0.941948782	0.341663094	-2.756952095	0.00583429	0.02977055
Smpd3	61.69173054	-2.203250535	0.217144048	-10.14649288	3.43E-24	4.82E-22
Smpdl3a	6769.536734	0.177781131	0.074891236	2.3738576	0.017603338	0.071572282
Smpdl3b	55.36081855	0.932124949	0.259400547	3.593380818	0.000326415	0.002551307
Smtn	38.28787454	-0.558102145	0.257562454	-2.166861419	0.030245423	0.109874862
Smyd2	152.8535543	0.307110678	0.149862976	2.049276519	0.040435083	0.136239178
Smyd4	54.94634142	0.63852359	0.198286332	3.220209808	0.001280968	0.008250424
Snai1	41.25788126	-1.706100301	0.241583055	-7.062168749	1.64E-12	5.58E-11
Snap29	460.9816512	0.332849552	0.095418912	3.488297479	0.000486107	0.00359537
Snapc1	163.451931	0.315771783	0.136003355	2.321794059	0.020244027	0.080137726
Snhg18	40.67893379	-0.559697421	0.277058823	-2.020139314	0.043368939	0.143041245
Snhg8	333.864604	0.232387669	0.110309697	2.106683952	0.035144984	0.12302794
Snhg9	117.7834565	-0.456688753	0.174787355	-2.612824898	0.00897973	0.041785675
Snord13	56.81403673	3.08893208	0.27674529	11.16164281	6.28E-29	1.27E-26
Snrnp25	169.2672469	0.575031698	0.149785335	3.839038713	0.000123517	0.001093581
Snrnp35	51.39791477	0.493958784	0.202944502	2.433959925	0.014934649	0.062727196
Snrnp40	265.936148	0.233846482	0.110147267	2.123034812	0.033750935	0.119513988
Snrpa1	208.4615711	0.348315543	0.152852325	2.278771633	0.022680644	0.087814351
Snrpb2	2622.586486	0.198307297	0.069736196	2.843678153	0.004459607	0.023850792
Snrpd1	760.4853284	0.253740471	0.117697862	2.155863037	0.031094361	0.112225369
Snrpd3	868.9096713	0.374501184	0.105667447	3.544149074	0.000393882	0.00300814
Snrpe	418.7800644	0.356354765	0.110496177	3.225041569	0.001259544	0.008122894
Snrpf	187.254693	0.394963295	0.178200556	2.216397663	0.02666428	0.099605117
Sntb2	520.8935449	0.26761624	0.110606431	2.41953598	0.015540323	0.064709665
Snx11	262.3654569	0.321142853	0.107836999	2.978039595	0.002900985	0.01649681
Snx13	547.4411214	0.196553469	0.095101874	2.066767577	0.03875606	0.13194469

Snx16	391.6349582	0.207396144	0.101749928	2.038292786	0.041520661	0.13872223
Snx17	385.4014932	0.190615664	0.096326795	1.978843623	0.047833615	0.153712862
Snx18	887.4210889	0.288787884	0.099210417	2.910862512	0.003604326	0.019871461
Snx2	2314.050131	1.753984778	0.096434834	18.18829057	6.39E-74	7.99E-71
Snx20	328.0392	0.374827196	0.116192909	3.225904222	0.001255754	0.008104446
Snx24	456.5246386	0.621639273	0.12321113	5.045317511	4.53E-07	6.88E-06
Snx27	1506.802028	0.189184123	0.079601255	2.376647498	0.017470775	0.071143409
Snx3	2624.493031	0.615282469	0.110905524	5.54780724	2.89E-08	5.40E-07
Snx5	4155.129498	-0.417195394	0.070430465	-5.923507601	3.15E-09	6.82E-08
Snx6	1213.773835	0.811564631	0.089350454	9.082937923	1.06E-19	8.81E-18
Snx8	1318.405444	0.486854629	0.090196218	5.397727739	6.75E-08	1.18E-06
Soat1	5310.661677	-0.540162251	0.099678327	-5.419054145	5.99E-08	1.05E-06
Socs2	119.1141289	-0.885936887	0.242682184	-3.650605389	0.000261623	0.002115396
Socs6	767.8960704	-0.21418306	0.10527138	-2.034580157	0.041893136	0.13954537
Sod2	1537.207522	0.248338592	0.105454464	2.354936738	0.018525859	0.07455553
Soga1	260.1613334	0.403233971	0.142529289	2.829130594	0.004667465	0.024742015
Soga3	1.241724534	0.629301566	0.304123525	2.069230148	0.038524495	
Sorbs3	91.3860324	-0.414103239	0.203234162	-2.037567089	0.041593246	0.138863147
Sord	509.1718916	0.230891755	0.103255791	2.236114335	0.025344283	0.095837473
Sorl1	334.8981439	-1.001434155	0.165322472	-6.057459353	1.38E-09	3.22E-08
Sort1	1673.743462	-0.667898278	0.119788297	-5.57565552	2.47E-08	4.65E-07
Sowahc	553.3148619	-1.467730586	0.120077192	-12.2232254	2.34E-34	7.16E-32
Sox11	13.85870032	-1.229711118	0.365570838	-3.363810754	0.000768742	0.005316367
Sox4	291.8786381	-0.619305365	0.177560953	-3.487846601	0.000486927	0.003599664
Sox5	26.08946361	0.652930151	0.29628787	2.203701933	0.027545313	0.1019163
Sox7	29.97400465	0.747579358	0.288373452	2.592400069	0.009530887	0.04371333
Sp100	449.0313991	0.509772002	0.133713065	3.812432259	0.000137606	0.001199355
Sp140	139.6536913	-0.315794857	0.151086579	-2.090158227	0.036603589	0.126569137
Sp4	173.56968	-0.318651654	0.126802602	-2.51297409	0.011971813	0.052686508
Spaca6	61.07014885	-0.435073641	0.218512861	-1.991066513	0.046473575	0.150685113
Spag5	42.71177489	1.964091015	0.273579463	7.179234118	7.01E-13	2.46E-11
Spag9	1057.288787	-0.278879461	0.097647753	-2.855974166	0.0042905	0.023069703
Sparc	1742.626111	-1.209925697	0.1339317	-9.033900847	1.66E-19	1.37E-17
Spats2	243.6100591	0.590385724	0.114465786	5.157748383	2.50E-07	3.98E-06
Spc24	52.03446865	1.232617916	0.276678524	4.455054553	8.39E-06	9.97E-05
Spc25	191.7234801	1.718540323	0.209465333	8.204414053	2.32E-16	1.29E-14
Spdl1	77.14893708	0.994231991	0.217878831	4.563233558	5.04E-06	6.27E-05
Specc1l	384.2574463	-0.454533192	0.104903288	-4.332878396	1.47E-05	0.000165688
Spef1	30.51097012	0.731851219	0.251835965	2.906063162	0.003660076	0.020127063
Speg	48.6627969	-0.91636375	0.248397113	-3.68910789	0.000225042	0.001852552
Spen	843.7017478	-0.292860686	0.1105863	-2.648254677	0.008090855	0.038644003
Spg21	1287.79549	-0.266645702	0.073456986	-3.629957011	0.000283468	0.002272442
Sphk1	76.8226484	-0.524586558	0.219749867	-2.387198522	0.016977321	0.069516686
Spic	51.80133507	-0.731108001	0.209004034	-3.498056899	0.000468661	0.003490596

Spidr	95.97972951	0.482096562	0.167082312	2.885383587	0.00390937	0.02131054
Spire1	213.181931	0.407398896	0.120274486	3.387242871	0.000705989	0.004936986
Spop	1195.370504	-0.201207217	0.073543695	-2.73588669	0.006221244	0.031371712
Spp1	3421.188724	0.806511443	0.305522209	2.639780081	0.008295984	0.039435488
Sppl2b	131.1707275	-0.324786395	0.137487238	-2.362302136	0.018161834	0.073346243
Spred1	1429.845426	0.719064178	0.096399381	7.459219857	8.70E-14	3.44E-12
Spred2	157.6442151	1.112609546	0.176905608	6.289283641	3.19E-10	8.11E-09
Sprr1a	3.64953902	-0.813979278	0.377165585	-2.158148331	0.030916301	0.111636413
Spry2	96.23612934	0.55437177	0.176806667	3.1354687	0.001715797	0.010583219
Spryd7	372.4331499	-0.371629522	0.115146176	-3.227458647	0.001248951	0.008071924
Spsb1	43.57098008	-0.971305128	0.22322739	-4.351191518	1.35E-05	0.000154317
Spsb2	390.5738832	-0.423044935	0.11893306	-3.557000341	0.000375114	0.002879453
Spsb4	45.98987582	-0.897655877	0.245889162	-3.650652474	0.000261575	0.002115396
Sptan1	599.0795391	-0.343243404	0.102496959	-3.348815483	0.000811578	0.005568978
Sptlc1	618.6768411	0.309883195	0.085491067	3.624743561	0.000289248	0.002313576
Sptssa	1306.79679	0.671579421	0.102779032	6.534206499	6.39E-11	1.76E-09
Spty2d1	369.3733144	-0.31333624	0.118629235	-2.641307087	0.008258683	0.039295513
Sqle	334.3923439	0.444048347	0.116241937	3.820035697	0.000133432	0.001168273
Sqstm1	3928.822864	-0.282207516	0.108599459	-2.598608868	0.009360236	0.04310189
Src	42.03831125	-0.691587071	0.28545652	-2.422740497	0.015403926	0.064266532
Srgap1	16.59469941	-0.932993169	0.306357292	-3.045441363	0.002323391	0.013668023
Srgap2	875.1265788	0.213696602	0.101292201	2.109704403	0.034883825	0.122369893
Srl	4.87449978	0.822913349	0.381365122	2.157809671	0.030942633	0.111704616
Srp19	636.3470966	0.204061539	0.095737557	2.131467993	0.033050607	0.117477606
Srpk2	767.3225549	0.386548243	0.089595982	4.314347961	1.60E-05	0.000178905
Srr	68.12685474	0.453689485	0.181032763	2.506118105	0.012206484	0.053546537
Srrd	77.41983585	0.348074643	0.175150804	1.98728544	0.046890774	0.151689987
Srrm2	3590.203346	-0.224536354	0.089558958	-2.507134511	0.012171438	0.053453042
Srsf1	828.8663178	0.356894137	0.099308958	3.593775871	0.00032592	0.002548767
Srsf2	1831.460026	0.382764048	0.082330796	4.649099309	3.33E-06	4.29E-05
Srsf3	1040.346304	0.483087676	0.091165391	5.29902489	1.16E-07	1.96E-06
Srsf4	299.8433812	0.204357294	0.103698553	1.97068606	0.048759796	0.15525955
Srsf6	778.491906	0.164935727	0.077137185	2.138212938	0.032499465	0.115958029
Srsf7	718.3654995	0.438692428	0.106837468	4.10616646	4.02E-05	0.000404897
Srxn1	1419.767496	-0.717623081	0.16063751	-4.467344396	7.92E-06	9.46E-05
Ss18l2	198.0717182	-0.353326628	0.14323492	-2.466763185	0.013634049	0.058375515
Ssb	3548.873893	0.330530876	0.09403162	3.515103488	0.000439583	0.00331665
Ssbp2	57.42692186	-0.648413403	0.213007294	-3.044090139	0.002333852	0.013718808
Ssbp4	1073.926074	0.508862344	0.100167135	5.080132779	3.77E-07	5.81E-06
Ssc4d	6.348484512	-0.755883726	0.369813098	-2.043961478	0.040957353	0.137259266
Ssh2	2038.248894	1.03054495	0.101096326	10.19369338	2.12E-24	3.00E-22
Ssr3	2255.355285	0.261566159	0.090418618	2.892835186	0.003817816	0.020879723
Ssrp1	952.443126	0.276589929	0.105123801	2.631087589	0.00851121	0.040165951
Ssx2ip	81.9548413	0.409061802	0.168474451	2.42803463	0.015180894	0.063565757

St13	2128.102445	0.264819214	0.075879329	3.490004666	0.000483012	0.003577606
St3gal1	397.519564	-0.214409209	0.100096156	-2.1420324	0.032190875	0.115213084
St3gal2	214.7595202	-0.405391263	0.113347567	-3.576532543	0.000348182	0.002693385
St3gal4	93.16326361	-0.349292629	0.175555283	-1.989644646	0.046630094	0.15107466
St3gal5	1270.087738	0.747533081	0.142229414	5.255826195	1.47E-07	2.44E-06
St3gal6	13.57263704	0.920909373	0.339742456	2.710610219	0.006715952	0.033306774
St5	23.8690676	-0.700544308	0.281397823	-2.489515731	0.012791726	0.055529485
St6galnac6	134.3636617	-0.336997489	0.144529967	-2.331679007	0.019717584	0.07844692
St8sia4	1704.663969	-0.515055777	0.127821709	-4.029485928	5.59E-05	0.00054261
Stab1	867.6655575	1.625779285	0.113687061	14.30047779	2.17E-46	1.25E-43
Stac2	119.2002167	-1.810716134	0.165981705	-10.90913079	1.04E-27	1.84E-25
Stag3	10.39563349	0.773509493	0.355298538	2.177069165	0.029475407	0.107775428
Stam2	456.8833423	0.271246707	0.107213693	2.529963288	0.011407446	0.050603472
Stambpl1	108.5485031	0.441640218	0.162595218	2.716194384	0.006603713	0.032867334
Stap1	1099.536361	-0.672247541	0.246773034	-2.724153164	0.00644666	0.032248343
Stard13	48.87707116	-0.508787291	0.203390328	-2.501531399	0.012365748	0.054071322
Stard4	411.6233342	0.549412975	0.107415372	5.114844981	3.14E-07	4.89E-06
Stard5	206.7043284	-0.319278644	0.14251042	-2.240388068	0.02506574	0.095110383
Stard7	305.0190975	0.507198754	0.127217405	3.986866045	6.70E-05	0.000635513
Stard8	547.9349176	0.455975764	0.090456437	5.040832682	4.64E-07	7.04E-06
Stat4	52.24514333	1.546926109	0.263061211	5.880479693	4.09E-09	8.72E-08
Stat5b	456.9185593	-0.233723702	0.094376028	-2.476515559	0.013267184	0.05719639
Stau2	141.1374452	0.371807328	0.158051782	2.352439958	0.018650701	0.074937366
Steap1	37.53492629	-1.06688737	0.268416761	-3.974741983	7.05E-05	0.00066415
Steap2	56.74276485	-0.696363542	0.222610831	-3.128165594	0.00175901	0.010814203
Stfa3	17.85112879	0.870313658	0.371731116	2.341245112	0.019219544	0.076729902
Stil	29.55546186	1.709424536	0.304220664	5.61902835	1.92E-08	3.68E-07
Stim1	228.429536	0.455134478	0.146457764	3.107615913	0.00188603	0.011421975
Stimate	114.3440898	0.464463367	0.151901074	3.057670063	0.00223065	0.013195948
Stk17b	1346.908051	0.611023971	0.084509689	7.230223865	4.82E-13	1.74E-11
Stk24	1144.246882	-0.345939051	0.082880278	-4.173961046	2.99E-05	0.000310819
Stk3	351.4001673	0.36001804	0.104203459	3.45495287	0.000550389	0.004003727
Stk32c	9.182206103	-0.885028665	0.357203346	-2.477660625	0.013224687	0.057045954
Stk39	47.84622832	-0.579400615	0.214085261	-2.706401235	0.006801681	0.033642989
Stk40	203.2162996	0.370577465	0.137209511	2.700814708	0.006916986	0.034145794
Stmn1	73.06954487	2.403698019	0.249199605	9.645673485	5.13E-22	5.42E-20
Stoml1	204.5424663	-0.445570265	0.130939782	-3.402863945	0.000666835	0.00470363
Stox2	15.01213879	-1.006124148	0.31108187	-3.234274469	0.001219522	0.007922669
Stpg4	1.770214097	-0.87285325	0.345404043	-2.527049894	0.011502516	
Stra6l	63.38994291	-1.798376488	0.262635888	-6.847413369	7.52E-12	2.35E-10
Strap	676.4417366	0.222220908	0.089546611	2.481622767	0.013078565	0.056529385
Strbp	300.6120921	0.224005977	0.113318572	1.976780793	0.048066414	0.153966418
Strip1	273.3747922	0.235107159	0.112211392	2.095216498	0.036151751	0.125527379
Stub1	483.9478827	0.192875058	0.09777313	1.972679602	0.048532077	0.154764319

Stx12	945.6647344	0.237587688	0.074095933	3.206487556	0.001343661	0.008569218
Stx2	680.3448926	0.682566945	0.089153058	7.656124885	1.92E-14	8.34E-13
Stx3	539.9493956	-0.491654389	0.109635902	-4.484428726	7.31E-06	8.78E-05
Stx5a	331.4793525	-0.248026376	0.113846447	-2.178604455	0.029361065	0.107442453
Stx7	1752.673764	0.261166049	0.073818422	3.537952208	0.000403243	0.003071811
Stxbp2	757.578761	-0.234199405	0.092241813	-2.538972266	0.011117864	0.049641708
Stxbp3	421.6335728	-0.453359143	0.130022185	-3.486782982	0.000488868	0.003610453
Sub1	4630.435178	0.197888166	0.077448275	2.55510102	0.010615698	0.047753395
Suclg2	65.95534953	0.641129975	0.1994935	3.213788793	0.00130996	0.008401098
Suds3	406.5473289	-0.200354351	0.096520528	-2.075769328	0.03791529	0.129701928
Sulf1	11.4454538	-0.79584354	0.336217752	-2.367047946	0.01793061	0.072602909
Sulf2	637.9406774	-0.93073465	0.106494807	-8.73971862	2.34E-18	1.72E-16
Sult1a1	119.9346846	0.823716924	0.164698631	5.001358661	5.69E-07	8.53E-06
Sumf1	610.8466539	0.521579391	0.115341765	4.522034071	6.12E-06	7.47E-05
Sumo1	147.2830537	0.282325206	0.134338684	2.101592763	0.035588964	0.124205486
Sumo3	1674.340377	0.434004951	0.095201963	4.558781522	5.15E-06	6.39E-05
Sun1	206.8186249	0.27531735	0.121279973	2.270097382	0.023201675	0.089462369
Supt20	362.7723247	-0.231522006	0.111860326	-2.069741912	0.03847652	0.131171542
Supt5	477.898158	-0.294986227	0.096276247	-3.063956452	0.002184307	0.012992428
Surf4	2132.510163	0.169190342	0.084476027	2.002820778	0.045196529	0.147480823
Susd2	1.4514207	-0.669730742	0.322541911	-2.076414627	0.03785562	
Suv39h1	135.1506109	0.309340903	0.15595886	1.983477584	0.047314109	0.152533368
Suv39h2	16.05020616	1.083491956	0.338724136	3.198744468	0.001380275	0.008773308
Svip	77.25441711	0.822033199	0.178942131	4.593849386	4.35E-06	5.51E-05
Swap70	351.9893947	0.345351358	0.123320689	2.800433253	0.005103406	0.026611124
Swi5	995.883232	0.266418825	0.095154324	2.799860404	0.005112471	0.026649133
Syce2	75.5204719	1.504866647	0.238506288	6.309547061	2.80E-10	7.19E-09
Sycp2	1.974148042	-0.787143956	0.317299659	-2.48075891	0.013110301	
Syde1	16.81896316	-0.766143502	0.305544155	-2.507472288	0.012159812	0.053419875
Syf2	499.3477111	-0.346510841	0.094151724	-3.680345161	0.000232918	0.001906933
Syk	893.1000995	-0.392808349	0.090513087	-4.339796174	1.43E-05	0.00016104
Syn1	18.99481203	-0.727569148	0.288976064	-2.51774883	0.011810751	0.05209
Syncrip	1298.474419	0.322222348	0.085013037	3.790269821	0.000150484	0.001294902
Syne1	201.8413517	-0.420972675	0.186915806	-2.2522048	0.024309333	0.092803398
Syne2	239.5385534	-1.285976577	0.151938323	-8.463806553	2.59E-17	1.65E-15
Syne3	70.60350756	0.733001283	0.190327576	3.851261592	0.000117511	0.001044101
Syngr1	311.7577938	0.636789875	0.14381559	4.427822281	9.52E-06	0.00011204
Synj2	139.3528496	-0.458843457	0.147730163	-3.105956477	0.001896646	0.011481634
Sypl	1179.210239	0.475632985	0.082705694	5.750909765	8.88E-09	1.80E-07
Syt11	371.0880565	-0.977223311	0.119425307	-8.182715487	2.78E-16	1.54E-14
Sytl2	3.271762849	-1.308145198	0.375137862	-3.48710522	0.000488279	0.003607881
Szrd1	875.5996903	0.171081005	0.085926723	1.991010477	0.046479735	0.150685113
Tab2	1714.283198	0.20835517	0.06921701	3.010172923	0.00261099	0.015157882
Tacc2	176.797674	-0.72266501	0.16343232	-4.421799855	9.79E-06	0.000114851

Tacc3	212.0911432	1.278933587	0.201270991	6.354286727	2.09E-10	5.48E-09
Tada1	481.7369857	0.285767863	0.095560593	2.990436254	0.002785793	0.015938388
Tada2b	164.0645947	0.318112187	0.134021508	2.37359057	0.017616072	0.071589594
Taf12	175.6541914	0.394220347	0.123079781	3.202965956	0.001360201	0.008656713
Taf13	189.8712672	0.289947365	0.141697671	2.046239452	0.040732818	0.136923828
Taf5	73.73453936	0.435558519	0.187541469	2.322465108	0.020207905	0.080036957
Taf6l	1015.98664	-0.157819359	0.073701113	-2.141342948	0.032246392	0.115384265
Tafa5	6.401912599	-1.551311756	0.376789098	-4.117188537	3.84E-05	0.000387838
Tagap	31.17733648	0.500678215	0.247253344	2.024960335	0.042871437	0.141805524
Tagln	45.92162541	-1.599284479	0.244395719	-6.543831818	6.00E-11	1.65E-09
Tagln2	4876.001162	0.30089966	0.079886392	3.766594711	0.000165489	0.001404694
Taldo1	4166.194904	-0.443350806	0.127167024	-3.486366129	0.00048963	0.003612529
Tamm41	108.7556269	0.3812474	0.190028462	2.006264727	0.044828011	0.146565134
Tanc2	325.6143672	-0.655391402	0.128120201	-5.115441568	3.13E-07	4.88E-06
Tank	368.7173897	0.584564617	0.097703621	5.983039412	2.19E-09	4.89E-08
Taok1	3302.805917	0.21269673	0.094342038	2.254527615	0.024162995	0.092338699
Taok3	2707.168239	-0.205112996	0.086971976	-2.358380318	0.018354877	0.073986475
Tapbp	2228.853741	-0.800797791	0.104591556	-7.656428721	1.91E-14	8.34E-13
Tapbpl	178.6027785	-0.675193739	0.185766407	-3.634638521	0.000278371	0.002235157
Tardbp	514.2442677	0.26175182	0.09998056	2.618027159	0.008843977	0.041333403
Tars	477.4335975	0.306849147	0.106444242	2.882721884	0.003942554	0.021436921
Tasor	411.4040048	-0.309342058	0.155515543	-1.989139176	0.046685843	0.151222631
Taz	538.3936512	-0.249724368	0.085278496	-2.928339267	0.00340778	0.018950071
Tbc1d1	232.9972387	-0.575682926	0.118289275	-4.866738116	1.13E-06	1.62E-05
Tbc1d10c	37.05050646	0.617413965	0.304578812	2.027107406	0.04265143	0.14129419
Tbc1d12	155.6996979	0.453068192	0.139192072	3.254985606	0.001133981	0.007428047
Tbc1d14	348.012024	0.365702168	0.10189556	3.588990232	0.000331961	0.00259061
Tbc1d16	59.94389769	1.1779349	0.200910136	5.862993885	4.55E-09	9.57E-08
Tbc1d17	194.3445657	-0.33925671	0.142776047	-2.376145833	0.017494547	0.071207124
Tbc1d23	425.7443179	-0.614573492	0.102935573	-5.97046749	2.37E-09	5.23E-08
Tbc1d24	255.8888191	0.462386714	0.115070086	4.018305102	5.86E-05	0.0005639
Tbc1d2b	497.0425196	-0.321053112	0.092147042	-3.484139105	0.000493723	0.003639145
Tbc1d31	235.0455789	0.575377752	0.115339364	4.988563583	6.08E-07	9.07E-06
Tbc1d4	148.2217297	0.542213739	0.142188212	3.813352268	0.000137094	0.001196845
Tbc1d8	161.4840145	-0.835007523	0.146973263	-5.681356638	1.34E-08	2.65E-07
Tbc1d9	295.8626622	-0.249895288	0.110301554	-2.265564527	0.023478059	0.090305083
Tbce	152.5489325	-0.387808055	0.15044814	-2.577685931	0.009946436	0.04524588
Tbkbp1	126.1823246	0.475998854	0.148459685	3.206249922	0.001344771	0.00856942
Tbl1x	688.8251286	0.195352937	0.096236398	2.029927769	0.042363882	0.14054284
Tbl2	73.01180292	0.408464576	0.177125292	2.306077079	0.021106326	0.082786888
Tbrg1	263.0442372	-0.33012871	0.11411664	-2.892905969	0.003816955	0.020879723
Tbx15	2.624203329	0.732526923	0.363071844	2.017581187	0.043634898	0.143602831
Tbx20	4.99309974	-0.870642663	0.38139042	-2.282812095	0.022441439	0.087022914
Tbx6	8.718753326	-0.933536038	0.358061543	-2.607194369	0.009128752	0.042291918

Tbxas1	1539.897634	-0.31329459	0.066744532	-4.693936402	2.68E-06	3.52E-05
Tcea2	12.04171089	-0.710580959	0.335671829	-2.11689185	0.034269028	0.120863759
Tceal8	523.4240475	0.263530456	0.100493283	2.622368864	0.008732087	0.040886871
Tceal9	1946.074336	0.47921707	0.10523715	4.553687251	5.27E-06	6.54E-05
Tceanc	38.1166753	0.53260285	0.229907518	2.316596056	0.020525746	0.080996549
Tcerg1	1021.915108	0.291820235	0.107103056	2.724667685	0.006436624	0.032208873
Tcf12	814.2293382	-0.22224772	0.082131426	-2.706000985	0.006809884	0.033672466
Tcf19	41.32160502	1.928021794	0.275634335	6.994853509	2.66E-12	8.91E-11
Tcf24	8.561454933	-1.141095577	0.373094318	-3.058464099	0.002224747	0.013177796
Tcf25	1481.91686	-0.387213054	0.079138695	-4.892841028	9.94E-07	1.44E-05
Tcf4	2936.40424	0.252706287	0.078835192	3.205501004	0.001348276	0.008588105
Tchh	19.3262054	-0.884625061	0.299538248	-2.953295841	0.003144005	0.017737625
Tcirg1	1363.699737	-0.75785668	0.082530283	-9.182770848	4.20E-20	3.62E-18
Tcn2	451.2476809	0.76852795	0.099944411	7.689554083	1.48E-14	6.54E-13
Tcp11l1	88.7667726	0.329754366	0.165419453	1.993443695	0.046212883	0.150111848
Tcp11l2	339.6702672	-0.251218385	0.127181205	-1.975279166	0.048236476	0.154247773
Tctex1d2	174.987463	-0.374222519	0.152040358	-2.461336737	0.013842038	0.059076751
Tctex1d4	11.55071051	0.882864145	0.348773866	2.531336867	0.011362866	0.050495271
Tdrkh	61.1895491	0.612405929	0.196443105	3.117472255	0.001824091	0.011127698
Tead1	128.5596322	-0.887040373	0.165580983	-5.35713919	8.45E-08	1.45E-06
Tecpr1	502.0470887	-0.485520996	0.094268834	-5.150387195	2.60E-07	4.12E-06
Tedc1	11.67762809	1.21594003	0.347489032	3.499218447	0.000466624	0.003482162
Tedc2	31.13362128	0.50953386	0.251981421	2.022108847	0.043165108	0.142588329
Tef	410.791679	0.348097665	0.158136089	2.201253791	0.027718062	0.102429194
Tenm4	426.1805767	-1.801915394	0.135984335	-13.25090415	4.46E-40	2.09E-37
Tent5a	2389.645451	-0.375174387	0.118969289	-3.153539782	0.001613032	0.010061005
Tent5c	180.322756	-0.864330002	0.158276753	-5.460877773	4.74E-08	8.53E-07
Tep1	700.0710343	-0.340079088	0.098289566	-3.459971395	0.000540233	0.003939394
Tet2	495.3088466	-0.491985414	0.133095267	-3.696490681	0.0002186	0.001809451
Tex30	103.9283086	0.639358258	0.167257647	3.822595089	0.000132055	0.001158236
Tfb1m	35.5448467	0.47214871	0.238465198	1.979948073	0.047709364	0.153379268
Tfe3	1059.104117	-0.179751933	0.069686692	-2.579429853	0.009896356	0.045062291
Tfeb	211.5117599	-1.232664241	0.119876546	-10.28278076	8.43E-25	1.26E-22
Tfec	202.8318066	0.649541426	0.171221439	3.793575321	0.000148494	0.001279979
Tfpi	209.2388025	0.283868471	0.122113038	2.32463688	0.020091385	0.079680606
Tfrc	1553.223719	0.657800596	0.127096151	5.175613811	2.27E-07	3.63E-06
Tgfb1i1	41.82242172	-1.178638667	0.262359554	-4.492455675	7.04E-06	8.49E-05
Tgfb2	1390.2089	-0.695441013	0.162794171	-4.271903655	1.94E-05	0.000211837
Tgfb3	34.32975183	-1.169625884	0.243857475	-4.796350349	1.62E-06	2.23E-05
Tgfbi	1241.813429	0.666090481	0.106941047	6.228576403	4.71E-10	1.16E-08
Tgfbr1	737.7238899	-0.746026942	0.095476279	-7.813741295	5.55E-15	2.60E-13
Tgfbr2	1004.103921	0.696154095	0.115372927	6.033946699	1.60E-09	3.66E-08
Tgfbrap1	256.7759535	0.562162202	0.108295294	5.191012312	2.09E-07	3.36E-06
Tgif1	487.9160373	-0.783496659	0.103724355	-7.553642115	4.23E-14	1.76E-12

Tgm1	4.0589509	1.010612194	0.381309618	2.650371628	0.008040328	0.038463881
Tgtp2	3.655384168	-0.984726774	0.369974411	-2.661607791	0.007776844	0.037502279
Thap3	105.39007	-0.331180469	0.159533581	-2.075929514	0.037900471	0.129701928
Thbd	401.1710814	-0.677029	0.115113597	-5.881399037	4.07E-09	8.68E-08
Themis2	460.8116572	-0.507615053	0.127074116	-3.994637701	6.48E-05	0.000615413
Thoc3	349.1179644	0.233327982	0.110855019	2.104803061	0.035308455	0.123484964
Thoc6	181.6091957	0.410352096	0.128659944	3.18943163	0.001425529	0.009022736
Thoc7	687.5454163	0.329090883	0.091839071	3.583342904	0.000339225	0.002633599
Thrb	100.8173957	-0.435192783	0.164030152	-2.653126739	0.007974992	0.038200034
Ticam1	139.8057241	-0.28028972	0.131479984	-2.131805242	0.033022861	0.117445776
Ticam2	140.0521193	-0.521290156	0.145844659	-3.574283484	0.000351188	0.002713843
Ticrr	16.65791028	1.333496134	0.335144073	3.978874288	6.92E-05	0.000653534
Tifa	660.9477993	-0.258143983	0.086236476	-2.993443109	0.002758489	0.015824407
Tifab	343.6021848	-0.38852225	0.12086571	-3.214495238	0.001306741	0.008387622
Tigar	98.99655164	0.397265877	0.188665475	2.105662825	0.035233651	0.123280812
Timd4	136.7970575	1.38893757	0.183380094	7.574091292	3.62E-14	1.51E-12
Timeless	68.33640233	1.469993422	0.245773284	5.981095261	2.22E-09	4.93E-08
Timm10b	715.6189891	0.242592003	0.08314872	2.917567487	0.003527733	0.019508246
Timm22	153.2985497	0.333735471	0.138660228	2.406857951	0.016090427	0.066538726
Timp1	321.9727396	-0.897434765	0.162836152	-5.511274697	3.56E-08	6.54E-07
Timp3	719.6374923	-1.13557001	0.133609479	-8.499172514	1.91E-17	1.24E-15
Tinagl1	25.21822967	-0.792466923	0.287849009	-2.753064625	0.005904024	0.030065045
Tinf2	88.18936509	0.428329268	0.17365895	2.466496936	0.01364419	0.058402268
Tiparp	864.0238519	-0.211330876	0.106208253	-1.989778289	0.046615364	0.151059549
Tipin	285.9344029	0.884904934	0.158260448	5.591447163	2.25E-08	4.26E-07
Tjp2	293.8397703	-0.671380348	0.143109393	-4.69137863	2.71E-06	3.55E-05
Tk1	201.3886067	2.262048737	0.297102681	7.613693454	2.66E-14	1.13E-12
Tkfc	62.95843863	-0.556945879	0.199507304	-2.79160646	0.00524471	0.027215548
Tle1	202.6038317	-1.118714734	0.168981379	-6.620343249	3.58E-11	1.03E-09
Tle2	7.801956781	-0.735250465	0.372356906	-1.974585276	0.048315231	0.154287898
Tle3	400.2393315	0.595477331	0.115509174	5.155238398	2.53E-07	4.03E-06
Tle5	828.6251213	0.360648081	0.108157612	3.334467847	0.000854628	0.005827078
Tln2	76.43032244	1.043610378	0.202785268	5.146381631	2.66E-07	4.19E-06
Tlnrd1	355.795849	-0.209688646	0.103672452	-2.022607192	0.043113662	0.142481112
Tlr1	229.1253544	0.239883685	0.112533116	2.131671934	0.033033826	0.117445776
Tlr13	2184.074952	-0.239077202	0.107971129	-2.214269736	0.026810229	0.100035084
Tlr2	685.8715113	1.025203528	0.152457435	6.724523017	1.76E-11	5.18E-10
Tlr3	108.5369791	0.435731207	0.149133863	2.921745598	0.003480757	0.019289411
Tlr4	345.2572675	0.32724284	0.12937481	2.529416965	0.01142522	0.050667342
Tlr5	4.005489104	1.247163554	0.380267283	3.279702487	0.001039166	0.006888147
Tlr7	848.5299278	0.195526114	0.075284671	2.597157045	0.009399894	0.0432182
Tm2d2	475.5178868	-0.194230665	0.086788521	-2.237976439	0.025222592	0.095549262
Tm4sf1	100.1177151	-0.663740593	0.195600514	-3.393347899	0.000690439	0.004841783
Tm4sf5	11.720561	-0.96044051	0.344671032	-2.786542586	0.005327362	0.027577688

Tm6sf1	2504.940228	0.340227048	0.075397901	4.512420693	6.41E-06	7.79E-05
Tm7sf2	37.93567043	0.575853617	0.253693525	2.269879052	0.023214922	0.089490455
Tm9sf2	1505.590849	0.293628601	0.081067861	3.622009986	0.000292323	0.002329734
Tm9sf3	2454.775717	0.321424665	0.067913676	4.732841537	2.21E-06	2.95E-05
Tma16	143.8422018	-0.366951658	0.172187532	-2.131116307	0.033079562	0.117524853
Tmbim1	842.0317063	-0.305583146	0.097409026	-3.13711325	0.001706202	0.010541365
Tmbim4	738.3640941	-0.205788282	0.088747196	-2.318814454	0.020405099	0.080617936
Tmc4	8.125088574	-1.00747214	0.368374263	-2.734914574	0.006239647	0.031432822
Tmc6	145.0366284	-0.503143324	0.149341523	-3.369078556	0.000754199	0.005232672
Tmcc1	1595.566709	-0.321267348	0.09768804	-3.288707089	0.001006487	0.006710063
Tmcc3	627.2357089	-0.846479332	0.106923243	-7.916700873	2.44E-15	1.21E-13
Tmco4	79.25379112	-0.522342892	0.175213868	-2.981173226	0.002871463	0.016347514
Tmed3	325.2303759	-0.309001013	0.11370188	-2.717642067	0.006574892	0.03273703
Tmed5	1390.814291	0.359688831	0.070376167	5.110946569	3.21E-07	4.97E-06
Tmed8	95.24598536	0.640883289	0.168893483	3.794600467	0.000147881	0.001276019
Tmed9	1490.380984	0.192887881	0.096655962	1.99561287	0.045976078	0.149634135
Tmeff1	41.87076196	-1.004109757	0.238557959	-4.209080932	2.56E-05	0.000270412
Tmem117	23.728048	1.399800717	0.299126389	4.679629637	2.87E-06	3.75E-05
Tmem126a	481.5144065	0.605002654	0.12276897	4.927976947	8.31E-07	1.21E-05
Tmem126b	392.2078537	0.31553809	0.09291722	3.39590541	0.00068402	0.004801257
Tmem127	554.639079	-0.186256053	0.089537308	-2.080206084	0.037506634	0.128742466
Tmem134	878.3443814	-0.218109992	0.072788132	-2.996504865	0.002730939	0.015696361
Tmem140	976.522624	-0.657768655	0.132162223	-4.97697935	6.46E-07	9.60E-06
Tmem141	262.0725454	0.831316122	0.136876267	6.073486229	1.25E-09	2.93E-08
Tmem147	444.0396273	0.219606493	0.103786004	2.115954801	0.034348652	0.12103081
Tmem14c	1531.297778	0.262477079	0.09907451	2.649289708	0.008066115	0.038562661
Tmem158	43.17810765	-0.930090528	0.237166118	-3.921683817	8.79E-05	0.000807589
Tmem165	1069.041836	-0.613692434	0.108516659	-5.655283157	1.56E-08	3.04E-07
Tmem167	920.8339936	0.256392644	0.086857429	2.951879269	0.003158464	0.017799125
Tmem170b	327.6644739	0.400940538	0.133464864	3.004090565	0.00266376	0.01539278
Tmem171	106.2298336	1.257653204	0.21082998	5.965248409	2.44E-09	5.39E-08
Tmem181b-ps	62.79979438	-0.453426853	0.198619578	-2.282891036	0.022436788	0.087022914
Tmem183a	285.2662936	0.233035517	0.112710154	2.067564535	0.03868099	0.131748891
Tmem184c	443.8253734	0.192210803	0.095465107	2.013414196	0.044071073	0.144563094
Tmem189	918.1397281	-0.2635658	0.110870258	-2.377245299	0.017442484	0.07105303
Tmem200a	6.385840536	-1.149913834	0.376518044	-3.054073644	0.002257567	0.013317335
Tmem202	39.58715727	-1.150144117	0.276804976	-4.155070232	3.25E-05	0.000333796
Tmem205	210.8850539	-0.246560812	0.112714487	-2.187481109	0.028707421	0.105384605
Tmem214	251.5927892	-0.319922023	0.108554688	-2.947104632	0.003207646	0.018021044
Tmem219	341.5544859	-0.274739821	0.106095961	-2.589540819	0.009610403	0.044061885
Tmem230	319.5023167	-0.514386573	0.116584916	-4.412119422	1.02E-05	0.000119639
Tmem237	300.7882186	0.277179868	0.111815509	2.478903607	0.013178692	0.056863894
Tmem238	22.13037971	0.681722388	0.285525572	2.387605366	0.016958541	0.069477702
Tmem241	46.04305534	0.687997483	0.25023127	2.749446473	0.005969601	0.030326948

Tmem246	61.39507689	0.493925496	0.231355611	2.134919023	0.032767625	0.116637512
Tmem256	868.6001901	0.347736203	0.079324436	4.383721074	1.17E-05	0.000134993
Tmem259	321.2449375	-0.272438794	0.117060235	-2.327338513	0.019947255	0.07917177
Tmem260	178.709914	0.310112738	0.129455228	2.395521157	0.016596756	0.068275089
Tmem273	300.7974269	1.504933139	0.131220468	11.46873775	1.89E-30	4.58E-28
Tmem30a	1798.784587	0.243690202	0.082867882	2.94070748	0.003274636	0.018336741
Tmem35a	13.20471643	-1.002343012	0.34708252	-2.887909802	0.003878111	0.021163202
Tmem35b	184.2747508	-0.492835165	0.150154184	-3.282194029	0.001030027	0.006836628
Tmem38b	248.0775819	-0.586710028	0.134335439	-4.367499971	1.26E-05	0.000144745
Tmem47	12.96294612	-0.66366635	0.324099779	-2.047722316	0.040587216	0.136537178
Tmem51	526.1352224	-0.413178172	0.1030466	-4.009624487	6.08E-05	0.00058354
Tmem62	43.7487771	-1.084510913	0.22429042	-4.835297531	1.33E-06	1.87E-05
Tmem63a	550.6795664	0.395558871	0.099297994	3.983553487	6.79E-05	0.000643627
Tmem65	816.4400334	0.523652117	0.092684553	5.649831594	1.61E-08	3.13E-07
Tmem67	43.70809267	-0.668076569	0.239829362	-2.785632938	0.005342333	0.027645652
Tmem70	242.3326477	0.235113276	0.119027609	1.975283523	0.048235982	0.154247773
Tmem8	236.7346138	1.660116105	0.148116835	11.20815269	3.72E-29	7.86E-27
Tmem80	121.8379279	-0.327520704	0.144891833	-2.26044973	0.023793353	0.091298095
Tmem86a	678.1232382	0.462252892	0.096433579	4.793484742	1.64E-06	2.25E-05
Tmem97	172.4819668	0.480422229	0.136334618	3.523846235	0.000425331	0.003225337
Tmem98	15.76002363	-0.801467756	0.325936538	-2.458968734	0.013933675	0.05935358
Tmem9b	649.4950234	0.21153471	0.094636853	2.235225535	0.025402546	0.095975832
Tmf1	1327.332833	0.293544934	0.112809904	2.602120233	0.009264936	0.042771661
Tmod1	23.0581323	-1.132400931	0.28941717	-3.912694364	9.13E-05	0.000835704
Tmod3	1417.920037	0.339862311	0.113949811	2.982561422	0.002858473	0.016281444
Tmpo	604.775782	1.02518225	0.134008992	7.650100457	2.01E-14	8.71E-13
Tmsb10	275.8692246	-0.462319371	0.117399066	-3.938015738	8.22E-05	0.000761549
Tmsb4x	34780.71909	0.360478291	0.083447303	4.319831562	1.56E-05	0.000175005
Tmtc2	22.79786001	-0.767657561	0.294185381	-2.609434765	0.009069194	0.042123611
Tmtc3	147.5020528	0.410470799	0.142337788	2.883779539	0.003929337	0.021380553
Tmx1	1306.518674	0.498959629	0.07179481	6.949800798	3.66E-12	1.21E-10
Tmx2	270.7735385	0.270108124	0.12068724	2.238083533	0.025215608	0.095549262
Tmx3	1179.673404	0.233641065	0.079076838	2.954608105	0.003130664	0.017688962
Tnc	50.18144347	-1.924905321	0.249154574	-7.725747475	1.11E-14	5.00E-13
Tnf	113.070335	0.404654916	0.189232148	2.138404709	0.032483911	0.115930094
Tnfaip1	660.7320032	0.393255428	0.098749398	3.982357719	6.82E-05	0.000646058
Tnfaip2	3349.142478	-0.526544949	0.109115948	-4.825554447	1.40E-06	1.95E-05
Tnfaip3	159.671301	-0.587683722	0.181947748	-3.229958757	0.001238081	0.008021627
Tnfaip8	2473.424943	0.28611316	0.066879709	4.278026384	1.89E-05	0.000207303
Tnfaip8l1	13.67335631	0.954251048	0.333401369	2.862168955	0.004207525	0.022696742
Tnfaip8l3	4.460297419	-0.947103972	0.380869491	-2.486688995	0.012893806	0.055875643
Tnfrsf11a	613.0419242	0.299489883	0.090373016	3.313930386	0.000919944	0.006210347
Tnfrsf13b	368.4650471	0.680056232	0.124438535	5.464997111	4.63E-08	8.34E-07
Tnfrsf1a	2109.880401	0.16089005	0.06234389	2.580686737	0.009860401	0.044963549

Tnfrsf1b	2190.992603	-1.038405673	0.097057233	-10.69890041	1.03E-26	1.76E-24
Tnfsf12	378.6823551	-0.640750405	0.113825326	-5.629242889	1.81E-08	3.49E-07
Tnfsf14	21.38143466	0.930839273	0.345655768	2.692966123	0.007081946	0.034807309
Tnfsf9	5.227406533	1.45089202	0.381008541	3.808030171	0.000140078	0.001217934
Tnfsfm13	142.2435156	-0.376043039	0.142572221	-2.637561766	0.008350442	0.039596967
Tnip1	542.0545374	-0.23440588	0.101704724	-2.304768846	0.021179523	0.08296557
Tnip2	261.1897994	-0.527850574	0.11927186	-4.425608653	9.62E-06	0.000113018
Tnip3	207.1505172	-0.433957764	0.177127076	-2.449979826	0.014286422	0.060444413
Tnks2	1008.070191	0.221369487	0.078282994	2.8278107	0.004686751	0.024817951
Tnnc1	8.838160996	0.886730031	0.369960664	2.396822466	0.016537934	0.068051764
Tnni2	41.14422726	0.590181457	0.239027677	2.469092554	0.013545618	0.05806315
Tnni3	17.59493625	-0.880308193	0.333612084	-2.638717945	0.008322019	0.039517284
Tnnt1	10.1772328	0.948287215	0.34413087	2.755600548	0.00585845	0.029873514
Tnnt2	6.952136857	0.802097565	0.380566175	2.107642815	0.035061897	0.122794374
Tnrc6a	1409.270557	-0.199322478	0.075542681	-2.638541219	0.008326358	0.039517284
Tnrc6c	410.2807604	-0.38354831	0.109193956	-3.512541576	0.000443843	0.00334207
Tns1	830.9502276	-0.288649074	0.117441531	-2.457810895	0.013978676	0.059440585
Tns3	1210.888671	-0.666944029	0.095342725	-6.995227232	2.65E-12	8.91E-11
Tob1	105.3469322	0.423855373	0.189253552	2.239616478	0.025115832	0.095228219
Tob2	535.4452105	0.375853345	0.091757439	4.096162111	4.20E-05	0.000419985
Tollip	367.7379546	0.336602514	0.148575861	2.265526253	0.023480405	0.090305083
Top1	5318.182883	0.32615944	0.102104608	3.19436552	0.001401386	0.00888867
Top2a	1089.596022	2.083851959	0.16538652	12.59988996	2.11E-36	7.93E-34
Top3b	208.925271	-0.312157104	0.113509043	-2.75006374	0.005958367	0.030290386
Topbp1	458.9831994	0.732233519	0.111804256	6.549245495	5.78E-11	1.60E-09
Tor1aip2	626.9627677	0.251567249	0.101196452	2.485929538	0.012921355	0.05597886
Tor3a	495.103172	-0.673734953	0.106824638	-6.306924771	2.85E-10	7.30E-09
Tor4a	186.853204	0.332460411	0.131115087	2.535638106	0.011224266	0.050027491
Tpcn1	307.7517062	-0.258920664	0.100656358	-2.572322992	0.01010186	0.045814025
Tpcn2	150.7572682	-0.443632266	0.157140644	-2.823154177	0.00475537	0.025075135
Tpd52	2242.280221	-0.238477393	0.075186726	-3.171801811	0.001514964	0.00954052
Tpgs1	128.8512138	-0.277214126	0.137047225	-2.022763518	0.043097535	0.142459186
Tpgs2	114.851334	0.351937061	0.148607854	2.368226515	0.017873589	0.072430466
Tph1	31.0548155	-1.257690587	0.341806364	-3.679541165	0.000233654	0.001911911
Tpi1	910.966679	0.436696063	0.105137424	4.153573941	3.27E-05	0.000335528
Tpm1	1064.188472	-1.039156078	0.13121593	-7.919435373	2.39E-15	1.19E-13
Tpm2	99.55166616	-1.334586104	0.173811337	-7.678360489	1.61E-14	7.11E-13
Tpm3	1861.983698	0.184417914	0.081948945	2.250400104	0.024423558	0.093097369
Tpmt	9.174686943	0.851621057	0.352121113	2.418545852	0.015582682	0.064832077
Tpsab1	20.09883996	-1.304322993	0.339832723	-3.83813243	0.000123974	0.001095998
Tpst2	568.1617918	0.268066096	0.09064643	2.957271431	0.003103748	0.017556706
Tpx2	253.9536934	1.685454127	0.189685605	8.885514149	6.36E-19	4.95E-17
Tra2a	1114.432155	-0.274853499	0.084250346	-3.262342682	0.001104955	0.007263275
Trabd2b	14.79206468	-1.378763894	0.324529888	-4.248495887	2.15E-05	0.000231948

Traf3ip3	113.6316411	0.629069571	0.199927849	3.146482965	0.001652469	0.010272825
Traf4	18.80659942	-0.667019832	0.30095599	-2.216336788	0.026668445	0.099605117
Traf5	185.5898121	-0.829761713	0.165806741	-5.004390715	5.60E-07	8.40E-06
Traf7	291.2002659	-0.242225157	0.103833086	-2.332832096	0.019656959	0.078226462
Trafd1	764.1206755	-1.019793711	0.107948642	-9.447026737	3.49E-21	3.40E-19
Traip	16.93177229	0.909332057	0.320563012	2.836671803	0.004558645	0.024242232
Trak1	166.7236241	-0.275578155	0.13269569	-2.076767942	0.037822982	0.129532062
Trak2	245.0139321	0.298749226	0.112056386	2.666061595	0.007674563	0.037104435
Tram1	2062.763035	0.17521974	0.065767422	2.664233052	0.007716408	0.037287275
Tram2	126.1150132	-0.418424488	0.148006817	-2.82706227	0.004697719	0.024849726
Trappc10	877.9165766	0.575381854	0.076249508	7.546040198	4.49E-14	1.85E-12
Trappc4	752.433508	0.322103602	0.090313352	3.566511424	0.000361765	0.0027927
Trbc1	8.180518806	-0.783068415	0.375471268	-2.085561483	0.037018363	0.127621084
Trem1	7.147591057	1.147271861	0.375207115	3.05770284	0.002230406	0.013195948
Trem2	3139.247637	-0.683123615	0.101511249	-6.729536091	1.70E-11	5.02E-10
Trem3	6.263710663	1.846557631	0.380698634	4.850444593	1.23E-06	1.75E-05
Treml2	23.38242622	0.62205807	0.310245258	2.005052627	0.044957421	0.146858842
Treml4	20.94231266	1.189531874	0.312478564	3.806763124	0.000140797	0.001222771
Trerf1	124.4260049	-1.156308364	0.152291027	-7.59275439	3.13E-14	1.32E-12
Trib1	618.2652424	1.033363477	0.093496926	11.05237921	2.13E-28	4.06E-26
Trib2	51.85955386	-0.469529739	0.226591451	-2.072142339	0.038252169	0.130525306
Trim25	790.1476671	-0.171603051	0.082030747	-2.091935723	0.036444266	0.126192686
Trim3	121.3054252	-0.427383753	0.16128685	-2.649836314	0.008053078	0.038512599
Trim30a	1489.470744	0.496921399	0.120264404	4.131907561	3.60E-05	0.000365044
Trim30b	18.08824615	0.773016507	0.345012883	2.240543891	0.025055635	0.095096082
Trim30c	73.32459752	2.311566587	0.246024178	9.395688691	5.68E-21	5.47E-19
Trim30d	540.9164234	0.469504791	0.10640986	4.412230147	1.02E-05	0.000119639
Trim36	65.80124358	-1.165789376	0.193625939	-6.020832664	1.74E-09	3.92E-08
Trim37	179.5868081	0.410847595	0.15229064	2.697786259	0.006980224	0.034378807
Trim44	401.9335258	-0.250052727	0.093549475	-2.672946352	0.007518827	0.036492575
Trim59	144.1220647	1.156004875	0.178777754	6.466156156	1.01E-10	2.71E-09
Trim8	582.845666	0.246427922	0.105048513	2.345848753	0.018983809	0.076011212
Trip13	101.7940924	0.944804069	0.199441527	4.737248472	2.17E-06	2.91E-05
Trmo	23.38012727	0.566694184	0.281459455	2.013413209	0.044071177	0.144563094
Trmt10a	84.00293882	0.394977896	0.199768087	1.977182153	0.048021044	0.153966418
Trnp1	17.2839326	-0.9587067	0.308001566	-3.112668264	0.001854043	0.011283853
Troap	24.29655114	1.89353331	0.322545779	5.870587774	4.34E-09	9.19E-08
Trpc4	32.86892842	-1.399498001	0.317773003	-4.404080866	1.06E-05	0.000123681
Trpc4ap	390.7358157	-0.283018348	0.101893062	-2.777601755	0.005476169	0.028231147
Trpm2	234.426223	0.341936726	0.140445823	2.434652163	0.014906111	0.062659946
Trpv4	130.092334	0.550813132	0.14836177	3.712635219	0.000205112	0.001705331
Trrap	385.3023442	-0.334427953	0.12152074	-2.75202368	0.005922824	0.030138317
Tsc1	318.4326332	-0.492725656	0.118988194	-4.140962539	3.46E-05	0.000352381
Tsc2	155.9562794	-0.609196902	0.135048763	-4.510940265	6.45E-06	7.84E-05

Tsc22d1	389.5326528	-0.875632923	0.126727361	-6.909580684	4.86E-12	1.58E-10
Tsc22d3	360.3933488	0.360272054	0.1134605	3.175308181	0.001496774	0.00943333
Tsc22d4	663.3305168	0.303118709	0.114041324	2.657972548	0.00786123	0.037805947
Tsfm	104.6535303	0.338658035	0.166707001	2.031456577	0.0422087	0.140135481
Tshz2	33.06176526	-0.863219318	0.240268705	-3.592724728	0.000327238	0.00255641
Tsku	52.0002694	-0.501370565	0.205608545	-2.438471442	0.014749524	0.062036465
Tslp	12.40595017	1.335973535	0.379483815	3.520502016	0.000430731	0.00326134
Tspan11	3.447502262	1.00803255	0.376454994	2.677697374	0.007413016	0.036060659
Tspan13	484.9647055	-1.105291236	0.107430911	-10.28839119	7.95E-25	1.21E-22
Tspan14	1114.176317	0.618420108	0.117870341	5.246613378	1.55E-07	2.56E-06
Tspan17	347.7102727	-0.399951022	0.117292116	-3.409871319	0.000649935	0.004594244
Tspan18	12.89214954	0.879009531	0.355125306	2.475209499	0.013315804	0.057340107
Tspan3	756.0004393	0.449907425	0.0953805	4.716974911	2.39E-06	3.17E-05
Tspan32	148.1480078	0.929880595	0.172491177	5.390887874	7.01E-08	1.22E-06
Tspan4	667.5896098	0.247225297	0.081415341	3.03659351	0.002392679	0.014015196
Tspan5	392.9720378	-0.57768359	0.122960968	-4.69810542	2.63E-06	3.45E-05
Tspo	896.8742804	0.248694468	0.078994979	3.148231309	0.001642617	0.010224283
Tspyl2	60.29825244	-0.429719157	0.206447811	-2.0814905	0.037389033	0.128515166
Tst	13.92595992	-0.718239056	0.330390038	-2.173912564	0.029711702	0.108408343
Tstd3	186.4687132	0.323516057	0.143904776	2.248125919	0.024568162	0.09357726
Ttc21b	61.93486709	0.561066938	0.200006871	2.805238308	0.005027939	0.026299854
Ttc3	916.5780869	-0.233721288	0.094471344	-2.473991337	0.013361294	0.057437646
Ttc30a1	13.68641031	0.719176542	0.323162967	2.225429942	0.026052389	0.097888885
Ttc32	246.200759	0.839809738	0.13973408	6.010056645	1.85E-09	4.17E-08
Ttc8	40.56132163	0.664564997	0.219357727	3.029594647	0.002448822	0.014299403
Ttc9	26.14397121	-0.65817526	0.276817804	-2.377647862	0.017423456	0.070994788
Ttf2	211.230595	0.64596624	0.154813325	4.172549361	3.01E-05	0.000312389
Ttk	94.85633555	1.702991056	0.224307496	7.592216431	3.14E-14	1.32E-12
Ttyh2	231.3358354	-0.389557866	0.153975535	-2.529998452	0.011406303	0.050603472
Tuba1b	2354.506643	0.641277444	0.121124348	5.294372713	1.19E-07	2.00E-06
Tuba1c	1599.18169	0.316503825	0.128768924	2.457920866	0.013974396	0.059440585
Tuba4a	452.2170126	0.417437072	0.11482311	3.635479571	0.000277464	0.00222907
Tubb2a	803.6975834	-0.23827976	0.112952761	-2.109552324	0.034896935	0.122369893
Tubb4b	221.1974947	0.632609565	0.172709088	3.662862055	0.000249413	0.002026496
Tubb5	3276.68206	0.529580615	0.097305155	5.442472316	5.25E-08	9.38E-07
Tubd1	14.51821627	0.782139361	0.312353748	2.50401785	0.012279184	0.053740399
Tube1	10.67902719	0.757363791	0.34947417	2.167152411	0.030223235	0.109820843
Tubg1	127.0284354	0.843832256	0.178997643	4.714208753	2.43E-06	3.21E-05
Tubgcp4	400.7029682	-0.492017334	0.092433804	-5.322915531	1.02E-07	1.74E-06
Tubgcp5	233.187256	0.25099471	0.126554311	1.983296406	0.047334331	0.152565786
Tuft1	46.77644135	0.473124081	0.229791683	2.058926048	0.039501323	0.133809932
Tusc1	480.5821149	-0.903441443	0.105484642	-8.564672823	1.08E-17	7.36E-16
Tut7	1019.104125	0.232425351	0.106989854	2.172405533	0.029825088	0.108742735
Twf1	1279.396662	0.688971362	0.074910586	9.197249715	3.67E-20	3.24E-18

Twist1	15.90725161	-0.872376982	0.312883254	-2.788186873	0.005300396	0.02746652
Twist2	23.49759302	0.617265959	0.275922494	2.237099089	0.025279865	0.095656815
Txk	20.24776192	-0.657668961	0.28249481	-2.328074487	0.019908149	0.079079298
Txlnb	5.594211193	1.42202408	0.381390444	3.728525724	0.000192603	0.001608457
Txn1	8160.863873	-0.364266628	0.100074563	-3.639952207	0.000272689	0.00219799
Txndc12	196.2076692	0.251578469	0.121458231	2.071316759	0.038329204	0.130758437
Txndc17	871.385837	0.183401949	0.085306978	2.149905598	0.031562683	0.113583867
Txnip	1899.804149	0.608040553	0.104829256	5.800294462	6.62E-09	1.37E-07
Txnrd1	2532.366592	-0.593180781	0.128807902	-4.605158325	4.12E-06	5.24E-05
Tyms	137.2214416	1.06240872	0.195822918	5.425354359	5.78E-08	1.02E-06
Tyrobp	13628.43681	-0.338352621	0.081217292	-4.166017028	3.10E-05	0.000320364
U2af2	787.249554	0.2986642	0.103020752	2.899068333	0.003742733	0.020536452
U90926	2.629374053	-0.765081575	0.35202287	-2.173385991	0.029751278	0.108499984
Uaca	116.3694804	0.545013958	0.164115388	3.320919295	0.000897215	0.006073298
Uap1	394.2838193	0.493433184	0.130945681	3.768228028	0.00016441	0.001397117
Uba2	392.2243552	0.257222456	0.103070944	2.495586485	0.01257491	0.054794329
Uba3	264.9296941	0.331175719	0.122183554	2.710477055	0.00671865	0.033309143
Uba5	655.725057	0.220324934	0.08990786	2.450563658	0.014263274	0.06039756
Uba6	924.7751018	-0.187586084	0.082684683	-2.268692059	0.023287059	0.089722437
Ubap1	536.2071304	-0.215297199	0.095432833	-2.256007627	0.024070154	0.092030775
Ubash3b	1408.538354	-0.31111265	0.11089057	-2.805582572	0.005022571	0.026280936
Ubc	5964.42306	-0.170376378	0.064205842	-2.653596196	0.007963907	0.038159115
Ube2a	592.3308027	0.329151307	0.117203961	2.808363336	0.004979401	0.02610055
Ube2c	137.6265052	2.089337029	0.22265839	9.383598929	6.38E-21	6.06E-19
Ube2e3	612.5725926	0.415135601	0.098865347	4.199000072	2.68E-05	0.000281943
Ube2j1	625.8049069	0.20107716	0.099861759	2.013555153	0.044056259	0.144563094
Ube2j2	461.8677456	0.205092504	0.096057085	2.135110638	0.032751974	0.11660946
Ube2n	164.2391188	0.438327967	0.130690767	3.353932178	0.000796719	0.005484569
Ube2q1	1319.064541	0.17725063	0.068865318	2.573873692	0.010056698	0.04563679
Ube2t	26.35761589	1.417824266	0.320905208	4.418202735	9.95E-06	0.000116503
Ube2w	274.8007873	0.345995646	0.1070608	3.231767806	0.00123027	0.007975231
Ubl3	2430.283109	-0.183648697	0.066477172	-2.762582874	0.0057346	0.029331678
Ubl4a	202.236434	0.321121149	0.129307028	2.483400597	0.013013465	0.05631288
Ubox5	27.09588608	-0.603672271	0.290078429	-2.081065712	0.037427892	0.128530977
Ubtd1	182.5593286	0.271675368	0.136969112	1.983479079	0.047313942	0.152533368
Ubxn2a	195.9128927	0.339549494	0.163108118	2.081744899	0.037365777	0.128464655
Ubxn6	370.4394674	-0.342024425	0.092921331	-3.68079559	0.000232507	0.001904606
Uchl1	57.5838419	-0.621469823	0.255537709	-2.432008278	0.015015364	0.063013303
Uchl3	119.03061	1.000496968	0.201017382	4.977166436	6.45E-07	9.60E-06
Uchl5	382.7463015	0.502780871	0.138738589	3.623943955	0.000290145	0.00231475
Ucp2	1663.710848	-0.475637898	0.118882956	-4.000892254	6.31E-05	0.000601654
Ufsp2	671.9695877	0.336369234	0.096182276	3.497206023	0.000470159	0.003499836
Uhrf1	85.31481564	1.869508538	0.22261169	8.398069924	4.54E-17	2.80E-15
Uhrf1bp1	83.30217633	-0.416739866	0.174164776	-2.392790753	0.016720774	0.068634753

Uhrf1bp1l	994.7274645	-0.254861407	0.077864115	-3.273156143	0.001063537	0.007024868
Ulbp1	4.546384438	0.92399299	0.380938863	2.42556767	0.015284466	0.063892474
Ulk2	562.4404526	0.4840937	0.091419864	5.295279155	1.19E-07	2.00E-06
Ulk3	83.45169362	-0.385397286	0.194257357	-1.983952074	0.047261183	0.152470984
Unc5b	194.3750867	-1.226302517	0.139842239	-8.769185362	1.80E-18	1.35E-16
Unc93b1	4307.283335	-0.156078831	0.072136576	-2.163657335	0.030490658	0.110551655
Ung	39.79573361	1.292972454	0.277118921	4.665767504	3.07E-06	3.99E-05
Upk1b	24.29876695	-1.391692184	0.327580212	-4.248401258	2.15E-05	0.000231948
Uqcc1	159.0955204	0.357666841	0.131916817	2.711305882	0.006701877	0.033258951
Uqcc2	254.2583306	0.307407404	0.128383239	2.394451219	0.016645257	0.06843709
Uqcr11	393.7412162	0.299373327	0.099150945	3.019369377	0.002533015	0.014739417
Uqcrb	1047.987273	0.269281631	0.086529725	3.112013009	0.001858163	0.011289657
Uqcrc1	644.3176818	-0.204412819	0.086640758	-2.359314765	0.018308719	0.073840081
Uqcrq	1003.573815	0.220004639	0.096232675	2.286174002	0.022244081	0.08645867
Uri1	333.6499635	0.290650082	0.101919591	2.851758717	0.004347809	0.023352744
Usp1	584.9071929	0.27648665	0.112580218	2.455907934	0.014052915	0.059695831
Usp11	29.87732736	-0.508635513	0.256641418	-1.981891768	0.047491356	0.152940509
Usp13	12.00918102	-1.122165421	0.335914948	-3.340623657	0.000835904	0.005717602
Usp27x	24.62688524	-0.701744496	0.265602365	-2.642086775	0.008239694	0.039242492
Usp30	68.20532803	-0.412745482	0.192654419	-2.142413781	0.0321602	0.115158225
Usp33	245.9544797	-0.281974614	0.106922271	-2.637192521	0.008359537	0.039609024
Usp34	1464.568051	-0.200178145	0.099097151	-2.020019183	0.043381398	0.143050898
Usp35	73.62809567	-0.673204294	0.197962409	-3.400667309	0.000672216	0.004730445
Usp4	485.2827913	-0.194017844	0.089682457	-2.163386795	0.030511442	0.110600293
Usp40	256.1162687	-0.272580934	0.134160903	-2.031746408	0.042179335	0.140135481
Usp47	772.3506312	-0.175053959	0.08546386	-2.048280513	0.040532521	0.13643664
Usp48	1003.76929	-0.171977732	0.082163774	-2.093108957	0.036339429	0.126004116
Usp49	25.29526267	0.512143319	0.260796504	1.963766047	0.049557229	0.157264821
Usp6nl	198.6871534	-0.546399867	0.131721428	-4.148147154	3.35E-05	0.000342643
Utp11	573.4575446	0.279107714	0.080161732	3.481807435	0.000498042	0.003667376
Utp14b	97.18887908	0.491154107	0.184583313	2.660880327	0.007793665	0.037552304
Utp4	171.2502159	0.284701537	0.140135907	2.031610193	0.042193134	0.140135481
Utp6	204.2207888	0.324678906	0.130408399	2.489708548	0.012784789	0.05551543
Utrn	476.818838	-0.440804085	0.124062649	-3.553076519	0.000380754	0.0029175
Uvrag	633.140881	0.311286266	0.091118186	3.416291288	0.000634803	0.004508514
Vamp2	106.4891192	-0.39377215	0.162473672	-2.423605908	0.015367272	0.064167125
Vamp3	1258.312846	0.702544387	0.081685566	8.600593986	7.93E-18	5.43E-16
Vamp4	949.8869797	-0.325567305	0.085309569	-3.81630466	0.000135465	0.001183999
Vars	149.2789242	0.319757189	0.144450349	2.213613133	0.026855403	0.100128953
Vasp	880.8386236	0.208357878	0.079352546	2.625723904	0.008646492	0.040619448
Vat1	867.8683183	-0.81944727	0.123853166	-6.616280356	3.68E-11	1.05E-09
Vav2	126.9881735	-0.560957444	0.138314721	-4.055659719	5.00E-05	0.000489397
Vav3	611.6229137	0.434639684	0.117780926	3.690238311	0.000224044	0.001845351
Vbp1	436.9455742	0.278184879	0.108846249	2.555759915	0.01059562	0.047693001

Vcam1	53.33215293	-0.698262362	0.201704208	-3.461813564	0.000536549	0.003918241
Vcan	267.6909419	0.725245183	0.186159367	3.895829647	9.79E-05	0.000887928
Vcl	314.0258168	-0.545091097	0.113113785	-4.818962576	1.44E-06	2.01E-05
Vcp	2220.727903	0.185082188	0.086506313	2.139522334	0.032393389	0.115744664
Vdac2	2606.441187	-0.222977997	0.093321581	-2.389350837	0.016878176	0.069205133
Vdr	13.6115562	-0.892654471	0.329187542	-2.711689711	0.006694122	0.033231457
Vegfa	1065.327011	-0.74580501	0.133349059	-5.592877927	2.23E-08	4.23E-07
Vegfb	240.6887585	0.501781028	0.123569005	4.060735366	4.89E-05	0.000480425
Vegfc	27.37270396	-1.15353698	0.279725957	-4.123811008	3.73E-05	0.000377612
Vgll3	21.77658347	-1.586289894	0.294035986	-5.394883508	6.86E-08	1.20E-06
Vkorc1	297.8814707	0.222854647	0.112727636	1.976930019	0.048049541	0.153966418
Vldlr	15.23933468	-0.832513015	0.319389292	-2.606577724	0.009145207	0.042332546
Vma21	449.655067	0.279305768	0.105691171	2.642659415	0.008225773	0.039199778
Vmac	62.6193673	-0.393497246	0.189882308	-2.072321797	0.038235441	0.130497899
Vmp1	1741.111599	-0.35802314	0.084121318	-4.256033388	2.08E-05	0.000225306
Vpreb3	3.873046612	0.753513028	0.37416389	2.013858226	0.04402442	0.144563094
Vps13b	480.8904615	-0.358447303	0.120744788	-2.968635814	0.002991249	0.016984363
Vps26c	383.2563866	0.227037603	0.104298347	2.176809221	0.029494805	0.107800423
Vps29	1198.488668	0.482457008	0.070847712	6.809775402	9.78E-12	2.99E-10
Vps35	3360.939461	0.356641035	0.074709024	4.773734364	1.81E-06	2.46E-05
Vps36	609.8585024	0.388011048	0.107436033	3.61155411	0.000304368	0.002409095
Vps39	317.6898718	-0.344556531	0.098170072	-3.509791987	0.000448457	0.003371743
Vps4b	1591.623182	0.199031893	0.083950898	2.370813159	0.017749001	0.07200845
Vps53	290.54706	0.220815588	0.109113742	2.02371933	0.042999039	0.142194033
Vrk1	237.6022522	0.722088893	0.132924635	5.432318036	5.56E-08	9.86E-07
Vsig10	37.02233824	-0.958088525	0.238991757	-4.008876856	6.10E-05	0.000585016
Vsig10l	16.80974552	-0.601446723	0.306725126	-1.96086552	0.049894712	0.158101763
Vsig4	3991.574119	1.138650624	0.144760479	7.86575611	3.67E-15	1.78E-13
Vsig8	46.09381868	0.682871356	0.228134751	2.99328074	0.002759957	0.015826779
Vsir	4340.21922	-0.447950646	0.091381583	-4.901979474	9.49E-07	1.37E-05
Vta1	327.0482957	0.284539119	0.10694086	2.660714691	0.0077975	0.03755362
Vti1b	582.2505653	0.336782983	0.097703782	3.446979992	0.000566891	0.004107835
Vwf	22.45886903	-1.128173417	0.306845212	-3.676685744	0.000236284	0.001932377
Wapl	983.0267056	0.218767445	0.110620877	1.977632537	0.047970176	0.153963543
Wasf2	2450.673981	0.361448714	0.085490161	4.227956872	2.36E-05	0.000251167
Washc3	265.8956389	0.287772244	0.110633866	2.60112255	0.009291925	0.042879436
Washc4	1156.477431	0.243129584	0.083778325	2.902058294	0.003707195	0.020363793
Washc5	523.9438613	0.608929649	0.092631172	6.573701251	4.91E-11	1.38E-09
Wbp11	735.0294388	0.232068032	0.100678892	2.305031634	0.021164802	0.082929551
Wbp1l	656.3950062	-0.183689922	0.088972322	-2.064573771	0.038963347	0.132500102
Wdfy3	1075.788611	0.259221578	0.082326056	3.148718541	0.001639881	0.010211489
Wdfy4	650.9284465	0.374917945	0.098733753	3.797262191	0.000146303	0.001264729
Wdhd1	101.8594137	1.064607374	0.180925725	5.884223351	4.00E-09	8.56E-08
Wdpcp	32.91465825	-0.518700044	0.259423857	-1.999430778	0.045561765	0.148511164

Wdr26	5253.429553	0.257070172	0.069108676	3.719795938	0.000199384	0.001662307
Wdr36	314.3278433	0.244284003	0.103117366	2.368989946	0.017836738	0.072305762
Wdr45b	704.1407597	0.183888032	0.075691731	2.429433558	0.015122436	0.063409445
Wdr5	297.3574184	0.404061843	0.119763046	3.373844064	0.000741263	0.005157228
Wdr59	44.65209768	0.610240158	0.219630375	2.778487078	0.005461269	0.028173688
Wdr6	146.5497881	0.295026335	0.140293184	2.102927078	0.035472144	0.123913052
Wdr62	43.50896502	0.518855747	0.240069942	2.161269099	0.030674558	0.110950373
Wdr7	351.0212443	0.253518855	0.102648786	2.469769651	0.013520008	0.057986499
Wdr76	124.898344	0.903129383	0.162454478	5.559276636	2.71E-08	5.08E-07
Wdr89	284.966377	-0.256670737	0.112067731	-2.290317956	0.022002892	0.085698779
Wdr90	41.64596905	0.538876637	0.241327074	2.232972158	0.02555078	0.096395319
Wdr91	187.1613304	-0.415318967	0.126017465	-3.295725454	0.000981679	0.006565088
Wdsub1	127.6887211	-0.697220067	0.140269017	-4.970592084	6.67E-07	9.89E-06
Wfdc17	18637.62144	0.77790567	0.130499439	5.96098862	2.51E-09	5.52E-08
Wfdc18	55.31719761	0.809424924	0.227899191	3.55167967	0.000382781	0.002927823
Wfs1	153.8015088	-1.031538943	0.16743343	-6.16088999	7.23E-10	1.74E-08
Wipi1	460.9409049	0.487159294	0.113493887	4.292383569	1.77E-05	0.000195628
Wnk1	4459.433855	-0.152265241	0.072234139	-2.107940141	0.035036168	0.122732905
Wnt10a	2.805038896	-0.825211925	0.368819738	-2.237439703	0.025257616	0.095597287
Wnt2	22.35404874	-0.880617499	0.286192047	-3.077015972	0.002090841	0.012515856
Wrn	258.5008689	-0.304369363	0.153187591	-1.986906131	0.0469328	0.151760511
Wsb2	769.9022564	-0.551350688	0.106269837	-5.18821432	2.12E-07	3.41E-06
Wt1	82.62231724	-0.88348484	0.213353398	-4.140945715	3.46E-05	0.000352381
Wwp1	2989.237199	0.62134091	0.086174504	7.210263818	5.58E-13	1.99E-11
Wwtr1	108.1559038	-1.008405912	0.185883571	-5.424932951	5.80E-08	1.02E-06
Xab2	159.1302213	-0.311210621	0.128154677	-2.428398462	0.015165671	0.063544719
Xbp1	773.4616067	0.220015866	0.108723162	2.023633804	0.043007844	0.142194033
Xdh	1341.632614	-0.393765379	0.098503049	-3.997494304	6.40E-05	0.000608806
Xk	11.64487583	0.98922428	0.335649772	2.947191868	0.003206742	0.018021044
Xlr4b	75.22416027	0.385409797	0.180742219	2.132372831	0.032976209	0.117324317
Xpo1	741.3105326	0.313148879	0.103520283	3.02500022	0.002486329	0.014495861
Xpot	359.1117504	0.238408316	0.115724523	2.060136519	0.039385492	0.133572446
Xpr1	1321.24116	-0.289413573	0.082381164	-3.513103693	0.000442905	0.003338357
Xrcc2	25.01793803	0.833808969	0.277345285	3.006393161	0.002643669	0.015306152
Xrn2	1550.815001	0.232403938	0.093173388	2.494316706	0.012619989	0.054958842
Xxylt1	37.8273641	0.843436872	0.260620273	3.236267317	0.001211039	0.007874378
Xylt1	574.3981354	-0.907071099	0.14286907	-6.348967625	2.17E-10	5.65E-09
Xylt2	296.7004219	0.423733824	0.106796644	3.967669838	7.26E-05	0.000680318
Yae1d1	23.93764048	-0.574718067	0.289687496	-1.983924316	0.047264278	0.152470984
Yaf2	405.1726131	0.611096301	0.096778652	6.314370865	2.71E-10	6.98E-09
Yap1	53.63932744	-0.55402195	0.219142446	-2.528136201	0.011466986	0.050777532
Ybx1	5962.09716	0.231645689	0.08929748	2.594089865	0.009484169	0.043552304
Ykt6	1037.211628	0.350549302	0.120219473	2.915911162	0.003546515	0.019581511
Ypel2	236.6583867	-0.402473458	0.160343717	-2.510066905	0.012070829	0.053075573

Ypel3	1163.575137	-0.60207018	0.105767943	-5.692369212	1.25E-08	2.49E-07
Ypel5	725.3379413	0.283622493	0.090439171	3.136058086	0.001712353	0.010570662
Ywhab	3244.613293	0.283369154	0.073868455	3.836132152	0.000124987	0.001103343
Ywhae	3310.300881	0.192246721	0.090567524	2.122689369	0.03377989	0.119588303
Ywhah	1057.890268	-0.284813767	0.09467522	-3.008324307	0.002626926	0.015226839
Zbed4	111.4597961	0.698908387	0.14613581	4.782594964	1.73E-06	2.37E-05
Zbtb10	39.02177642	-0.715454373	0.238136557	-3.004386987	0.002661166	0.015383713
Zbtb16	31.93276718	-0.763532033	0.283948677	-2.688979014	0.007167092	0.035103315
Zbtb18	185.0963476	-0.430079925	0.123553855	-3.48091061	0.000499712	0.003676069
Zbtb21	113.7045727	-0.366024918	0.1762601	-2.076618117	0.037836819	0.129543623
Zbtb34	206.0898311	0.470027291	0.119559424	3.931327817	8.45E-05	0.000779681
Zbtb37	140.7940278	-0.404529196	0.16691932	-2.423501334	0.015371697	0.064167748
Zbtb42	43.82735747	-0.517856904	0.23363098	-2.216559227	0.026653226	0.099597851
Zbtb46	27.08921624	-0.895102924	0.271684694	-3.294638756	0.000985483	0.00658759
Zbtb48	24.13934167	-0.756748285	0.276370965	-2.738161318	0.006178376	0.031228997
Zbtb7c	40.95471767	-0.964495785	0.243824998	-3.955688683	7.63E-05	0.000712221
Zbtb8os	250.5004811	0.326594108	0.108019034	3.023486673	0.0024988	0.014562911
Zc3h12a	106.8950675	-0.582579822	0.178339109	-3.266696943	0.001088101	0.007165042
Zc3h12d	104.2455008	-0.40370199	0.182967973	-2.206407948	0.027355449	0.101489054
Zc3h3	16.68889824	-0.638682882	0.312965776	-2.040743528	0.041276326	0.138020016
Zc3h7b	178.6742038	0.283502906	0.1312889	2.159382139	0.030820532	0.111317384
Zc3hav1l	6.958096324	1.020178359	0.371117433	2.748936776	0.005978892	0.030363868
Zcchc10	268.3760641	0.215746984	0.109763484	1.965562463	0.049349174	0.156820155
Zcchc24	792.419202	0.616540126	0.089225405	6.909916787	4.85E-12	1.58E-10
Zcwpw1	42.02640138	0.819976693	0.231571608	3.540920673	0.000398733	0.003040545
Zdbf2	7.659064573	-0.949848385	0.368742454	-2.575912744	0.009997587	0.045396003
Zdhhc12	133.5750619	0.323080257	0.148632333	2.17368759	0.029728605	0.108443651
Zdhhc13	86.04442334	0.402401281	0.169319748	2.376576186	0.017474152	0.071143409
Zdhhc18	182.3192092	-0.350928697	0.138508571	-2.533624408	0.011288966	0.050241255
Zdhhc2	32.20284034	1.903883516	0.282453074	6.740530355	1.58E-11	4.67E-10
Zdhhc21	492.6654451	0.211608703	0.094210583	2.246124539	0.024696032	0.093945083
Zdhhc5	331.8613534	0.238475736	0.103556502	2.302856239	0.021286933	0.083342813
Zdhhc9	578.4668645	-0.20146973	0.086221346	-2.336657214	0.019457015	0.07753357
Zeb2	4217.018221	-0.267148138	0.103184266	-2.589039476	0.009624406	0.04410182
Zeb2os	280.2397214	0.489950082	0.104898888	4.670688989	3.00E-06	3.91E-05
Zer1	77.42353887	-0.499394593	0.206178754	-2.422143808	0.015429243	0.064318515
Zfand5	829.7813236	-0.282532781	0.092007417	-3.070760944	0.00213514	0.012745445
Zfand6	1098.307698	0.187807913	0.074995539	2.504254454	0.012270975	0.053740399
Zfc3h1	418.0727639	-0.28693557	0.123204738	-2.328932928	0.019862619	0.078961146
Zfp113	110.0944855	-0.302319696	0.147072989	-2.055575939	0.039823409	0.134510443
Zfp131	405.3626797	0.259203218	0.112996159	2.293911761	0.021795568	0.085023677
Zfp142	90.58337688	-0.734620257	0.173765995	-4.227641065	2.36E-05	0.000251167
Zfp217	787.7167037	0.487949387	0.088131817	5.536586022	3.08E-08	5.72E-07
Zfp219	202.1951491	-0.528101714	0.126017336	-4.190706858	2.78E-05	0.000291021

Zfp24	455.433706	-0.201762415	0.097704937	-2.065017598	0.038921335	0.132423726
Zfp276	111.6885464	-0.303870949	0.153653053	-1.97764342	0.047968947	0.153963543
Zfp281	419.5806558	-0.250706039	0.109865875	-2.281928199	0.02249358	0.087180049
Zfp286	3.945006026	0.781091304	0.381389628	2.048014018	0.040558626	0.136471591
Zfp316	56.88617592	-0.436595414	0.195523875	-2.232951934	0.025552114	0.096395319
Zfp318	680.762468	-0.41145514	0.100812571	-4.081387224	4.48E-05	0.000443744
Zfp36	733.4008283	-0.40336565	0.122109887	-3.303300496	0.000955539	0.006410272
Zfp367	163.3642138	0.891742692	0.14073969	6.336113784	2.36E-10	6.12E-09
Zfp36l1	2306.885793	0.416914313	0.131859162	3.161815276	0.00156789	0.009836674
Zfp40	72.97220045	0.633375834	0.206140975	3.072537296	0.002122473	0.012674872
Zfp438	25.89275449	-0.593704195	0.266512165	-2.227681409	0.025901768	0.097484642
Zfp469	4.790999692	-1.295413558	0.381378863	-3.396657981	0.000682142	0.004792556
Zfp518b	78.5357653	0.367086013	0.179137937	2.049180755	0.040444443	0.136240126
Zfp52	27.62907171	0.527237604	0.266362226	1.979400802	0.047770898	0.1535442
Zfp524	75.56364978	-0.430613203	0.171633877	-2.508905644	0.012110583	0.053225219
Zfp532	77.31236984	-0.986490421	0.191511632	-5.151073136	2.59E-07	4.11E-06
Zfp541	4.219126733	-0.870430888	0.381388539	-2.28226808	0.022473518	0.087124795
Zfp553	86.05892975	-0.345159702	0.17458028	-1.977082989	0.04803225	0.153966418
Zfp592	222.1380327	-0.400180913	0.12473394	-3.208276047	0.001335333	0.008531008
Zfp597	118.0066649	0.422901334	0.154938557	2.729477677	0.006343474	0.031817019
Zfp607b	22.00807358	-0.627886656	0.279540274	-2.246140235	0.024695027	0.093945083
Zfp646	93.59347067	-0.640454311	0.183542883	-3.489398775	0.000484108	0.003582354
Zfp691	23.91930527	-1.005197911	0.292570687	-3.435743755	0.00059093	0.004251086
Zfp697	19.57196951	-0.576055153	0.289039709	-1.992996586	0.046261821	0.150238292
Zfp704	364.3589796	-1.220840857	0.126358981	-9.661686472	4.39E-22	4.70E-20
Zfp710	803.7508535	0.328822169	0.079761511	4.122566972	3.75E-05	0.000379145
Zfp772	30.14948521	0.573029448	0.266275524	2.152016975	0.031396017	0.113096504
Zfp820	27.03922722	1.005524627	0.282826285	3.555272908	0.000377587	0.002895476
Zfp867	36.36582819	-0.536458042	0.232218036	-2.310148046	0.020879959	0.082135137
Zfp874a	50.57586603	-0.682821608	0.210798082	-3.239221164	0.001198566	0.007803418
Zfp874b	79.88198895	-0.635216171	0.172998114	-3.671809809	0.000240839	0.001964277
Zfp91	1676.715548	0.242555437	0.097182905	2.495865265	0.012565032	0.054775271
Zfp945	134.8131779	-0.339584935	0.166943387	-2.034132298	0.041938258	0.139611234
Zfp952	99.59521396	-0.37812317	0.187481438	-2.016856566	0.043710484	0.143725511
Zfp958	113.5960716	0.497222795	0.160451571	3.098896397	0.001942429	0.011716253
Zfp979	6.946105565	0.829175365	0.375118689	2.210434697	0.027075008	0.100706321
Zfpm1	65.1368394	-0.603115715	0.193763201	-3.112643232	0.0018542	0.011283853
Zfpm2	15.87034806	-0.785740501	0.30845121	-2.547373704	0.010853713	0.048664976
Zfx	549.9637203	0.359613166	0.100311127	3.584977817	0.000337107	0.002622583
Zfyve28	87.78811391	-1.900135234	0.185257528	-10.25672345	1.10E-24	1.64E-22
Zfyve9	280.8877334	-0.306019539	0.14208551	-2.15377022	0.031258196	0.112708253
Zgrf1	38.66072515	0.736078361	0.259128387	2.840593298	0.00450297	0.024039868
Zkscan3	383.2890795	-0.470196505	0.107085848	-4.390837029	1.13E-05	0.000130952
Zkscan5	34.77155373	-0.796716855	0.242074928	-3.291199386	0.000997612	0.006662732

Zmiz1	1561.306364	-0.42473826	0.08587772	-4.945849282	7.58E-07	1.12E-05
Zmiz2	212.9418196	-0.336432948	0.130971979	-2.568739908	0.010206903	0.046150949
Zmym4	251.5656955	-0.28985768	0.141678656	-2.045881073	0.040768073	0.136992045
Zmynd15	29.89354064	-1.070426852	0.267071872	-4.008010449	6.12E-05	0.000586417
Zmynd19	66.71770281	0.70661809	0.194149044	3.639565121	0.000273099	0.00219989
Znhit3	60.65674017	0.398629656	0.188563589	2.114033037	0.034512446	0.121451171
Znrf2	715.1666702	-0.180148782	0.090677075	-1.986707019	0.046954874	0.151766484
Zranb1	445.237641	-0.340946185	0.096900985	-3.518500725	0.000433993	0.003282705
Zranb2	845.4041268	0.257849585	0.097709216	2.638948466	0.008316362	0.039507327
Zranb3	438.6082518	0.52931558	0.182834506	2.89505297	0.003790946	0.020755462
Zrsr1	325.8649214	-0.408511168	0.118599079	-3.44447167	0.000572177	0.004138147
Zscan26	215.0807673	-0.397862484	0.12174296	-3.268053299	0.0010829	0.00714333
Zscan29	249.830188	-0.432783957	0.114459643	-3.781105236	0.000156134	0.001336622
Zswim7	37.5013225	-0.735397865	0.238290174	-3.086144315	0.002027704	0.012167034
Zup1	76.87425413	-0.420393212	0.196340631	-2.141142203	0.032262572	0.115405492
Zw10	132.8211391	0.516790718	0.148583048	3.478127044	0.000504931	0.003705375
Zwilch	71.16819939	1.479241613	0.209345579	7.066027484	1.59E-12	5.44E-11
Zxdc	145.39008	-0.393890081	0.133700201	-2.946069469	0.003218401	0.018062283
Zyx	617.033451	-0.726491301	0.092577355	-7.847397475	4.25E-15	2.04E-13
Zzz3	682.391147	-0.242577878	0.093499883	-2.594419063	0.009475092	0.043523938

Table S2. Enriched gene sets after GSEA analysis are listed with corresponding enrichment scores (ES) and normalized ES (NES).

NAME	ES	NES	NOM p-val	FDR q-val	FWER p-val
CHEMNITZ_RESPONSE_TO_PROSTAGLANDIN_E2_UP	-0.7	-2.5	0	0	0
FISCHER_G1_S_CELL_CYCLE	-0.6	-2.4	0	0	0
PID_ATR_PATHWAY	-0.8	-2.4	0	0	0
LASTOWSKA_NEUROBLASTOMA_COPY_NUMBER_UP	-0.4	-2.4	0	0	0
MITSIADES_RESPONSE_TO_APLIDIN_DN	-0.6	-2.4	0	0	0
YU_BAP1_TARGETS	-0.7	-2.3	0	0	0
REACTOME_RNA_POLYMERASE_II_TRANSCRIPTION_TERMINATION	-0.5	-2.3	0	0	0
BIOCARTA_RACCYCD_PATHWAY	-0.5	-2.3	0	0	0
RIZ_ERYTHROID_DIFFERENTIATION	-0.7	-2.3	0	0	0
SEIDEN_MET_SIGNALING	-0.7	-2.3	0	0	0
FUJII_YBX1_TARGETS_DN	-0.7	-2.3	0	0	0
VERNELL_RETINOBLASTOMA_PATHWAY_UP	-0.7	-2.3	0	0	0
WANG_RESPONSE_TO_GSK3_INHIBITOR_SB216763_DN	-0.6	-2.2	0	0	0
VILLANUEVA_LIVER_CANCER_KRT19_UP	-0.7	-2.2	0	0	0
KAUFFMANN_DNA_REPLICATION_GENES	-0.6	-2.2	0	0	0
KOKKINAKIS_METHIONINE_DEPRIVATION_96HR_DN	-0.6	-2.2	0	0	0
PYEON_CANCER_HEAD_AND_NECK_VS_CERVICAL_UP	-0.6	-2.2	0	0	0
SU_TESTIS	-0.7	-2.2	0	0	0
REACTOME_RNA_POLYMERASE_II_TRANSCRIPTION_PRE_INITIATION_AND_PROMOTER_OPENING	-0.5	-2.2	0	0	0
THILLAINADESAN_ZNF217_TARGETS_UP	-0.6	-2.1	0	0	0
KAUFFMANN_MELANOMA_RELAPSE_UP	-0.8	-2.1	0	0	0
NUNODA_RESPONSE_TO_DASATINIB_IMATINIB_UP	-0.6	-2.1	0	0	0
PID_E2F_PATHWAY	-0.6	-2.1	0	0	0
KEGG_OOCYTE_MEIOSIS	-0.5	-2.1	0	0	0
SHEPARD_BMYB_MORPHOLINO_DN	-0.5	-2.1	0	0	0
REACTOME_TRANSCRIPTION_OF_THE_HIV_GENOME	-0.4	-2.1	0	0	0
KEGG_CELL_CYCLE	-0.6	-2.1	0	0	0
KEGG_PROGESTERONE_MEDIATED_OOCYTE_MATURATION	-0.5	-2.1	0	0	0
KONG_E2F3_TARGETS	-0.9	-2.1	0	0	0
BROWNE_HCMV_INFECTION_10HR_DN	-0.5	-2.1	0	0	0
SARRIO_EPITHELIAL_MESENCHYMAL_TRANSITION_UP	-0.7	-2.1	0	0	0

RODRIGUES_THYROID_CARCINOMA_POORLY_DIFFERENTIATED_UP	-0.5	-2.1	0	0	0
BIDUS_METASTASIS_UP	-0.5	-2.1	0	0	0
SHEPARD_CRUSH_AND_BURN_MUTANT_DN	-0.6	-2.1	0	0	0
FISCHER_DREAM_TARGETS	-0.7	-2.1	0	0	0
SMIRNOV_RESPONSE_TO_IR_6HR_DN	-0.5	-2.1	0	0	0
KEGG_HOMOLOGOUS_RECOMBINATION	-0.7	-2.1	0	0	0
LANG_MYB_FAMILY_TARGETS	-0.5	-2.1	0	0	0
FISCHER_G2_M_CELL_CYCLE	-0.7	-2.1	0	0	0
IWANAGA_E2F1_TARGETS_INDUCED_BY_SERUM	-0.7	-2.1	0	0	0
WILCOX_RESPONSE_TO_PROGESTERONE_UP	-0.5	-2.1	0	0	0
REACTOME_RESOLUTION_OF_D_LOOP_STRUCTURES	-0.7	-2.1	0	0	0
FERREIRA_EWINGS_SARCOMA_UNSTABLE_VS_STABLE_UP	-0.7	-2.1	0	0	0
BRACHAT_RESPONSE_TO_METHOTREXATE_DN	-0.6	-2.1	0	0	0
PEART_HDAC_PROLIFERATION_CLUSTER_DN	-0.6	-2.1	0	0	0
CHEN_ETV5_TARGETS_TESTIS	-0.8	-2.1	0	0	0
BIOCARTA_FMLP_PATHWAY	-0.5	-2.1	0	0	0
JOHNSTONE_PARVB_TARGETS_3_DN	-0.5	-2.1	0	0	0
PUJANA_BRCA_CENTERED_NETWORK	-0.7	-2.1	0	0	0
SHEPARD_BMYB_TARGETS	-0.7	-2.1	0	0	0
WHITFIELD_CELL_CYCLE_G2	-0.6	-2.1	0	0	0
PUJANA_BREAST_CANCER_LIT_INT_NETWORK	-0.6	-2	0	0	0
JIANG_HYPOXIA_CANCER	-0.5	-2	0	0	0
ODONNELL_TFRC_TARGETS_DN	-0.8	-2	0	0.001	0.034
BENPORATH_CYCLING_GENES	-0.5	-2	0	0.001	0.034
MORI_EMU_MYC_LYMPHOMA_BY_ONSET_TIME_UP	-0.6	-2	0	0.001	0.034
MARSON_BOUND_BY_E2F4_UNSTIMULATED	-0.6	-2	0	0.001	0.034
KOBAYASHI_EGFR_SIGNALING_24HR_DN	-0.8	-2	0	0.001	0.034
PID_FOXM1_PATHWAY	-0.7	-2	0	0.001	0.034
BIOCARTA_CHREBP_PATHWAY	-0.6	-2	0	0.001	0.034
KIM_WT1_TARGETS_DN	-0.4	-2	0	0.001	0.034
REACTOME_RESOLUTION_OF_D_LOOP_STRUCTURES_THROUGH_SYNTHESIS_DEPENDENT_STRAND_ANNEALING_SDSA	-0.7	-2	0	0.001	0.034
MISSIAGLIA_REGULATED_BY_METHYLATION_DN	-0.7	-2	0	0.001	0.034
TOYOTA_TARGETS_OF_MIR34B_AND_MIR34C	-0.5	-2	0	0.001	0.068
PID_PLK1_PATHWAY	-0.8	-2	0	0.001	0.068
DUTERTRE_ESTRADIOL_RESPONSE_24HR_UP	-0.8	-2	0	0.001	0.068
SONG_TARGETS_OF_IE86_CMV_PROTEIN	-0.8	-2	0	0.001	0.068
REACTOME_TRANSCRIPTIONAL_REGULATION_BY_E2F6	-0.6	-2	0	0.001	0.068
JOHANSSON_GLIOMAGENESIS_BY_PDGFB_UP	-0.6	-2	0	0.001	0.068

SHEDDEN_LUNG_CANCER_POOR_SURVIVAL_A6	-0.7	-2	0	0.001	0.068
TANG_SENESCENCE_TP53_TARGETS_DN	-0.8	-2	0	0.001	0.068
BIOCARTA_CELLCYCLE_PATHWAY	-0.8	-2	0	0.001	0.068
REACTOME_TRANSPORT_OF_MATURE_TRANSCRIPT_TO_CYTOPLASM	-0.6	-2	0	0.001	0.068
REACTOME_CELL_CYCLE	-0.6	-2	0	0.001	0.068
CHIANG_LIVER_CANCER_SUBCLASS_PROLIFERATION_UP	-0.7	-2	0	0.001	0.068
PYEON_HPV_POSITIVE_TUMORS_UP	-0.6	-2	0	0.001	0.068
KEGG_BASAL_TRANSCRIPTION_FACTORS	-0.5	-2	0	0.001	0.068
REACTOME_PHOSPHORYLATION_OF_THE_APC_C	-0.7	-2	0	0.001	0.068
REACTOME_G1_PHASE	-0.5	-2	0	0.001	0.068
WHITFIELD_CELL_CYCLE_G1_S	-0.6	-2	0	0.001	0.068
YAO_TEMPORAL_RESPONSE_TO_PROGESTERONE_CLUSTER_15	-0.6	-2	0	0.001	0.068
REACTOME_ONCOGENE_INDUCED_SENESCENCE	-0.5	-2	0	0.001	0.068
BURTON_ADIPOGENESIS_3	-0.8	-2	0	0.001	0.068
PUJANA_XPRSS_INT_NETWORK	-0.7	-2	0	0.001	0.068
CAFFAREL_RESPONSE_TO_THC_DN	-0.8	-2	0	0.001	0.098
WEST_ADRENOCORTICAL_TUMOR_MARKERS_UP	-0.8	-2	0	0.001	0.098
BIOCARTA_G1_PATHWAY	-0.6	-2	0	0.001	0.098
WHITFIELD_CELL_CYCLE_S	-0.6	-2	0	0.001	0.098
STANELLE_E2F1_TARGETS	-0.5	-1.9	0	0.001	0.098
PUJANA_BRCA2_PCC_NETWORK	-0.6	-1.9	0	0.001	0.098
FERRANDO_T_ALL_WITH_MLL_ENL_FUSION_DN	-0.6	-1.9	0	0.001	0.098
REACTOME_PROCESSING_OF_CAPPED_INTRONLESS_PRE_MRNA	-0.5	-1.9	0	0.001	0.098
DUTERTRE_ESTRADIOL_RESPONSE_6HR_UP	-0.5	-1.9	0	0.001	0.121
REACTOME_HOMOLOGOUS_DNA_PAIRING_AND_STRAND_EXCHANGE	-0.7	-1.9	0	0.001	0.121
PUJANA_CHEK2_PCC_NETWORK	-0.5	-1.9	0	0.001	0.121
GOBERT_OLIGODENDROCYTE_DIFFERENTIATION_UP	-0.7	-1.9	0	0.002	0.143
YU_MYC_TARGETS_UP	-0.8	-1.9	0	0.002	0.143
KINSEY_TARGETS_OF_EWSR1_FLII_FUSION_UP	-0.5	-1.9	0	0.002	0.143
NADERI_BREAST_CANCER_PROGNOSIS_UP	-0.7	-1.9	0	0.002	0.143
ZHENG_GLIOBLASTOMA_PLASTICITY_UP	-0.6	-1.9	0	0.002	0.143
REACTOME_PROCESSING_OF_CAPPED_INTRON_CONTAINING_PRE_MRNA	-0.4	-1.9	0	0.002	0.143
REACTOME_REGULATION_OF_TP53_ACTIVITY_THROUGH_PHOSPHORYLATION	-0.6	-1.9	0	0.002	0.143
REACTOME_CELL_CYCLE_MITOTIC	-0.6	-1.9	0	0.002	0.17
ROSTY_CERVICAL_CANCER_PROLIFERATION_CLUSTER	-0.8	-1.9	0	0.002	0.17

LINDGREN_BLADDER_CANCER_CLUSTER_1_DN	-0.5	-1.9	0	0.002	0.17
FLORIO_NEOCORTEX_BASAL_RADIAL_GLIA_DN	-0.8	-1.9	0	0.002	0.17
FURUKAWA_DUSP6_TARGETS_PCI35_DN	-0.8	-1.9	0	0.002	0.17
KEGG_PYRIMIDINE_METABOLISM	-0.6	-1.9	0	0.002	0.17
CHIARETTI_T_ALL_RELAPSE_PROGNOSIS	-0.7	-1.9	0	0.002	0.17
ZHOU_CELL_CYCLE_GENES_IN_IR_RESPONSE_6HR	-0.8	-1.9	0	0.002	0.17
PUJANA_BREAST_CANCER_WITH_BRCA1_MUTATED_UP	-0.7	-1.9	0	0.002	0.17
WU_APOPTOSIS_BY_CDKN1A_VIA_TP53	-0.9	-1.9	0	0.002	0.17
MARKEY_RB1_ACUTE_LOF_UP	-0.6	-1.9	0	0.002	0.17
GAL_LEUKEMIC_STEM_CELL_DN	-0.5	-1.9	0	0.002	0.17
NUYTTEN_EZH2_TARGETS_DN	-0.5	-1.9	0	0.002	0.17
REACTOME_CELL_CYCLE_CHECKPOINTS	-0.6	-1.9	0	0.002	0.17
STEIN_ESR1_TARGETS	-0.5	-1.9	0	0.002	0.17
REACTOME_MAPK_TARGETS_NUCLEAR_EVENTS_MEDIATED_BY_MAP_KINASES	-0.5	-1.9	0	0.002	0.17
LEE_EARLY_T_LYMPHOCYTE_UP	-0.8	-1.9	0	0.002	0.17
WHITFIELD_CELL_CYCLE_G2_M	-0.6	-1.9	0	0.002	0.17
CHNG_MULTIPLE_MYELOMA_HYPERPLOID_DN	-0.5	-1.9	0	0.002	0.17
BIOCARTA_G2_PATHWAY	-0.7	-1.9	0	0.002	0.199
REACTOME_HOMOLOGY_DIRECTED_REPAIR	-0.6	-1.9	0	0.002	0.199
PETROVA_PROX1_TARGETS_UP	-0.7	-1.9	0	0.002	0.199
REACTOME_ACTIVATION_OF_ATR_IN_RESPONSE_TO_REPLICATION_STRESS	-0.9	-1.9	0	0.002	0.199
REACTOME_MRNA_SPLICING	-0.4	-1.9	0	0.002	0.199
SCIBETTA_KDM5B_TARGETS_DN	-0.6	-1.9	0	0.002	0.199
HONRADO_BREAST_CANCER_BRCA1_VS_BRCA2	-0.8	-1.9	0	0.002	0.199
REACTOME_REGULATION_OF_TP53_ACTIVITY	-0.4	-1.9	0	0.002	0.229
MATZUK_SPERMATOCYTE	-0.5	-1.9	0	0.002	0.229
STEIN_ESRRA_TARGETS_RESPONSIVE_TO_ESTROGEN_DN	-0.7	-1.9	0	0.002	0.229
REACTOME_RESOLUTION_OF_SISTER_CHROMATID_COHESION	-0.7	-1.9	0	0.002	0.229
FLOTHO_PEDIATRIC_ALL_THERAPY_RESPONSE_UP	-0.5	-1.9	0	0.002	0.229
RUIZ_TNC_TARGETS_DN	-0.7	-1.9	0	0.002	0.229
GEORGES_CELL_CYCLE_MIR192_TARGETS	-0.6	-1.9	0	0.002	0.229
GOLDRATH_ANTIGEN_RESPONSE	-0.6	-1.9	0	0.002	0.229
HOFFMANN_LARGE_TO_SMALL_PRE_BII_LYMPHOCYTE_UP	-0.7	-1.9	0	0.002	0.229
KEGG_P53_SIGNALING_PATHWAY	-0.5	-1.9	0	0.002	0.229
LIN_MELANOMA_COPY_NUMBER_UP	-0.4	-1.9	0	0.002	0.229
AFFAR_YY1_TARGETS_DN	-0.5	-1.9	0	0.002	0.229
BOHN_PRIMARY_IMMUNODEFICIENCY_SYNDROM_UP	-0.6	-1.9	0	0.002	0.277

REACTOME_HDR_THROUGH_HOMOLOGOUS_RECOMBINATIO N_HRR	-0.7	-1.9	0	0.002	0.277
LEE_LIVER_CANCER_SURVIVAL_DN	-0.5	-1.9	0	0.002	0.3
BRACHAT_RESPONSE_TO_CAMPTOTHECIN_DN	-0.5	-1.9	0	0.003	0.327
KANG_DOXORUBICIN_RESISTANCE_UP	-0.9	-1.9	0	0.003	0.327
REACTOME_E2F_MEDIATED_REGULATION_OF_DNA_REPLICA TION	-0.7	-1.9	0	0.003	0.327
HORIUCHI_WTAP_TARGETS_DN	-0.6	-1.9	0	0.003	0.327
CROONQUIST_IL6_DEPRIVATION_DN	-0.8	-1.9	0	0.003	0.377
BENPORATH_PROLIFERATION	-0.7	-1.9	0	0.003	0.377
BURTON_ADIPOGENESIS_PEAK_AT_16HR	-0.7	-1.9	0	0.003	0.377
REACTOME_MITOTIC_SPINDLE_CHECKPOINT	-0.7	-1.9	0	0.003	0.377
MOLENAAR_TARGETS_OF_CCND1_AND_CDK4_DN	-0.8	-1.9	0	0.003	0.377
PID_FANCONI_PATHWAY	-0.7	-1.8	0	0.003	0.409
REN_BOUND_BY_E2F	-0.8	-1.8	0	0.003	0.409
BOYAULT_LIVER_CANCER_SUBCLASS_G23_UP	-0.7	-1.8	0	0.003	0.409
PETROVA_ENDOTHELIUM_LYMPHATIC_VS_BLOOD_UP	-0.5	-1.8	0	0.003	0.409
REICHERT_MITOSIS_LIN9_TARGETS	-0.9	-1.8	0	0.003	0.409
PUJANA_BRCA1_PCC_NETWORK	-0.4	-1.8	0	0.003	0.409
DORMOY_ELAVL1_TARGETS	-0.7	-1.8	0	0.004	0.409
REACTOME_MISCELLANEOUS_TRANSPORT_AND_BINDING_E VENTS	-0.6	-1.8	0	0.004	0.409
SOTIRIOU_BREAST_CANCER_GRADE_1_VS_3_UP	-0.8	-1.8	0	0.004	0.409
FARMER_BREAST_CANCER_CLUSTER_2	-0.8	-1.8	0	0.004	0.409
MOREAUX_B_LYMPHOCYTE_MATURATION_BY_TACI_DN	-0.6	-1.8	0	0.004	0.409
FEVR_CTNNB1_TARGETS_DN	-0.5	-1.8	0	0.004	0.409
KAUFFMANN_DNA_REPAIR_GENES	-0.5	-1.8	0	0.004	0.433
CHICAS_RB1_TARGETS_LOW_SERUM	-0.6	-1.8	0	0.004	0.467
REACTOME_G0_AND_EARLY_G1	-0.8	-1.8	0	0.004	0.467
REACTOME_G2_M_CHECKPOINTS	-0.6	-1.8	0	0.004	0.467
REACTOME_KINESINS	-0.6	-1.8	0	0.004	0.467
ZHANG_BREAST_CANCER_PROGENITORS_UP	-0.5	-1.8	0	0.004	0.467
LINDGREN_BLADDER_CANCER_CLUSTER_3_UP	-0.6	-1.8	0	0.004	0.467
CAIRO_HEPATOBLASTOMA_CLASSES_UP	-0.5	-1.8	0	0.005	0.497
ODONNELL_TARGETS_OF_MYC_AND_TFRC_DN	-0.8	-1.8	0	0.005	0.497
REACTOME_PROCESSING_OF_DNA_DOUBLE_STRAND_BREA K_ENDS	-0.6	-1.8	0	0.005	0.52
BLUM_RESPONSE_TO_SALIRASIB_DN	-0.6	-1.8	0	0.005	0.551
REACTOME_ACTIVATION_OF_THE_PRE_REPLICATIVE_COMPL EX	-0.9	-1.8	0	0.005	0.551
REACTOME_HIV_LIFE_CYCLE	-0.4	-1.8	0	0.005	0.551

FRASOR_RESPONSE_TO_SERM_OR_FULVESTRANT_DN	-0.8	-1.8	0	0.005	0.551
PID_RB_1PATHWAY	-0.4	-1.8	0	0.005	0.551
MOREAUX_MULTIPLE_MYELOMA_BY_TACI_DN	-0.5	-1.8	0	0.005	0.551
BOYLAN_MULTIPLE_MYELOMA_C_UP	-0.5	-1.8	0	0.005	0.551
KEGG_ONE_CARBON_POOL_BY_FOLATE	-0.7	-1.8	0	0.006	0.551
GRAHAM_CML_QUIESCENT_VS_NORMAL_QUIESCENT_UP	-0.5	-1.8	0	0.006	0.551
VANTVEER_BREAST_CANCER_POOR_PROGNOSIS	-0.5	-1.8	0	0.006	0.551
MORI_IMMATURE_B_LYMPHOCYTE_DN	-0.8	-1.8	0	0.006	0.551
BIOCARTA_MCM_PATHWAY	-0.8	-1.8	0	0.006	0.551
SLEBOS_HEAD_AND_NECK_CANCER_WITH_HPV_UP	-0.6	-1.8	0	0.006	0.573
REACTOME_MITOTIC_G1_G1_S_PHASES	-0.6	-1.8	0	0.007	0.631
WHITFIELD_CELL_CYCLE_LITERATURE	-0.9	-1.8	0	0.007	0.631
KEGG_PANCREATIC_CANCER	-0.4	-1.8	0	0.007	0.631
REACTOME_FACTORS_INVOLVED_IN_MEGAKARYOCYTE_DEVELOPMENT_AND_PLATELET_PRODUCTION	-0.4	-1.8	0	0.007	0.631
FLECHNER_PBL_KIDNEY_TRANSPLANT_OK_VS_DONOR_DN	-0.4	-1.8	0	0.007	0.631
CONCANNON_APOPTOSIS_BY_EPOXOMICIN_DN	-0.5	-1.8	0	0.007	0.631
NAKAJIMA_EOSINOPHIL	-0.6	-1.8	0	0.007	0.631
KEGG_CHRONIC_MYELOID_LEUKEMIA	-0.4	-1.8	0	0.007	0.631
KEGG_N_GLYCAN_BIOSYNTHESIS	-0.4	-1.8	0	0.007	0.631
WU_HBX_TARGETS_3_UP	-0.6	-1.8	0	0.007	0.631
REACTOME_TP53_REGULATES_TRANSCRIPTION_OF_DNA_REPAIR_GENES	-0.4	-1.8	0	0.007	0.631
REACTOME_MITOTIC_PROMETAPHASE	-0.6	-1.8	0	0.007	0.631
REACTOME_HDR_THROUGH_SINGLE_STRAND_ANNEALING_SSA	-0.7	-1.8	0	0.007	0.631
ISHIDA_E2F_TARGETS	-0.9	-1.8	0	0.007	0.631
LE_EGR2_TARGETS_UP	-0.7	-1.8	0	0.007	0.631
HOFMANN_CELL_LYMPHOMA_UP	-0.4	-1.8	0	0.007	0.631
KAMMINGA_EZH2_TARGETS	-0.8	-1.8	0	0.008	0.673
SWEET_KRAS_ONCOGENIC_SIGNATURE	-0.4	-1.8	0	0.008	0.673
REACTOME_S_PHASE	-0.6	-1.8	0	0.009	0.673
MARKEY_RB1_CHRONIC_LOF_UP	-0.5	-1.8	0	0.009	0.673
MANALO_HYPOXIA_DN	-0.6	-1.8	0	0.009	0.673
REACTOME_G1_S_SPECIFIC_TRANSCRIPTION	-0.8	-1.8	0	0.009	0.699
PID_P73PATHWAY	-0.5	-1.8	0	0.009	0.699
MOREIRA_RESPONSE_TO_TSA_UP	-0.5	-1.8	0	0.009	0.699
RODRIGUES_THYROID_CARCINOMA_ANAPLASTIC_UP	-0.4	-1.8	0	0.009	0.699
REACTOME_MITOTIC_METAPHASE_AND_ANAPHASE	-0.6	-1.8	0	0.009	0.699

REACTOME_INTERACTIONS_OF_VPR_WITH_HOST_CELLULAR_PROTEINS	-0.6	-1.8	0	0.01	0.732
REACTOME_TRANSPORT_OF_MATURE_MRNAS_DERIVED_FROM_INTRONLESS_TRANSCRIPTS	-0.6	-1.8	0	0.01	0.732
REACTOME_DNA_REPLICATION	-0.7	-1.8	0	0.01	0.732
TARTE_PLASMA_CELL_VS_PLASMABLAST_DN	-0.5	-1.8	0	0.01	0.732
SENGUPTA_NASOPHARYNGEAL_CARCINOMA_UP	-0.4	-1.8	0	0.01	0.732
BENPORATH_ES_1	-0.5	-1.8	0	0.01	0.732
REACTOME_RNA_POLYMERASE_II_PRE_TRANSCRIPTION_EVENTS	-0.4	-1.8	0	0.01	0.732
VECCHI_GASTRIC_CANCER_EARLY_UP	-0.6	-1.8	0	0.01	0.732
GEORGES_TARGETS_OF_MIR192_AND_MIR215	-0.4	-1.8	0	0.01	0.732
MORI_LARGE_PRE_BII_LYMPHOCYTE_UP	-0.8	-1.8	0	0.01	0.732
REACTOME_TRANSCRIPTIONAL_REGULATION_BY_TP53	-0.3	-1.8	0	0.01	0.732
MARIADASON_REGULATED_BY_HISTONE_ACETYLATION_DN	-0.4	-1.8	0	0.01	0.732
VANTVEER_BREAST_CANCER_METASTASIS_DN	-0.6	-1.8	0	0.01	0.732
CROONQUIST_NRAS_SIGNALING_DN	-0.8	-1.8	0	0.01	0.732
FOURNIER_ACINAR_DEVELOPMENT_LATE_2	-0.5	-1.8	0	0.01	0.732
RIZ_ERYTHROID_DIFFERENTIATION_CCNE1	-0.5	-1.8	0	0.011	0.732
MARTINEZ_RESPONSE_TO_TRABECTEDIN_DN	-0.3	-1.8	0	0.011	0.732
WINNEPENNINCKX_MELANOMA_METASTASIS_UP	-0.7	-1.7	0	0.011	0.732
GARY_CD5_TARGETS_DN	-0.4	-1.7	0	0.011	0.732
REACTOME_DNA_REPLICATION_PRE_INITIATION	-0.7	-1.7	0	0.011	0.732
GARGALOVIC_RESPONSE_TO_OXIDIZED_PHOSPHOLIPIDS_TURQUOISE_DN	-0.7	-1.7	0	0.011	0.732
REACTOME_RHO_GTPASES_ACTIVATE_FORMINS	-0.6	-1.7	0	0.012	0.732
REACTOME_PIWI_INTERACTING_RNA_PIRNA_BIOGENESIS	-0.6	-1.7	0	0.012	0.732
IVANOVA_HEMATOPOIESIS_MATURE_CELL	-0.3	-1.7	0	0.012	0.765
LI_WILMS_TUMOR_VS_FETAL_KIDNEY_1_DN	-0.6	-1.7	0	0.012	0.765
LAU_APOPTOSIS_CDKN2A_UP	-0.4	-1.7	0	0.012	0.765
KEGG_SPLICEOSOME	-0.4	-1.7	0	0.012	0.765
CHICAS_RB1_TARGETS_GROWING	-0.5	-1.7	0	0.012	0.765
REACTOME_GLYCOLYSIS	-0.5	-1.7	0	0.013	0.765
GAVIN_FOXP3_TARGETS_CLUSTER_P6	-0.7	-1.7	0	0.012	0.765
LE_NEURONAL_DIFFERENTIATION_DN	-0.7	-1.7	0	0.013	0.765
WAKASUGI_HAVE_ZNF143_BINDING_SITES	-0.6	-1.7	0	0.013	0.765
BASAKI_YBX1_TARGETS_UP	-0.6	-1.7	0	0.013	0.765
ZHAN_MULTIPLE_MYELOMA_SUBGROUPS	-0.7	-1.7	0	0.013	0.765
REACTOME_M_PHASE	-0.5	-1.7	0	0.013	0.765

REACTOME_INHIBITION_OF_THE_PROTEOLYTIC_ACTIVITY_OF_APC_C_REQUIRED_FOR_THE_ONSET_OF_ANAPHASE_BY_MITOTIC_SPINDLE_CHECKPOINT_COMPONENTS	-0.6	-1.7	0	0.013	0.765
CAFFAREL_RESPONSE_TO_THC_24HR_5_UP	-0.6	-1.7	0	0.013	0.765
TONG_INTERACT_WITH_PTTG1	-0.4	-1.7	0	0.014	0.827
ZAMORA_NOS2_TARGETS_UP	-0.6	-1.7	0	0.014	0.827
REACTOME_DNA_STRAND_ELONGATION	-0.8	-1.7	0	0.015	0.827
ALCALAY_AML_BY_NPM1_LOCALIZATION_DN	-0.5	-1.7	0	0.015	0.827
NEMETH_INFLAMMATORY_RESPONSE_LPS_UP	-0.4	-1.7	0	0.015	0.827
BREDEMEYER_RAG_SIGNALING_NOT_VIA_ATM_UP	-0.5	-1.7	0	0.016	0.855
GALLUZZI_PREVENT_MITOCHONDIAL_PERMEABILIZATION	-0.5	-1.7	0	0.016	0.875
REACTOME_APC_C:CDC20_MEDIATED_DEGRADATION_OF_CYCLIN_B	-0.6	-1.7	0	0.016	0.875
REACTOME_APC_CDC20_MEDIATED_DEGRADATION_OF_NEK2A	-0.6	-1.7	0	0.017	0.902
COATES_MACROPHAGE_M1_VS_M2_UP	-0.4	-1.7	0	0.017	0.902
BIOCARTA_GSK3_PATHWAY	-0.5	-1.7	0	0.017	0.902
IIZUKA_LIVER_CANCER_PROGRESSION_L1_G1_UP	-0.5	-1.7	0	0.017	0.902
TOMIDA_METASTASIS_UP	-0.5	-1.7	0	0.017	0.902
SUNG_METASTASIS_STROMA_DN	-0.5	-1.7	0	0.017	0.902
WHITEFORD_PEDIATRIC_CANCER_MARKERS	-0.8	-1.7	0	0.017	0.902
LUI_THYROID_CANCER_CLUSTER_1	-0.5	-1.7	0	0.017	0.902
REACTOME_MRNA_CAPPING	-0.4	-1.7	0	0.017	0.902
REACTOME_INTERCONVERSION_OF_NUCLEOTIDE_DI_AND_TRIPHOSPHATES	-0.6	-1.7	0	0.017	0.902
ZHOU_CELL_CYCLE_GENES_IN_IR_RESPONSE_24HR	-0.7	-1.7	0	0.017	0.902
REACTOME_TRANSCRIPTION_OF_E2F_TARGETS_UNDER_NEGATIVE_CONTROL_BY_DREAM_COMPLEX	-0.7	-1.7	0	0.018	0.902
GRAHAM_CML_DIVIDING_VS_NORMAL_QUIESCENT_UP	-0.7	-1.7	0	0.018	0.902
KEGG_MISMATCH_REPAIR	-0.7	-1.7	0	0.018	0.902
REACTOME_TRANSCRIPTION_OF_E2F_TARGETS_UNDER_NEGATIVE_CONTROL_BY_P107_RBL1_AND_P130_RBL2_IN_COMPLEX_WITH_HDAC1	-0.7	-1.7	0	0.018	0.902
REACTOME_DNA_REPAIR	-0.5	-1.7	0	0.018	0.902
EPPERT_PROGENITOR	-0.5	-1.7	0	0.02	0.902
PUIFFE_INVASION_INHIBITED_BY_ASCITES_UP	-0.4	-1.7	0	0.02	0.902
EGUCHI_CELL_CYCLE_RB1_TARGETS	-0.9	-1.7	0	0.02	0.902
REACTOME_DNA_DOUBLE_STRAND_BREAK_REPAIR	-0.5	-1.7	0	0.02	0.902
BERENJENO_TRANSFORMED_BY_RHOA_UP	-0.5	-1.7	0	0.02	0.902
GARCIA_TARGETS_OF_FLI1_AND_DAX1_DN	-0.5	-1.7	0	0.02	0.902

REACTOME_POLO_LIKE_KINASE_MEDIATED_EVENTS	-0.8	-1.7	0	0.021	0.902
REACTOME_CYCLIN_A_B1_B2_ASSOCIATED_EVENTS_DURING_G2_M_TRANSITION	-0.7	-1.7	0	0.021	0.902
RIZ_ERYTHROID_DIFFERENTIATION_HBZ	-0.6	-1.7	0	0.023	0.902
SASAKI_ADULT_T_CELL_LEUKEMIA	-0.5	-1.7	0	0.023	0.902
GRAHAM_NORMAL_QUIESCENT_VS_NORMAL_DIVIDING_DN	-0.8	-1.7	0	0.023	0.902
HU_GENOTOXIC_DAMAGE_4HR	-0.6	-1.7	0	0.023	0.902
MATZUK_MEIOTIC_AND_DNA_REPAIR	-0.6	-1.7	0	0.023	0.902
REACTOME_TP53_REGULATES_TRANSCRIPTION_OF_CELL_CYCLE_GENES	-0.5	-1.7	0	0.023	0.902
MORI_MATURE_B_LYMPHOCYTE_DN	-0.6	-1.7	0	0.024	0.902
XU_HGF_TARGETS_INDUCED_BY_AKT1_48HR_DN	-0.6	-1.7	0	0.025	0.97
DODD_NASOPHARYNGEAL_CARCINOMA_DN	-0.4	-1.7	0	0.025	0.97
GROSS_HYPOXIA_VIA_ELK3_AND_HIF1A_DN	-0.4	-1.7	0	0.026	0.97
REACTOME_FORMATION_OF_HIV_ELONGATION_COMPLEX_IN_THE_ABSENCE_OF_HIV_TAT	-0.4	-1.7	0	0.026	0.97
KEGG_PURINE_METABOLISM	-0.4	-1.7	0	0.026	0.97
REACTOME_METALLOPROTEASE_DUBS	-0.5	-1.7	0	0.027	0.97
BIOCARTA_ARENRF2_PATHWAY	-0.4	-1.7	0	0.027	0.97
CROONQUIST_NRAS_VS_STROMAL_STIMULATION_DN	-0.5	-1.7	0	0.027	0.97
REACTOME_ACTIVATION_OF_AMPK_DOWNSTREAM_OF_NMDARS	-0.6	-1.7	0	0.027	0.97
RHODES_UNDIFFERENTIATED_CANCER	-0.7	-1.7	0	0.027	0.97
SCHLOSSER_MYC_TARGETS_REPRESSED_BY_SERUM	-0.4	-1.7	0	0.028	0.97
KEGG_DNA_REPLICATION	-0.8	-1.7	0	0.028	0.97
MORI_PRE_BI_LYMPHOCYTE_UP	-0.7	-1.7	0	0.029	0.97
HADDAD_T_LYMPHOCYTE_AND_NK_PROGENITOR_DN	-0.5	-1.7	0	0.03	0.97
SCIAN_CELL_CYCLE_TARGETS_OF_TP53_AND_TP73_DN	-0.9	-1.7	0	0.031	0.97
REACTOME_RHO_GTPASE_EFFECTORS	-0.5	-1.7	0	0.032	0.97
YANG_BCL3_TARGETS_UP	-0.5	-1.7	0	0.031	0.97
REACTOME_DUAL_INCISION_IN_TC_NER	-0.4	-1.7	0	0.032	0.97
CAFFAREL_RESPONSE_TO_THC_24HR_5_DN	-0.5	-1.7	0	0.032	0.97
GRADE_COLON_VS_RECTAL_CANCER_DN	-0.4	-1.7	0	0.033	0.97
FINETTI_BREAST_CANCER_KINOME_RED	-1	-1.7	0	0.033	0.97
REACTOME_PROCESSING_OF_INTRONLESS_PRE_MRNAS	-0.4	-1.6	0	0.036	0.97
REACTOME_DARPP_32_EVENTS	-0.5	-1.6	0	0.036	0.97
WANG_CISPLATIN_RESPONSE_AND_XPC_UP	-0.5	-1.6	0	0.038	0.97

REACTOME_COPI_DEPENDENT_GOLGI_TO_ER_RETROGRADE_TRAFFIC	-0.5	-1.6	0	0.038	1
SMID_BREAST_CANCER_LUMINAL_A_DN	-0.9	-1.6	0	0.038	1
NAKAYAMA_SOFT_TISSUE_TUMORS_PCA2_UP	-0.6	-1.6	0	0.041	1
GENTILE_RESPONSE_CLUSTER_D3	-0.4	-1.6	0	0.042	1
REACTOME_SIGNALING_BY_RHO_GTPASES	-0.4	-1.6	0	0.042	1
GREENBAUM_E2A_TARGETS_UP	-0.8	-1.6	0	0.043	1
GARGALOVIC_RESPONSE_TO_OXIDIZED_PHOSPHOLIPIDS_GREEN_UP	-0.6	-1.6	0	0.043	1
HE_PTEN_TARGETS_UP	-0.6	-1.6	0	0.043	1
REACTOME_FORMATION_OF_THE_EARLY_ELONGATION_COMPLEX	-0.4	-1.6	0	0.043	1
WONG_EMBRYONIC_STEM_CELL_CORE	-0.6	-1.6	0	0.044	1
LY_AGING_PREMATURE_DN	-0.7	-1.6	0	0.046	1
REACTOME_HIV_INFECTION	-0.4	-1.6	0	0.046	1
CASORELLI_ACUTE_PROMYELOCYTIC_LEUKEMIA_DN	-0.4	-1.6	0	0.046	1
PID_AURORA_B_PATHWAY	-0.7	-1.6	0	0.047	1
POOLA_INVASIVE_BREAST_CANCER_UP	-0.5	-1.6	0	0.048	1
WANG_RECURRENT_LIVER_CANCER_UP	-0.4	-1.6	0	0.05	1
BIOCARTA_RAS_PATHWAY	-0.4	-1.6	0	0.05	1
TAKEDA_TARGETS_OF_NUP98_HOXA9_FUSION_3D_DN	-0.6	-1.6	0	0.05	1
MUELLER_PLURINET	-0.5	-1.6	0	0.051	1
YIH_RESPONSE_TO_ARSENITE_C3	-0.6	-1.6	0	0.05	1
KYNG_NORMAL_AGING_DN	-0.6	-1.6	0	0.053	1
REACTOME_NUCLEAR_EVENTS_KINASE_AND_TRANSCRIPTION_FACTOR_ACTIVATION	-0.5	-1.6	0	0.055	1
BIOCARTA_ATRBRCA_PATHWAY	-0.7	-1.6	0	0.055	1
WEST_ADRENOCORTICAL_TUMOR_UP	-0.4	-1.6	0	0.056	1
GOLDRATH_HOMEOSTATIC_PROLIFERATION	-0.4	-1.6	0	0.057	1
BIOCARTA_CARM_ER_PATHWAY	-0.5	-1.6	0	0.057	1
COLINA_TARGETS_OF_4EBP1_AND_4EBP2	-0.4	-1.6	0	0.057	1
PRAMOONJAGO_SOX4_TARGETS_DN	-0.5	-1.6	0	0.058	1
XU_CREBBP_TARGETS_UP	-0.5	-1.6	0	0.058	1
KANNAN_TP53_TARGETS_DN	-0.5	-1.6	0	0.059	1
NIKOLSKY_BREAST_CANCER_17Q11_Q21_AMPLICON	-0.4	-1.6	0	0.06	1
LI_WILMS_TUMOR_ANAPLASTIC_UP	-0.8	-1.6	0	0.061	1
BORCZUK_MALIGNANT_MESOTHELIOMA_UP	-0.4	-1.6	0	0.061	1
TURASHVILI_BREAST_DUCTAL_CARCINOMA_VS_LOBULAR_NORMAL_UP	-0.4	-1.6	0.046	0.06	1
REACTOME_MEIOSIS	-0.5	-1.6	0.046	0.061	1
ZHAN_MULTIPLE_MYELOMA_PR_UP	-0.9	-1.6	0	0.061	1
GOLUB_ALL_VS_AML_UP	-0.5	-1.6	0	0.061	1

BIOCARTA_EIF_PATHWAY	-0.4	-1.6	0	0.063	1
BROWNE_HCMV_INFECTION_14HR_UP	-0.3	-1.6	0	0.063	1
REACTOME_SIGNALING_BY_NTRKS	-0.3	-1.6	0	0.065	1
PUJANA_ATM_PCC_NETWORK	-0.3	-1.6	0	0.065	1
LUCAS_HNF4A_TARGETS_UP	-0.4	-1.6	0	0.065	1
CHIARADONNA_NEOPLASTIC_TRANSFORMATION_KRAS_UP	-0.4	-1.6	0	0.065	1
PID_NOTCH_PATHWAY	-0.4	-1.6	0	0.065	1
REACTOME_CONVERSION_FROM_APC_C:CDC20_TO_APC_C:CDH1_IN_LATE_ANAPHASE	-0.5	-1.6	0	0.065	1
TIEN_INTESTINE_PROBIOTICS_24HR_UP	-0.5	-1.6	0	0.065	1
NEMETH_INFLAMMATORY_RESPONSE_LPS_DN	-0.4	-1.6	0	0.065	1
RASHI_RESPONSE_TO_IONIZING_RADIATION_5	-0.3	-1.6	0	0.068	1
SEIDEN_ONCOGENESIS_BY_MET	-0.3	-1.6	0	0.069	1
IVANOVA_HEMATOPOIESIS_LATE_PROGENITOR	-0.3	-1.6	0	0.069	1
REACTOME_ARACHIDONIC_ACID_METABOLISM	-0.4	-1.6	0	0.071	1
BHATTACHARYA_EMBRYONIC_STEM_CELL	-0.5	-1.6	0	0.073	1
CHIN_BREAST_CANCER_COPY_NUMBER_UP	-0.4	-1.6	0	0.075	1
LY_AGING_OLD_DN	-0.6	-1.6	0	0.075	1
SIMBULAN_PARP1_TARGETS_DN	-0.7	-1.6	0	0.076	1
CHICAS_RB1_TARGETS_SENESCENT	-0.3	-1.6	0	0.076	1
TARTE_PLASMA_CELL_VS_B_LYMPHOCYTE_UP	-0.5	-1.6	0	0.076	1
REACTOME_LAGGING_STRAND_SYNTHESIS	-0.8	-1.6	0	0.076	1
BIOCARTA_STATHMIN_PATHWAY	-0.6	-1.6	0	0.079	1
BECKER_TAMOXIFEN_RESISTANCE_UP	-0.5	-1.6	0	0.079	1
LEE_BMP2_TARGETS_DN	-0.4	-1.6	0	0.081	1
REACTOME_CHROMOSOME_MAINTENANCE	-0.7	-1.6	0	0.084	1
GRADE_COLON_AND_RECTAL_CANCER_UP	-0.4	-1.6	0	0.086	1
YAMAZAKI_TCEB3_TARGETS_DN	-0.4	-1.6	0	0.087	1
BIOCARTA_EFP_PATHWAY	-0.6	-1.6	0.045	0.084	1
MEINHOLD_OVARIAN_CANCER_LOW_GRADE_DN	-0.7	-1.5	0	0.088	1
BURTON_ADIPOGENESIS_PEAK_AT_24HR	-0.8	-1.5	0	0.088	1
REACTOME_GLUCOSE_METABOLISM	-0.5	-1.5	0	0.088	1
REACTOME_DISEASES_ASSOCIATED_WITH_GLYCOSYLATION_PRECURSOR_BIOSYNTHESIS	-0.5	-1.5	0	0.09	1
DUNNE_TARGETS_OF_AML1_MTG8_FUSION_DN	-0.6	-1.5	0.043	0.091	1
KEGG_PATHOGENIC_ESCHERICHIA_COLI_INFECTION	-0.4	-1.5	0	0.096	1
GARGALOVIC_RESPONSE_TO_OXIDIZED_PHOSPHOLIPIDS_RED_DN	-0.5	-1.5	0	0.097	1
SANSOM_APC_TARGETS_UP	-0.4	-1.5	0	0.098	1
BIOCARTA_MPR_PATHWAY	-0.6	-1.5	0	0.1	1
REACTOME_GLYCOGEN_METABOLISM	-0.5	-1.5	0.046	0.097	1
REACTOME_REPRODUCTION	-0.5	-1.5	0.047	0.097	1

TAKEDA_TARGETS_OF_NUP98_HOXA9_FUSION_10D_DN	-0.4	-1.5	0	0.102	1
FOURNIER_ACINAR_DEVELOPMENT_LATE_DN	-0.8	-1.5	0	0.104	1
XU_HGF_SIGNALING_NOT_VIA_AKT1_48HR_DN	-0.7	-1.5	0	0.104	1
PID_CASPASE_PATHWAY	-0.4	-1.5	0	0.106	1
ZHANG_RESPONSE_TO_IKK_INHIBITOR_AND_TNF_DN	-0.4	-1.5	0	0.107	1
REACTOME_METABOLISM_OF_COFACTORS	-0.6	-1.5	0	0.108	1
KRIEG_HYPOXIA_NOT_VIA_KDM3A	-0.3	-1.5	0	0.108	1
ROESSLER_LIVER_CANCER_METASTASIS_DN	-0.4	-1.5	0	0.108	1
PID_MAPK_TRK_PATHWAY	-0.3	-1.5	0	0.108	1
LIU_BREAST_CANCER	-0.4	-1.5	0	0.109	1
ROZANOV_MMP14_TARGETS_DN	-0.4	-1.5	0	0.112	1
REACTOME_TELOMERE_C_STRAND_LAGGING_STRAND_SYNTHESIS	-0.7	-1.5	0	0.114	1
SANSOM_APC_MYC_TARGETS	-0.3	-1.5	0	0.114	1
GROSS_HYPOXIA_VIA_ELK3_ONLY_DN	-0.4	-1.5	0	0.114	1
KRASNOSELSKAYA_ILF3_TARGETS_DN	-0.4	-1.5	0	0.114	1
SHETH_LIVER_CANCER_VS_TXNIP_LOSS_PAM6	-0.4	-1.5	0	0.114	1
PATIL_LIVER_CANCER	-0.4	-1.5	0	0.114	1
REACTOME_PCNA_DEPENDENT_LONG_PATCH_BASE_EXCISION_REPAIR	-0.7	-1.5	0	0.114	1
WEI_MYCN_TARGETS_WITH_E_BOX	-0.4	-1.5	0	0.114	1
MULLIGHAN_NPM1_MUTATED_SIGNATURE_2_UP	-0.3	-1.5	0	0.117	1
REACTOME_NUCLEOSOME_ASSEMBLY	-0.7	-1.5	0	0.12	1
PID_BARD1_PATHWAY	-0.7	-1.5	0	0.123	1
REACTOME_NUCLEOTIDE_SALVAGE	-0.6	-1.5	0	0.126	1
REACTOME_RESOLUTION_OF_AP_SITES_VIA_THE_MULTIPLE_NUCLEOTIDE_PATCH_REPLACEMENT_PATHWAY	-0.6	-1.5	0.047	0.125	1
BROWNE_HCMV_INFECTION_24HR_UP	-0.3	-1.5	0	0.127	1
FLOTHO_PEDIATRIC_ALL_THERAPY_RESPONSE_DN	-0.5	-1.5	0	0.129	1
RHEIN_ALL_GLUCOCORTICOID_THERAPY_DN	-0.5	-1.5	0	0.134	1
WANG_METASTASIS_OF_BREAST_CANCER_ESR1_UP	-0.6	-1.5	0.046	0.132	1
BROWNE_HCMV_INFECTION_8HR_DN	-0.5	-1.5	0	0.135	1
FERRANDO_HOX11_NEIGHBORS	-0.8	-1.5	0	0.137	1
REACTOME_TRANSCRIPTION_COUPLED_NUCLEOTIDE_EXCISION_REPAIR_TC_NER	-0.4	-1.5	0	0.139	1
KOKKINAKIS_METHIONINE_DEPRIVATION_48HR_DN	-0.4	-1.5	0	0.139	1
REACTOME_RECYCLING_PATHWAY_OF_L1	-0.4	-1.5	0	0.139	1
GAZIN_EPIGENETIC_SILENCING_BY_KRAS	-0.5	-1.5	0	0.139	1
REACTOME_CELLULAR_SENESCENCE	-0.4	-1.5	0.045	0.139	1

PEDERSEN_METASTASIS_BY_ERBB2_ISOFORM_7	-0.3	-1.5	0	0.145	1
SCHUHMACHER_MYC_TARGETS_UP	-0.5	-1.5	0	0.146	1
REACTOME_EXTENSION_OF_TELOMERES	-0.7	-1.5	0	0.148	1
REACTOME_VESICLE_MEDIATED_TRANSPORT	-0.2	-1.5	0	0.148	1
VIETOR_IFRD1_TARGETS	-0.6	-1.5	0	0.148	1
ZHANG_RESPONSE_TO_CANTHARIDIN_DN	-0.5	-1.5	0	0.149	1
REACTOME_PRE_NOTCH_EXPRESSION_AND_PROCESSING	-0.4	-1.5	0	0.149	1
BIOCARTA_FAS_PATHWAY	-0.4	-1.5	0	0.149	1
PEART_HDAC_PROLIFERATION_CLUSTER_UP	-0.4	-1.5	0	0.149	1
BIOCARTA_GPCR_PATHWAY	-0.4	-1.5	0	0.15	1
BENPORATH_MYC_MAX_TARGETS	-0.3	-1.5	0	0.153	1
PID_AURORA_A_PATHWAY	-0.6	-1.5	0	0.154	1
WIERENGA_PML_INTERACTOME	-0.4	-1.5	0	0.154	1
REACTOME_GOLGI_TO_ER_RETROGRADE_TRANSPORT	-0.4	-1.5	0	0.154	1
AUNG_GASTRIC_CANCER	-0.4	-1.5	0	0.155	1
HERNANDEZ_MITOTIC_ARREST_BY_DOCETAXEL_1_DN	-0.5	-1.5	0	0.158	1
OUELLET_OVARIAN_CANCER_INVASIVE_VS_LMP_UP	-0.4	-1.5	0	0.158	1
REACTOME_DISEASES_ASSOCIATED_WITH_N_GLYCOSYLATION_OF_PROTEINS	-0.6	-1.5	0	0.161	1
REACTOME_RNA_POLYMERASE_II_TRANSCRIPTION_ELONGATION	-0.3	-1.5	0	0.164	1
SCHLOSSER_MYC_TARGETS_AND_SERUM_RESPONSE_DN	-0.5	-1.5	0	0.168	1
HAHTOLA_SEZARY_SYNDROM_UP	-0.4	-1.5	0	0.17	1
LEE_LIVER_CANCER_MYC_UP	-0.4	-1.4	0	0.174	1
BIOCARTA_ARF_PATHWAY	-0.5	-1.4	0	0.174	1
KUMAR_PATHOGEN_LOAD_BY_MACROPHAGES	-0.3	-1.4	0	0.177	1
BIOCARTA_RAC1_PATHWAY	-0.4	-1.4	0.044	0.18	1
YEGNASUBRAMANIAN_PROSTATE_CANCER	-0.4	-1.4	0.048	0.179	1
LIU_SOX4_TARGETS_DN	-0.3	-1.4	0	0.182	1
REACTOME_SYNTHESIS_OF_PIPS_AT_THE_EARLY_ENDOSOME_MEMBRANE	-0.4	-1.4	0	0.191	1
NAKAMURA_TUMOR_ZONE_PERIPHERAL_VS_CENTRAL_UP	-0.4	-1.4	0	0.191	1
WARTERS_IR_RESPONSE_5GY	-0.4	-1.4	0	0.193	1
CHEN_NEUROBLASTOMA_COPY_NUMBER_GAINS	-0.5	-1.4	0.043	0.188	1
BIOCARTA_MITOCHONDRIA_PATHWAY	-0.5	-1.4	0	0.2	1
KIM_WT1_TARGETS_12HR_DN	-0.3	-1.4	0	0.202	1
WILLIAMS_ESR1_TARGETS_UP	-0.6	-1.4	0	0.205	1
MATTIOLI_MGUS_VS_PCL	-0.4	-1.4	0	0.205	1

PAL_PRMT5_TARGETS_UP	-0.4	-1.4	0.046	0.196	1
RHODES_CANCER_META_SIGNATURE	-0.5	-1.4	0.046	0.203	1
KEGG_BASE_EXCISION_REPAIR	-0.5	-1.4	0.047	0.196	1
PROVENZANI_METASTASIS_UP	-0.3	-1.4	0	0.208	1
TAKEDA_TARGETS_OF_NUP98_HOXA9_FUSION_8D_DN	-0.4	-1.4	0	0.209	1
REACTOME_METABOLISM_OF_NUCLEOTIDES	-0.4	-1.4	0	0.209	1
JAEGER_METASTASIS_UP	-0.5	-1.4	0	0.21	1
MARSON_BOUND_BY_FOXP3_UNSTIMULATED	-0.3	-1.4	0	0.214	1
BAELDE_DIABETIC_NEPHROPATHY_UP	-0.4	-1.4	0	0.221	1
KEGG_NON_SMALL_CELL_LUNG_CANCER	-0.3	-1.4	0	0.222	1
WANG_ADIPOGENIC_GENES_REPRESSED_BY_SIRT1	-0.6	-1.4	0	0.234	1
MCCLUNG_COCAIN_REWARD_4WK	-0.4	-1.4	0	0.237	1
REACTOME_SIGNALING_BY_NTRK1_TRKA	-0.3	-1.4	0.043	0.228	1
REACTOME_CARGO_RECOGNITION_FOR_CLATHRIN_MEDIATED_ENDOCYTOSIS	-0.3	-1.4	0.044	0.234	1
SESTO_RESPONSE_TO_UV_C7	-0.5	-1.4	0	0.241	1
GRAESSMANN_RESPONSE_TO_MC_AND_DOXORUBICIN_DN	-0.3	-1.4	0	0.241	1
DANG_BOUND_BY_MYC	-0.3	-1.4	0	0.246	1
KEGG_AMINO_SUGAR_AND_NUCLEOTIDE_SUGAR_METABOLISM	-0.5	-1.4	0	0.246	1
LU_EZH2_TARGETS_DN	-0.3	-1.4	0	0.247	1
REACTOME_TERMINATION_OF_TRANSLESION_DNA_SYNTHESIS	-0.6	-1.4	0	0.247	1
BIDUS_METASTASIS_DN	-0.3	-1.4	0	0.246	1
KENNY_CTNNB1_TARGETS_UP	-0.4	-1.4	0.047	0.241	1
BROWNE_HCMV_INFECTION_30MIN_DN	-0.4	-1.4	0	0.249	1
TAYLOR_METHYLATED_IN_ACUTE_LYMPHOBLASTIC_LEUKEMIA	-0.3	-1.4	0	0.256	1
REACTOME_INTRA_GOLGI_AND_RETROGRADE_GOLGI_TO_ER_TRAFFIC	-0.3	-1.4	0	0.259	1
KATSANOU_ELAVL1_TARGETS_DN	-0.3	-1.4	0	0.263	1
THEILGAARD_NEUTROPHIL_AT_SKIN_WOUND_UP	-0.3	-1.4	0	0.264	1
TURASHVILI_BREAST_CARCINOMA_DUCTAL_VS_LOBULAR_UP	-0.5	-1.4	0	0.273	1
ZWANG_DOWN_BY_2ND_EGF_PULSE	-0.3	-1.4	0	0.283	1
REACTOME_RNA_POLYMERASE_II_TRANSCRIBES_SNRNA_GENES	-0.3	-1.4	0	0.282	1
REACTOME_GAP_FILLING_DNA_REPAIR_SYNTHESIS_AND_LIGATION_IN_GG_NER	-0.6	-1.4	0.047	0.271	1
HAHTOLA_MYCOSIS_FUNGOIDES_SKIN_UP	-0.2	-1.3	0	0.284	1
BUYTAERT_PHOTODYNAMIC_THERAPY_STRESS_DN	-0.3	-1.3	0	0.284	1

CHIANG_LIVER_CANCER_SUBCLASS_UNANNOTATED_DN	-0.5	-1.3	0	0.289	1
FARMER_BREAST_CANCER_APOCRINE_VS_BASAL	-0.3	-1.3	0	0.29	1
REACTOME_CLATHRIN_MEDIATED_ENDOCYTOSIS	-0.3	-1.3	0.042	0.285	1
TORCHIA_TARGETS_OF_EWSR1_FLI1_FUSION_DN	-0.3	-1.3	0	0.299	1
KEGG_INSULIN_SIGNALING_PATHWAY	-0.3	-1.3	0.047	0.304	1
HOFFMANN_IMMATURE_TO_MATURE_B_LYMPHOCYTE_DN	-0.3	-1.3	0	0.319	1
STEIN_ESRRA_TARGETS_RESPONSIVE_TO_ESTROGEN_UP	-0.4	-1.3	0.046	0.307	1
ONDER_CDH1_TARGETS_1_DN	-0.3	-1.3	0.04	0.321	1
KYNG_WERNER_SYNDROM_AND_NORMAL_AGING_UP	-0.3	-1.3	0.045	0.32	1
VANLOO_SP3_TARGETS_DN	-0.4	-1.3	0.049	0.322	1
FARMER_BREAST_CANCER_BASAL_VS_LULMINAL	-0.3	-1.3	0	0.334	1
BENPORATH_ES_2	-0.6	-1.3	0	0.336	1
BILD_E2F3_ONCOGENIC_SIGNATURE	-0.3	-1.3	0	0.34	1
BROWNE_HCMV_INFECTION_14HR_DN	-0.3	-1.3	0	0.342	1
SU_LIVER	-0.4	-1.3	0.044	0.343	1
WANG_LMO4_TARGETS_UP	-0.2	-1.3	0	0.352	1
WANG_SMARCE1_TARGETS_DN	-0.3	-1.3	0	0.357	1
ALFANO_MYC_TARGETS	-0.3	-1.3	0	0.366	1
BRUINS_UVC_RESPONSE_EARLY_LATE	-0.3	-1.3	0	0.376	1
REACTOME_ACTIVATION_OF_NMDA_RECEPTORS_AND_POST SYNAPTIC_EVENTS	-0.3	-1.3	0	0.398	1
MATSUDA_NATURAL_KILLER_DIFFERENTIATION	-0.3	-1.3	0	0.401	1
REACTOME_SIALIC_ACID_METABOLISM	-0.4	-1.3	0.046	0.398	1
REACTOME_GENE_EXPRESSION_TRANSCRIPTION	-0.2	-1.2	0	0.433	1
HELLER_HDAC_TARGETS_DN	-0.2	-1.2	0	0.436	1
REACTOME_GENERIC_TRANSCRIPTION_PATHWAY	-0.2	-1.2	0	0.511	1
